# T Cell Leukemia/Lymphoma 1A is essential for mouse epidermal keratinocytes proliferation promoted by insulin-like growth factor 1

**DOI:** 10.1371/journal.pone.0204775

**Published:** 2018-10-04

**Authors:** Antonella Bresin, Gianluca Ragone, Cristina Cristofoletti, Diego Arcelli, Cristian Bassi, Elisabetta Caprini, Maria Teresa Fiorenza, Mauro Helmer Citterich, Giandomenico Russo, Maria Grazia Narducci

**Affiliations:** 1 Molecular Oncology Laboratory, Istituto Dermopatico dell'Immacolata, IDI-IRCCS, Rome, Italy; 2 Department of Morphology, Surgery and Experimental Medicine, Università degli Studi di Ferrara, Ferrara, Italy; 3 Sezione di Neuroscienze, Dipartimento di Psicologia, Università degli studi di Roma La Sapienza, Rome, Italy; IRCCS-Policlinico San Donato, ITALY

## Abstract

T Cell Leukemia/Lymphoma 1A is expressed during B-cell differentiation and, when over-expressed, acts as an oncogene in mouse (*Tcl1a*) and human (*TCL1A*) B-cell chronic lymphocytic leukemia (B-CLL) and T-cell prolymphocytic leukemia (T-PLL). Furthermore, in the murine system *Tcl1a* is expressed in the ovary, testis and in pre-implantation embryos, where it plays an important role in blastomere proliferation and in embryonic stem cell (ESC) proliferation and self-renewal. We have also observed that *Tcl1-/-* adult mice exhibit alopecia and deep ulcerations. This finding has led us to investigate the role of TCL1 in mouse skin and hair follicles. We have found that TCL1 is expressed in the proliferative structure (i.e. the secondary hair germ) and in the stem cell niche (i.e. the bulge) of the hair follicle during regeneration phase and it is constitutively expressed in the basal layer of epidermis where it is required for the correct proliferative–differentiation program of the keratinocytes (KCs). Taking advantage of the murine models we have generated, including the *Tcl1-/-* and the *K14-TCL1* transgenic mouse, we have analysed the function of TCL1 in mouse KCs and the molecular pathways involved. We provide evidence that in the epidermal compartment TCL1 has a role in the regulation of KC proliferation, differentiation, and apoptosis. In particular, the colony-forming efficiency (CFE) and the insulin-like growth factor 1 (IGF1)-induced proliferation are dramatically impaired, while apoptosis is increased, in KCs from *Tcl1-/-* mice when compared to WT. Moreover, the expression of differentiation markers such as cytokeratin 6 (KRT6), filaggrin (FLG) and involucrin (IVL) are profoundly altered in mutant mice (*Tcl1-/-*). Importantly, by over-expressing *TCL1A* in basal KCs of the *K14-TCL1* transgenic mouse model, we observed a significant rescue of cell proliferation, differentiation and apoptosis of the mutant phenotype. Finally, we found TCL1 to act, at least in part, via increasing phospho-ERK1/2 and decreasing phospho-P38 MAPK. Hence, our data demonstrate that regulated levels of *Tcl1a* are necessary for the correct proliferation and differentiation of the interfollicular KCs.

## Introduction

Skin homeostasis is a complex process in which epidermal stem cells (SCs) self-renew and generate daughter cells, which proliferate, undergo terminal differentiation and eventually die. Among the genes regulating epidermal homeostasis, we have previously described the role of the *Tcl1a* gene in a mouse model [1]. *TCL1A* has been initially isolated as an oncogene involved in human T-PLL and it is widely studied in B-CLL [2–8], albeit *Tcl1-/-* mice show a mild impairment of B- and T-cell differentiation [9]. *TCL1A* overexpression in cancer or in mouse models is tightly associated to proliferative/pro-survival conditions [10–12]. Further, TCL1 affects the proliferation/differentiation balance, the self-renewal ability and pluripotency of murine embryonic stem cells (ESC) [13–18] and it is highly expressed in pre-implantation embryo, allowing the progression beyond the 4-cell stage [19, 20].

In our previous study, we have shown that TCL1 affects both hair growth and epidermis integrity [1]. We found that *Tcl1a* loss led to the proliferation impairment of transient amplifying (TA) cells, in the regenerative phase of hair follicle, and to the lack of expression of the SC marker CD34 in the bulge (i.e., the SC niche), thus affecting the ability to incorporate BrdU in the slow-cycling SC and in TA cells. Additionally, *Tcl1a* was found to be expressed in the basal layer of mouse epidermis, where its loss induced a differentiation/proliferation imbalance, resulting in the aberrant co-expression of proliferation and apoptotic markers in the same cell. As a consequence, adult *Tcl1-/-* mice developed skin defects, represented by early alopecia and late ulcerations. Interestingly, the mutant phenotype was almost completely rescued by epidermal *K14* promoter-driven *TCL1A* over-expression, *in vivo* [1].

TCL1 is known to enhance AKT Ser-473 phosphorylation and its nuclear translocation, both in humans and in mice [21–24]. Furthermore, TCL1 acts as an NFkB activator, through the interaction with p300, and as an inhibitor of AP1-dependent transcription, through the direct interaction with cFOS and cJUN [25].

In this study we used the gene expression profile (GEP) analysis to unravel the pathways that are affected by *Tcl1a* loss-of-function in epidermal keratinocytes (KCs) by comparing the *Tcl1-/-* and the WT mouse models. Furthermore, we evaluated the reactivation of TCL1 in the basal layer of epidermis in the *K14-TCL1* mouse model for its ability to rescue the aberrant phenotype, both at the molecular and functional levels. We especially focused on proliferation/differentiation/apoptosis functions and our findings show that TCL1 affects several growth factor-induced pathways with the RAS-MAPK pathway resulting strongly involved. In particular, we found that TCL1 is essential for IGF1-induced proliferation of primary KCs and for their clonogenic ability. Additionally, cleavage of apoptosis-related proteins such as Caspase 3 (CASP3) and Poly(ADP-ribose) polymerase 1 (PARP1) is increased in *Tcl1-/-* KCs. Alterations in the expression pattern of differentiation markers, such as KRT6, FLG and IVL, are also described. Interestingly, restoring the *TCL1A* expression in the *K14-TCL1* mouse model almost completely reverts the aberrant phenotype.

## Materials and methods

### Animals

*Wild Type* C57bl/6 (WT), *Tcl1-/-* and *K14-TCL1;Tcl1-/-* (*K14-TCL1*) mice were studied [1]. All experimental procedures, animal care and housing were conformed to European(Directive 86/609/ECC and 2010/63/UE) and Italian (D.L. 116/92 and D.L. 26/2014) laws on theuse of animals in scientific research Animal experiments were approved by the IDI-IRCCS Animal Care and Use Committee ‘Organismo Preposto al Benessere Animale’ (OPBA) and the protocol was approved by the Italian Ministry of Health. All efforts were made to minimize suffering.

### RNA isolation

For gene expression experiments, skin patches of 1 cm^2^ were harvested three days’ post-depilation (3dpd). The skin biopsy was excised and placed in PBS containing 1U/mL of dispase (BD Biosciences, Belgium) and incubated overnight at 4°C in gentle fluctuation. The epidermal sheet was then separated from the dermis using sterile forceps and placed in 1mL of TRIZOL Reagent (Life Technologies, UK). Total RNA was extracted following manufacturer’s protocol. RNA quality was checked through agarose gel and quantification was obtained using NanoDrop spectrophotometer (Thermo Fisher Scientific, MA USA).

### Microarray expression profile

Biotinylated cRNAs probes were prepared and hybridized to pan-genomic Affymetrix Mouse Genome 430 2.0 arrays, as per manufacturer protocol (Affymetrix, Santa Clara, CA USA). Three animals and three independent chips were used for each genotype. Unsupervised hierarchical analyses were performed using the BRB-Array Tools software (Biometric Research Branch, National Cancer Institute) [26] and Partek Genomic Suitesoftware (Partek Inc. St. Louis, MO, USA) for principal component analysis (PCA). To identify genes that were differentially expressed between WT, *Tcl1-/-* and *K14–TCL1* groups supervised analysis was performed using ‘class comparison tools’ of BRB array tools. The random variance model was applied to the filtered data sets. Genes with p-values less than 0.001 were considered statistically significant. Moreover, genes were excluded if their expression was less than 1.4 fold from gene’s median value in at least 20% of the samples. The false discovery rate (FDR) was also estimated for each gene using the method of Benjamini and Hochberg, to control for false positives [27]. Gene ontology (GO) and pathway analysis used two-sample t tests and random variance model. Significant gene sets were found using LS/KS permutation test and Efron-Tibshirani's GSA maxmean test. For gene categories identified by GO the threshold was P<0.005. For pathway analysis, the threshold of determining significant gene sets was P<0.05. Gene sets are defined by the Biocarta Pathway (https://cgap.nci.nih.gov/Pathways/BioCarta). Pathways and GO analysis have been performed using the “Gene Set Expression Comparison” tool of BRB array tools. According to the PLOS Data Availability policy, microarray data are deposited at GEO (accession number GSE118345).

### Real time PCR

Real Time quantification of target mRNAs relative to RNA polymerase II (RPII) mRNA was performed with a SYBR Green 2X SensiMix One-Step Kits (Quantace, UK), using the ABI PRISM 7000 detection system (Applied Biosystems, UK). cDNAs were made using 1μg total RNA, 0.5μg oligo dT, 1μl ImProm-II Reverse Transcriptase (Promega, WI USA). 3μl of cDNA diluted 1:7 were added in a 25μl total reaction mix. Primers for target genes (*Krt6*; *cFos*; *Gjb*2; *Dio2*; *Dusp1*; *Epgn*; *Rptn*; *S100a*9; *Sprr1b*; *Stk25*) and reference gene (*RPII*) (MWG, Biotech, Germany) were optimized for a final concentration of 50nM and are listed in S1 Table. The following experimental run protocol was used: initial denaturation 95°C for 10 min followed by 40 cycles of denaturation 95°C for 15sec and annealing 60°C for 1min. RT-PCR products, analyzed by gel electrophoresis, resulted in a single product of the expected length. In addition, a melting curve analysis was performed, which confirmed the specificity of the reaction and the absence of primer dimers. Relative gene expression was quantified as described in User Bulletin #2 for the ABI PRISM 7000.

### Cell culture

Mouse KCs were isolated from the skin of newborn mice, as described [28], except that the epidermis was dissociated following 0.05% DNase addition (Sigma-Aldrich, Italy). 5*10^5 KCs were plated in T25 cell culture flask coated with mouse type IV collagen (BD Biosciences, Belgium) and grown in Cnt-57 progenitor cell-targeted medium (CELLnTEC, Switzerland). At subconfluence, cells were detached using TrypLE enzyme (Thermo Fisher Scientific, MA USA) and either used for the experiments, or seeded for further passages at 2*10^5 cells in 5 mL medium.

### Kinase assay

AKT kinase assay was performed following the manufacturer’s instruction (CycLex CY-1168, MBL, Japan). Briefly, equal amounts of keratinocyte extract were added to the plate wells together with ATP-containing kinase reaction buffer. After 60 minutes of incubation, wells were washed and then were incubated with HRP-conjugated antibody for additional 60 minutes. After washing, wells were incubated with substrate reagent for 10 minutes. Finally, stop solution was added and absorbance was measured using a plate reader at dual wavelengths of 450/540 nm (BIO-RAD model 680).

### Western blot analysis (WB)

1-cm^2^ epidermal samples were obtained as described for RNA isolation, lysed in RIPA buffer and boiled in SDS sample buffer. 30-μg protein were separated in SDS-PAGE gels and transferred to nitrocellulose membranes (BIO-RAD). Membranes were incubated overnight at 4°C with primary antibodies followed by 1-hour incubation at room temperature with appropriate HRP-conjugated secondary antibodies (Santa Cruz Biotechnology). Immuno-detection was enhanced by chemiluminescence detection reagents (Thermo Scientific Pierce). Films were scanned on a GS710 Calibrated Imaging Densitometer and absorbance of the bands was measured by means of Quantity One software version 4.1.1 (BIO-RAD). Antibody used are listed in the S2 Table.

### Immunofluorescence

Skin biopsies of 1 cm^2^ were excised 3dpd, embedded in cryostat medium Killik (Bio-Optica) and rapidly frozen. 5μm sections were cut, processed as described [1] and incubated overnight at 4°C with anti-TCL1 (5A4 rat monoclonal antibody from Areta International), anti-phospho-AKT (9271 Cell Signaling Technology), anti-cFOS (PC38 Calbiochem-MERK), anti-cJUN (610326 BD biosciences), anti-phospho-cJUN (3270 Cell Signaling Technology), anti-KRT6A (PRB-169P) or anti-KRT10 (PRB-159P Covance-Biolegend). FITC- or CY3- or CY5-conjugated secondary antibodies were all from Jackson ImmunoResearch. The specimens were observed by laser scanning confocal microscopy using a Zeiss LSM 510 Meta microscope.

### Colony-forming efficiency assay (CFE)

Freshly isolated KCs were low-density seeded in 6-well plates (500 cells/well in triplicate) and cultured for two weeks. Cells were than fixed with 2% paraformaldehyde for 15 minutes and stained with 1.5% Rhodamine B in water for 20 minutes. Colonies were counted and average number of three independent experiments were graphed. In addition, the percentage of total number of colonies developed from *Tcl1-/-*, with respect to WT or *K14–TCL1* cells was calculated for each passage.

### Proliferation assay

Cryopreserved p0 KCs were plated in T25 cell culture flask. At p1 passage 0.5x10^4^ cells were seeded in 96-well plates, in triplicate. Subconfluent cells were starved for 16 hours in Cnt-57 basal medium (CELLnTEC, Switzerland), and then cultured with either 100 ng ml^-1^ of mouse recombinant IGF-1 (Peprotech, UK) in basal medium, basal medium alone or complete medium. After 24 hours, cells were fixed with 3% formaldehyde in PBS, then washed and stained with 0.5% crystal violet solution for 30 minutes at RT. Samples were eluted with 100 μl of 0.1M Na citrate in 50% ethanol pH 4.2 and Optical Density (OD) was measured at 595 nm using a microplate reader BIO-RAD Model 680. For each treatment, the differences in cell proliferation between the three genotypes were evaluated as the ratio of OD values compared to the WT. In addition, the effect of different treatments on the proliferation was evaluated, for each genotype, as the OD ratio of the complete- or IGF1-treated vs basal value.

### Statistical analysis

Statistical analysis was performed using GraphPad Prism 6 software. For CFE and proliferation assays, statistical significance was determined by multiple T-test using the Holm-Sidak method, with alpha = 5.000%. For Western blot analysis, significance was calculated by paired T-test. Gene expression experiments were analyzed as described in the ‘microarray expression profile’ paragraph of this section.

## Results

### The epidermis of *Tcl1-/-* mice shows defective proliferation and differentiation signalling, as revealed by gene expression profiling (GEP)

In order to identify the molecular mechanisms involved in TCL1 function on skin homeostasis of adult mice, we used pan-genomic Affymetrix Mouse Genome 430 2.0 arrays to generate GEP of epidermal KCs obtained from WT, *Tcl1-/-* and K14-*TCL1* mouse models [1].

#### The GEP of *K14-TCL1* KCs is more similar to the WT than to *Tcl1-/-*

**Fig 1:**
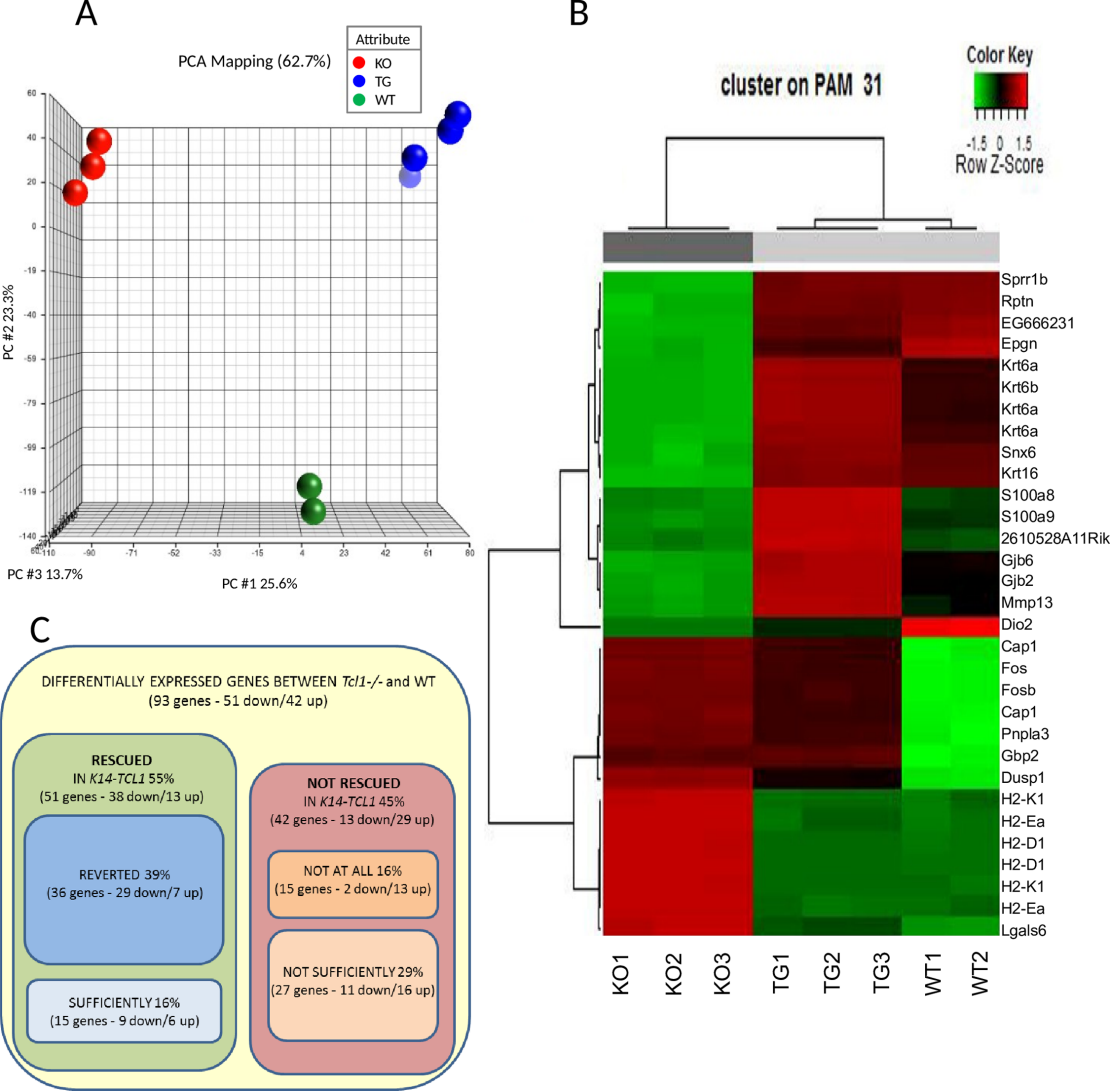
GEP analysis of the WT, *Tcl1-/-* and *K14-TCL1* keratinocytes. (A) PCA analysis obtained by Partek GS on the totality of the genes shows that *K14-TCL1* (TG, blue spheres), *Tcl1-/-* (KO, red spheres) and WT (green spheres) samples stay as three well-separated groups. (B) Unsupervised hierarchical analysis by R on the most significant differentially expressed genes (31 genes, *P*<1e-07) shows that *K14-TCL1* KCs are more similar to WT than to *Tcl1-/-* ones. (C) Percentage of “rescued” and “not rescued” genes in the *K14-TCL1* KCs among the 93 most significant and most differentially expressed genes between *Tcl1-/-* and WT KCs. See the text for further explanation.

Principal components analysis (PCA) of the three groups, using Partek software, reveals clearly distinct clustering among them (Fig 1A), confirming a major role for *Tcl1a* in the epidermis. Moreover, unsupervised hierarchical cluster analysis, using BRB software, shows that the GEP of mice carrying the *K14*-*TCL1* transgene is more similar to the WT than to the *Tcl1-/-* one (Fig 1B). Supervised analysis generated a list of 271 genes differentially expressed between WT, *Tcl1-/-* and *K14-TCL1* KCs (S3 Table, *P*<0.001). Two-sample comparison was also performed in WT vs *Tcl1-/-*, WT vs *K14-TCL1*, and *Tcl1-/-* vs *K14-TCL1* mice (S4–S6 Tables, respectively). The results of gene expression experiments were validated by real-time PCR for 10 genes, randomly selected among the most up- or under-regulated ones (S1 Table).

To evaluate at what extent the restoration of *TCL1A* expression in the basal KCs is able to rescue the *Tcl1-/-* GEP at the WT level, we performed an overall analysis of the genes listed in S3 Table. We assigned each gene to the “rescued” or “not rescued” group (Fig 1C), based on the following criteria: first, we restricted the analysis to the 93 genes with a FDR score lower than 1.00e-05 and a fold change (FC) higher than 1.5 or lower than 0.75, regarding the *Tcl1-/-* vs WT ratio; second, on the bases of pairwise significance, we considered “rescued” the genes that were not significantly different between WT and *K14-TCL1* KCs, indicating they were reverted at physiologic level, and “not rescued” the genes that were not significantly different between *Tcl1-/-* and *K14-TCL1* (16 and 15 genes, respectively). Additional 20 genes were considered “rescued” as their expression was reverted in the *K14-TCL1* compared to the *Tcl1-/-* although excessively, compared to the WT, most likely due to the over-expression of the transgene. The remaining 42 genes were expressed in the *K14-TCL1* KCs at an intermediate level between the WT and the *Tcl1-/-*. Among these, we arbitrarily considered the expression of the *K14-TCL1* KCs’ genes to be “sufficiently” similar to the WT when the ratio to WT was lower than the ratio to *Tcl1-/-*. In the last column of the S3 Table, the assignment for each gene to the corresponding group is reported. These data indicate the *Tcl1a* loss to affect the expression of several genes in the mouse epidermis and the restoring of *TCL1A* expression in the basal layer to rescue 55% of these genes (Fig 1C).

#### Gene ontology (GO) and pathway analyses define functional and molecular defects in *Tcl1-/-* KCs

GO and pathway analyses were used to investigate the biological mechanisms that might be influenced by *Tcl1a* loss- or gain-of-function. GO analysis was conducted with a 0.005 significance threshold for the gene sets. Among the GO terms, we focused on those with a prevalence in the gene sets of either the over- or under-expressed genes (≥80%, gray marked in the S7–S9 Tables). The analysis showed that *Tcl1-/-* mice are defective to WT in the keratinocyte differentiation, epithelium development and cytoskeleton components, as well as in the mitosis process. Conversely, genes involved in antigen processing, are over-expressed in *Tcl1-/-* vs WT (S7 Table). Interestingly, when *K14-TCL1* mice are compared to WT, the defects in differentiation, development and mitosis are no more found. The over-represented genes are mainly involved in the response to stimuli, actin organization and locomotion. A slight increase in the antigen presentation is still observed, even if the FCs of the annotated genes are all but one lower than 1.15 (S8 Table). Comparison between mutant and transgenic mice strikingly resembled that between *Tcl1-/-* and WT except for the downregulation of mitosis (S9 Table). Through pathway analysis, we identified gene sets that were significantly perturbed (*P*<0.05). Complete lists of altered pathways in WT versus *Tcl1-/-*, WT versus *K14-TCL1* and *Tcl1-/-* versus *K14-TCL1* are given in supplement (S10–S12 Tables, respectively). Focusing on those involved in proliferation, differentiation and survival regulation, most of the altered pathways belong to growth factor signaling (IGF1, EGF, PDGF, NGF), with MAPK/AP1 cascade being also involved (Tables 1, 2 and 3, respectively).

**Table 1:** Genes of S4 Table, with relations between *Tcl1-/-* and WT, are subdivided as pathways.

Biocarta Pathway	Pathway description	Number of genes	LS permutation p-value	KS permutation p-value	Efron-Tibshirani's GSA test p-value
m_insulinPathway	Insulin Signaling Pathway	10	0.00181	0.00356	< 0.005 (-)
m_tollpathway	Toll-Like Receptor Pathway	10	0.00335	0.04491	< 0.005 (-)
m_igf1Pathway	IGF-1 Signaling Pathway	11	0.00414	0.02449	< 0.005 (-)
m_keratinocytePathway	Keratinocyte Differentiation	19	0.00582	0.08422	0.155 (-)
m_pdgfPathway	PDGF Signaling Pathway	13	0.00704	0.05368	< 0.005 (-)
m_ngfPathway	Nerve growth factor pathway (NGF)	10	0.00824	0.00356	< 0.005 (-)
m_egfPathway	EGF Signaling Pathway	13	0.00901	0.15252	< 0.005 (-)
m_tnfr2Pathway	TNFR2 Signaling Pathway	5	0.01526	0.12641	0.105 (-)
m_cdk5Pathway	Phosphorylation of MEK1 by cdk5/p35 down regulates the MAP kinase pathway	5	0.01881	0.07774	0.255 (+)
m_cd40Pathway	CD40L Signaling Pathway	5	0.01954	0.12641	0.105 (-)
m_dspPathway	Regulation of MAP Kinase Pathways Through Dual Specificity Phosphatases	6	0.02606	0.4878	0.295 (+)
m_chemicalPathway	Apoptotic Signaling in Response to DNA Damage	5	0.02607	0.20826	0.21 (-)
m_gleevecpathway	Inhibition of Cellular Proliferation by Gleevec	11	0.03291	0.12153	< 0.005 (-)
m_gpcrPathway	Signaling Pathway from G-Protein Families	8	0.03294	0.35841	0.115 (-)
m_mcmPathway	CDK Regulation of DNA Replication	7	0.07259	0.16498	< 0.005 (+)
m_akap95Pathway	AKAP95 role in mitosis and chromosome dynamics	5	0.10241	0.06738	< 0.005 (+)
m_rasPathway	Ras Signaling Pathway	5	0.19114	0.06091	< 0.005 (+)
m_g1Pathway	Cell Cycle: G1/S Check Point	13	0.23224	0.01245	0.125 (+)
m_RacCycDPathway	Influence of Ras and Rho proteins on G1 to S Transition	7	0.27633	0.02567	0.335 (+)

**Table 2:** Genes of S5 Table, with relations between *K14-TCL1* and WT, are subdivided as pathways.

Biocarta Pathway	Pathway description	Number of genes	LS permutation p-value	KS permutation p-value	Efron-Tibshirani's GSA test p-value
m_akap95Pathway	AKAP95 role in mitosis and chromosome dynamics	5	0.00419	0.02644	< 0.005 (+)
m_tollpathway	Toll-Like Receptor Pathway	10	0.00553	0.07202	< 0.005 (-)
m_chemicalPathway	Apoptotic Signaling in Response to DNA Damage	5	0.00695	0.03123	0.135 (-)
m_egfPathway	EGF Signaling Pathway	13	0.00835	0.04904	< 0.005 (-)
m_cd40Pathway	CD40L Signaling Pathway	5	0.01208	0.16315	0.065 (-)
m_tnfr2Pathway	TNFR2 Signaling Pathway	5	0.01278	0.16315	0.23 (+)
m_pdgfPathway	PDGF Signaling Pathway	13	0.01652	0.16707	< 0.005 (-)
m_dspPathway	Regulation of MAP Kinase Pathways Through Dual Specificity Phosphatases	6	0.01716	0.12992	0.18 (-)
m_gleevecpathway	Inhibition of Cellular Proliferation by Gleevec	11	0.0199	0.16504	< 0.005 (-)
m_cdk5Pathway	Phosphorylation of MEK1 by cdk5/p35 down regulates the MAP kinase pathway	5	0.02752	0.15282	0.455 (-)
m_gpcrPathway	Signaling Pathway from G-Protein Families	8	0.03275	0.21625	< 0.005 (-)
m_g2Pathway	Cell Cycle: G2/M Checkpoint	9	0.03701	0.04128	0.065 (+)
m_ngfPathway	Nerve growth factor pathway (NGF)	10	0.05594	0.25037	< 0.005 (-)
m_atmPathway	ATM Signaling Pathway	10	0.15562	0.02225	0.105 (+)
m_igf1Pathway	IGF-1 Signaling Pathway	11	0.23682	0.48638	< 0.005 (-)
m_fasPathway	FAS signaling pathway (CD95)	8	0.25341	0.27857	< 0.005 (+)
m_tgfbPathway	TGF beta signaling pathway	7	0.43	0.09677	< 0.005 (+)

**Table 3:** Genes of S6 Table, with relations between *Tcl1-/-* and *K14-TCL1* are subdivided as pathways.

Biocarta Pathway	Pathway description	Number of genes	LS permutation p-value	KS permutation p-value	Efron-Tibshirani's GSA test p-value
m_pdgfPathway	PDGF Signaling Pathway	13	0.01738	0.06222	0.24 (-)
m_egfPathway	EGF Signaling Pathway	13	0.01881	0.06222	0.235 (-)
m_insulinPathway	Insulin Signaling Pathway	10	0.04619	0.07895	0.14 (-)
m_igf1Pathway	IGF-1 Signaling Pathway	11	0.05016	0.04466	0.21 (-)
m_wntPathway	WNT Signaling Pathway	8	0.06155	0.0295	0.05 (+)
m_g2Pathway	Cell Cycle: G2/M Checkpoint	9	0.08182	0.01766	0.49 (+)
m_akap95Pathway	AKAP95 role in mitosis and chromosome dynamics	5	0.0887	0.00149	0.47 (+)
m_cd40Pathway	CD40L Signaling Pathway	5	0.11107	0.02473	0.14 (-)
m_aktPathway	AKT Signaling Pathway	6	0.19848	0.00206	0.06 (+)
m_erkPathway	Erk1/Erk2 Mapk Signaling pathway	6	0.29669	0.04846	0.39 (+)

These data strongly confirm, by a molecular point of view, the functional defects observed in the *Tcl1-/-* and rescued in the K14-TCL1 mouse *in vivo* [1] and indicate the growth factor and MAPK signaling among the major molecular deregulations due to *Tcl1a* loss.

### *Tcl1a* loss affects the progenitor cell number and the proliferation rate of murine keratinocytes

**Fig 2:**
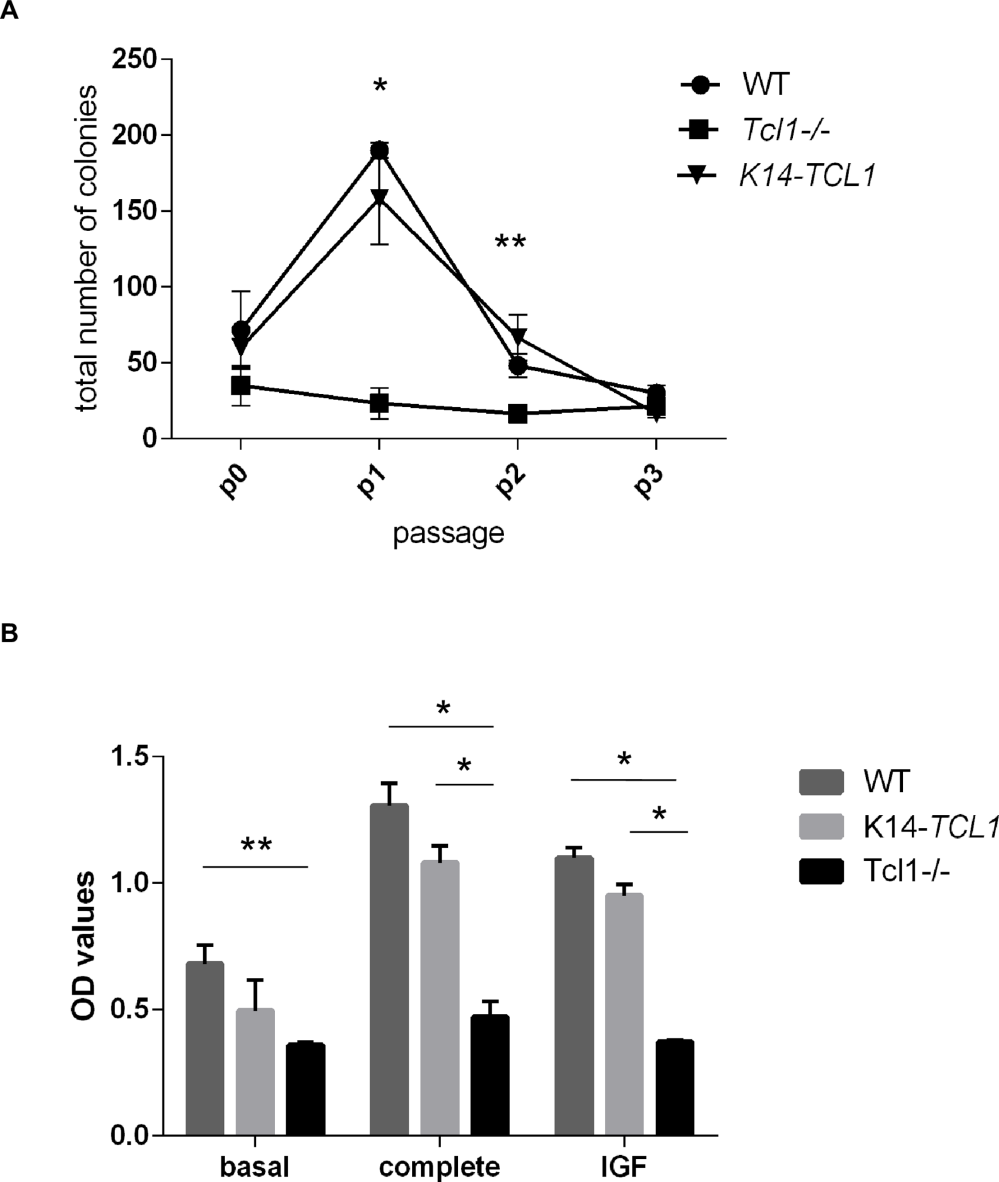
Colony-forming efficiency (CFE) and proliferation are impaired in *Tcl1-/-* mice. (A) CFE assay performed on first three passages KCs of WT (circles), *Tcl1-/-* (squares) and *K14-TCL1; Tcl1-/-* (triangles down) mice; values are expressed as total number of colonies; CFE of WT and *K14-TCL1* are significantly different with respect to *Tcl1-/-* at passages p1 and p2 (*P<0.001; **P<0.005). (B) Proliferation in cultured KCs from WT (dark gray), *Tcl1-/-* (black) or *K14-TCL1* (light gray) mice. KCs at p1 passage were cultured in basal medium, in complete medium, or in basal medium plus 100 ng ml^-1^ IGF-1; OD values are reported. All experiments were performed in triplicates. Significant growth differences between the groups are indicated (*P<0.001; **P<0.005).

Previously, we found a lack of TA proliferating cells and a progressive loss of CD34+ SCs in the hair follicles [1]. Taking advantage of the gene expression data described above, we examined the progenitor cell number in primary KCs isolated from *Tcl1-/-* and from *K14-TCL1* mice compared to WT, through colony-forming efficiency (CFE) analysis (Fig 2A). Soon after isolation (p0), *Tcl1-/-* KCs gave rise to 49%_+/-18_ and 58%_+/-22_ total number of colonies when compared to WT and *K14-TCL1* cells, respectively, although these differences were not significant. The differences became striking at the first passage (p1) as *Tcl1-/-* colonies became 12%_+/-5_ and 15%_+/-7_ of the WT and *K14-TCL1*, respectively. Indeed, *Tcl1-/-* KCs showed no or little variations in their CFE where WT and *K14-TCL1* showed their peak. At p2, the difference in CFE between TCL1-null and TCL1-expressing cells was decreased but still significant (34%_+/-6_ and 25%_+/-4_ of WT and K14-TCL1, respectively). Thereafter, CFE values from the three different types of cells tend to 0 in a similar way (CFE assay has been performed until p10, not shown).

In addition, we analyzed KC growth rate in the three genotypes (Fig 2B). At p1 passage, KCs were cultured in basal or complete medium for 24 hours. In basal medium, cells from *Tcl1-/-* mice grew less than the WT ones (0.53_+/-0.02_-fold change, *P*<0.005), while in *K14-TCL1* cells the defect was less severe (0.73_+/-0.18_-fold change, not significant). When KCs were cultured in complete medium, WT cells about doubled their proliferation rate, as well as *K14-TCL1* ones (1.92_+/-0.13_- and 2.18_+/-0.14_-fold change, respectively), while *Tcl1-/-* cells showed a significant lower increase (1.31_+/-0.17_-fold change) (Fig 2B). Our GEP data showed that the IGF1 pathway is one of the most altered in *Tcl1-/-* mice with respect to WT (Table 1). Since, IGF1 is known to regulate epidermal proliferation [29, 30] we tested its ability to stimulate KCs with respect to basal medium condition. We found a similar proliferation increment in WT and *K14*-*TCL1* cells (1.62_+/-0.06_- and 1.4_+/-0.07_-fold change, respectively) while *Tcl1-/-* cells were completely unresponsive (1.04_+/-0.02_-fold change).

Taken together, these results confirm an important role for TCL1 in maintaining KC progenitor cells and its requirement as a proliferative factor, particularly when acting through the IGF1 pathway.

### The expression of differentiation markers is altered in *Tcl1a* mouse models

**Fig 3:**
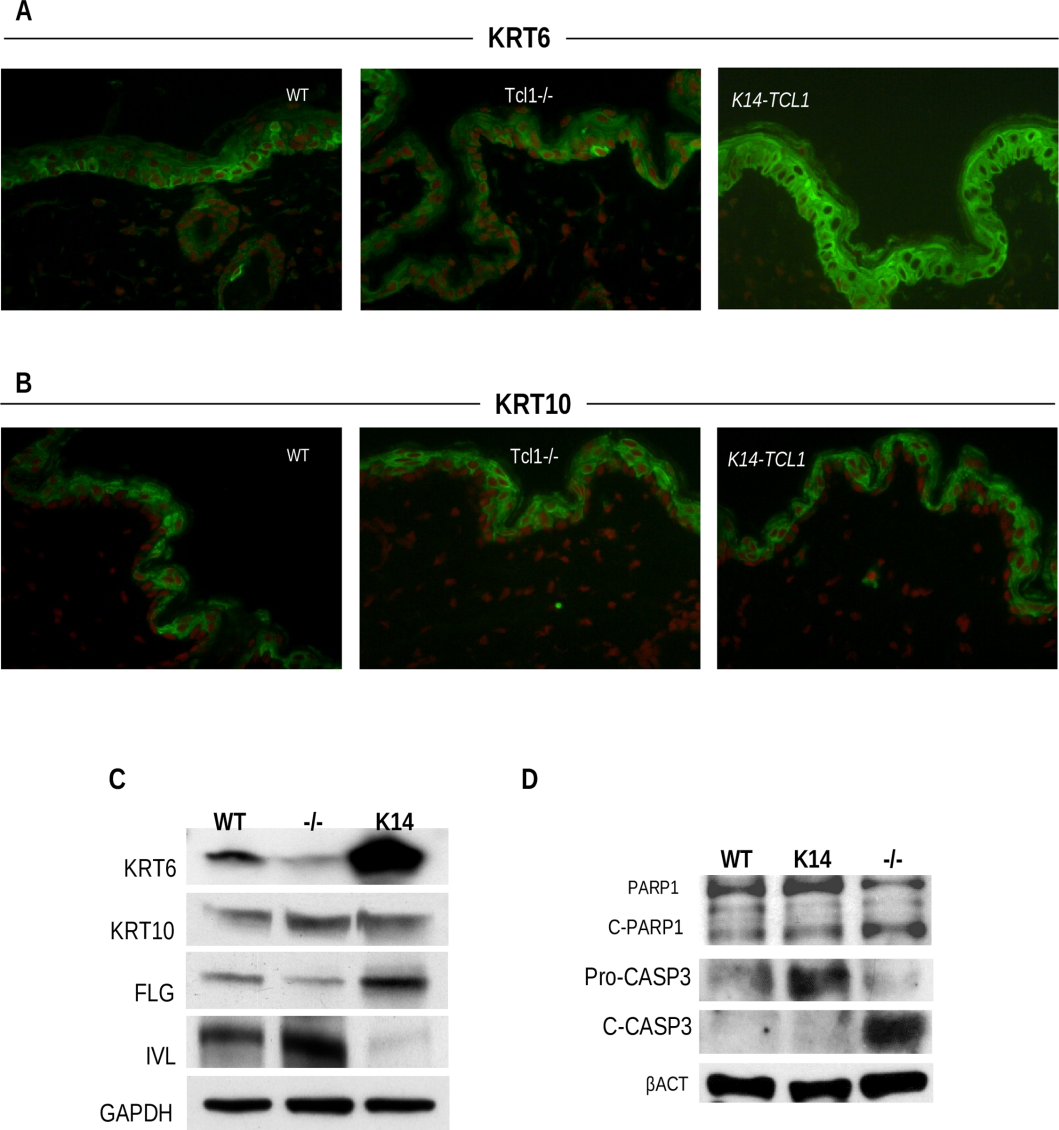
Differentiation and apoptosis are altered in *Tcl1-/-* KCs. Immunofluorescence for the activation marker KRT6 (green) (A) or the differentiation marker KRT10 (green) (B) on epidermis of WT, *Tcl1-/-* and *K14-TCL1* mice. Nuclear counterstain is propidium iodide (red). Magnification: 40X. Images were acquired by confocal laser scanning. Representative images of three experiments are shown. (C) Western blot analysis on protein extracts of epidermal sheet from WT, *K14-TCL1* (K14) and *Tcl1-/-* (-/-) mice for differentiation markers. The results were calculated as fold change of the *K14-TCL1* or *Tcl1-/-* to the WT OD values. Samples were analyzed also for PARP1 and CASP3 apoptosis markers (D). C-PARP1 and C-CASP3 are the cleaved forms. The results were calculated as percentage of the cleaved form to the sum of cleaved and entire normalized OD values.

Our GO analysis showed that modulation of *Tcl1a* expression has consequences in KCs differentiation. Hence, we examined the pattern of expression of differentiation markers that identify upper epidermal layers (spinous and granular) (Fig 3A–3C). Cytokeratin 6 (KRT6) is considered as an activation marker for KCs [31, 32] and is one of the most differentially expressed gene in our GEP analysis (S3–S6 Tables). Accordingly, KRT6 immunofluorescence in 3-day post-depilation (3dpd) skin samples shows localized expression in epidermis surrounding hair follicles in WT mice, while only few scattered cells express KRT6 in *Tcl1-/-* mice. Interestingly, *K14-TCL1* mice show strong and diffuse KRT6 immunoreactivity, in all the epidermis (Fig 3A). WB analysis of epidermal sheet samples 3dpd, confirmed the expression of KRT6 to be dramatically reduced in *Tcl1-/-* mice and highly overexpressed in *K14-TCL1* with respect to WT mice (0.36- and 8-fold change, respectively, Fig 3C). Cytokeratin 10 (KRT10) is one of the first differentiation marker to be expressed by post-mitotic cells in spinous layer and it has a role in proliferation control [33]. Immunofluorescence confirmed supra-basal expression of KRT10 in all three genotypes (Fig 3B). In this case, WB analysis showed both *Tcl1-/-* and *K14-TCL1* mice to have more than 2-fold change in KRT10 expression with respect to WT mice (2.8- and 2.2-fold, respectively) (Fig 3C). FLG and IVL are terminal differentiation markers of granular layer, but are regulated in an opposite way in *Tcl1a* mouse models. WB experiments showed that FLG is more expressed in *K14-TCL1* mice (3-fold) and less in *Tcl1-/-* mice (0.4-fold), when compared to WT; conversely, IVL is augmented in *Tcl1-/-* KCs (4-fold), while it is almost undetectable in *K14-TCL1* ones (0.12-fold) (Fig 3C). Overall, these data demonstrate that TCL1 is profoundly involved in the regulation of both early and terminal KC differentiation programs.

Finally, we previously observed that basal KCs in the epidermis of *Tcl1-/-* mice aberrantly co-express proliferation and apoptosis markers *in vivo* [1]. Moreover, there are evidences for TCL1 anti-apoptotic effect through the decrease of PARP1 cleavage in humans [25]. Consistently with this function, by WB experiments in 3dpd epidermis, we found that the percentage of PARP1 cleavage in *Tcl1-/-* is higher than in WT (63% and 35%, respectively), while in *K14-TCL1* it is comparable to WT (38%) (Fig 3D). Similar results were obtained analyzing Caspase 3 cleavage (69%, 33% and 37%, respectively). Hence, these experiments confirm the anti-apoptotic role of TCL1 in murine KCs.

### TCL1 modulation affects the phosphorylation levels of ERK1/2, SAPK/JNK and P38 MAPK

Unlike the TCL1 function in the immune system [22], when we analyzed AKT activation in mutant and transgenic KCs by kinase assay, we didn’t find significant differences with respect to WT (Panel A in S1 Fig). Consistently with this result, phosphorylated AKT(Ser473) does not co-localize with TCL1 in WT follicular keratinocytes, analyzed by immunofluorescence (Panel B in S1 Fig). Moreover, pathway analysis have not pointed out the AKT signaling among those affected by TCL1 modulation. Rather, our GEP data have highlighted the involvement of MAPKs in response to *Tcl1a* modulation. The MAPK cascade has a central role on the regulation of differentiation and programmed cell death in the epidermis, through the phosphorylation of ERK1/2, SAPK/JNK and P38 [34].

**Fig 4:**
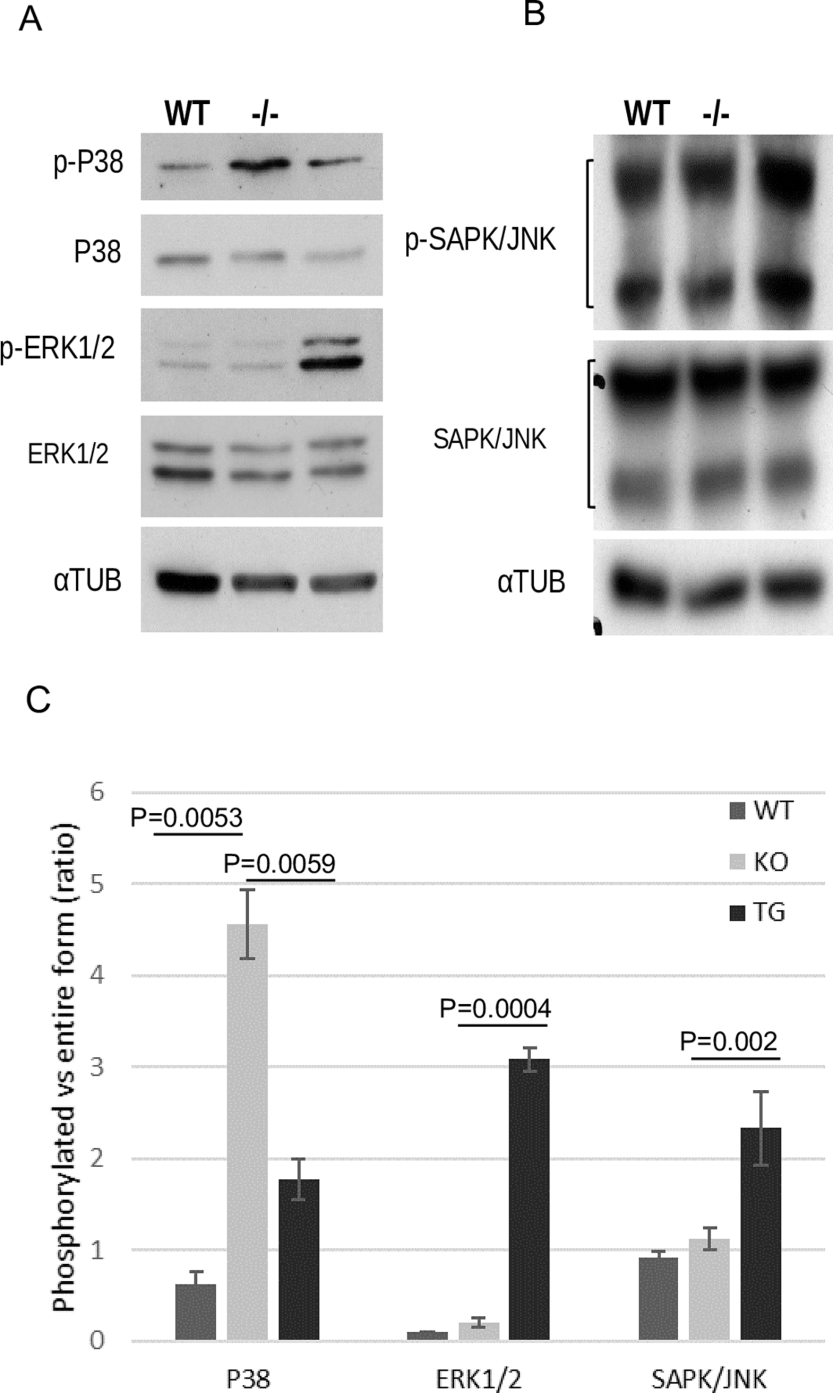
MAPKs phosphorylation pattern is modified by TCL1 loss- or gain-of function. Western blot analysis of phosphorylated forms of P38, ERK1/2 (A) and SAPK/JNK MAPKs (B) on protein extracts from keratinocytes of WT, *Tcl1-/-* and *K14-TCL1* mice. Tubulin alpha (αTUB) was used to normalize protein load. (C) Normalized OD values were used to calculate the ratio of phosphorylated to entire forms and graphed as the mean and standard deviation of three independent experiments for the WT (dark gray), *Tcl1-/-* (light gray) and *K14-TCL1* (black) KCs.

Thus, we investigated by WB analysis the phosphorylation rate of ERK1/2, SAPK/JNK and P38 MAPKs in KCs from *Tcl1-/-* and *K14-TCL1* mice, compared to the WT samples (Fig 4). We found that *Tcl1a* loss increased the P38 phosphorylation rate when compared to WT and *K14-TCL1* mice by 7.2- and 2.8-fold, respectively (Fig 4A and 4C). On the opposite, the over-expression of *TCL1A* induced a 32-fold increased ERK1/2 (Fig 4A–4C) and a 2.6-fold increased SAPK/JNK (Fig 4B and 4C) phosphorylation with respect to WT. Normalized optical density values (OD) of the phosphorylated and entire forms are given in supplement (Panels A, B and C in S2 Fig). The graph in Fig 4C shows the phosphorylation rate for these proteins calculated as the OD ratio of phosphorylated to entire form. These results indicate that in the absence of *Tcl1a*, P38 route is hyper-activated, leaving unaffected ERK1/2 and SAPK/JNK signaling. On the other hand, over-expression of *TCL1A* in the basal KCs partially restores the phosphorylation of P38 to WT levels and enhances the activation of ERK1/2 and SAPK/JNK.

### TCL1 inhibits cFOS expression and its nuclear localization and induces cJUN phosphorylation

The activator protein 1 (AP1) transcriptional complex represents one of the main effector of the MAPK signaling cascade in the epidermis [34, 35]. Since our GEP data show 8.4-fold increased *cFos* mRNA in *Tcl1-/-* mice with respect to WT (S4 Table), we investigated changes in the main AP1 subunits cFOS and cJUN.

**Fig 5:**
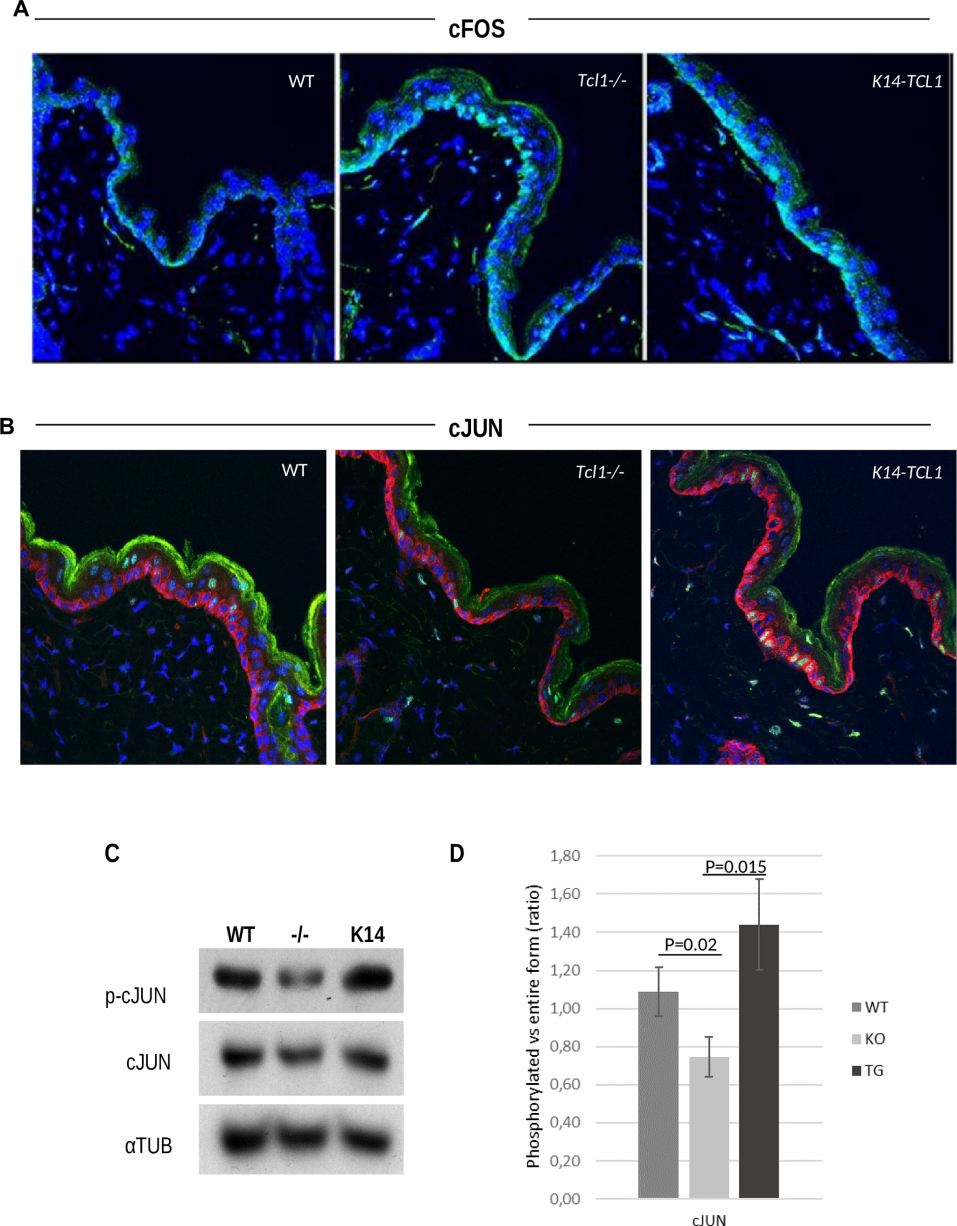
cFos and cJUN AP1 components are affected by TCL1 loss- or gain-of function through different mechanisms. (A) Immunofluorescence of cFOS (green), or (B) cJUN (red) and phospho-cJUN (green) on WT, *Tcl1-/-* and *K14-TCL1* mice epidermis. Nuclear counterstain is propidium iodide (blue).Merging of green and blue fluorescence represents nuclear localization (light blue). Magnification: 40X. Images were acquired by confocal laser scanning. (C) Western blot analysis of cJUN and phospho-cJUN on protein extracts of KCs from WT, *Tcl1-/-* and *K14-TCL1* mice. (D) Normalized OD values were used to calculate the ratio of phosphorylated to entire form and graphed as the mean and standard deviation of three independent experiments for the WT (dark gray), *Tcl1-/-* (light gray) and *K14-TCL1* (black) mice.

We analyzed the cFOS protein by confocal analysis on 3dpd skin samples from the three genotypes (Fig 5A). The first evidence is that, accordingly to mRNA data, cFOS expression is enhanced in *Tcl1-/-* when compared to WT, also at the protein level and it is only partially restored in *K14-TCL1* mice. Interestingly, we also found that, in the WT skin, cFOS cellular localization is totally restricted to the cytoplasmic compartment of the basal layer. In the WT panel of Fig 5A the green fluorescence, representing cFOS staining, is all around the blue fluorescence of the nuclei. Conversely, in the absence of *Tcl1a*, cFOS is strongly localized to the nuclear compartment of the cell visualized as light blue fluorescence in Fig 5A. In the *K14-TCL1* panel, the cFOS staining pattern is intermediate between WT and Tcl1-/- samples. These data suggest the TCL1-mediated inhibition of cFOS transcriptional activity occurring in human B-CLL [25], might be functional in the murine KCs as well. When we analyzed cJUN expression in the skin of the three mouse models, we observed comparable levels of cJUN total protein, which was restricted to the cytoplasm of basal KCs in all samples (Fig 5B). On the contrary, cJUN phosphorylation was associated with nuclear localization and the number of positive cells was found decreased in *Tcl1-/-* and increased in *K14-TCL1* KCs, when compared to the WT counterpart. To confirm and quantify this difference, we analyzed cJUN phosphorylation rate by WB and we found a 0.7-fold decreased cJUN phosphorylation in *Tcl1-/-* KCs, when compared to the WT (Fig 5C and 5D). At the same time, over-expression of *TCL1A* leads to a 1.9- and 1.3-fold increased cJUN phosphorylation rate when compared to *Tcl1-/-* and WT, respectively. Normalized OD values of the phosphorylated and entire forms are given in supplement (Panel D in S2 Fig). These experiments show that TCL1 takes part in AP1 regulation, by controlling both cFOS localization and cJUN phosphorylation.

## Discussion

Previously we showed that adult *Tcl1-/-* mice developed skin defects due to dramatic reduction of CD34 stem cell marker and proliferating TA cells in the hair follicle, as well as dysregulated proliferation/differentiation/apoptosis program in the interfollicular epidermis [1]. In this work, we finally demonstrated that *Tcl1a* is required for the correct programming and commitment of the epidermal KCs.

The GEP analysis shows that *Tcl1a* loss provokes profound gene expression deregulation in the epidermal tissue. Furthermore, the mouse model carrying the *K14*-driven *TCL1A* gene in a *Tcl1a* null background is more similar to the WT than to the *Tcl1-/-* one, therefore giving a first general statistical confirmation of the previously observed ability of the *K14*-*TCL1* transgenic mouse to rescue the *Tcl1-/-* mutant phenotype. In particular, the expression of 55% of genes deregulated in the *Tcl1-/-* are restored in *K14*-*TCL1* KCs. Among these, 36 genes (39%) are completely rescued at physiologic level or overturned, suggesting a close relationship with TCL1. Interestingly, 29 out of these 36 genes were downregulated in the *Tcl1-/-* KCs, confirming the role for TCL1 as a transcription activator [25] also in epidermal cells. On the other hand, the *K14-TCL1* mouse is not able to rescue all the genes affected by the absence of *Tcl1a*, especially those over-expressed, suggesting that the presence of *Tcl1a* is necessary, but not sufficient, for these factors. Another possibility is that the *K14*-driven *TCL1A* is unable to regulate factors acting in supra-basal layers due to its expression limited to basal layer.

At the functional level, the GO analysis pointed out the differentiation and cell division as two functions defective in *Tcl1-/-* KCs, which are rescued in the *K14-TCL1*. In contrast with the T and B cell *TCL1A* transgenic models, overexpression in epidermal KCs did not induce hyper-proliferation, nor did we observe skin tumours formation in the *K14-TCL1* mice [1]. This phenotype is in agreement with our GEP data and GO analysis and might be due to a milder activity of the *K14* promoter with respect to the *lck* or *Eμ* ones. Alternatively, the proliferation/survival programs in the KCs might be subjected to a tight control, able to overcome the TCL1A overexpression. Rather, the over-represented genes belong to the response to stimulus and cell motility GO terms, with no evidence for any aberrant phenotype.

*Krt6* is one of the most significant differentially expressed gene found by our GEP analysis. *Krt6* is not constitutively expressed in the skin, but is rapidly induced after injury of the epidermis (e.g. waxing) and is considered a marker of KCs activation both for proliferation and for differentiation [31, 32]. Interestingly, we observed that in 3dpd WT epidermis, KRT6 protein is mainly expressed by basal cells and maintained in some supra-basal cells; in *Tcl1-/-* epidermis, KRT6 expression is dramatically reduced and in *K14-TCL1*, KRT6 is highly expressed in all the layers. Thus, KRT6 expression is strongly dependent from TCL1 regulation, is induced in the basal layer and maintained in supra-basal layers, indicating that KRT6 might be a good candidate, at least in part, for the observed differentiative and/or proliferative phenotypes. Conversely, the supra-basal marker KRT10 is increased in *Tcl1-/-* as well as in *K14-TCL1* mouse, when compared to WT, suggesting to exemplify the above mentioned ‘not rescued’ over-expressed genes. Yet, the expression of the terminal differentiation markers FLG and IVL are altered in *Tcl1-/-* but rescued in *K14-TCL1* mouse, suggesting that in supra-basal KCs, some differentiation programs might be activated from the basal layer. Apoptosis signaling represents another interesting example of the basal/supra-basal stratification affected by *Tcl1a* loss. In our previous work, we observed proliferative marker coexisting with apoptotic events in basal layer of *Tcl1-/-* mouse, although programmed cell death is normally activated in terminally differentiated layer only. This aberration was rescued by K14-driven *TCL1A* over-expression [1]. Here, we showed that the apoptotic factors PARP1 and CASP3 are abnormally activated in *Tcl1-/-* KCs with respect to WT and the K14-*TCL1* expression is sufficient to restore physiologic activation levels, resembling the TCL1 anti-apoptotic effect observed in human CLL [25].

Proliferation is another defective function we described in the *Tcl1-/-* skin [1], which has been pointed out also in this work by the GO analysis. Accordingly, primary KCs from *Tcl1-/-* mice are almost unable to form colonies or to proliferate when placed in complete media. On the other hand, this phenotype is completely rescued when *TCL1A* is reinserted in basal cells of K14-*TCL1* mouse, mimicking the WT behavior. These experiments confirmed the TCL1A overexpression to induce no hyper-proliferation in KCs. Pathway analysis identified growth factor and MAPK as some of the most significantly pathways altered in the Tcl1-/- KCs. Since IGF1 is known to regulate epidermal proliferation, motility, and progenitor cell behaviour, acting through MAPK-AP1 and/or PI3K-AKT pathways [29, 30, 36, 37], we focused on IGF1-induced proliferation. Interestingly, we found a proliferation pattern closely resembling that obtained with serum stimulation, with impaired proliferation of *Tcl1-/-* KCs, which was significantly rescued by K14-TCL1 expression. This finding demonstrates that *Tcl1a* gene is definitely necessary for KCs proliferation both by serum- or IGF1-induction.

Actually, despite the most known TCL1 function is to enhance AKT kinase activity [21, 23], we did not found any difference in the AKT kinase activity between the three mouse models. This finding is consistent with the two-cell embryo model, which showed no differences between the phosphorylated AKT content of *Tcl1-/-* and WT mice [20]. Noguchi et al. had proposed that the AKT kinase activity maintained in the *Tcl1a* null mice might be due in part to the presence of five TCL1B family proteins and MTCP1 in the mouse genome [38]. Accordingly, we had reported the high level expression of the *Tcl1B2* isoform in the mouse epidermis, especially in the Tcl1-/- one [1].

As for IGF1, most of the incoming signals that regulate proliferation, differentiation, and apoptosis in the interfollicular epidermis are shuttled through the three major MAPK cascades ERK1/2, SAPK/JNK, and P38 with several interconnections among the cascades [39]. ERK1/2 activation primarily mediates proliferative and survival signals from a range of different surface molecules, including growth factors and cellular adhesion proteins; on the other hand, P38 MAPK-dependent mechanisms are mainly involved in regulating differentiation and apoptosis processes [34, 40–43]. SAPK/JNK is able to regulate proliferation, differentiation, survival and/or migration, thus tuning a variety of processes such as tissue homeostasis, cell metabolism, inflammation and carcinogenesis [44]. The relative activities of MAPKs can be evaluated by their phosphorylation rate and here we provide evidences that *Tcl1-/-* mouse shows a hyper-activation of P38 MAPK, which might be responsible for the apoptotic phenotype observed. Additionally, P38 hyper-phosphorylation might account for differentiative disorders such as IVL overexpression [45]. On the converse, the overexpression of *TCL1A* by *K14*-driven transgene increases ERK1/2 and SAPK/JNK phosphorylation. That TCL1 may influence ERK1/2 phosphorylation was observed also in B-CLL, where the TCL1-targeting microRNA-181b decreased phospho-ERK1/2 as well [46]. Thus, TCL1 could act as a proliferative/pro-survival factor and a differentiation regulator by increasing the phosphorylation on ERK1/2 and SAPK/JNK and maintaining low levels of phospho-P38.

In epidermal KCs, downstream targets of MAPK signalling are primarily represented by AP1 transcription factor, which is composed of homo- or hetero-dimers mainly belonging to the FOS and JUN protein families. MAPKs can both affect AP1 activity, by direct phosphorylation, and by transcriptional activation of the promoters of specific AP1 components. Multiple AP1 family members are expressed in the epidermis and form different dimer pairs, regulating expression of several target genes and controlling KC functions [45, 47, 48]. It is not by haphazard that by chip analysis we have found, among the most dysregulated genes, several factors participating to the MAPK signaling and, in particular, the AP1 effector *cFos*. Nevertheless, TCL1 has been reported to bind cFOS directly, thus inhibiting apoptosis [25, 49]. Our data are consistent with this observation and we can hypothesize that TCL1 prevents cFOS transcriptional function by sequestering this factor into the cytoplasm, having cFOS mainly nuclear localization in the *Tcl1-/-* epidermal KCs. This phenotype is rescued, although not entirely, in the K14-*TCL1* mice. Moreover, we have shown that phosphorylation of cJUN is reduced in the absence of *Tcl1a* and increased once *TCL1A* has been reintroduced into the KCs. Hence, in mouse KCs TCL1 seems to have a stimulating rather than an inhibitory effect on cJUN activity that was seen in B-CLL by Pekarsky et al. [25]. This would be consistent with a role for cJUN in proliferation, differentiation and wound healing, in mouse epidermal cells [50–52].

## Conclusions

In conclusion, analyzing the effects of altered *Tcl1a* gene expression in knockout or transgenic mouse models, we have demonstrated, for the first time, that TCL1 is a major factor on epidermal KCs' proliferation, in particular when serum- or IGF1-stimulated. Additionally, *Tcl1a* loss is associated with differentiation disorders and uncontrolled apoptosis. Conversely, overexpression of TCL1A is able to rescue almost completely the mutant defects and do not show any evident aberration. Our data suggest that TCL1 function in KCs is exerted, at least in part, by regulating the MAPK phosphorylation balance between ERK1/2, SAPK/JNK and p38, the phosphorylation rate of cJUN and the cellular localization of cFOS, that means multilevel control of the activity of the MAPK/AP1 axis. Whether the role of TCL1 is due to direct interactions with the MAPK factors above mentioned or with the AP1 subunits, cJUN and cFOS, will be the object of further investigations. Finally, we intend to analyze TCL1 role also in the human hair follicle and epidermis from skin tissues of both healthy donors and patients affected by skin diseases.

## Supporting information

S1 Table Real Time PCR primers and FC. The sequence of forward and reverse primers is reported for the target and housekeeping genes. FC values were calculated as described in M&M section and compared to those obtained by GEP analysis.
(XLSX)

S2 Table List of antibodies used for WB experiments. The company, catalog number and working dilution for every antibody is reported.
(XLSX)

S3 Table Differentially expressed genes between WT, *Tcl1-/-* and *K14-TCL1* KCs. Class 1: *Tcl1-/-*; Class 2: *K14-TCL1*; Class 3: WT. The first 271 genes are significant at the nominal 0.001 level of the univariate test The 'Pairwise significant' column shows pairs of classes with significantly different gene expression at alpha = 0.01. Class labels in a pair are ordered (ascending) by their averaged gene expression. In the last column the “rescued” (light gray), “not rescued” (gray) and “not included” (NI, dark gray) groups are reported.
(XLSX)

S4 Table Differentially expressed genes in *Tcl1-/-* vs WT KCs. Class 1: *Tcl1-/-*; Class 2: WT. The first 254 genes are significant at the nominal 0.001 level of the univariate test.
(XLSX)

S5 Table Differentially expressed genes in *K14-TCL1* vs WT KCs. Class 1: *K14-TCL1*; Class 2: WT. The first 223 genes are significant at the nominal 0.001 level of the univariate test.
(XLSX)

S6 Table Differentially expressed genes in Tcl1-/- vs *K14-TCL1* KCs. Class 1: *Tcl1-/-*; Class 2: *K14-TCL1*. The first 218 genes are significant at the nominal 0.001 level of the univariate test.
(XLSX)

S7 Table GO analysis in Tcl1-/- vs WT KCs. 120 out of 442 investigated gene sets passed the 0.005 significance threshold. LS/KS permutation test found 4 significant gene sets. Efron-Tibshirani's maxmean test found 120 significant gene sets (under 200 permutations). Gene sets with at least 80% of either under-expressed or over-expressed genes are highlighted (gray). Class 1: *Tcl1-/-*; Class 2: WT.
(XLSX)

S8 Table GO analysis in *K14-TCL1* vs WT KCs. 69 out of 442 investigated gene sets passed the 0.005 significance threshold. LS/KS permutation test found 2 significant gene sets. Efron-Tibshirani's maxmean test found 67 significant gene sets (under 200 permutations). Gene sets with at least 80% of either under-expressed or over-expressed genes are highlighted (gray). Class 1: *K14-TCL1*; Class 2: WT.
(XLSX)

S9 Table GO analysis in Tcl1-/- vs WT KCs. 64 out of 442 investigated gene sets passed the 0.005 significance threshold. LS/KS permutation test found 15 significant gene sets. Efron-Tibshirani's maxmean test found 62 significant gene sets (under 200 permutations). Gene sets with at least 80% of either under-expressed or over-expressed genes are highlighted (gray). Class 1: *Tcl1-/-*; Class 2: *K14-TCL1*.
(XLSX)

S10 Table Pathway analysis in Tcl1-/- vs WT KCs. 49 out of 151 investigated gene sets passed the 0.05 significance threshold. LS/KS permutation test found 40 significant gene sets. Efron-Tibshirani's maxmean test found 33 significant gene sets (under 200 permutations). Class 1: *Tcl1-/-*; Class 2: WT.
(DOCX)

S11 Table Pathway analysis in *K14-TCL1* vs WT KCs. 44 out of 151 investigated gene sets passed the 0.05 significance threshold. LS/KS permutation test found 34 significant gene sets. Efron-Tibshirani's maxmean test found 22 significant gene sets (under 200 permutations). Class 1: *K14-TCL1*; Class 2: WT.
(DOCX)

S12 Table Pathway analysis in Tcl1-/- vs WT KCs. 23 out of 151 investigated gene sets passed the 0.05 significance threshold. LS/KS permutation test found 20 significant gene sets. Efron-Tibshirani's maxmean test found 7 significant gene sets (under 200 permutations). Class 1: *Tcl1-/-*; Class 2: *K14-TCL1*.
(DOCX)

S1 Fig TCL1 loss- or gain-of-function do not influence AKT activity. (A) Kinase assay was performed on keratinocyte lysates, according to manufacturer’s protocol. Graph represents mean OD values and standard error bars of three independent experiments. No differences were observed between the three genotypes. (B) Immunofluorescence for mouse TCL1 protein (red) and phosphorylated AKT(Ser473) (green). Nuclear counterstain is propidium iodide (PI, blue). Merging of red and blue fluorescence represents TCL1 nuclear localization (pink). Magnification: 40X. Images were acquired by confocal laser scanning.
(TIF)

S2 Fig Western blot analysis of the MAPKs and cJUN phosphorylation pattern. WB analysis of phosphorylated (dark gray) and entire (light gray) forms of P38 (A), ERK1/2 (B) SAPK/JNK MAPKs (C) and cJUN (D) on protein extracts from keratinocytes of WT, *Tcl1-/-* and *K14-TCL1* mice. Tubulin alpha (αTUB) was used to normalize protein load. Normalized OD values are graphed as the mean and standard deviation of three independent experiments.
(TIF)
