# Supplementary material for: T Cell Leukemia/Lymphoma 1A is essential for mouse epidermal keratinocytes proliferation promoted by insulin-like growth factor 1
Source: PLoS One. 2018 Oct 4;13(10):e0204775. doi: 10.1371/journal.pone.0204775 (PMC6171881; doi:10.1371/journal.pone.0204775)
Supplement: S10 Table — 49 out of 151 investigated gene sets passed the 0.05 significance threshold. LS/KS permutation test found 40 significant gene sets. Efron-Tibshirani's maxmean test found 33 significant gene sets (under 200 permutations). Class 1: Tcl1-/-; Class 2: WT. (DOCX) [file pone.0204775.s010.docx]

|  | **Biocarta Pathway** | **Pathway description** | **Gene symbol** | **Description** | **Parametric p-value** | **Geom mean of intensities in class 1** | **Geom mean of intensities in class 2** | **Fold-change** |
| --- | --- | --- | --- | --- | --- | --- | --- | --- |
| 1 | m_cdMacPathway | [Cadmium induces DNA synthesis and proliferation in macrophages](http://cgap.nci.nih.gov/Pathways/BioCarta/m_cdMacPathway) | [Fos](http://www.ncbi.nlm.nih.gov/entrez/query.fcgi?cmd=search&db=gene&term=Fos) | FBJ osteosarcoma oncogene | < 1e-07 | 2482.37 | 294.09 | 8.44 |
| 2 |  | [Cadmium induces DNA synthesis and proliferation in macrophages](http://cgap.nci.nih.gov/Pathways/BioCarta/m_cdMacPathway) | [Prkcb](http://www.ncbi.nlm.nih.gov/entrez/query.fcgi?cmd=search&db=gene&term=Prkcb) | protein kinase C, beta | 1.3e-06 | 231.8 | 135.08 | 1.72 |
| 3 |  | [Cadmium induces DNA synthesis and proliferation in macrophages](http://cgap.nci.nih.gov/Pathways/BioCarta/m_cdMacPathway) | [Nfkbia](http://www.ncbi.nlm.nih.gov/entrez/query.fcgi?cmd=search&db=gene&term=Nfkbia) | nuclear factor of kappa light polypeptide gene enhancer in B-cells inhibitor, alpha | 4.2e-06 | 1283.3 | 852.99 | 1.5 |
| 4 |  | [Cadmium induces DNA synthesis and proliferation in macrophages](http://cgap.nci.nih.gov/Pathways/BioCarta/m_cdMacPathway) | [Jun](http://www.ncbi.nlm.nih.gov/entrez/query.fcgi?cmd=search&db=gene&term=Jun) | Jun oncogene | 7.6e-06 | 325.38 | 205.78 | 1.58 |
| 5 |  | [Cadmium induces DNA synthesis and proliferation in macrophages](http://cgap.nci.nih.gov/Pathways/BioCarta/m_cdMacPathway) | [Nfkb1](http://www.ncbi.nlm.nih.gov/entrez/query.fcgi?cmd=search&db=gene&term=Nfkb1) | nuclear factor of kappa light polypeptide gene enhancer in B-cells 1, p105 | 0.0055039 | 708.56 | 804.29 | 0.88 |
| 6 |  | [Cadmium induces DNA synthesis and proliferation in macrophages](http://cgap.nci.nih.gov/Pathways/BioCarta/m_cdMacPathway) | [Mapk1](http://www.ncbi.nlm.nih.gov/entrez/query.fcgi?cmd=search&db=gene&term=Mapk1) | mitogen-activated protein kinase 1 | 0.0854614 | 196.98 | 247.01 | 0.8 |
| 7 |  | [Cadmium induces DNA synthesis and proliferation in macrophages](http://cgap.nci.nih.gov/Pathways/BioCarta/m_cdMacPathway) | [Tnf](http://www.ncbi.nlm.nih.gov/entrez/query.fcgi?cmd=search&db=gene&term=Tnf) | tumor necrosis factor | 0.3010041 | 58.77 | 63.31 | 0.93 |
| 1 | m_insulinPathway | [Insulin Signaling Pathway](http://cgap.nci.nih.gov/Pathways/BioCarta/m_insulinPathway) | [Fos](http://www.ncbi.nlm.nih.gov/entrez/query.fcgi?cmd=search&db=gene&term=Fos) | FBJ osteosarcoma oncogene | < 1e-07 | 2482.37 | 294.09 | 8.44 |
| 2 |  | [Insulin Signaling Pathway](http://cgap.nci.nih.gov/Pathways/BioCarta/m_insulinPathway) | [Irs1](http://www.ncbi.nlm.nih.gov/entrez/query.fcgi?cmd=search&db=gene&term=Irs1) | insulin receptor substrate 1 | 1.2e-06 | 652.96 | 397.14 | 1.64 |
| 3 |  | [Insulin Signaling Pathway](http://cgap.nci.nih.gov/Pathways/BioCarta/m_insulinPathway) | [Jun](http://www.ncbi.nlm.nih.gov/entrez/query.fcgi?cmd=search&db=gene&term=Jun) | Jun oncogene | 7.6e-06 | 325.38 | 205.78 | 1.58 |
| 4 |  | [Insulin Signaling Pathway](http://cgap.nci.nih.gov/Pathways/BioCarta/m_insulinPathway) | [Ptpn11](http://www.ncbi.nlm.nih.gov/entrez/query.fcgi?cmd=search&db=gene&term=Ptpn11) | protein tyrosine phosphatase, non-receptor type 11 | 0.0002035 | 49.82 | 67.58 | 0.74 |
| 5 |  | [Insulin Signaling Pathway](http://cgap.nci.nih.gov/Pathways/BioCarta/m_insulinPathway) | [Pik3r1](http://www.ncbi.nlm.nih.gov/entrez/query.fcgi?cmd=search&db=gene&term=Pik3r1) | phosphatidylinositol 3-kinase, regulatory subunit, polypeptide 1 (p85 alpha) | 0.0011172 | 134.94 | 110.05 | 1.23 |
| 6 |  | [Insulin Signaling Pathway](http://cgap.nci.nih.gov/Pathways/BioCarta/m_insulinPathway) | [Csnk2a2](http://www.ncbi.nlm.nih.gov/entrez/query.fcgi?cmd=search&db=gene&term=Csnk2a2) | casein kinase 2, alpha prime polypeptide | 0.0020382 | 2035.41 | 1719.9 | 1.18 |
| 7 |  | [Insulin Signaling Pathway](http://cgap.nci.nih.gov/Pathways/BioCarta/m_insulinPathway) | [Csnk2a1](http://www.ncbi.nlm.nih.gov/entrez/query.fcgi?cmd=search&db=gene&term=Csnk2a1) | casein kinase 2, alpha 1 polypeptide | 0.0029286 | 200.24 | 272.9 | 0.73 |
| 8 |  | [Insulin Signaling Pathway](http://cgap.nci.nih.gov/Pathways/BioCarta/m_insulinPathway) | [Mapk8](http://www.ncbi.nlm.nih.gov/entrez/query.fcgi?cmd=search&db=gene&term=Mapk8) | mitogen-activated protein kinase 8 | 0.0235064 | 29.15 | 25.43 | 1.15 |
| 9 |  | [Insulin Signaling Pathway](http://cgap.nci.nih.gov/Pathways/BioCarta/m_insulinPathway) | [Pik3cg](http://www.ncbi.nlm.nih.gov/entrez/query.fcgi?cmd=search&db=gene&term=Pik3cg) | phosphoinositide-3-kinase, catalytic, gamma polypeptide | 0.0302332 | 18.24 | 14.76 | 1.24 |
| 10 |  | [Insulin Signaling Pathway](http://cgap.nci.nih.gov/Pathways/BioCarta/m_insulinPathway) | [Sos1](http://www.ncbi.nlm.nih.gov/entrez/query.fcgi?cmd=search&db=gene&term=Sos1) | son of sevenless homolog 1 (Drosophila) | 0.0657446 | 82.16 | 76.11 | 1.08 |
| 1 | m_nkcellsPathway | [Ras-Independent pathway in NK cell-mediated cytotoxicity](http://cgap.nci.nih.gov/Pathways/BioCarta/m_nkcellsPathway) | [H2-D1](http://www.ncbi.nlm.nih.gov/entrez/query.fcgi?cmd=search&db=gene&term=H2-D1) | histocompatibility 2, D region locus 1 | < 1e-07 | 2751.11 | 57.77 | 47.62 |
| 2 |  | [Ras-Independent pathway in NK cell-mediated cytotoxicity](http://cgap.nci.nih.gov/Pathways/BioCarta/m_nkcellsPathway) | [H2-K1](http://www.ncbi.nlm.nih.gov/entrez/query.fcgi?cmd=search&db=gene&term=H2-K1) | histocompatibility 2, K1, K region | < 1e-07 | 496.79 | 78.88 | 6.3 |
| 3 |  | [Ras-Independent pathway in NK cell-mediated cytotoxicity](http://cgap.nci.nih.gov/Pathways/BioCarta/m_nkcellsPathway) | [Pik3r1](http://www.ncbi.nlm.nih.gov/entrez/query.fcgi?cmd=search&db=gene&term=Pik3r1) | phosphatidylinositol 3-kinase, regulatory subunit, polypeptide 1 (p85 alpha) | 0.0011172 | 134.94 | 110.05 | 1.23 |
| 4 |  | [Ras-Independent pathway in NK cell-mediated cytotoxicity](http://cgap.nci.nih.gov/Pathways/BioCarta/m_nkcellsPathway) | [Klrc1](http://www.ncbi.nlm.nih.gov/entrez/query.fcgi?cmd=search&db=gene&term=Klrc1) | killer cell lectin-like receptor subfamily C, member 1 | 0.0042434 | 9.86 | 14.42 | 0.68 |
| 5 |  | [Ras-Independent pathway in NK cell-mediated cytotoxicity](http://cgap.nci.nih.gov/Pathways/BioCarta/m_nkcellsPathway) | [Vav1](http://www.ncbi.nlm.nih.gov/entrez/query.fcgi?cmd=search&db=gene&term=Vav1) | vav 1 oncogene | 0.005748 | 23.69 | 19.16 | 1.24 |
| 6 |  | [Ras-Independent pathway in NK cell-mediated cytotoxicity](http://cgap.nci.nih.gov/Pathways/BioCarta/m_nkcellsPathway) | [Klrd1](http://www.ncbi.nlm.nih.gov/entrez/query.fcgi?cmd=search&db=gene&term=Klrd1) | killer cell lectin-like receptor, subfamily D, member 1 | 0.1831396 | 86.87 | 80.86 | 1.07 |
| 1 | m_arenrf2Pathway | [Oxidative Stress Induced Gene Expression Via Nrf2](http://cgap.nci.nih.gov/Pathways/BioCarta/m_arenrf2Pathway) | [Fos](http://www.ncbi.nlm.nih.gov/entrez/query.fcgi?cmd=search&db=gene&term=Fos) | FBJ osteosarcoma oncogene | < 1e-07 | 2482.37 | 294.09 | 8.44 |
| 2 |  | [Oxidative Stress Induced Gene Expression Via Nrf2](http://cgap.nci.nih.gov/Pathways/BioCarta/m_arenrf2Pathway) | [Prkcb](http://www.ncbi.nlm.nih.gov/entrez/query.fcgi?cmd=search&db=gene&term=Prkcb) | protein kinase C, beta | 1.3e-06 | 231.8 | 135.08 | 1.72 |
| 3 |  | [Oxidative Stress Induced Gene Expression Via Nrf2](http://cgap.nci.nih.gov/Pathways/BioCarta/m_arenrf2Pathway) | [Gsta2](http://www.ncbi.nlm.nih.gov/entrez/query.fcgi?cmd=search&db=gene&term=Gsta2) | glutathione S-transferase, alpha 2 (Yc2) | 5.2e-06 | 634.09 | 402.79 | 1.57 |
| 4 |  | [Oxidative Stress Induced Gene Expression Via Nrf2](http://cgap.nci.nih.gov/Pathways/BioCarta/m_arenrf2Pathway) | [Jun](http://www.ncbi.nlm.nih.gov/entrez/query.fcgi?cmd=search&db=gene&term=Jun) | Jun oncogene | 7.6e-06 | 325.38 | 205.78 | 1.58 |
| 5 |  | [Oxidative Stress Induced Gene Expression Via Nrf2](http://cgap.nci.nih.gov/Pathways/BioCarta/m_arenrf2Pathway) | [Mapk8](http://www.ncbi.nlm.nih.gov/entrez/query.fcgi?cmd=search&db=gene&term=Mapk8) | mitogen-activated protein kinase 8 | 0.0235064 | 29.15 | 25.43 | 1.15 |
| 6 |  | [Oxidative Stress Induced Gene Expression Via Nrf2](http://cgap.nci.nih.gov/Pathways/BioCarta/m_arenrf2Pathway) | [Creb1](http://www.ncbi.nlm.nih.gov/entrez/query.fcgi?cmd=search&db=gene&term=Creb1) | cAMP responsive element binding protein 1 | 0.0492112 | 44.74 | 50.82 | 0.88 |
| 7 |  | [Oxidative Stress Induced Gene Expression Via Nrf2](http://cgap.nci.nih.gov/Pathways/BioCarta/m_arenrf2Pathway) | [Mapk1](http://www.ncbi.nlm.nih.gov/entrez/query.fcgi?cmd=search&db=gene&term=Mapk1) | mitogen-activated protein kinase 1 | 0.0854614 | 196.98 | 247.01 | 0.8 |
| 8 |  | [Oxidative Stress Induced Gene Expression Via Nrf2](http://cgap.nci.nih.gov/Pathways/BioCarta/m_arenrf2Pathway) | [Mapk14](http://www.ncbi.nlm.nih.gov/entrez/query.fcgi?cmd=search&db=gene&term=Mapk14) | mitogen-activated protein kinase 14 | 0.2051723 | 79.08 | 71.64 | 1.1 |
| 1 | m_tollpathway | [Toll-Like Receptor Pathway](http://cgap.nci.nih.gov/Pathways/BioCarta/m_tollpathway) | [Fos](http://www.ncbi.nlm.nih.gov/entrez/query.fcgi?cmd=search&db=gene&term=Fos) | FBJ osteosarcoma oncogene | < 1e-07 | 2482.37 | 294.09 | 8.44 |
| 2 |  | [Toll-Like Receptor Pathway](http://cgap.nci.nih.gov/Pathways/BioCarta/m_tollpathway) | [Nfkbia](http://www.ncbi.nlm.nih.gov/entrez/query.fcgi?cmd=search&db=gene&term=Nfkbia) | nuclear factor of kappa light polypeptide gene enhancer in B-cells inhibitor, alpha | 4.2e-06 | 1283.3 | 852.99 | 1.5 |
| 3 |  | [Toll-Like Receptor Pathway](http://cgap.nci.nih.gov/Pathways/BioCarta/m_tollpathway) | [Jun](http://www.ncbi.nlm.nih.gov/entrez/query.fcgi?cmd=search&db=gene&term=Jun) | Jun oncogene | 7.6e-06 | 325.38 | 205.78 | 1.58 |
| 4 |  | [Toll-Like Receptor Pathway](http://cgap.nci.nih.gov/Pathways/BioCarta/m_tollpathway) | [Ppara](http://www.ncbi.nlm.nih.gov/entrez/query.fcgi?cmd=search&db=gene&term=Ppara) | peroxisome proliferator activated receptor alpha | 0.0001792 | 269.41 | 188.35 | 1.43 |
| 5 |  | [Toll-Like Receptor Pathway](http://cgap.nci.nih.gov/Pathways/BioCarta/m_tollpathway) | [Map2k6](http://www.ncbi.nlm.nih.gov/entrez/query.fcgi?cmd=search&db=gene&term=Map2k6) | mitogen-activated protein kinase kinase 6 | 0.0003031 | 142.59 | 113.45 | 1.26 |
| 6 |  | [Toll-Like Receptor Pathway](http://cgap.nci.nih.gov/Pathways/BioCarta/m_tollpathway) | [Map2k4](http://www.ncbi.nlm.nih.gov/entrez/query.fcgi?cmd=search&db=gene&term=Map2k4) | mitogen-activated protein kinase kinase 4 | 0.0037785 | 400.03 | 495.55 | 0.81 |
| 7 |  | [Toll-Like Receptor Pathway](http://cgap.nci.nih.gov/Pathways/BioCarta/m_tollpathway) | [Nfkb1](http://www.ncbi.nlm.nih.gov/entrez/query.fcgi?cmd=search&db=gene&term=Nfkb1) | nuclear factor of kappa light polypeptide gene enhancer in B-cells 1, p105 | 0.0055039 | 708.56 | 804.29 | 0.88 |
| 8 |  | [Toll-Like Receptor Pathway](http://cgap.nci.nih.gov/Pathways/BioCarta/m_tollpathway) | [Mapk8](http://www.ncbi.nlm.nih.gov/entrez/query.fcgi?cmd=search&db=gene&term=Mapk8) | mitogen-activated protein kinase 8 | 0.0235064 | 29.15 | 25.43 | 1.15 |
| 9 |  | [Toll-Like Receptor Pathway](http://cgap.nci.nih.gov/Pathways/BioCarta/m_tollpathway) | [Pglyrp1](http://www.ncbi.nlm.nih.gov/entrez/query.fcgi?cmd=search&db=gene&term=Pglyrp1) | peptidoglycan recognition protein 1 | 0.0746578 | 15.94 | 13.77 | 1.16 |
| 10 |  | [Toll-Like Receptor Pathway](http://cgap.nci.nih.gov/Pathways/BioCarta/m_tollpathway) | [Mapk14](http://www.ncbi.nlm.nih.gov/entrez/query.fcgi?cmd=search&db=gene&term=Mapk14) | mitogen-activated protein kinase 14 | 0.2051723 | 79.08 | 71.64 | 1.1 |
| 1 | m_TPOPathway | [TPO Signaling Pathway](http://cgap.nci.nih.gov/Pathways/BioCarta/m_TPOPathway) | [Fos](http://www.ncbi.nlm.nih.gov/entrez/query.fcgi?cmd=search&db=gene&term=Fos) | FBJ osteosarcoma oncogene | < 1e-07 | 2482.37 | 294.09 | 8.44 |
| 2 |  | [TPO Signaling Pathway](http://cgap.nci.nih.gov/Pathways/BioCarta/m_TPOPathway) | [Prkcb](http://www.ncbi.nlm.nih.gov/entrez/query.fcgi?cmd=search&db=gene&term=Prkcb) | protein kinase C, beta | 1.3e-06 | 231.8 | 135.08 | 1.72 |
| 3 |  | [TPO Signaling Pathway](http://cgap.nci.nih.gov/Pathways/BioCarta/m_TPOPathway) | [Jun](http://www.ncbi.nlm.nih.gov/entrez/query.fcgi?cmd=search&db=gene&term=Jun) | Jun oncogene | 7.6e-06 | 325.38 | 205.78 | 1.58 |
| 4 |  | [TPO Signaling Pathway](http://cgap.nci.nih.gov/Pathways/BioCarta/m_TPOPathway) | [Stat1](http://www.ncbi.nlm.nih.gov/entrez/query.fcgi?cmd=search&db=gene&term=Stat1) | signal transducer and activator of transcription 1 | 1.47e-05 | 589.81 | 394.42 | 1.5 |
| 5 |  | [TPO Signaling Pathway](http://cgap.nci.nih.gov/Pathways/BioCarta/m_TPOPathway) | [Pik3r1](http://www.ncbi.nlm.nih.gov/entrez/query.fcgi?cmd=search&db=gene&term=Pik3r1) | phosphatidylinositol 3-kinase, regulatory subunit, polypeptide 1 (p85 alpha) | 0.0011172 | 134.94 | 110.05 | 1.23 |
| 6 |  | [TPO Signaling Pathway](http://cgap.nci.nih.gov/Pathways/BioCarta/m_TPOPathway) | [Csnk2a2](http://www.ncbi.nlm.nih.gov/entrez/query.fcgi?cmd=search&db=gene&term=Csnk2a2) | casein kinase 2, alpha prime polypeptide | 0.0020382 | 2035.41 | 1719.9 | 1.18 |
| 7 |  | [TPO Signaling Pathway](http://cgap.nci.nih.gov/Pathways/BioCarta/m_TPOPathway) | [Csnk2a1](http://www.ncbi.nlm.nih.gov/entrez/query.fcgi?cmd=search&db=gene&term=Csnk2a1) | casein kinase 2, alpha 1 polypeptide | 0.0029286 | 200.24 | 272.9 | 0.73 |
| 8 |  | [TPO Signaling Pathway](http://cgap.nci.nih.gov/Pathways/BioCarta/m_TPOPathway) | [Pik3cg](http://www.ncbi.nlm.nih.gov/entrez/query.fcgi?cmd=search&db=gene&term=Pik3cg) | phosphoinositide-3-kinase, catalytic, gamma polypeptide | 0.0302332 | 18.24 | 14.76 | 1.24 |
| 9 |  | [TPO Signaling Pathway](http://cgap.nci.nih.gov/Pathways/BioCarta/m_TPOPathway) | [Sos1](http://www.ncbi.nlm.nih.gov/entrez/query.fcgi?cmd=search&db=gene&term=Sos1) | son of sevenless homolog 1 (Drosophila) | 0.0657446 | 82.16 | 76.11 | 1.08 |
| 10 |  | [TPO Signaling Pathway](http://cgap.nci.nih.gov/Pathways/BioCarta/m_TPOPathway) | [Mpl](http://www.ncbi.nlm.nih.gov/entrez/query.fcgi?cmd=search&db=gene&term=Mpl) | myeloproliferative leukemia virus oncogene | 0.0989211 | 22.22 | 20.1 | 1.11 |
| 11 |  | [TPO Signaling Pathway](http://cgap.nci.nih.gov/Pathways/BioCarta/m_TPOPathway) | [Stat5a](http://www.ncbi.nlm.nih.gov/entrez/query.fcgi?cmd=search&db=gene&term=Stat5a) | signal transducer and activator of transcription 5A | 0.4703697 | 76.75 | 73.88 | 1.04 |
| 1 | m_Ccr5Pathway | [Pertussis toxin-insensitive CCR5 Signaling in Macrophage](http://cgap.nci.nih.gov/Pathways/BioCarta/m_Ccr5Pathway) | [Fos](http://www.ncbi.nlm.nih.gov/entrez/query.fcgi?cmd=search&db=gene&term=Fos) | FBJ osteosarcoma oncogene | < 1e-07 | 2482.37 | 294.09 | 8.44 |
| 2 |  | [Pertussis toxin-insensitive CCR5 Signaling in Macrophage](http://cgap.nci.nih.gov/Pathways/BioCarta/m_Ccr5Pathway) | [Cxcl12](http://www.ncbi.nlm.nih.gov/entrez/query.fcgi?cmd=search&db=gene&term=Cxcl12) | chemokine (C-X-C motif) ligand 12 | 6e-07 | 89.92 | 43.22 | 2.08 |
| 3 |  | [Pertussis toxin-insensitive CCR5 Signaling in Macrophage](http://cgap.nci.nih.gov/Pathways/BioCarta/m_Ccr5Pathway) | [Prkcb](http://www.ncbi.nlm.nih.gov/entrez/query.fcgi?cmd=search&db=gene&term=Prkcb) | protein kinase C, beta | 1.3e-06 | 231.8 | 135.08 | 1.72 |
| 4 |  | [Pertussis toxin-insensitive CCR5 Signaling in Macrophage](http://cgap.nci.nih.gov/Pathways/BioCarta/m_Ccr5Pathway) | [Jun](http://www.ncbi.nlm.nih.gov/entrez/query.fcgi?cmd=search&db=gene&term=Jun) | Jun oncogene | 7.6e-06 | 325.38 | 205.78 | 1.58 |
| 5 |  | [Pertussis toxin-insensitive CCR5 Signaling in Macrophage](http://cgap.nci.nih.gov/Pathways/BioCarta/m_Ccr5Pathway) | [Mapk8](http://www.ncbi.nlm.nih.gov/entrez/query.fcgi?cmd=search&db=gene&term=Mapk8) | mitogen-activated protein kinase 8 | 0.0235064 | 29.15 | 25.43 | 1.15 |
| 6 |  | [Pertussis toxin-insensitive CCR5 Signaling in Macrophage](http://cgap.nci.nih.gov/Pathways/BioCarta/m_Ccr5Pathway) | [Mapk14](http://www.ncbi.nlm.nih.gov/entrez/query.fcgi?cmd=search&db=gene&term=Mapk14) | mitogen-activated protein kinase 14 | 0.2051723 | 79.08 | 71.64 | 1.1 |
| 7 |  | [Pertussis toxin-insensitive CCR5 Signaling in Macrophage](http://cgap.nci.nih.gov/Pathways/BioCarta/m_Ccr5Pathway) | [Ccl4](http://www.ncbi.nlm.nih.gov/entrez/query.fcgi?cmd=search&db=gene&term=Ccl4) | chemokine (C-C motif) ligand 4 | 0.3797163 | 69.72 | 64.65 | 1.08 |
| 8 |  | [Pertussis toxin-insensitive CCR5 Signaling in Macrophage](http://cgap.nci.nih.gov/Pathways/BioCarta/m_Ccr5Pathway) | [Cxcr4](http://www.ncbi.nlm.nih.gov/entrez/query.fcgi?cmd=search&db=gene&term=Cxcr4) | chemokine (C-X-C motif) receptor 4 | 0.6196968 | 9.8 | 9.27 | 1.06 |
| 1 | m_igf1Pathway | [IGF-1 Signaling Pathway](http://cgap.nci.nih.gov/Pathways/BioCarta/m_igf1Pathway) | [Fos](http://www.ncbi.nlm.nih.gov/entrez/query.fcgi?cmd=search&db=gene&term=Fos) | FBJ osteosarcoma oncogene | < 1e-07 | 2482.37 | 294.09 | 8.44 |
| 2 |  | [IGF-1 Signaling Pathway](http://cgap.nci.nih.gov/Pathways/BioCarta/m_igf1Pathway) | [Irs1](http://www.ncbi.nlm.nih.gov/entrez/query.fcgi?cmd=search&db=gene&term=Irs1) | insulin receptor substrate 1 | 1.2e-06 | 652.96 | 397.14 | 1.64 |
| 3 |  | [IGF-1 Signaling Pathway](http://cgap.nci.nih.gov/Pathways/BioCarta/m_igf1Pathway) | [Jun](http://www.ncbi.nlm.nih.gov/entrez/query.fcgi?cmd=search&db=gene&term=Jun) | Jun oncogene | 7.6e-06 | 325.38 | 205.78 | 1.58 |
| 4 |  | [IGF-1 Signaling Pathway](http://cgap.nci.nih.gov/Pathways/BioCarta/m_igf1Pathway) | [Ptpn11](http://www.ncbi.nlm.nih.gov/entrez/query.fcgi?cmd=search&db=gene&term=Ptpn11) | protein tyrosine phosphatase, non-receptor type 11 | 0.0002035 | 49.82 | 67.58 | 0.74 |
| 5 |  | [IGF-1 Signaling Pathway](http://cgap.nci.nih.gov/Pathways/BioCarta/m_igf1Pathway) | [Pik3r1](http://www.ncbi.nlm.nih.gov/entrez/query.fcgi?cmd=search&db=gene&term=Pik3r1) | phosphatidylinositol 3-kinase, regulatory subunit, polypeptide 1 (p85 alpha) | 0.0011172 | 134.94 | 110.05 | 1.23 |
| 6 |  | [IGF-1 Signaling Pathway](http://cgap.nci.nih.gov/Pathways/BioCarta/m_igf1Pathway) | [Csnk2a2](http://www.ncbi.nlm.nih.gov/entrez/query.fcgi?cmd=search&db=gene&term=Csnk2a2) | casein kinase 2, alpha prime polypeptide | 0.0020382 | 2035.41 | 1719.9 | 1.18 |
| 7 |  | [IGF-1 Signaling Pathway](http://cgap.nci.nih.gov/Pathways/BioCarta/m_igf1Pathway) | [Csnk2a1](http://www.ncbi.nlm.nih.gov/entrez/query.fcgi?cmd=search&db=gene&term=Csnk2a1) | casein kinase 2, alpha 1 polypeptide | 0.0029286 | 200.24 | 272.9 | 0.73 |
| 8 |  | [IGF-1 Signaling Pathway](http://cgap.nci.nih.gov/Pathways/BioCarta/m_igf1Pathway) | [Mapk8](http://www.ncbi.nlm.nih.gov/entrez/query.fcgi?cmd=search&db=gene&term=Mapk8) | mitogen-activated protein kinase 8 | 0.0235064 | 29.15 | 25.43 | 1.15 |
| 9 |  | [IGF-1 Signaling Pathway](http://cgap.nci.nih.gov/Pathways/BioCarta/m_igf1Pathway) | [Pik3cg](http://www.ncbi.nlm.nih.gov/entrez/query.fcgi?cmd=search&db=gene&term=Pik3cg) | phosphoinositide-3-kinase, catalytic, gamma polypeptide | 0.0302332 | 18.24 | 14.76 | 1.24 |
| 10 |  | [IGF-1 Signaling Pathway](http://cgap.nci.nih.gov/Pathways/BioCarta/m_igf1Pathway) | [Sos1](http://www.ncbi.nlm.nih.gov/entrez/query.fcgi?cmd=search&db=gene&term=Sos1) | son of sevenless homolog 1 (Drosophila) | 0.0657446 | 82.16 | 76.11 | 1.08 |
| 11 |  | [IGF-1 Signaling Pathway](http://cgap.nci.nih.gov/Pathways/BioCarta/m_igf1Pathway) | [Igf1r](http://www.ncbi.nlm.nih.gov/entrez/query.fcgi?cmd=search&db=gene&term=Igf1r) | insulin-like growth factor I receptor | 0.251263 | 33.81 | 31.96 | 1.06 |
| 1 | m_cardiacegfPathway | [Role of EGF Receptor Transactivation by GPCRs in Cardiac Hypertrophy](http://cgap.nci.nih.gov/Pathways/BioCarta/m_cardiacegfPathway) | [Fos](http://www.ncbi.nlm.nih.gov/entrez/query.fcgi?cmd=search&db=gene&term=Fos) | FBJ osteosarcoma oncogene | < 1e-07 | 2482.37 | 294.09 | 8.44 |
| 2 |  | [Role of EGF Receptor Transactivation by GPCRs in Cardiac Hypertrophy](http://cgap.nci.nih.gov/Pathways/BioCarta/m_cardiacegfPathway) | [Prkcb](http://www.ncbi.nlm.nih.gov/entrez/query.fcgi?cmd=search&db=gene&term=Prkcb) | protein kinase C, beta | 1.3e-06 | 231.8 | 135.08 | 1.72 |
| 3 |  | [Role of EGF Receptor Transactivation by GPCRs in Cardiac Hypertrophy](http://cgap.nci.nih.gov/Pathways/BioCarta/m_cardiacegfPathway) | [Jun](http://www.ncbi.nlm.nih.gov/entrez/query.fcgi?cmd=search&db=gene&term=Jun) | Jun oncogene | 7.6e-06 | 325.38 | 205.78 | 1.58 |
| 4 |  | [Role of EGF Receptor Transactivation by GPCRs in Cardiac Hypertrophy](http://cgap.nci.nih.gov/Pathways/BioCarta/m_cardiacegfPathway) | [Edn1](http://www.ncbi.nlm.nih.gov/entrez/query.fcgi?cmd=search&db=gene&term=Edn1) | endothelin 1 | 0.0002898 | 80.6 | 56.14 | 1.44 |
| 5 |  | [Role of EGF Receptor Transactivation by GPCRs in Cardiac Hypertrophy](http://cgap.nci.nih.gov/Pathways/BioCarta/m_cardiacegfPathway) | [Nfkb1](http://www.ncbi.nlm.nih.gov/entrez/query.fcgi?cmd=search&db=gene&term=Nfkb1) | nuclear factor of kappa light polypeptide gene enhancer in B-cells 1, p105 | 0.0055039 | 708.56 | 804.29 | 0.88 |
| 6 |  | [Role of EGF Receptor Transactivation by GPCRs in Cardiac Hypertrophy](http://cgap.nci.nih.gov/Pathways/BioCarta/m_cardiacegfPathway) | [Egf](http://www.ncbi.nlm.nih.gov/entrez/query.fcgi?cmd=search&db=gene&term=Egf) | epidermal growth factor | 0.1278655 | 12.86 | 11.05 | 1.16 |
| 7 |  | [Role of EGF Receptor Transactivation by GPCRs in Cardiac Hypertrophy](http://cgap.nci.nih.gov/Pathways/BioCarta/m_cardiacegfPathway) | [Ednra](http://www.ncbi.nlm.nih.gov/entrez/query.fcgi?cmd=search&db=gene&term=Ednra) | endothelin receptor type A | 0.9116664 | 8.91 | 8.97 | 0.99 |
| 1 | m_etsPathway | [METS affect on Macrophage Differentiation](http://cgap.nci.nih.gov/Pathways/BioCarta/m_etsPathway) | [Fos](http://www.ncbi.nlm.nih.gov/entrez/query.fcgi?cmd=search&db=gene&term=Fos) | FBJ osteosarcoma oncogene | < 1e-07 | 2482.37 | 294.09 | 8.44 |
| 2 |  | [METS affect on Macrophage Differentiation](http://cgap.nci.nih.gov/Pathways/BioCarta/m_etsPathway) | [Jun](http://www.ncbi.nlm.nih.gov/entrez/query.fcgi?cmd=search&db=gene&term=Jun) | Jun oncogene | 7.6e-06 | 325.38 | 205.78 | 1.58 |
| 3 |  | [METS affect on Macrophage Differentiation](http://cgap.nci.nih.gov/Pathways/BioCarta/m_etsPathway) | [Csf1r](http://www.ncbi.nlm.nih.gov/entrez/query.fcgi?cmd=search&db=gene&term=Csf1r) | colony stimulating factor 1 receptor | 3.32e-05 | 1176.61 | 829.17 | 1.42 |
| 4 |  | [METS affect on Macrophage Differentiation](http://cgap.nci.nih.gov/Pathways/BioCarta/m_etsPathway) | [Ets1](http://www.ncbi.nlm.nih.gov/entrez/query.fcgi?cmd=search&db=gene&term=Ets1) | E26 avian leukemia oncogene 1, 5' domain | 4.29e-05 | 92.32 | 130.49 | 0.71 |
| 5 |  | [METS affect on Macrophage Differentiation](http://cgap.nci.nih.gov/Pathways/BioCarta/m_etsPathway) | [Hdac5](http://www.ncbi.nlm.nih.gov/entrez/query.fcgi?cmd=search&db=gene&term=Hdac5) | histone deacetylase 5 | 9.14e-05 | 708.71 | 547.32 | 1.29 |
| 6 |  | [METS affect on Macrophage Differentiation](http://cgap.nci.nih.gov/Pathways/BioCarta/m_etsPathway) | [Sin3b](http://www.ncbi.nlm.nih.gov/entrez/query.fcgi?cmd=search&db=gene&term=Sin3b) | transcriptional regulator, SIN3B (yeast) | 0.0055566 | 39.21 | 32.02 | 1.22 |
| 7 |  | [METS affect on Macrophage Differentiation](http://cgap.nci.nih.gov/Pathways/BioCarta/m_etsPathway) | [Csf1](http://www.ncbi.nlm.nih.gov/entrez/query.fcgi?cmd=search&db=gene&term=Csf1) | colony stimulating factor 1 (macrophage) | 0.005888 | 24.61 | 29.17 | 0.84 |
| 8 |  | [METS affect on Macrophage Differentiation](http://cgap.nci.nih.gov/Pathways/BioCarta/m_etsPathway) | [Sin3a](http://www.ncbi.nlm.nih.gov/entrez/query.fcgi?cmd=search&db=gene&term=Sin3a) | transcriptional regulator, SIN3A (yeast) | 0.064544 | 129.7 | 148.64 | 0.87 |
| 9 |  | [METS affect on Macrophage Differentiation](http://cgap.nci.nih.gov/Pathways/BioCarta/m_etsPathway) | [Rbl1](http://www.ncbi.nlm.nih.gov/entrez/query.fcgi?cmd=search&db=gene&term=Rbl1) | retinoblastoma-like 1 (p107) | 0.1036563 | 19.15 | 22.45 | 0.85 |
| 10 |  | [METS affect on Macrophage Differentiation](http://cgap.nci.nih.gov/Pathways/BioCarta/m_etsPathway) | [Etv3](http://www.ncbi.nlm.nih.gov/entrez/query.fcgi?cmd=search&db=gene&term=Etv3) | ets variant gene 3 | 0.1939366 | 629.8 | 581.33 | 1.08 |
| 1 | m_keratinocytePathway | [Keratinocyte Differentiation](http://cgap.nci.nih.gov/Pathways/BioCarta/m_keratinocytePathway) | [Fos](http://www.ncbi.nlm.nih.gov/entrez/query.fcgi?cmd=search&db=gene&term=Fos) | FBJ osteosarcoma oncogene | < 1e-07 | 2482.37 | 294.09 | 8.44 |
| 2 |  | [Keratinocyte Differentiation](http://cgap.nci.nih.gov/Pathways/BioCarta/m_keratinocytePathway) | [Prkcb](http://www.ncbi.nlm.nih.gov/entrez/query.fcgi?cmd=search&db=gene&term=Prkcb) | protein kinase C, beta | 1.3e-06 | 231.8 | 135.08 | 1.72 |
| 3 |  | [Keratinocyte Differentiation](http://cgap.nci.nih.gov/Pathways/BioCarta/m_keratinocytePathway) | [Nfkbia](http://www.ncbi.nlm.nih.gov/entrez/query.fcgi?cmd=search&db=gene&term=Nfkbia) | nuclear factor of kappa light polypeptide gene enhancer in B-cells inhibitor, alpha | 4.2e-06 | 1283.3 | 852.99 | 1.5 |
| 4 |  | [Keratinocyte Differentiation](http://cgap.nci.nih.gov/Pathways/BioCarta/m_keratinocytePathway) | [Jun](http://www.ncbi.nlm.nih.gov/entrez/query.fcgi?cmd=search&db=gene&term=Jun) | Jun oncogene | 7.6e-06 | 325.38 | 205.78 | 1.58 |
| 5 |  | [Keratinocyte Differentiation](http://cgap.nci.nih.gov/Pathways/BioCarta/m_keratinocytePathway) | [Ets1](http://www.ncbi.nlm.nih.gov/entrez/query.fcgi?cmd=search&db=gene&term=Ets1) | E26 avian leukemia oncogene 1, 5' domain | 4.29e-05 | 92.32 | 130.49 | 0.71 |
| 6 |  | [Keratinocyte Differentiation](http://cgap.nci.nih.gov/Pathways/BioCarta/m_keratinocytePathway) | [Map2k6](http://www.ncbi.nlm.nih.gov/entrez/query.fcgi?cmd=search&db=gene&term=Map2k6) | mitogen-activated protein kinase kinase 6 | 0.0003031 | 142.59 | 113.45 | 1.26 |
| 7 |  | [Keratinocyte Differentiation](http://cgap.nci.nih.gov/Pathways/BioCarta/m_keratinocytePathway) | [Cebpa](http://www.ncbi.nlm.nih.gov/entrez/query.fcgi?cmd=search&db=gene&term=Cebpa) | CCAAT/enhancer binding protein (C/EBP), alpha | 0.000687 | 3772.79 | 3131.75 | 1.2 |
| 8 |  | [Keratinocyte Differentiation](http://cgap.nci.nih.gov/Pathways/BioCarta/m_keratinocytePathway) | [Prkcq](http://www.ncbi.nlm.nih.gov/entrez/query.fcgi?cmd=search&db=gene&term=Prkcq) | protein kinase C, theta | 0.0012006 | 139.55 | 182.42 | 0.77 |
| 9 |  | [Keratinocyte Differentiation](http://cgap.nci.nih.gov/Pathways/BioCarta/m_keratinocytePathway) | [Prkch](http://www.ncbi.nlm.nih.gov/entrez/query.fcgi?cmd=search&db=gene&term=Prkch) | protein kinase C, eta | 0.0013622 | 279.44 | 228.53 | 1.22 |
| 10 |  | [Keratinocyte Differentiation](http://cgap.nci.nih.gov/Pathways/BioCarta/m_keratinocytePathway) | [Map2k4](http://www.ncbi.nlm.nih.gov/entrez/query.fcgi?cmd=search&db=gene&term=Map2k4) | mitogen-activated protein kinase kinase 4 | 0.0037785 | 400.03 | 495.55 | 0.81 |
| 11 |  | [Keratinocyte Differentiation](http://cgap.nci.nih.gov/Pathways/BioCarta/m_keratinocytePathway) | [Nfkb1](http://www.ncbi.nlm.nih.gov/entrez/query.fcgi?cmd=search&db=gene&term=Nfkb1) | nuclear factor of kappa light polypeptide gene enhancer in B-cells 1, p105 | 0.0055039 | 708.56 | 804.29 | 0.88 |
| 12 |  | [Keratinocyte Differentiation](http://cgap.nci.nih.gov/Pathways/BioCarta/m_keratinocytePathway) | [Prkcd](http://www.ncbi.nlm.nih.gov/entrez/query.fcgi?cmd=search&db=gene&term=Prkcd) | protein kinase C, delta | 0.0098367 | 920.89 | 809.95 | 1.14 |
| 13 |  | [Keratinocyte Differentiation](http://cgap.nci.nih.gov/Pathways/BioCarta/m_keratinocytePathway) | [Mapk8](http://www.ncbi.nlm.nih.gov/entrez/query.fcgi?cmd=search&db=gene&term=Mapk8) | mitogen-activated protein kinase 8 | 0.0235064 | 29.15 | 25.43 | 1.15 |
| 14 |  | [Keratinocyte Differentiation](http://cgap.nci.nih.gov/Pathways/BioCarta/m_keratinocytePathway) | [Map3k5](http://www.ncbi.nlm.nih.gov/entrez/query.fcgi?cmd=search&db=gene&term=Map3k5) | mitogen-activated protein kinase kinase kinase 5 | 0.0440586 | 54.99 | 65.09 | 0.84 |
| 15 |  | [Keratinocyte Differentiation](http://cgap.nci.nih.gov/Pathways/BioCarta/m_keratinocytePathway) | [Mapk1](http://www.ncbi.nlm.nih.gov/entrez/query.fcgi?cmd=search&db=gene&term=Mapk1) | mitogen-activated protein kinase 1 | 0.0854614 | 196.98 | 247.01 | 0.8 |
| 16 |  | [Keratinocyte Differentiation](http://cgap.nci.nih.gov/Pathways/BioCarta/m_keratinocytePathway) | [Egf](http://www.ncbi.nlm.nih.gov/entrez/query.fcgi?cmd=search&db=gene&term=Egf) | epidermal growth factor | 0.1278655 | 12.86 | 11.05 | 1.16 |
| 17 |  | [Keratinocyte Differentiation](http://cgap.nci.nih.gov/Pathways/BioCarta/m_keratinocytePathway) | [Mapk14](http://www.ncbi.nlm.nih.gov/entrez/query.fcgi?cmd=search&db=gene&term=Mapk14) | mitogen-activated protein kinase 14 | 0.2051723 | 79.08 | 71.64 | 1.1 |
| 18 |  | [Keratinocyte Differentiation](http://cgap.nci.nih.gov/Pathways/BioCarta/m_keratinocytePathway) | [Tnf](http://www.ncbi.nlm.nih.gov/entrez/query.fcgi?cmd=search&db=gene&term=Tnf) | tumor necrosis factor | 0.3010041 | 58.77 | 63.31 | 0.93 |
| 19 |  | [Keratinocyte Differentiation](http://cgap.nci.nih.gov/Pathways/BioCarta/m_keratinocytePathway) | [Prkce](http://www.ncbi.nlm.nih.gov/entrez/query.fcgi?cmd=search&db=gene&term=Prkce) | protein kinase C, epsilon | 0.9565615 | 38.64 | 38.47 | 1 |
| 1 | m_pdgfPathway | [PDGF Signaling Pathway](http://cgap.nci.nih.gov/Pathways/BioCarta/m_pdgfPathway) | [Fos](http://www.ncbi.nlm.nih.gov/entrez/query.fcgi?cmd=search&db=gene&term=Fos) | FBJ osteosarcoma oncogene | < 1e-07 | 2482.37 | 294.09 | 8.44 |
| 2 |  | [PDGF Signaling Pathway](http://cgap.nci.nih.gov/Pathways/BioCarta/m_pdgfPathway) | [Prkcb](http://www.ncbi.nlm.nih.gov/entrez/query.fcgi?cmd=search&db=gene&term=Prkcb) | protein kinase C, beta | 1.3e-06 | 231.8 | 135.08 | 1.72 |
| 3 |  | [PDGF Signaling Pathway](http://cgap.nci.nih.gov/Pathways/BioCarta/m_pdgfPathway) | [Jun](http://www.ncbi.nlm.nih.gov/entrez/query.fcgi?cmd=search&db=gene&term=Jun) | Jun oncogene | 7.6e-06 | 325.38 | 205.78 | 1.58 |
| 4 |  | [PDGF Signaling Pathway](http://cgap.nci.nih.gov/Pathways/BioCarta/m_pdgfPathway) | [Stat1](http://www.ncbi.nlm.nih.gov/entrez/query.fcgi?cmd=search&db=gene&term=Stat1) | signal transducer and activator of transcription 1 | 1.47e-05 | 589.81 | 394.42 | 1.5 |
| 5 |  | [PDGF Signaling Pathway](http://cgap.nci.nih.gov/Pathways/BioCarta/m_pdgfPathway) | [Pik3r1](http://www.ncbi.nlm.nih.gov/entrez/query.fcgi?cmd=search&db=gene&term=Pik3r1) | phosphatidylinositol 3-kinase, regulatory subunit, polypeptide 1 (p85 alpha) | 0.0011172 | 134.94 | 110.05 | 1.23 |
| 6 |  | [PDGF Signaling Pathway](http://cgap.nci.nih.gov/Pathways/BioCarta/m_pdgfPathway) | [Csnk2a2](http://www.ncbi.nlm.nih.gov/entrez/query.fcgi?cmd=search&db=gene&term=Csnk2a2) | casein kinase 2, alpha prime polypeptide | 0.0020382 | 2035.41 | 1719.9 | 1.18 |
| 7 |  | [PDGF Signaling Pathway](http://cgap.nci.nih.gov/Pathways/BioCarta/m_pdgfPathway) | [Csnk2a1](http://www.ncbi.nlm.nih.gov/entrez/query.fcgi?cmd=search&db=gene&term=Csnk2a1) | casein kinase 2, alpha 1 polypeptide | 0.0029286 | 200.24 | 272.9 | 0.73 |
| 8 |  | [PDGF Signaling Pathway](http://cgap.nci.nih.gov/Pathways/BioCarta/m_pdgfPathway) | [Map2k4](http://www.ncbi.nlm.nih.gov/entrez/query.fcgi?cmd=search&db=gene&term=Map2k4) | mitogen-activated protein kinase kinase 4 | 0.0037785 | 400.03 | 495.55 | 0.81 |
| 9 |  | [PDGF Signaling Pathway](http://cgap.nci.nih.gov/Pathways/BioCarta/m_pdgfPathway) | [Mapk8](http://www.ncbi.nlm.nih.gov/entrez/query.fcgi?cmd=search&db=gene&term=Mapk8) | mitogen-activated protein kinase 8 | 0.0235064 | 29.15 | 25.43 | 1.15 |
| 10 |  | [PDGF Signaling Pathway](http://cgap.nci.nih.gov/Pathways/BioCarta/m_pdgfPathway) | [Pik3cg](http://www.ncbi.nlm.nih.gov/entrez/query.fcgi?cmd=search&db=gene&term=Pik3cg) | phosphoinositide-3-kinase, catalytic, gamma polypeptide | 0.0302332 | 18.24 | 14.76 | 1.24 |
| 11 |  | [PDGF Signaling Pathway](http://cgap.nci.nih.gov/Pathways/BioCarta/m_pdgfPathway) | [Sos1](http://www.ncbi.nlm.nih.gov/entrez/query.fcgi?cmd=search&db=gene&term=Sos1) | son of sevenless homolog 1 (Drosophila) | 0.0657446 | 82.16 | 76.11 | 1.08 |
| 12 |  | [PDGF Signaling Pathway](http://cgap.nci.nih.gov/Pathways/BioCarta/m_pdgfPathway) | [Stat5a](http://www.ncbi.nlm.nih.gov/entrez/query.fcgi?cmd=search&db=gene&term=Stat5a) | signal transducer and activator of transcription 5A | 0.4703697 | 76.75 | 73.88 | 1.04 |
| 13 |  | [PDGF Signaling Pathway](http://cgap.nci.nih.gov/Pathways/BioCarta/m_pdgfPathway) | [Jak1](http://www.ncbi.nlm.nih.gov/entrez/query.fcgi?cmd=search&db=gene&term=Jak1) | Janus kinase 1 | 0.5074408 | 151.14 | 156.56 | 0.97 |
| 1 | m_ngfPathway | [Nerve growth factor pathway (NGF)](http://cgap.nci.nih.gov/Pathways/BioCarta/m_ngfPathway) | [Fos](http://www.ncbi.nlm.nih.gov/entrez/query.fcgi?cmd=search&db=gene&term=Fos) | FBJ osteosarcoma oncogene | < 1e-07 | 2482.37 | 294.09 | 8.44 |
| 2 |  | [Nerve growth factor pathway (NGF)](http://cgap.nci.nih.gov/Pathways/BioCarta/m_ngfPathway) | [Jun](http://www.ncbi.nlm.nih.gov/entrez/query.fcgi?cmd=search&db=gene&term=Jun) | Jun oncogene | 7.6e-06 | 325.38 | 205.78 | 1.58 |
| 3 |  | [Nerve growth factor pathway (NGF)](http://cgap.nci.nih.gov/Pathways/BioCarta/m_ngfPathway) | [Ngfr](http://www.ncbi.nlm.nih.gov/entrez/query.fcgi?cmd=search&db=gene&term=Ngfr) | nerve growth factor receptor (TNFR superfamily, member 16) | 0.0001535 | 243.71 | 189.19 | 1.29 |
| 4 |  | [Nerve growth factor pathway (NGF)](http://cgap.nci.nih.gov/Pathways/BioCarta/m_ngfPathway) | [Pik3r1](http://www.ncbi.nlm.nih.gov/entrez/query.fcgi?cmd=search&db=gene&term=Pik3r1) | phosphatidylinositol 3-kinase, regulatory subunit, polypeptide 1 (p85 alpha) | 0.0011172 | 134.94 | 110.05 | 1.23 |
| 5 |  | [Nerve growth factor pathway (NGF)](http://cgap.nci.nih.gov/Pathways/BioCarta/m_ngfPathway) | [Csnk2a2](http://www.ncbi.nlm.nih.gov/entrez/query.fcgi?cmd=search&db=gene&term=Csnk2a2) | casein kinase 2, alpha prime polypeptide | 0.0020382 | 2035.41 | 1719.9 | 1.18 |
| 6 |  | [Nerve growth factor pathway (NGF)](http://cgap.nci.nih.gov/Pathways/BioCarta/m_ngfPathway) | [Csnk2a1](http://www.ncbi.nlm.nih.gov/entrez/query.fcgi?cmd=search&db=gene&term=Csnk2a1) | casein kinase 2, alpha 1 polypeptide | 0.0029286 | 200.24 | 272.9 | 0.73 |
| 7 |  | [Nerve growth factor pathway (NGF)](http://cgap.nci.nih.gov/Pathways/BioCarta/m_ngfPathway) | [Ngf](http://www.ncbi.nlm.nih.gov/entrez/query.fcgi?cmd=search&db=gene&term=Ngf) | nerve growth factor | 0.0034743 | 41.98 | 72.78 | 0.58 |
| 8 |  | [Nerve growth factor pathway (NGF)](http://cgap.nci.nih.gov/Pathways/BioCarta/m_ngfPathway) | [Mapk8](http://www.ncbi.nlm.nih.gov/entrez/query.fcgi?cmd=search&db=gene&term=Mapk8) | mitogen-activated protein kinase 8 | 0.0235064 | 29.15 | 25.43 | 1.15 |
| 9 |  | [Nerve growth factor pathway (NGF)](http://cgap.nci.nih.gov/Pathways/BioCarta/m_ngfPathway) | [Pik3cg](http://www.ncbi.nlm.nih.gov/entrez/query.fcgi?cmd=search&db=gene&term=Pik3cg) | phosphoinositide-3-kinase, catalytic, gamma polypeptide | 0.0302332 | 18.24 | 14.76 | 1.24 |
| 10 |  | [Nerve growth factor pathway (NGF)](http://cgap.nci.nih.gov/Pathways/BioCarta/m_ngfPathway) | [Sos1](http://www.ncbi.nlm.nih.gov/entrez/query.fcgi?cmd=search&db=gene&term=Sos1) | son of sevenless homolog 1 (Drosophila) | 0.0657446 | 82.16 | 76.11 | 1.08 |
| 1 | m_egfPathway | [EGF Signaling Pathway](http://cgap.nci.nih.gov/Pathways/BioCarta/m_egfPathway) | [Fos](http://www.ncbi.nlm.nih.gov/entrez/query.fcgi?cmd=search&db=gene&term=Fos) | FBJ osteosarcoma oncogene | < 1e-07 | 2482.37 | 294.09 | 8.44 |
| 2 |  | [EGF Signaling Pathway](http://cgap.nci.nih.gov/Pathways/BioCarta/m_egfPathway) | [Prkcb](http://www.ncbi.nlm.nih.gov/entrez/query.fcgi?cmd=search&db=gene&term=Prkcb) | protein kinase C, beta | 1.3e-06 | 231.8 | 135.08 | 1.72 |
| 3 |  | [EGF Signaling Pathway](http://cgap.nci.nih.gov/Pathways/BioCarta/m_egfPathway) | [Jun](http://www.ncbi.nlm.nih.gov/entrez/query.fcgi?cmd=search&db=gene&term=Jun) | Jun oncogene | 7.6e-06 | 325.38 | 205.78 | 1.58 |
| 4 |  | [EGF Signaling Pathway](http://cgap.nci.nih.gov/Pathways/BioCarta/m_egfPathway) | [Stat1](http://www.ncbi.nlm.nih.gov/entrez/query.fcgi?cmd=search&db=gene&term=Stat1) | signal transducer and activator of transcription 1 | 1.47e-05 | 589.81 | 394.42 | 1.5 |
| 5 |  | [EGF Signaling Pathway](http://cgap.nci.nih.gov/Pathways/BioCarta/m_egfPathway) | [Pik3r1](http://www.ncbi.nlm.nih.gov/entrez/query.fcgi?cmd=search&db=gene&term=Pik3r1) | phosphatidylinositol 3-kinase, regulatory subunit, polypeptide 1 (p85 alpha) | 0.0011172 | 134.94 | 110.05 | 1.23 |
| 6 |  | [EGF Signaling Pathway](http://cgap.nci.nih.gov/Pathways/BioCarta/m_egfPathway) | [Csnk2a2](http://www.ncbi.nlm.nih.gov/entrez/query.fcgi?cmd=search&db=gene&term=Csnk2a2) | casein kinase 2, alpha prime polypeptide | 0.0020382 | 2035.41 | 1719.9 | 1.18 |
| 7 |  | [EGF Signaling Pathway](http://cgap.nci.nih.gov/Pathways/BioCarta/m_egfPathway) | [Csnk2a1](http://www.ncbi.nlm.nih.gov/entrez/query.fcgi?cmd=search&db=gene&term=Csnk2a1) | casein kinase 2, alpha 1 polypeptide | 0.0029286 | 200.24 | 272.9 | 0.73 |
| 8 |  | [EGF Signaling Pathway](http://cgap.nci.nih.gov/Pathways/BioCarta/m_egfPathway) | [Map2k4](http://www.ncbi.nlm.nih.gov/entrez/query.fcgi?cmd=search&db=gene&term=Map2k4) | mitogen-activated protein kinase kinase 4 | 0.0037785 | 400.03 | 495.55 | 0.81 |
| 9 |  | [EGF Signaling Pathway](http://cgap.nci.nih.gov/Pathways/BioCarta/m_egfPathway) | [Mapk8](http://www.ncbi.nlm.nih.gov/entrez/query.fcgi?cmd=search&db=gene&term=Mapk8) | mitogen-activated protein kinase 8 | 0.0235064 | 29.15 | 25.43 | 1.15 |
| 10 |  | [EGF Signaling Pathway](http://cgap.nci.nih.gov/Pathways/BioCarta/m_egfPathway) | [Sos1](http://www.ncbi.nlm.nih.gov/entrez/query.fcgi?cmd=search&db=gene&term=Sos1) | son of sevenless homolog 1 (Drosophila) | 0.0657446 | 82.16 | 76.11 | 1.08 |
| 11 |  | [EGF Signaling Pathway](http://cgap.nci.nih.gov/Pathways/BioCarta/m_egfPathway) | [Egf](http://www.ncbi.nlm.nih.gov/entrez/query.fcgi?cmd=search&db=gene&term=Egf) | epidermal growth factor | 0.1278655 | 12.86 | 11.05 | 1.16 |
| 12 |  | [EGF Signaling Pathway](http://cgap.nci.nih.gov/Pathways/BioCarta/m_egfPathway) | [Stat5a](http://www.ncbi.nlm.nih.gov/entrez/query.fcgi?cmd=search&db=gene&term=Stat5a) | signal transducer and activator of transcription 5A | 0.4703697 | 76.75 | 73.88 | 1.04 |
| 13 |  | [EGF Signaling Pathway](http://cgap.nci.nih.gov/Pathways/BioCarta/m_egfPathway) | [Jak1](http://www.ncbi.nlm.nih.gov/entrez/query.fcgi?cmd=search&db=gene&term=Jak1) | Janus kinase 1 | 0.5074408 | 151.14 | 156.56 | 0.97 |
| 1 | m_ctlPathway | [CTL mediated immune response against target cells](http://cgap.nci.nih.gov/Pathways/BioCarta/m_ctlPathway) | [H2-D1](http://www.ncbi.nlm.nih.gov/entrez/query.fcgi?cmd=search&db=gene&term=H2-D1) | histocompatibility 2, D region locus 1 | < 1e-07 | 2751.11 | 57.77 | 47.62 |
| 2 |  | [CTL mediated immune response against target cells](http://cgap.nci.nih.gov/Pathways/BioCarta/m_ctlPathway) | [H2-K1](http://www.ncbi.nlm.nih.gov/entrez/query.fcgi?cmd=search&db=gene&term=H2-K1) | histocompatibility 2, K1, K region | < 1e-07 | 496.79 | 78.88 | 6.3 |
| 3 |  | [CTL mediated immune response against target cells](http://cgap.nci.nih.gov/Pathways/BioCarta/m_ctlPathway) | [Cd3g](http://www.ncbi.nlm.nih.gov/entrez/query.fcgi?cmd=search&db=gene&term=Cd3g) | CD3 antigen, gamma polypeptide | 0.0006733 | 228.71 | 181.14 | 1.26 |
| 4 |  | [CTL mediated immune response against target cells](http://cgap.nci.nih.gov/Pathways/BioCarta/m_ctlPathway) | [Tcra](http://www.ncbi.nlm.nih.gov/entrez/query.fcgi?cmd=search&db=gene&term=Tcra) | T-cell receptor alpha chain | 0.0010497 | 30.87 | 24.36 | 1.27 |
| 5 |  | [CTL mediated immune response against target cells](http://cgap.nci.nih.gov/Pathways/BioCarta/m_ctlPathway) | [Gzmb](http://www.ncbi.nlm.nih.gov/entrez/query.fcgi?cmd=search&db=gene&term=Gzmb) | granzyme B | 0.0021411 | 27.4 | 22.72 | 1.21 |
| 6 |  | [CTL mediated immune response against target cells](http://cgap.nci.nih.gov/Pathways/BioCarta/m_ctlPathway) | [Prf1](http://www.ncbi.nlm.nih.gov/entrez/query.fcgi?cmd=search&db=gene&term=Prf1) | perforin 1 (pore forming protein) | 0.0022755 | 72.94 | 57.61 | 1.27 |
| 7 |  | [CTL mediated immune response against target cells](http://cgap.nci.nih.gov/Pathways/BioCarta/m_ctlPathway) | [Itgb2](http://www.ncbi.nlm.nih.gov/entrez/query.fcgi?cmd=search&db=gene&term=Itgb2) | integrin beta 2 | 0.0051773 | 75.8 | 59.67 | 1.27 |
| 8 |  | [CTL mediated immune response against target cells](http://cgap.nci.nih.gov/Pathways/BioCarta/m_ctlPathway) | [Cd3d](http://www.ncbi.nlm.nih.gov/entrez/query.fcgi?cmd=search&db=gene&term=Cd3d) | CD3 antigen, delta polypeptide | 0.0636825 | 116.66 | 106.54 | 1.1 |
| 9 |  | [CTL mediated immune response against target cells](http://cgap.nci.nih.gov/Pathways/BioCarta/m_ctlPathway) | [Cd247](http://www.ncbi.nlm.nih.gov/entrez/query.fcgi?cmd=search&db=gene&term=Cd247) | CD247 antigen | 0.3675336 | 12.93 | 13.92 | 0.93 |
| 10 |  | [CTL mediated immune response against target cells](http://cgap.nci.nih.gov/Pathways/BioCarta/m_ctlPathway) | [Icam1](http://www.ncbi.nlm.nih.gov/entrez/query.fcgi?cmd=search&db=gene&term=Icam1) | intercellular adhesion molecule 1 | 0.5889182 | 128.64 | 131.2 | 0.98 |
| 1 | m_tcrPathway | [T Cell Receptor Signaling Pathway](http://cgap.nci.nih.gov/Pathways/BioCarta/m_tcrPathway) | [Fos](http://www.ncbi.nlm.nih.gov/entrez/query.fcgi?cmd=search&db=gene&term=Fos) | FBJ osteosarcoma oncogene | < 1e-07 | 2482.37 | 294.09 | 8.44 |
| 2 |  | [T Cell Receptor Signaling Pathway](http://cgap.nci.nih.gov/Pathways/BioCarta/m_tcrPathway) | [Prkcb](http://www.ncbi.nlm.nih.gov/entrez/query.fcgi?cmd=search&db=gene&term=Prkcb) | protein kinase C, beta | 1.3e-06 | 231.8 | 135.08 | 1.72 |
| 3 |  | [T Cell Receptor Signaling Pathway](http://cgap.nci.nih.gov/Pathways/BioCarta/m_tcrPathway) | [Nfkbia](http://www.ncbi.nlm.nih.gov/entrez/query.fcgi?cmd=search&db=gene&term=Nfkbia) | nuclear factor of kappa light polypeptide gene enhancer in B-cells inhibitor, alpha | 4.2e-06 | 1283.3 | 852.99 | 1.5 |
| 4 |  | [T Cell Receptor Signaling Pathway](http://cgap.nci.nih.gov/Pathways/BioCarta/m_tcrPathway) | [Jun](http://www.ncbi.nlm.nih.gov/entrez/query.fcgi?cmd=search&db=gene&term=Jun) | Jun oncogene | 7.6e-06 | 325.38 | 205.78 | 1.58 |
| 5 |  | [T Cell Receptor Signaling Pathway](http://cgap.nci.nih.gov/Pathways/BioCarta/m_tcrPathway) | [Cd3g](http://www.ncbi.nlm.nih.gov/entrez/query.fcgi?cmd=search&db=gene&term=Cd3g) | CD3 antigen, gamma polypeptide | 0.0006733 | 228.71 | 181.14 | 1.26 |
| 6 |  | [T Cell Receptor Signaling Pathway](http://cgap.nci.nih.gov/Pathways/BioCarta/m_tcrPathway) | [Tcra](http://www.ncbi.nlm.nih.gov/entrez/query.fcgi?cmd=search&db=gene&term=Tcra) | T-cell receptor alpha chain | 0.0010497 | 30.87 | 24.36 | 1.27 |
| 7 |  | [T Cell Receptor Signaling Pathway](http://cgap.nci.nih.gov/Pathways/BioCarta/m_tcrPathway) | [Pik3r1](http://www.ncbi.nlm.nih.gov/entrez/query.fcgi?cmd=search&db=gene&term=Pik3r1) | phosphatidylinositol 3-kinase, regulatory subunit, polypeptide 1 (p85 alpha) | 0.0011172 | 134.94 | 110.05 | 1.23 |
| 8 |  | [T Cell Receptor Signaling Pathway](http://cgap.nci.nih.gov/Pathways/BioCarta/m_tcrPathway) | [Map2k4](http://www.ncbi.nlm.nih.gov/entrez/query.fcgi?cmd=search&db=gene&term=Map2k4) | mitogen-activated protein kinase kinase 4 | 0.0037785 | 400.03 | 495.55 | 0.81 |
| 9 |  | [T Cell Receptor Signaling Pathway](http://cgap.nci.nih.gov/Pathways/BioCarta/m_tcrPathway) | [Lck](http://www.ncbi.nlm.nih.gov/entrez/query.fcgi?cmd=search&db=gene&term=Lck) | lymphocyte protein tyrosine kinase | 0.0037891 | 81.12 | 69.9 | 1.16 |
| 10 |  | [T Cell Receptor Signaling Pathway](http://cgap.nci.nih.gov/Pathways/BioCarta/m_tcrPathway) | [Nfkb1](http://www.ncbi.nlm.nih.gov/entrez/query.fcgi?cmd=search&db=gene&term=Nfkb1) | nuclear factor of kappa light polypeptide gene enhancer in B-cells 1, p105 | 0.0055039 | 708.56 | 804.29 | 0.88 |
| 11 |  | [T Cell Receptor Signaling Pathway](http://cgap.nci.nih.gov/Pathways/BioCarta/m_tcrPathway) | [Vav1](http://www.ncbi.nlm.nih.gov/entrez/query.fcgi?cmd=search&db=gene&term=Vav1) | vav 1 oncogene | 0.005748 | 23.69 | 19.16 | 1.24 |
| 12 |  | [T Cell Receptor Signaling Pathway](http://cgap.nci.nih.gov/Pathways/BioCarta/m_tcrPathway) | [Zap70](http://www.ncbi.nlm.nih.gov/entrez/query.fcgi?cmd=search&db=gene&term=Zap70) | zeta-chain (TCR) associated protein kinase | 0.0073629 | 503.81 | 424.72 | 1.19 |
| 13 |  | [T Cell Receptor Signaling Pathway](http://cgap.nci.nih.gov/Pathways/BioCarta/m_tcrPathway) | [Fyn](http://www.ncbi.nlm.nih.gov/entrez/query.fcgi?cmd=search&db=gene&term=Fyn) | Fyn proto-oncogene | 0.012453 | 99.23 | 88.59 | 1.12 |
| 14 |  | [T Cell Receptor Signaling Pathway](http://cgap.nci.nih.gov/Pathways/BioCarta/m_tcrPathway) | [Mapk8](http://www.ncbi.nlm.nih.gov/entrez/query.fcgi?cmd=search&db=gene&term=Mapk8) | mitogen-activated protein kinase 8 | 0.0235064 | 29.15 | 25.43 | 1.15 |
| 15 |  | [T Cell Receptor Signaling Pathway](http://cgap.nci.nih.gov/Pathways/BioCarta/m_tcrPathway) | [Pik3cg](http://www.ncbi.nlm.nih.gov/entrez/query.fcgi?cmd=search&db=gene&term=Pik3cg) | phosphoinositide-3-kinase, catalytic, gamma polypeptide | 0.0302332 | 18.24 | 14.76 | 1.24 |
| 16 |  | [T Cell Receptor Signaling Pathway](http://cgap.nci.nih.gov/Pathways/BioCarta/m_tcrPathway) | [Nfatc3](http://www.ncbi.nlm.nih.gov/entrez/query.fcgi?cmd=search&db=gene&term=Nfatc3) | nuclear factor of activated T-cells, cytoplasmic, calcineurin-dependent 3 | 0.0370984 | 207.98 | 241.23 | 0.86 |
| 17 |  | [T Cell Receptor Signaling Pathway](http://cgap.nci.nih.gov/Pathways/BioCarta/m_tcrPathway) | [Cd3d](http://www.ncbi.nlm.nih.gov/entrez/query.fcgi?cmd=search&db=gene&term=Cd3d) | CD3 antigen, delta polypeptide | 0.0636825 | 116.66 | 106.54 | 1.1 |
| 18 |  | [T Cell Receptor Signaling Pathway](http://cgap.nci.nih.gov/Pathways/BioCarta/m_tcrPathway) | [Sos1](http://www.ncbi.nlm.nih.gov/entrez/query.fcgi?cmd=search&db=gene&term=Sos1) | son of sevenless homolog 1 (Drosophila) | 0.0657446 | 82.16 | 76.11 | 1.08 |
| 19 |  | [T Cell Receptor Signaling Pathway](http://cgap.nci.nih.gov/Pathways/BioCarta/m_tcrPathway) | [Cd247](http://www.ncbi.nlm.nih.gov/entrez/query.fcgi?cmd=search&db=gene&term=Cd247) | CD247 antigen | 0.3675336 | 12.93 | 13.92 | 0.93 |
| 20 |  | [T Cell Receptor Signaling Pathway](http://cgap.nci.nih.gov/Pathways/BioCarta/m_tcrPathway) | [Nfatc2](http://www.ncbi.nlm.nih.gov/entrez/query.fcgi?cmd=search&db=gene&term=Nfatc2) | nuclear factor of activated T-cells, cytoplasmic, calcineurin-dependent 2 | 0.520464 | 17.55 | 16.65 | 1.05 |
| 1 | m_tsp1Pathway | [TSP-1 Induced Apoptosis in Microvascular Endothelial Cell](http://cgap.nci.nih.gov/Pathways/BioCarta/m_tsp1Pathway) | [Fos](http://www.ncbi.nlm.nih.gov/entrez/query.fcgi?cmd=search&db=gene&term=Fos) | FBJ osteosarcoma oncogene | < 1e-07 | 2482.37 | 294.09 | 8.44 |
| 2 |  | [TSP-1 Induced Apoptosis in Microvascular Endothelial Cell](http://cgap.nci.nih.gov/Pathways/BioCarta/m_tsp1Pathway) | [Jun](http://www.ncbi.nlm.nih.gov/entrez/query.fcgi?cmd=search&db=gene&term=Jun) | Jun oncogene | 7.6e-06 | 325.38 | 205.78 | 1.58 |
| 3 |  | [TSP-1 Induced Apoptosis in Microvascular Endothelial Cell](http://cgap.nci.nih.gov/Pathways/BioCarta/m_tsp1Pathway) | [Thbs1](http://www.ncbi.nlm.nih.gov/entrez/query.fcgi?cmd=search&db=gene&term=Thbs1) | thrombospondin 1 | 0.0003554 | 145.8 | 196.89 | 0.74 |
| 4 |  | [TSP-1 Induced Apoptosis in Microvascular Endothelial Cell](http://cgap.nci.nih.gov/Pathways/BioCarta/m_tsp1Pathway) | [Fyn](http://www.ncbi.nlm.nih.gov/entrez/query.fcgi?cmd=search&db=gene&term=Fyn) | Fyn proto-oncogene | 0.012453 | 99.23 | 88.59 | 1.12 |
| 5 |  | [TSP-1 Induced Apoptosis in Microvascular Endothelial Cell](http://cgap.nci.nih.gov/Pathways/BioCarta/m_tsp1Pathway) | [Cd36](http://www.ncbi.nlm.nih.gov/entrez/query.fcgi?cmd=search&db=gene&term=Cd36) | CD36 antigen | 0.0138767 | 88.3 | 76.85 | 1.15 |
| 6 |  | [TSP-1 Induced Apoptosis in Microvascular Endothelial Cell](http://cgap.nci.nih.gov/Pathways/BioCarta/m_tsp1Pathway) | [Mapk14](http://www.ncbi.nlm.nih.gov/entrez/query.fcgi?cmd=search&db=gene&term=Mapk14) | mitogen-activated protein kinase 14 | 0.2051723 | 79.08 | 71.64 | 1.1 |
| 1 | m_tnfr2Pathway | [TNFR2 Signaling Pathway](http://cgap.nci.nih.gov/Pathways/BioCarta/m_tnfr2Pathway) | [Dusp1](http://www.ncbi.nlm.nih.gov/entrez/query.fcgi?cmd=search&db=gene&term=Dusp1) | dual specificity phosphatase 1 | < 1e-07 | 2280.02 | 673.69 | 3.38 |
| 2 |  | [TNFR2 Signaling Pathway](http://cgap.nci.nih.gov/Pathways/BioCarta/m_tnfr2Pathway) | [Nfkbia](http://www.ncbi.nlm.nih.gov/entrez/query.fcgi?cmd=search&db=gene&term=Nfkbia) | nuclear factor of kappa light polypeptide gene enhancer in B-cells inhibitor, alpha | 4.2e-06 | 1283.3 | 852.99 | 1.5 |
| 3 |  | [TNFR2 Signaling Pathway](http://cgap.nci.nih.gov/Pathways/BioCarta/m_tnfr2Pathway) | [Nfkb1](http://www.ncbi.nlm.nih.gov/entrez/query.fcgi?cmd=search&db=gene&term=Nfkb1) | nuclear factor of kappa light polypeptide gene enhancer in B-cells 1, p105 | 0.0055039 | 708.56 | 804.29 | 0.88 |
| 4 |  | [TNFR2 Signaling Pathway](http://cgap.nci.nih.gov/Pathways/BioCarta/m_tnfr2Pathway) | [Ikbkap](http://www.ncbi.nlm.nih.gov/entrez/query.fcgi?cmd=search&db=gene&term=Ikbkap) | inhibitor of kappa light polypeptide enhancer in B-cells, kinase complex-associated protein | 0.036169 | 68.75 | 83.19 | 0.83 |
| 5 |  | [TNFR2 Signaling Pathway](http://cgap.nci.nih.gov/Pathways/BioCarta/m_tnfr2Pathway) | [Tnfaip3](http://www.ncbi.nlm.nih.gov/entrez/query.fcgi?cmd=search&db=gene&term=Tnfaip3) | tumor necrosis factor, alpha-induced protein 3 | 0.1387187 | 80.55 | 86.44 | 0.93 |
| 1 | m_mef2dPathway | [Role of MEF2D in T-cell Apoptosis](http://cgap.nci.nih.gov/Pathways/BioCarta/m_mef2dPathway) | [Prkcb](http://www.ncbi.nlm.nih.gov/entrez/query.fcgi?cmd=search&db=gene&term=Prkcb) | protein kinase C, beta | 1.3e-06 | 231.8 | 135.08 | 1.72 |
| 2 |  | [Role of MEF2D in T-cell Apoptosis](http://cgap.nci.nih.gov/Pathways/BioCarta/m_mef2dPathway) | [Capn2](http://www.ncbi.nlm.nih.gov/entrez/query.fcgi?cmd=search&db=gene&term=Capn2) | calpain 2 | 1.55e-05 | 266.92 | 403.74 | 0.66 |
| 3 |  | [Role of MEF2D in T-cell Apoptosis](http://cgap.nci.nih.gov/Pathways/BioCarta/m_mef2dPathway) | [Capns1](http://www.ncbi.nlm.nih.gov/entrez/query.fcgi?cmd=search&db=gene&term=Capns1) | calpain, small subunit 1 | 0.0002753 | 728.59 | 916.71 | 0.79 |
| 4 |  | [Role of MEF2D in T-cell Apoptosis](http://cgap.nci.nih.gov/Pathways/BioCarta/m_mef2dPathway) | [Tcra](http://www.ncbi.nlm.nih.gov/entrez/query.fcgi?cmd=search&db=gene&term=Tcra) | T-cell receptor alpha chain | 0.0010497 | 30.87 | 24.36 | 1.27 |
| 5 |  | [Role of MEF2D in T-cell Apoptosis](http://cgap.nci.nih.gov/Pathways/BioCarta/m_mef2dPathway) | [Nfatc2](http://www.ncbi.nlm.nih.gov/entrez/query.fcgi?cmd=search&db=gene&term=Nfatc2) | nuclear factor of activated T-cells, cytoplasmic, calcineurin-dependent 2 | 0.520464 | 17.55 | 16.65 | 1.05 |
| 1 | m_cdk5Pathway | [Phosphorylation of MEK1 by cdk5/p35 down regulates the MAP kinase pathway](http://cgap.nci.nih.gov/Pathways/BioCarta/m_cdk5Pathway) | [Egr1](http://www.ncbi.nlm.nih.gov/entrez/query.fcgi?cmd=search&db=gene&term=Egr1) | early growth response 1 | 2e-07 | 2821.17 | 1401.3 | 2.01 |
| 2 |  | [Phosphorylation of MEK1 by cdk5/p35 down regulates the MAP kinase pathway](http://cgap.nci.nih.gov/Pathways/BioCarta/m_cdk5Pathway) | [Ngfr](http://www.ncbi.nlm.nih.gov/entrez/query.fcgi?cmd=search&db=gene&term=Ngfr) | nerve growth factor receptor (TNFR superfamily, member 16) | 0.0001535 | 243.71 | 189.19 | 1.29 |
| 3 |  | [Phosphorylation of MEK1 by cdk5/p35 down regulates the MAP kinase pathway](http://cgap.nci.nih.gov/Pathways/BioCarta/m_cdk5Pathway) | [Cdk5r1](http://www.ncbi.nlm.nih.gov/entrez/query.fcgi?cmd=search&db=gene&term=Cdk5r1) | cyclin-dependent kinase 5, regulatory subunit 1 (p35) | 0.0002963 | 54 | 40.71 | 1.33 |
| 4 |  | [Phosphorylation of MEK1 by cdk5/p35 down regulates the MAP kinase pathway](http://cgap.nci.nih.gov/Pathways/BioCarta/m_cdk5Pathway) | [Ngf](http://www.ncbi.nlm.nih.gov/entrez/query.fcgi?cmd=search&db=gene&term=Ngf) | nerve growth factor | 0.0034743 | 41.98 | 72.78 | 0.58 |
| 5 |  | [Phosphorylation of MEK1 by cdk5/p35 down regulates the MAP kinase pathway](http://cgap.nci.nih.gov/Pathways/BioCarta/m_cdk5Pathway) | [Mapk1](http://www.ncbi.nlm.nih.gov/entrez/query.fcgi?cmd=search&db=gene&term=Mapk1) | mitogen-activated protein kinase 1 | 0.0854614 | 196.98 | 247.01 | 0.8 |
| 1 | m_cd40Pathway | [CD40L Signaling Pathway](http://cgap.nci.nih.gov/Pathways/BioCarta/m_cd40Pathway) | [Dusp1](http://www.ncbi.nlm.nih.gov/entrez/query.fcgi?cmd=search&db=gene&term=Dusp1) | dual specificity phosphatase 1 | < 1e-07 | 2280.02 | 673.69 | 3.38 |
| 2 |  | [CD40L Signaling Pathway](http://cgap.nci.nih.gov/Pathways/BioCarta/m_cd40Pathway) | [Nfkbia](http://www.ncbi.nlm.nih.gov/entrez/query.fcgi?cmd=search&db=gene&term=Nfkbia) | nuclear factor of kappa light polypeptide gene enhancer in B-cells inhibitor, alpha | 4.2e-06 | 1283.3 | 852.99 | 1.5 |
| 3 |  | [CD40L Signaling Pathway](http://cgap.nci.nih.gov/Pathways/BioCarta/m_cd40Pathway) | [Nfkb1](http://www.ncbi.nlm.nih.gov/entrez/query.fcgi?cmd=search&db=gene&term=Nfkb1) | nuclear factor of kappa light polypeptide gene enhancer in B-cells 1, p105 | 0.0055039 | 708.56 | 804.29 | 0.88 |
| 4 |  | [CD40L Signaling Pathway](http://cgap.nci.nih.gov/Pathways/BioCarta/m_cd40Pathway) | [Cd40lg](http://www.ncbi.nlm.nih.gov/entrez/query.fcgi?cmd=search&db=gene&term=Cd40lg) | CD40 ligand | 0.1265964 | 34.93 | 31.48 | 1.11 |
| 5 |  | [CD40L Signaling Pathway](http://cgap.nci.nih.gov/Pathways/BioCarta/m_cd40Pathway) | [Tnfaip3](http://www.ncbi.nlm.nih.gov/entrez/query.fcgi?cmd=search&db=gene&term=Tnfaip3) | tumor necrosis factor, alpha-induced protein 3 | 0.1387187 | 80.55 | 86.44 | 0.93 |
| 1 | m_d4gdiPathway | [D4-GDI Signaling Pathway](http://cgap.nci.nih.gov/Pathways/BioCarta/m_d4gdiPathway) | [Casp1](http://www.ncbi.nlm.nih.gov/entrez/query.fcgi?cmd=search&db=gene&term=Casp1) | caspase 1 | 3.7e-06 | 357.28 | 218.62 | 1.63 |
| 2 |  | [D4-GDI Signaling Pathway](http://cgap.nci.nih.gov/Pathways/BioCarta/m_d4gdiPathway) | [Jun](http://www.ncbi.nlm.nih.gov/entrez/query.fcgi?cmd=search&db=gene&term=Jun) | Jun oncogene | 7.6e-06 | 325.38 | 205.78 | 1.58 |
| 3 |  | [D4-GDI Signaling Pathway](http://cgap.nci.nih.gov/Pathways/BioCarta/m_d4gdiPathway) | [Gzmb](http://www.ncbi.nlm.nih.gov/entrez/query.fcgi?cmd=search&db=gene&term=Gzmb) | granzyme B | 0.0021411 | 27.4 | 22.72 | 1.21 |
| 4 |  | [D4-GDI Signaling Pathway](http://cgap.nci.nih.gov/Pathways/BioCarta/m_d4gdiPathway) | [Prf1](http://www.ncbi.nlm.nih.gov/entrez/query.fcgi?cmd=search&db=gene&term=Prf1) | perforin 1 (pore forming protein) | 0.0022755 | 72.94 | 57.61 | 1.27 |
| 5 |  | [D4-GDI Signaling Pathway](http://cgap.nci.nih.gov/Pathways/BioCarta/m_d4gdiPathway) | [Arhgap5](http://www.ncbi.nlm.nih.gov/entrez/query.fcgi?cmd=search&db=gene&term=Arhgap5) | Rho GTPase activating protein 5 | 0.0468195 | 250.1 | 288.8 | 0.87 |
| 1 | m_eradPathway | [ER¿associated degradation (ERAD) Pathway](http://cgap.nci.nih.gov/Pathways/BioCarta/m_eradPathway) | [Skp1a](http://www.ncbi.nlm.nih.gov/entrez/query.fcgi?cmd=search&db=gene&term=Skp1a) | S-phase kinase-associated protein 1A | 3.2e-05 | 653.2 | 899.69 | 0.73 |
| 2 |  | [ER¿associated degradation (ERAD) Pathway](http://cgap.nci.nih.gov/Pathways/BioCarta/m_eradPathway) | [Edem1](http://www.ncbi.nlm.nih.gov/entrez/query.fcgi?cmd=search&db=gene&term=Edem1) | ER degradation enhancer, mannosidase alpha-like 1 | 4.49e-05 | 343.12 | 463.23 | 0.74 |
| 3 |  | [ER¿associated degradation (ERAD) Pathway](http://cgap.nci.nih.gov/Pathways/BioCarta/m_eradPathway) | [Uba2](http://www.ncbi.nlm.nih.gov/entrez/query.fcgi?cmd=search&db=gene&term=Uba2) | ubiquitin-like modifier activating enzyme 2 | 0.0001159 | 765.65 | 1058.33 | 0.72 |
| 4 |  | [ER¿associated degradation (ERAD) Pathway](http://cgap.nci.nih.gov/Pathways/BioCarta/m_eradPathway) | [Man1a](http://www.ncbi.nlm.nih.gov/entrez/query.fcgi?cmd=search&db=gene&term=Man1a) | mannosidase 1, alpha | 0.0060467 | 53.9 | 74.9 | 0.72 |
| 5 |  | [ER¿associated degradation (ERAD) Pathway](http://cgap.nci.nih.gov/Pathways/BioCarta/m_eradPathway) | [Ganab](http://www.ncbi.nlm.nih.gov/entrez/query.fcgi?cmd=search&db=gene&term=Ganab) | alpha glucosidase 2 alpha neutral subunit | 0.0081557 | 8.31 | 10.46 | 0.79 |
| 1 | m_dspPathway | [Regulation of MAP Kinase Pathways Through Dual Specificity Phosphatases](http://cgap.nci.nih.gov/Pathways/BioCarta/m_dspPathway) | [Dusp1](http://www.ncbi.nlm.nih.gov/entrez/query.fcgi?cmd=search&db=gene&term=Dusp1) | dual specificity phosphatase 1 | < 1e-07 | 2280.02 | 673.69 | 3.38 |
| 2 |  | [Regulation of MAP Kinase Pathways Through Dual Specificity Phosphatases](http://cgap.nci.nih.gov/Pathways/BioCarta/m_dspPathway) | [Dusp6](http://www.ncbi.nlm.nih.gov/entrez/query.fcgi?cmd=search&db=gene&term=Dusp6) | dual specificity phosphatase 6 | 2.2e-06 | 166.28 | 266.43 | 0.62 |
| 3 |  | [Regulation of MAP Kinase Pathways Through Dual Specificity Phosphatases](http://cgap.nci.nih.gov/Pathways/BioCarta/m_dspPathway) | [Dusp2](http://www.ncbi.nlm.nih.gov/entrez/query.fcgi?cmd=search&db=gene&term=Dusp2) | dual specificity phosphatase 2 | 0.0003959 | 42.76 | 33.25 | 1.29 |
| 4 |  | [Regulation of MAP Kinase Pathways Through Dual Specificity Phosphatases](http://cgap.nci.nih.gov/Pathways/BioCarta/m_dspPathway) | [Mapk8](http://www.ncbi.nlm.nih.gov/entrez/query.fcgi?cmd=search&db=gene&term=Mapk8) | mitogen-activated protein kinase 8 | 0.0235064 | 29.15 | 25.43 | 1.15 |
| 5 |  | [Regulation of MAP Kinase Pathways Through Dual Specificity Phosphatases](http://cgap.nci.nih.gov/Pathways/BioCarta/m_dspPathway) | [Mapk14](http://www.ncbi.nlm.nih.gov/entrez/query.fcgi?cmd=search&db=gene&term=Mapk14) | mitogen-activated protein kinase 14 | 0.2051723 | 79.08 | 71.64 | 1.1 |
| 6 |  | [Regulation of MAP Kinase Pathways Through Dual Specificity Phosphatases](http://cgap.nci.nih.gov/Pathways/BioCarta/m_dspPathway) | [Dusp9](http://www.ncbi.nlm.nih.gov/entrez/query.fcgi?cmd=search&db=gene&term=Dusp9) | dual specificity phosphatase 9 | 0.6954847 | 9.82 | 10.13 | 0.97 |
| 1 | m_chemicalPathway | [Apoptotic Signaling in Response to DNA Damage](http://cgap.nci.nih.gov/Pathways/BioCarta/m_chemicalPathway) | [Prkcb](http://www.ncbi.nlm.nih.gov/entrez/query.fcgi?cmd=search&db=gene&term=Prkcb) | protein kinase C, beta | 1.3e-06 | 231.8 | 135.08 | 1.72 |
| 2 |  | [Apoptotic Signaling in Response to DNA Damage](http://cgap.nci.nih.gov/Pathways/BioCarta/m_chemicalPathway) | [Stat1](http://www.ncbi.nlm.nih.gov/entrez/query.fcgi?cmd=search&db=gene&term=Stat1) | signal transducer and activator of transcription 1 | 1.47e-05 | 589.81 | 394.42 | 1.5 |
| 3 |  | [Apoptotic Signaling in Response to DNA Damage](http://cgap.nci.nih.gov/Pathways/BioCarta/m_chemicalPathway) | [Bid](http://www.ncbi.nlm.nih.gov/entrez/query.fcgi?cmd=search&db=gene&term=Bid) | BH3 interacting domain death agonist | 0.0002221 | 79.7 | 56.96 | 1.4 |
| 4 |  | [Apoptotic Signaling in Response to DNA Damage](http://cgap.nci.nih.gov/Pathways/BioCarta/m_chemicalPathway) | [Trp53](http://www.ncbi.nlm.nih.gov/entrez/query.fcgi?cmd=search&db=gene&term=Trp53) | transformation related protein 53 | 0.0115487 | 12.39 | 14.37 | 0.86 |
| 5 |  | [Apoptotic Signaling in Response to DNA Damage](http://cgap.nci.nih.gov/Pathways/BioCarta/m_chemicalPathway) | [Eif2s1](http://www.ncbi.nlm.nih.gov/entrez/query.fcgi?cmd=search&db=gene&term=Eif2s1) | eukaryotic translation initiation factor 2, subunit 1 alpha | 0.2971571 | 19.56 | 17.93 | 1.09 |
| 1 | m_dreampathway | [Repression of Pain Sensation by the Transcriptional Regulator DREAM](http://cgap.nci.nih.gov/Pathways/BioCarta/m_dreampathway) | [Fos](http://www.ncbi.nlm.nih.gov/entrez/query.fcgi?cmd=search&db=gene&term=Fos) | FBJ osteosarcoma oncogene | < 1e-07 | 2482.37 | 294.09 | 8.44 |
| 2 |  | [Repression of Pain Sensation by the Transcriptional Regulator DREAM](http://cgap.nci.nih.gov/Pathways/BioCarta/m_dreampathway) | [Jun](http://www.ncbi.nlm.nih.gov/entrez/query.fcgi?cmd=search&db=gene&term=Jun) | Jun oncogene | 7.6e-06 | 325.38 | 205.78 | 1.58 |
| 3 |  | [Repression of Pain Sensation by the Transcriptional Regulator DREAM](http://cgap.nci.nih.gov/Pathways/BioCarta/m_dreampathway) | [Creb1](http://www.ncbi.nlm.nih.gov/entrez/query.fcgi?cmd=search&db=gene&term=Creb1) | cAMP responsive element binding protein 1 | 0.0492112 | 44.74 | 50.82 | 0.88 |
| 4 |  | [Repression of Pain Sensation by the Transcriptional Regulator DREAM](http://cgap.nci.nih.gov/Pathways/BioCarta/m_dreampathway) | [Prkacb](http://www.ncbi.nlm.nih.gov/entrez/query.fcgi?cmd=search&db=gene&term=Prkacb) | protein kinase, cAMP dependent, catalytic, beta | 0.073778 | 210.45 | 190.67 | 1.1 |
| 5 |  | [Repression of Pain Sensation by the Transcriptional Regulator DREAM](http://cgap.nci.nih.gov/Pathways/BioCarta/m_dreampathway) | [Crem](http://www.ncbi.nlm.nih.gov/entrez/query.fcgi?cmd=search&db=gene&term=Crem) | cAMP responsive element modulator | 0.0781337 | 31.01 | 35.59 | 0.87 |
| 1 | m_epoPathway | [EPO Signaling Pathway](http://cgap.nci.nih.gov/Pathways/BioCarta/m_epoPathway) | [Fos](http://www.ncbi.nlm.nih.gov/entrez/query.fcgi?cmd=search&db=gene&term=Fos) | FBJ osteosarcoma oncogene | < 1e-07 | 2482.37 | 294.09 | 8.44 |
| 2 |  | [EPO Signaling Pathway](http://cgap.nci.nih.gov/Pathways/BioCarta/m_epoPathway) | [Jun](http://www.ncbi.nlm.nih.gov/entrez/query.fcgi?cmd=search&db=gene&term=Jun) | Jun oncogene | 7.6e-06 | 325.38 | 205.78 | 1.58 |
| 3 |  | [EPO Signaling Pathway](http://cgap.nci.nih.gov/Pathways/BioCarta/m_epoPathway) | [Csnk2a2](http://www.ncbi.nlm.nih.gov/entrez/query.fcgi?cmd=search&db=gene&term=Csnk2a2) | casein kinase 2, alpha prime polypeptide | 0.0020382 | 2035.41 | 1719.9 | 1.18 |
| 4 |  | [EPO Signaling Pathway](http://cgap.nci.nih.gov/Pathways/BioCarta/m_epoPathway) | [Csnk2a1](http://www.ncbi.nlm.nih.gov/entrez/query.fcgi?cmd=search&db=gene&term=Csnk2a1) | casein kinase 2, alpha 1 polypeptide | 0.0029286 | 200.24 | 272.9 | 0.73 |
| 5 |  | [EPO Signaling Pathway](http://cgap.nci.nih.gov/Pathways/BioCarta/m_epoPathway) | [Mapk8](http://www.ncbi.nlm.nih.gov/entrez/query.fcgi?cmd=search&db=gene&term=Mapk8) | mitogen-activated protein kinase 8 | 0.0235064 | 29.15 | 25.43 | 1.15 |
| 6 |  | [EPO Signaling Pathway](http://cgap.nci.nih.gov/Pathways/BioCarta/m_epoPathway) | [Sos1](http://www.ncbi.nlm.nih.gov/entrez/query.fcgi?cmd=search&db=gene&term=Sos1) | son of sevenless homolog 1 (Drosophila) | 0.0657446 | 82.16 | 76.11 | 1.08 |
| 7 |  | [EPO Signaling Pathway](http://cgap.nci.nih.gov/Pathways/BioCarta/m_epoPathway) | [Stat5a](http://www.ncbi.nlm.nih.gov/entrez/query.fcgi?cmd=search&db=gene&term=Stat5a) | signal transducer and activator of transcription 5A | 0.4703697 | 76.75 | 73.88 | 1.04 |
| 1 | m_gleevecpathway | [Inhibition of Cellular Proliferation by Gleevec](http://cgap.nci.nih.gov/Pathways/BioCarta/m_gleevecpathway) | [Fos](http://www.ncbi.nlm.nih.gov/entrez/query.fcgi?cmd=search&db=gene&term=Fos) | FBJ osteosarcoma oncogene | < 1e-07 | 2482.37 | 294.09 | 8.44 |
| 2 |  | [Inhibition of Cellular Proliferation by Gleevec](http://cgap.nci.nih.gov/Pathways/BioCarta/m_gleevecpathway) | [Jun](http://www.ncbi.nlm.nih.gov/entrez/query.fcgi?cmd=search&db=gene&term=Jun) | Jun oncogene | 7.6e-06 | 325.38 | 205.78 | 1.58 |
| 3 |  | [Inhibition of Cellular Proliferation by Gleevec](http://cgap.nci.nih.gov/Pathways/BioCarta/m_gleevecpathway) | [Stat1](http://www.ncbi.nlm.nih.gov/entrez/query.fcgi?cmd=search&db=gene&term=Stat1) | signal transducer and activator of transcription 1 | 1.47e-05 | 589.81 | 394.42 | 1.5 |
| 4 |  | [Inhibition of Cellular Proliferation by Gleevec](http://cgap.nci.nih.gov/Pathways/BioCarta/m_gleevecpathway) | [Pik3r1](http://www.ncbi.nlm.nih.gov/entrez/query.fcgi?cmd=search&db=gene&term=Pik3r1) | phosphatidylinositol 3-kinase, regulatory subunit, polypeptide 1 (p85 alpha) | 0.0011172 | 134.94 | 110.05 | 1.23 |
| 5 |  | [Inhibition of Cellular Proliferation by Gleevec](http://cgap.nci.nih.gov/Pathways/BioCarta/m_gleevecpathway) | [Bcr](http://www.ncbi.nlm.nih.gov/entrez/query.fcgi?cmd=search&db=gene&term=Bcr) | breakpoint cluster region | 0.003165 | 165.7 | 223.1 | 0.74 |
| 6 |  | [Inhibition of Cellular Proliferation by Gleevec](http://cgap.nci.nih.gov/Pathways/BioCarta/m_gleevecpathway) | [Map2k4](http://www.ncbi.nlm.nih.gov/entrez/query.fcgi?cmd=search&db=gene&term=Map2k4) | mitogen-activated protein kinase kinase 4 | 0.0037785 | 400.03 | 495.55 | 0.81 |
| 7 |  | [Inhibition of Cellular Proliferation by Gleevec](http://cgap.nci.nih.gov/Pathways/BioCarta/m_gleevecpathway) | [Mapk8](http://www.ncbi.nlm.nih.gov/entrez/query.fcgi?cmd=search&db=gene&term=Mapk8) | mitogen-activated protein kinase 8 | 0.0235064 | 29.15 | 25.43 | 1.15 |
| 8 |  | [Inhibition of Cellular Proliferation by Gleevec](http://cgap.nci.nih.gov/Pathways/BioCarta/m_gleevecpathway) | [Pik3cg](http://www.ncbi.nlm.nih.gov/entrez/query.fcgi?cmd=search&db=gene&term=Pik3cg) | phosphoinositide-3-kinase, catalytic, gamma polypeptide | 0.0302332 | 18.24 | 14.76 | 1.24 |
| 9 |  | [Inhibition of Cellular Proliferation by Gleevec](http://cgap.nci.nih.gov/Pathways/BioCarta/m_gleevecpathway) | [Sos1](http://www.ncbi.nlm.nih.gov/entrez/query.fcgi?cmd=search&db=gene&term=Sos1) | son of sevenless homolog 1 (Drosophila) | 0.0657446 | 82.16 | 76.11 | 1.08 |
| 10 |  | [Inhibition of Cellular Proliferation by Gleevec](http://cgap.nci.nih.gov/Pathways/BioCarta/m_gleevecpathway) | [Stat5a](http://www.ncbi.nlm.nih.gov/entrez/query.fcgi?cmd=search&db=gene&term=Stat5a) | signal transducer and activator of transcription 5A | 0.4703697 | 76.75 | 73.88 | 1.04 |
| 11 |  | [Inhibition of Cellular Proliferation by Gleevec](http://cgap.nci.nih.gov/Pathways/BioCarta/m_gleevecpathway) | [Crkl](http://www.ncbi.nlm.nih.gov/entrez/query.fcgi?cmd=search&db=gene&term=Crkl) | v-crk sarcoma virus CT10 oncogene homolog (avian)-like | 0.6778896 | 18.99 | 19.51 | 0.97 |
| 1 | m_gpcrPathway | [Signaling Pathway from G-Protein Families](http://cgap.nci.nih.gov/Pathways/BioCarta/m_gpcrPathway) | [Fos](http://www.ncbi.nlm.nih.gov/entrez/query.fcgi?cmd=search&db=gene&term=Fos) | FBJ osteosarcoma oncogene | < 1e-07 | 2482.37 | 294.09 | 8.44 |
| 2 |  | [Signaling Pathway from G-Protein Families](http://cgap.nci.nih.gov/Pathways/BioCarta/m_gpcrPathway) | [Prkcb](http://www.ncbi.nlm.nih.gov/entrez/query.fcgi?cmd=search&db=gene&term=Prkcb) | protein kinase C, beta | 1.3e-06 | 231.8 | 135.08 | 1.72 |
| 3 |  | [Signaling Pathway from G-Protein Families](http://cgap.nci.nih.gov/Pathways/BioCarta/m_gpcrPathway) | [Jun](http://www.ncbi.nlm.nih.gov/entrez/query.fcgi?cmd=search&db=gene&term=Jun) | Jun oncogene | 7.6e-06 | 325.38 | 205.78 | 1.58 |
| 4 |  | [Signaling Pathway from G-Protein Families](http://cgap.nci.nih.gov/Pathways/BioCarta/m_gpcrPathway) | [Nfatc3](http://www.ncbi.nlm.nih.gov/entrez/query.fcgi?cmd=search&db=gene&term=Nfatc3) | nuclear factor of activated T-cells, cytoplasmic, calcineurin-dependent 3 | 0.0370984 | 207.98 | 241.23 | 0.86 |
| 5 |  | [Signaling Pathway from G-Protein Families](http://cgap.nci.nih.gov/Pathways/BioCarta/m_gpcrPathway) | [Creb1](http://www.ncbi.nlm.nih.gov/entrez/query.fcgi?cmd=search&db=gene&term=Creb1) | cAMP responsive element binding protein 1 | 0.0492112 | 44.74 | 50.82 | 0.88 |
| 6 |  | [Signaling Pathway from G-Protein Families](http://cgap.nci.nih.gov/Pathways/BioCarta/m_gpcrPathway) | [Prkacb](http://www.ncbi.nlm.nih.gov/entrez/query.fcgi?cmd=search&db=gene&term=Prkacb) | protein kinase, cAMP dependent, catalytic, beta | 0.073778 | 210.45 | 190.67 | 1.1 |
| 7 |  | [Signaling Pathway from G-Protein Families](http://cgap.nci.nih.gov/Pathways/BioCarta/m_gpcrPathway) | [Rps6ka3](http://www.ncbi.nlm.nih.gov/entrez/query.fcgi?cmd=search&db=gene&term=Rps6ka3) | ribosomal protein S6 kinase polypeptide 3 | 0.3653453 | 28.72 | 31.84 | 0.9 |
| 8 |  | [Signaling Pathway from G-Protein Families](http://cgap.nci.nih.gov/Pathways/BioCarta/m_gpcrPathway) | [Nfatc2](http://www.ncbi.nlm.nih.gov/entrez/query.fcgi?cmd=search&db=gene&term=Nfatc2) | nuclear factor of activated T-cells, cytoplasmic, calcineurin-dependent 2 | 0.520464 | 17.55 | 16.65 | 1.05 |
| 1 | m_fcer1Pathway | [Fc Epsilon Receptor I Signaling in Mast Cells](http://cgap.nci.nih.gov/Pathways/BioCarta/m_fcer1Pathway) | [Fos](http://www.ncbi.nlm.nih.gov/entrez/query.fcgi?cmd=search&db=gene&term=Fos) | FBJ osteosarcoma oncogene | < 1e-07 | 2482.37 | 294.09 | 8.44 |
| 2 |  | [Fc Epsilon Receptor I Signaling in Mast Cells](http://cgap.nci.nih.gov/Pathways/BioCarta/m_fcer1Pathway) | [Prkcb](http://www.ncbi.nlm.nih.gov/entrez/query.fcgi?cmd=search&db=gene&term=Prkcb) | protein kinase C, beta | 1.3e-06 | 231.8 | 135.08 | 1.72 |
| 3 |  | [Fc Epsilon Receptor I Signaling in Mast Cells](http://cgap.nci.nih.gov/Pathways/BioCarta/m_fcer1Pathway) | [Jun](http://www.ncbi.nlm.nih.gov/entrez/query.fcgi?cmd=search&db=gene&term=Jun) | Jun oncogene | 7.6e-06 | 325.38 | 205.78 | 1.58 |
| 4 |  | [Fc Epsilon Receptor I Signaling in Mast Cells](http://cgap.nci.nih.gov/Pathways/BioCarta/m_fcer1Pathway) | [Fcer1g](http://www.ncbi.nlm.nih.gov/entrez/query.fcgi?cmd=search&db=gene&term=Fcer1g) | Fc receptor, IgE, high affinity I, gamma polypeptide | 0.0003718 | 134.33 | 105.34 | 1.28 |
| 5 |  | [Fc Epsilon Receptor I Signaling in Mast Cells](http://cgap.nci.nih.gov/Pathways/BioCarta/m_fcer1Pathway) | [Pik3r1](http://www.ncbi.nlm.nih.gov/entrez/query.fcgi?cmd=search&db=gene&term=Pik3r1) | phosphatidylinositol 3-kinase, regulatory subunit, polypeptide 1 (p85 alpha) | 0.0011172 | 134.94 | 110.05 | 1.23 |
| 6 |  | [Fc Epsilon Receptor I Signaling in Mast Cells](http://cgap.nci.nih.gov/Pathways/BioCarta/m_fcer1Pathway) | [Map2k4](http://www.ncbi.nlm.nih.gov/entrez/query.fcgi?cmd=search&db=gene&term=Map2k4) | mitogen-activated protein kinase kinase 4 | 0.0037785 | 400.03 | 495.55 | 0.81 |
| 7 |  | [Fc Epsilon Receptor I Signaling in Mast Cells](http://cgap.nci.nih.gov/Pathways/BioCarta/m_fcer1Pathway) | [Vav1](http://www.ncbi.nlm.nih.gov/entrez/query.fcgi?cmd=search&db=gene&term=Vav1) | vav 1 oncogene | 0.005748 | 23.69 | 19.16 | 1.24 |
| 8 |  | [Fc Epsilon Receptor I Signaling in Mast Cells](http://cgap.nci.nih.gov/Pathways/BioCarta/m_fcer1Pathway) | [Mapk8](http://www.ncbi.nlm.nih.gov/entrez/query.fcgi?cmd=search&db=gene&term=Mapk8) | mitogen-activated protein kinase 8 | 0.0235064 | 29.15 | 25.43 | 1.15 |
| 9 |  | [Fc Epsilon Receptor I Signaling in Mast Cells](http://cgap.nci.nih.gov/Pathways/BioCarta/m_fcer1Pathway) | [Pik3cg](http://www.ncbi.nlm.nih.gov/entrez/query.fcgi?cmd=search&db=gene&term=Pik3cg) | phosphoinositide-3-kinase, catalytic, gamma polypeptide | 0.0302332 | 18.24 | 14.76 | 1.24 |
| 10 |  | [Fc Epsilon Receptor I Signaling in Mast Cells](http://cgap.nci.nih.gov/Pathways/BioCarta/m_fcer1Pathway) | [Nfatc3](http://www.ncbi.nlm.nih.gov/entrez/query.fcgi?cmd=search&db=gene&term=Nfatc3) | nuclear factor of activated T-cells, cytoplasmic, calcineurin-dependent 3 | 0.0370984 | 207.98 | 241.23 | 0.86 |
| 11 |  | [Fc Epsilon Receptor I Signaling in Mast Cells](http://cgap.nci.nih.gov/Pathways/BioCarta/m_fcer1Pathway) | [Sos1](http://www.ncbi.nlm.nih.gov/entrez/query.fcgi?cmd=search&db=gene&term=Sos1) | son of sevenless homolog 1 (Drosophila) | 0.0657446 | 82.16 | 76.11 | 1.08 |
| 12 |  | [Fc Epsilon Receptor I Signaling in Mast Cells](http://cgap.nci.nih.gov/Pathways/BioCarta/m_fcer1Pathway) | [Pak2](http://www.ncbi.nlm.nih.gov/entrez/query.fcgi?cmd=search&db=gene&term=Pak2) | p21 protein (Cdc42/Rac)-activated kinase 2 | 0.0661669 | 52.52 | 58.87 | 0.89 |
| 13 |  | [Fc Epsilon Receptor I Signaling in Mast Cells](http://cgap.nci.nih.gov/Pathways/BioCarta/m_fcer1Pathway) | [Mapk1](http://www.ncbi.nlm.nih.gov/entrez/query.fcgi?cmd=search&db=gene&term=Mapk1) | mitogen-activated protein kinase 1 | 0.0854614 | 196.98 | 247.01 | 0.8 |
| 14 |  | [Fc Epsilon Receptor I Signaling in Mast Cells](http://cgap.nci.nih.gov/Pathways/BioCarta/m_fcer1Pathway) | [Btk](http://www.ncbi.nlm.nih.gov/entrez/query.fcgi?cmd=search&db=gene&term=Btk) | Bruton agammaglobulinemia tyrosine kinase | 0.106813 | 64.07 | 57.15 | 1.12 |
| 15 |  | [Fc Epsilon Receptor I Signaling in Mast Cells](http://cgap.nci.nih.gov/Pathways/BioCarta/m_fcer1Pathway) | [Nfatc2](http://www.ncbi.nlm.nih.gov/entrez/query.fcgi?cmd=search&db=gene&term=Nfatc2) | nuclear factor of activated T-cells, cytoplasmic, calcineurin-dependent 2 | 0.520464 | 17.55 | 16.65 | 1.05 |
| 1 | m_nthiPathway | [NFkB activation by Nontypeable Hemophilus influenzae](http://cgap.nci.nih.gov/Pathways/BioCarta/m_nthiPathway) | [Dusp1](http://www.ncbi.nlm.nih.gov/entrez/query.fcgi?cmd=search&db=gene&term=Dusp1) | dual specificity phosphatase 1 | < 1e-07 | 2280.02 | 673.69 | 3.38 |
| 2 |  | [NFkB activation by Nontypeable Hemophilus influenzae](http://cgap.nci.nih.gov/Pathways/BioCarta/m_nthiPathway) | [Nfkbia](http://www.ncbi.nlm.nih.gov/entrez/query.fcgi?cmd=search&db=gene&term=Nfkbia) | nuclear factor of kappa light polypeptide gene enhancer in B-cells inhibitor, alpha | 4.2e-06 | 1283.3 | 852.99 | 1.5 |
| 3 |  | [NFkB activation by Nontypeable Hemophilus influenzae](http://cgap.nci.nih.gov/Pathways/BioCarta/m_nthiPathway) | [Smad4](http://www.ncbi.nlm.nih.gov/entrez/query.fcgi?cmd=search&db=gene&term=Smad4) | MAD homolog 4 (Drosophila) | 0.0001341 | 463.34 | 636.93 | 0.73 |
| 4 |  | [NFkB activation by Nontypeable Hemophilus influenzae](http://cgap.nci.nih.gov/Pathways/BioCarta/m_nthiPathway) | [Map2k6](http://www.ncbi.nlm.nih.gov/entrez/query.fcgi?cmd=search&db=gene&term=Map2k6) | mitogen-activated protein kinase kinase 6 | 0.0003031 | 142.59 | 113.45 | 1.26 |
| 5 |  | [NFkB activation by Nontypeable Hemophilus influenzae](http://cgap.nci.nih.gov/Pathways/BioCarta/m_nthiPathway) | [Tgfbr2](http://www.ncbi.nlm.nih.gov/entrez/query.fcgi?cmd=search&db=gene&term=Tgfbr2) | transforming growth factor, beta receptor II | 0.0004807 | 589.48 | 761.54 | 0.77 |
| 6 |  | [NFkB activation by Nontypeable Hemophilus influenzae](http://cgap.nci.nih.gov/Pathways/BioCarta/m_nthiPathway) | [Nfkb1](http://www.ncbi.nlm.nih.gov/entrez/query.fcgi?cmd=search&db=gene&term=Nfkb1) | nuclear factor of kappa light polypeptide gene enhancer in B-cells 1, p105 | 0.0055039 | 708.56 | 804.29 | 0.88 |
| 7 |  | [NFkB activation by Nontypeable Hemophilus influenzae](http://cgap.nci.nih.gov/Pathways/BioCarta/m_nthiPathway) | [Nr3c1](http://www.ncbi.nlm.nih.gov/entrez/query.fcgi?cmd=search&db=gene&term=Nr3c1) | nuclear receptor subfamily 3, group C, member 1 | 0.011527 | 128 | 102.81 | 1.25 |
| 8 |  | [NFkB activation by Nontypeable Hemophilus influenzae](http://cgap.nci.nih.gov/Pathways/BioCarta/m_nthiPathway) | [Smad3](http://www.ncbi.nlm.nih.gov/entrez/query.fcgi?cmd=search&db=gene&term=Smad3) | MAD homolog 3 (Drosophila) | 0.1793711 | 36.92 | 43.06 | 0.86 |
| 9 |  | [NFkB activation by Nontypeable Hemophilus influenzae](http://cgap.nci.nih.gov/Pathways/BioCarta/m_nthiPathway) | [Mapk14](http://www.ncbi.nlm.nih.gov/entrez/query.fcgi?cmd=search&db=gene&term=Mapk14) | mitogen-activated protein kinase 14 | 0.2051723 | 79.08 | 71.64 | 1.1 |
| 10 |  | [NFkB activation by Nontypeable Hemophilus influenzae](http://cgap.nci.nih.gov/Pathways/BioCarta/m_nthiPathway) | [Tnf](http://www.ncbi.nlm.nih.gov/entrez/query.fcgi?cmd=search&db=gene&term=Tnf) | tumor necrosis factor | 0.3010041 | 58.77 | 63.31 | 0.93 |
| 11 |  | [NFkB activation by Nontypeable Hemophilus influenzae](http://cgap.nci.nih.gov/Pathways/BioCarta/m_nthiPathway) | [Tgfbr1](http://www.ncbi.nlm.nih.gov/entrez/query.fcgi?cmd=search&db=gene&term=Tgfbr1) | transforming growth factor, beta receptor I | 0.9268967 | 9.68 | 9.73 | 0.99 |
| 1 | m_cxcr4Pathway | [CXCR4 Signaling Pathway](http://cgap.nci.nih.gov/Pathways/BioCarta/m_cxcr4Pathway) | [Cxcl12](http://www.ncbi.nlm.nih.gov/entrez/query.fcgi?cmd=search&db=gene&term=Cxcl12) | chemokine (C-X-C motif) ligand 12 | 6e-07 | 89.92 | 43.22 | 2.08 |
| 2 |  | [CXCR4 Signaling Pathway](http://cgap.nci.nih.gov/Pathways/BioCarta/m_cxcr4Pathway) | [Prkcb](http://www.ncbi.nlm.nih.gov/entrez/query.fcgi?cmd=search&db=gene&term=Prkcb) | protein kinase C, beta | 1.3e-06 | 231.8 | 135.08 | 1.72 |
| 3 |  | [CXCR4 Signaling Pathway](http://cgap.nci.nih.gov/Pathways/BioCarta/m_cxcr4Pathway) | [Pik3r1](http://www.ncbi.nlm.nih.gov/entrez/query.fcgi?cmd=search&db=gene&term=Pik3r1) | phosphatidylinositol 3-kinase, regulatory subunit, polypeptide 1 (p85 alpha) | 0.0011172 | 134.94 | 110.05 | 1.23 |
| 4 |  | [CXCR4 Signaling Pathway](http://cgap.nci.nih.gov/Pathways/BioCarta/m_cxcr4Pathway) | [Nfkb1](http://www.ncbi.nlm.nih.gov/entrez/query.fcgi?cmd=search&db=gene&term=Nfkb1) | nuclear factor of kappa light polypeptide gene enhancer in B-cells 1, p105 | 0.0055039 | 708.56 | 804.29 | 0.88 |
| 5 |  | [CXCR4 Signaling Pathway](http://cgap.nci.nih.gov/Pathways/BioCarta/m_cxcr4Pathway) | [Mapk1](http://www.ncbi.nlm.nih.gov/entrez/query.fcgi?cmd=search&db=gene&term=Mapk1) | mitogen-activated protein kinase 1 | 0.0854614 | 196.98 | 247.01 | 0.8 |
| 6 |  | [CXCR4 Signaling Pathway](http://cgap.nci.nih.gov/Pathways/BioCarta/m_cxcr4Pathway) | [Cxcr4](http://www.ncbi.nlm.nih.gov/entrez/query.fcgi?cmd=search&db=gene&term=Cxcr4) | chemokine (C-X-C motif) receptor 4 | 0.6196968 | 9.8 | 9.27 | 1.06 |
| 1 | m_bcrPathway | [BCR Signaling Pathway](http://cgap.nci.nih.gov/Pathways/BioCarta/m_bcrPathway) | [Fos](http://www.ncbi.nlm.nih.gov/entrez/query.fcgi?cmd=search&db=gene&term=Fos) | FBJ osteosarcoma oncogene | < 1e-07 | 2482.37 | 294.09 | 8.44 |
| 2 |  | [BCR Signaling Pathway](http://cgap.nci.nih.gov/Pathways/BioCarta/m_bcrPathway) | [Prkcb](http://www.ncbi.nlm.nih.gov/entrez/query.fcgi?cmd=search&db=gene&term=Prkcb) | protein kinase C, beta | 1.3e-06 | 231.8 | 135.08 | 1.72 |
| 3 |  | [BCR Signaling Pathway](http://cgap.nci.nih.gov/Pathways/BioCarta/m_bcrPathway) | [Jun](http://www.ncbi.nlm.nih.gov/entrez/query.fcgi?cmd=search&db=gene&term=Jun) | Jun oncogene | 7.6e-06 | 325.38 | 205.78 | 1.58 |
| 4 |  | [BCR Signaling Pathway](http://cgap.nci.nih.gov/Pathways/BioCarta/m_bcrPathway) | [Vav1](http://www.ncbi.nlm.nih.gov/entrez/query.fcgi?cmd=search&db=gene&term=Vav1) | vav 1 oncogene | 0.005748 | 23.69 | 19.16 | 1.24 |
| 5 |  | [BCR Signaling Pathway](http://cgap.nci.nih.gov/Pathways/BioCarta/m_bcrPathway) | [Mapk8](http://www.ncbi.nlm.nih.gov/entrez/query.fcgi?cmd=search&db=gene&term=Mapk8) | mitogen-activated protein kinase 8 | 0.0235064 | 29.15 | 25.43 | 1.15 |
| 6 |  | [BCR Signaling Pathway](http://cgap.nci.nih.gov/Pathways/BioCarta/m_bcrPathway) | [Nfatc3](http://www.ncbi.nlm.nih.gov/entrez/query.fcgi?cmd=search&db=gene&term=Nfatc3) | nuclear factor of activated T-cells, cytoplasmic, calcineurin-dependent 3 | 0.0370984 | 207.98 | 241.23 | 0.86 |
| 7 |  | [BCR Signaling Pathway](http://cgap.nci.nih.gov/Pathways/BioCarta/m_bcrPathway) | [Sos1](http://www.ncbi.nlm.nih.gov/entrez/query.fcgi?cmd=search&db=gene&term=Sos1) | son of sevenless homolog 1 (Drosophila) | 0.0657446 | 82.16 | 76.11 | 1.08 |
| 8 |  | [BCR Signaling Pathway](http://cgap.nci.nih.gov/Pathways/BioCarta/m_bcrPathway) | [Btk](http://www.ncbi.nlm.nih.gov/entrez/query.fcgi?cmd=search&db=gene&term=Btk) | Bruton agammaglobulinemia tyrosine kinase | 0.106813 | 64.07 | 57.15 | 1.12 |
| 9 |  | [BCR Signaling Pathway](http://cgap.nci.nih.gov/Pathways/BioCarta/m_bcrPathway) | [Mapk14](http://www.ncbi.nlm.nih.gov/entrez/query.fcgi?cmd=search&db=gene&term=Mapk14) | mitogen-activated protein kinase 14 | 0.2051723 | 79.08 | 71.64 | 1.1 |
| 10 |  | [BCR Signaling Pathway](http://cgap.nci.nih.gov/Pathways/BioCarta/m_bcrPathway) | [Nfatc2](http://www.ncbi.nlm.nih.gov/entrez/query.fcgi?cmd=search&db=gene&term=Nfatc2) | nuclear factor of activated T-cells, cytoplasmic, calcineurin-dependent 2 | 0.520464 | 17.55 | 16.65 | 1.05 |
| 1 | m_At1rPathway | [Angiotensin II mediated activation of JNK Pathway via Pyk2 dependent signaling](http://cgap.nci.nih.gov/Pathways/BioCarta/m_At1rPathway) | [Prkcb](http://www.ncbi.nlm.nih.gov/entrez/query.fcgi?cmd=search&db=gene&term=Prkcb) | protein kinase C, beta | 1.3e-06 | 231.8 | 135.08 | 1.72 |
| 2 |  | [Angiotensin II mediated activation of JNK Pathway via Pyk2 dependent signaling](http://cgap.nci.nih.gov/Pathways/BioCarta/m_At1rPathway) | [Jun](http://www.ncbi.nlm.nih.gov/entrez/query.fcgi?cmd=search&db=gene&term=Jun) | Jun oncogene | 7.6e-06 | 325.38 | 205.78 | 1.58 |
| 3 |  | [Angiotensin II mediated activation of JNK Pathway via Pyk2 dependent signaling](http://cgap.nci.nih.gov/Pathways/BioCarta/m_At1rPathway) | [Map2k4](http://www.ncbi.nlm.nih.gov/entrez/query.fcgi?cmd=search&db=gene&term=Map2k4) | mitogen-activated protein kinase kinase 4 | 0.0037785 | 400.03 | 495.55 | 0.81 |
| 4 |  | [Angiotensin II mediated activation of JNK Pathway via Pyk2 dependent signaling](http://cgap.nci.nih.gov/Pathways/BioCarta/m_At1rPathway) | [Mapk8](http://www.ncbi.nlm.nih.gov/entrez/query.fcgi?cmd=search&db=gene&term=Mapk8) | mitogen-activated protein kinase 8 | 0.0235064 | 29.15 | 25.43 | 1.15 |
| 5 |  | [Angiotensin II mediated activation of JNK Pathway via Pyk2 dependent signaling](http://cgap.nci.nih.gov/Pathways/BioCarta/m_At1rPathway) | [Sos1](http://www.ncbi.nlm.nih.gov/entrez/query.fcgi?cmd=search&db=gene&term=Sos1) | son of sevenless homolog 1 (Drosophila) | 0.0657446 | 82.16 | 76.11 | 1.08 |
| 6 |  | [Angiotensin II mediated activation of JNK Pathway via Pyk2 dependent signaling](http://cgap.nci.nih.gov/Pathways/BioCarta/m_At1rPathway) | [Mapk1](http://www.ncbi.nlm.nih.gov/entrez/query.fcgi?cmd=search&db=gene&term=Mapk1) | mitogen-activated protein kinase 1 | 0.0854614 | 196.98 | 247.01 | 0.8 |
| 1 | m_mcmPathway | [CDK Regulation of DNA Replication](http://cgap.nci.nih.gov/Pathways/BioCarta/m_mcmPathway) | [Mcm6](http://www.ncbi.nlm.nih.gov/entrez/query.fcgi?cmd=search&db=gene&term=Mcm6) | minichromosome maintenance deficient 6 (MIS5 homolog, S. pombe) (S. cerevisiae) | 5.1e-06 | 118.66 | 237.09 | 0.5 |
| 2 |  | [CDK Regulation of DNA Replication](http://cgap.nci.nih.gov/Pathways/BioCarta/m_mcmPathway) | [Kitl](http://www.ncbi.nlm.nih.gov/entrez/query.fcgi?cmd=search&db=gene&term=Kitl) | kit ligand | 0.0001164 | 74.16 | 111.24 | 0.67 |
| 3 |  | [CDK Regulation of DNA Replication](http://cgap.nci.nih.gov/Pathways/BioCarta/m_mcmPathway) | [Mcm7](http://www.ncbi.nlm.nih.gov/entrez/query.fcgi?cmd=search&db=gene&term=Mcm7) | minichromosome maintenance deficient 7 (S. cerevisiae) | 0.0003091 | 303.14 | 381.13 | 0.8 |
| 4 |  | [CDK Regulation of DNA Replication](http://cgap.nci.nih.gov/Pathways/BioCarta/m_mcmPathway) | [Mcm4](http://www.ncbi.nlm.nih.gov/entrez/query.fcgi?cmd=search&db=gene&term=Mcm4) | minichromosome maintenance deficient 4 homolog (S. cerevisiae) | 0.0003213 | 171.99 | 238.23 | 0.72 |
| 5 |  | [CDK Regulation of DNA Replication](http://cgap.nci.nih.gov/Pathways/BioCarta/m_mcmPathway) | [Orc2l](http://www.ncbi.nlm.nih.gov/entrez/query.fcgi?cmd=search&db=gene&term=Orc2l) | origin recognition complex, subunit 2-like (S. cerevisiae) | 0.0125974 | 56.16 | 71.46 | 0.79 |
| 6 |  | [CDK Regulation of DNA Replication](http://cgap.nci.nih.gov/Pathways/BioCarta/m_mcmPathway) | [Cdkn1b](http://www.ncbi.nlm.nih.gov/entrez/query.fcgi?cmd=search&db=gene&term=Cdkn1b) | cyclin-dependent kinase inhibitor 1B | 0.0729406 | 41.45 | 55.78 | 0.74 |
| 7 |  | [CDK Regulation of DNA Replication](http://cgap.nci.nih.gov/Pathways/BioCarta/m_mcmPathway) | [Cdc6](http://www.ncbi.nlm.nih.gov/entrez/query.fcgi?cmd=search&db=gene&term=Cdc6) | cell division cycle 6 homolog (S. cerevisiae) | 0.7019116 | 50.08 | 51.77 | 0.97 |
| 1 | m_mPRPathway | [How Progesterone Initiates the Oocyte Maturation](http://cgap.nci.nih.gov/Pathways/BioCarta/m_mPRPathway) | [Cap1](http://www.ncbi.nlm.nih.gov/entrez/query.fcgi?cmd=search&db=gene&term=Cap1) | CAP, adenylate cyclase-associated protein 1 (yeast) | < 1e-07 | 464.38 | 61.37 | 7.57 |
| 2 |  | [How Progesterone Initiates the Oocyte Maturation](http://cgap.nci.nih.gov/Pathways/BioCarta/m_mPRPathway) | [Ccnb1](http://www.ncbi.nlm.nih.gov/entrez/query.fcgi?cmd=search&db=gene&term=Ccnb1) | cyclin B1 | 0.0003148 | 155.23 | 194.83 | 0.8 |
| 3 |  | [How Progesterone Initiates the Oocyte Maturation](http://cgap.nci.nih.gov/Pathways/BioCarta/m_mPRPathway) | [Cdk1](http://www.ncbi.nlm.nih.gov/entrez/query.fcgi?cmd=search&db=gene&term=Cdk1) | cyclin-dependent kinase 1 | 0.0033808 | 246.97 | 282.88 | 0.87 |
| 4 |  | [How Progesterone Initiates the Oocyte Maturation](http://cgap.nci.nih.gov/Pathways/BioCarta/m_mPRPathway) | [Prkacb](http://www.ncbi.nlm.nih.gov/entrez/query.fcgi?cmd=search&db=gene&term=Prkacb) | protein kinase, cAMP dependent, catalytic, beta | 0.073778 | 210.45 | 190.67 | 1.1 |
| 5 |  | [How Progesterone Initiates the Oocyte Maturation](http://cgap.nci.nih.gov/Pathways/BioCarta/m_mPRPathway) | [Mapk1](http://www.ncbi.nlm.nih.gov/entrez/query.fcgi?cmd=search&db=gene&term=Mapk1) | mitogen-activated protein kinase 1 | 0.0854614 | 196.98 | 247.01 | 0.8 |
| 6 |  | [How Progesterone Initiates the Oocyte Maturation](http://cgap.nci.nih.gov/Pathways/BioCarta/m_mPRPathway) | [Cdc25c](http://www.ncbi.nlm.nih.gov/entrez/query.fcgi?cmd=search&db=gene&term=Cdc25c) | cell division cycle 25 homolog C (S. pombe) | 0.3290423 | 55.1 | 58.92 | 0.94 |
| 1 | m_akap95Pathway | [AKAP95 role in mitosis and chromosome dynamics](http://cgap.nci.nih.gov/Pathways/BioCarta/m_akap95Pathway) | [Ncapd2](http://www.ncbi.nlm.nih.gov/entrez/query.fcgi?cmd=search&db=gene&term=Ncapd2) | non-SMC condensin I complex, subunit D2 | 0.0002948 | 260.95 | 330.57 | 0.79 |
| 2 |  | [AKAP95 role in mitosis and chromosome dynamics](http://cgap.nci.nih.gov/Pathways/BioCarta/m_akap95Pathway) | [Ccnb1](http://www.ncbi.nlm.nih.gov/entrez/query.fcgi?cmd=search&db=gene&term=Ccnb1) | cyclin B1 | 0.0003148 | 155.23 | 194.83 | 0.8 |
| 3 |  | [AKAP95 role in mitosis and chromosome dynamics](http://cgap.nci.nih.gov/Pathways/BioCarta/m_akap95Pathway) | [Ddx5](http://www.ncbi.nlm.nih.gov/entrez/query.fcgi?cmd=search&db=gene&term=Ddx5) | DEAD (Asp-Glu-Ala-Asp) box polypeptide 5 | 0.0005481 | 2543.94 | 3186.66 | 0.8 |
| 4 |  | [AKAP95 role in mitosis and chromosome dynamics](http://cgap.nci.nih.gov/Pathways/BioCarta/m_akap95Pathway) | [Cdk1](http://www.ncbi.nlm.nih.gov/entrez/query.fcgi?cmd=search&db=gene&term=Cdk1) | cyclin-dependent kinase 1 | 0.0033808 | 246.97 | 282.88 | 0.87 |
| 5 |  | [AKAP95 role in mitosis and chromosome dynamics](http://cgap.nci.nih.gov/Pathways/BioCarta/m_akap95Pathway) | [Prkacb](http://www.ncbi.nlm.nih.gov/entrez/query.fcgi?cmd=search&db=gene&term=Prkacb) | protein kinase, cAMP dependent, catalytic, beta | 0.073778 | 210.45 | 190.67 | 1.1 |
| 1 | m_il6Pathway | [IL 6 signaling pathway](http://cgap.nci.nih.gov/Pathways/BioCarta/m_il6Pathway) | [Fos](http://www.ncbi.nlm.nih.gov/entrez/query.fcgi?cmd=search&db=gene&term=Fos) | FBJ osteosarcoma oncogene | < 1e-07 | 2482.37 | 294.09 | 8.44 |
| 2 |  | [IL 6 signaling pathway](http://cgap.nci.nih.gov/Pathways/BioCarta/m_il6Pathway) | [Jun](http://www.ncbi.nlm.nih.gov/entrez/query.fcgi?cmd=search&db=gene&term=Jun) | Jun oncogene | 7.6e-06 | 325.38 | 205.78 | 1.58 |
| 3 |  | [IL 6 signaling pathway](http://cgap.nci.nih.gov/Pathways/BioCarta/m_il6Pathway) | [Ptpn11](http://www.ncbi.nlm.nih.gov/entrez/query.fcgi?cmd=search&db=gene&term=Ptpn11) | protein tyrosine phosphatase, non-receptor type 11 | 0.0002035 | 49.82 | 67.58 | 0.74 |
| 4 |  | [IL 6 signaling pathway](http://cgap.nci.nih.gov/Pathways/BioCarta/m_il6Pathway) | [Csnk2a2](http://www.ncbi.nlm.nih.gov/entrez/query.fcgi?cmd=search&db=gene&term=Csnk2a2) | casein kinase 2, alpha prime polypeptide | 0.0020382 | 2035.41 | 1719.9 | 1.18 |
| 5 |  | [IL 6 signaling pathway](http://cgap.nci.nih.gov/Pathways/BioCarta/m_il6Pathway) | [Csnk2a1](http://www.ncbi.nlm.nih.gov/entrez/query.fcgi?cmd=search&db=gene&term=Csnk2a1) | casein kinase 2, alpha 1 polypeptide | 0.0029286 | 200.24 | 272.9 | 0.73 |
| 6 |  | [IL 6 signaling pathway](http://cgap.nci.nih.gov/Pathways/BioCarta/m_il6Pathway) | [Sos1](http://www.ncbi.nlm.nih.gov/entrez/query.fcgi?cmd=search&db=gene&term=Sos1) | son of sevenless homolog 1 (Drosophila) | 0.0657446 | 82.16 | 76.11 | 1.08 |
| 7 |  | [IL 6 signaling pathway](http://cgap.nci.nih.gov/Pathways/BioCarta/m_il6Pathway) | [Il6st](http://www.ncbi.nlm.nih.gov/entrez/query.fcgi?cmd=search&db=gene&term=Il6st) | interleukin 6 signal transducer | 0.2053222 | 96.65 | 107.3 | 0.9 |
| 8 |  | [IL 6 signaling pathway](http://cgap.nci.nih.gov/Pathways/BioCarta/m_il6Pathway) | [Jak3](http://www.ncbi.nlm.nih.gov/entrez/query.fcgi?cmd=search&db=gene&term=Jak3) | Janus kinase 3 | 0.2367555 | 10.9 | 10.31 | 1.06 |
| 9 |  | [IL 6 signaling pathway](http://cgap.nci.nih.gov/Pathways/BioCarta/m_il6Pathway) | [Jak1](http://www.ncbi.nlm.nih.gov/entrez/query.fcgi?cmd=search&db=gene&term=Jak1) | Janus kinase 1 | 0.5074408 | 151.14 | 156.56 | 0.97 |
| 10 |  | [IL 6 signaling pathway](http://cgap.nci.nih.gov/Pathways/BioCarta/m_il6Pathway) | [Cebpb](http://www.ncbi.nlm.nih.gov/entrez/query.fcgi?cmd=search&db=gene&term=Cebpb) | CCAAT/enhancer binding protein (C/EBP), beta | 0.6008885 | 2150.51 | 2197.52 | 0.98 |
| 1 | m_Lis1Pathway | [Lissencephaly gene (LIS1) in neuronal migration and development](http://cgap.nci.nih.gov/Pathways/BioCarta/m_Lis1Pathway) | [Ndel1](http://www.ncbi.nlm.nih.gov/entrez/query.fcgi?cmd=search&db=gene&term=Ndel1) | nuclear distribution gene E-like homolog 1 (A. nidulans) | 1.93e-05 | 302.18 | 480.81 | 0.63 |
| 2 |  | [Lissencephaly gene (LIS1) in neuronal migration and development](http://cgap.nci.nih.gov/Pathways/BioCarta/m_Lis1Pathway) | [Cdk5r1](http://www.ncbi.nlm.nih.gov/entrez/query.fcgi?cmd=search&db=gene&term=Cdk5r1) | cyclin-dependent kinase 5, regulatory subunit 1 (p35) | 0.0002963 | 54 | 40.71 | 1.33 |
| 3 |  | [Lissencephaly gene (LIS1) in neuronal migration and development](http://cgap.nci.nih.gov/Pathways/BioCarta/m_Lis1Pathway) | [Csnk2a2](http://www.ncbi.nlm.nih.gov/entrez/query.fcgi?cmd=search&db=gene&term=Csnk2a2) | casein kinase 2, alpha prime polypeptide | 0.0020382 | 2035.41 | 1719.9 | 1.18 |
| 4 |  | [Lissencephaly gene (LIS1) in neuronal migration and development](http://cgap.nci.nih.gov/Pathways/BioCarta/m_Lis1Pathway) | [Csnk2a1](http://www.ncbi.nlm.nih.gov/entrez/query.fcgi?cmd=search&db=gene&term=Csnk2a1) | casein kinase 2, alpha 1 polypeptide | 0.0029286 | 200.24 | 272.9 | 0.73 |
| 5 |  | [Lissencephaly gene (LIS1) in neuronal migration and development](http://cgap.nci.nih.gov/Pathways/BioCarta/m_Lis1Pathway) | [Clip1](http://www.ncbi.nlm.nih.gov/entrez/query.fcgi?cmd=search&db=gene&term=Clip1) | CAP-GLY domain containing linker protein 1 | 0.0035212 | 125.72 | 182.87 | 0.69 |
| 6 |  | [Lissencephaly gene (LIS1) in neuronal migration and development](http://cgap.nci.nih.gov/Pathways/BioCarta/m_Lis1Pathway) | [Pafah1b1](http://www.ncbi.nlm.nih.gov/entrez/query.fcgi?cmd=search&db=gene&term=Pafah1b1) | platelet-activating factor acetylhydrolase, isoform 1b, subunit 1 | 0.0289507 | 101.43 | 119.8 | 0.85 |
| 7 |  | [Lissencephaly gene (LIS1) in neuronal migration and development](http://cgap.nci.nih.gov/Pathways/BioCarta/m_Lis1Pathway) | [Dcx](http://www.ncbi.nlm.nih.gov/entrez/query.fcgi?cmd=search&db=gene&term=Dcx) | doublecortin | 0.1029627 | 11.73 | 10.53 | 1.11 |
| 1 | m_il2Pathway | [IL 2 signaling pathway](http://cgap.nci.nih.gov/Pathways/BioCarta/m_il2Pathway) | [Fos](http://www.ncbi.nlm.nih.gov/entrez/query.fcgi?cmd=search&db=gene&term=Fos) | FBJ osteosarcoma oncogene | < 1e-07 | 2482.37 | 294.09 | 8.44 |
| 2 |  | [IL 2 signaling pathway](http://cgap.nci.nih.gov/Pathways/BioCarta/m_il2Pathway) | [Jun](http://www.ncbi.nlm.nih.gov/entrez/query.fcgi?cmd=search&db=gene&term=Jun) | Jun oncogene | 7.6e-06 | 325.38 | 205.78 | 1.58 |
| 3 |  | [IL 2 signaling pathway](http://cgap.nci.nih.gov/Pathways/BioCarta/m_il2Pathway) | [Il2rg](http://www.ncbi.nlm.nih.gov/entrez/query.fcgi?cmd=search&db=gene&term=Il2rg) | interleukin 2 receptor, gamma chain | 0.0001378 | 193.73 | 271.54 | 0.71 |
| 4 |  | [IL 2 signaling pathway](http://cgap.nci.nih.gov/Pathways/BioCarta/m_il2Pathway) | [Csnk2a2](http://www.ncbi.nlm.nih.gov/entrez/query.fcgi?cmd=search&db=gene&term=Csnk2a2) | casein kinase 2, alpha prime polypeptide | 0.0020382 | 2035.41 | 1719.9 | 1.18 |
| 5 |  | [IL 2 signaling pathway](http://cgap.nci.nih.gov/Pathways/BioCarta/m_il2Pathway) | [Csnk2a1](http://www.ncbi.nlm.nih.gov/entrez/query.fcgi?cmd=search&db=gene&term=Csnk2a1) | casein kinase 2, alpha 1 polypeptide | 0.0029286 | 200.24 | 272.9 | 0.73 |
| 6 |  | [IL 2 signaling pathway](http://cgap.nci.nih.gov/Pathways/BioCarta/m_il2Pathway) | [Lck](http://www.ncbi.nlm.nih.gov/entrez/query.fcgi?cmd=search&db=gene&term=Lck) | lymphocyte protein tyrosine kinase | 0.0037891 | 81.12 | 69.9 | 1.16 |
| 7 |  | [IL 2 signaling pathway](http://cgap.nci.nih.gov/Pathways/BioCarta/m_il2Pathway) | [Mapk8](http://www.ncbi.nlm.nih.gov/entrez/query.fcgi?cmd=search&db=gene&term=Mapk8) | mitogen-activated protein kinase 8 | 0.0235064 | 29.15 | 25.43 | 1.15 |
| 8 |  | [IL 2 signaling pathway](http://cgap.nci.nih.gov/Pathways/BioCarta/m_il2Pathway) | [Sos1](http://www.ncbi.nlm.nih.gov/entrez/query.fcgi?cmd=search&db=gene&term=Sos1) | son of sevenless homolog 1 (Drosophila) | 0.0657446 | 82.16 | 76.11 | 1.08 |
| 9 |  | [IL 2 signaling pathway](http://cgap.nci.nih.gov/Pathways/BioCarta/m_il2Pathway) | [Jak3](http://www.ncbi.nlm.nih.gov/entrez/query.fcgi?cmd=search&db=gene&term=Jak3) | Janus kinase 3 | 0.2367555 | 10.9 | 10.31 | 1.06 |
| 10 |  | [IL 2 signaling pathway](http://cgap.nci.nih.gov/Pathways/BioCarta/m_il2Pathway) | [Stat5a](http://www.ncbi.nlm.nih.gov/entrez/query.fcgi?cmd=search&db=gene&term=Stat5a) | signal transducer and activator of transcription 5A | 0.4703697 | 76.75 | 73.88 | 1.04 |
| 11 |  | [IL 2 signaling pathway](http://cgap.nci.nih.gov/Pathways/BioCarta/m_il2Pathway) | [Jak1](http://www.ncbi.nlm.nih.gov/entrez/query.fcgi?cmd=search&db=gene&term=Jak1) | Janus kinase 1 | 0.5074408 | 151.14 | 156.56 | 0.97 |
| 12 |  | [IL 2 signaling pathway](http://cgap.nci.nih.gov/Pathways/BioCarta/m_il2Pathway) | [Il2rb](http://www.ncbi.nlm.nih.gov/entrez/query.fcgi?cmd=search&db=gene&term=Il2rb) | interleukin 2 receptor, beta chain | 0.9423035 | 63.16 | 63.56 | 0.99 |
| 1 | m_rac1Pathway | [Rac 1 cell motility signaling pathway](http://cgap.nci.nih.gov/Pathways/BioCarta/m_rac1Pathway) | [Pld1](http://www.ncbi.nlm.nih.gov/entrez/query.fcgi?cmd=search&db=gene&term=Pld1) | phospholipase D1 | 4.16e-05 | 110.04 | 160.06 | 0.69 |
| 2 |  | [Rac 1 cell motility signaling pathway](http://cgap.nci.nih.gov/Pathways/BioCarta/m_rac1Pathway) | [Cdk5r1](http://www.ncbi.nlm.nih.gov/entrez/query.fcgi?cmd=search&db=gene&term=Cdk5r1) | cyclin-dependent kinase 5, regulatory subunit 1 (p35) | 0.0002963 | 54 | 40.71 | 1.33 |
| 3 |  | [Rac 1 cell motility signaling pathway](http://cgap.nci.nih.gov/Pathways/BioCarta/m_rac1Pathway) | [Pik3r1](http://www.ncbi.nlm.nih.gov/entrez/query.fcgi?cmd=search&db=gene&term=Pik3r1) | phosphatidylinositol 3-kinase, regulatory subunit, polypeptide 1 (p85 alpha) | 0.0011172 | 134.94 | 110.05 | 1.23 |
| 4 |  | [Rac 1 cell motility signaling pathway](http://cgap.nci.nih.gov/Pathways/BioCarta/m_rac1Pathway) | [Vav1](http://www.ncbi.nlm.nih.gov/entrez/query.fcgi?cmd=search&db=gene&term=Vav1) | vav 1 oncogene | 0.005748 | 23.69 | 19.16 | 1.24 |
| 5 |  | [Rac 1 cell motility signaling pathway](http://cgap.nci.nih.gov/Pathways/BioCarta/m_rac1Pathway) | [Rps6kb1](http://www.ncbi.nlm.nih.gov/entrez/query.fcgi?cmd=search&db=gene&term=Rps6kb1) | ribosomal protein S6 kinase, polypeptide 1 | 0.028282 | 236.59 | 277.39 | 0.85 |
| 6 |  | [Rac 1 cell motility signaling pathway](http://cgap.nci.nih.gov/Pathways/BioCarta/m_rac1Pathway) | [Pik3cg](http://www.ncbi.nlm.nih.gov/entrez/query.fcgi?cmd=search&db=gene&term=Pik3cg) | phosphoinositide-3-kinase, catalytic, gamma polypeptide | 0.0302332 | 18.24 | 14.76 | 1.24 |
| 7 |  | [Rac 1 cell motility signaling pathway](http://cgap.nci.nih.gov/Pathways/BioCarta/m_rac1Pathway) | [Trio](http://www.ncbi.nlm.nih.gov/entrez/query.fcgi?cmd=search&db=gene&term=Trio) | triple functional domain (PTPRF interacting) | 0.0373332 | 141.87 | 126.38 | 1.12 |
| 8 |  | [Rac 1 cell motility signaling pathway](http://cgap.nci.nih.gov/Pathways/BioCarta/m_rac1Pathway) | [Chn1](http://www.ncbi.nlm.nih.gov/entrez/query.fcgi?cmd=search&db=gene&term=Chn1) | chimerin (chimaerin) 1 | 0.0765822 | 10.63 | 8.63 | 1.23 |
| 1 | m_rasPathway | [Ras Signaling Pathway](http://cgap.nci.nih.gov/Pathways/BioCarta/m_rasPathway) | [Pld1](http://www.ncbi.nlm.nih.gov/entrez/query.fcgi?cmd=search&db=gene&term=Pld1) | phospholipase D1 | 4.16e-05 | 110.04 | 160.06 | 0.69 |
| 2 |  | [Ras Signaling Pathway](http://cgap.nci.nih.gov/Pathways/BioCarta/m_rasPathway) | [Pik3r1](http://www.ncbi.nlm.nih.gov/entrez/query.fcgi?cmd=search&db=gene&term=Pik3r1) | phosphatidylinositol 3-kinase, regulatory subunit, polypeptide 1 (p85 alpha) | 0.0011172 | 134.94 | 110.05 | 1.23 |
| 3 |  | [Ras Signaling Pathway](http://cgap.nci.nih.gov/Pathways/BioCarta/m_rasPathway) | [Nfkb1](http://www.ncbi.nlm.nih.gov/entrez/query.fcgi?cmd=search&db=gene&term=Nfkb1) | nuclear factor of kappa light polypeptide gene enhancer in B-cells 1, p105 | 0.0055039 | 708.56 | 804.29 | 0.88 |
| 4 |  | [Ras Signaling Pathway](http://cgap.nci.nih.gov/Pathways/BioCarta/m_rasPathway) | [Pik3cg](http://www.ncbi.nlm.nih.gov/entrez/query.fcgi?cmd=search&db=gene&term=Pik3cg) | phosphoinositide-3-kinase, catalytic, gamma polypeptide | 0.0302332 | 18.24 | 14.76 | 1.24 |
| 5 |  | [Ras Signaling Pathway](http://cgap.nci.nih.gov/Pathways/BioCarta/m_rasPathway) | [Rala](http://www.ncbi.nlm.nih.gov/entrez/query.fcgi?cmd=search&db=gene&term=Rala) | v-ral simian leukemia viral oncogene homolog A (ras related) | 0.0671018 | 20.18 | 23.67 | 0.85 |
| 1 | m_erythPathway | [Erythrocyte Differentiation Pathway](http://cgap.nci.nih.gov/Pathways/BioCarta/m_erythPathway) | [Kitl](http://www.ncbi.nlm.nih.gov/entrez/query.fcgi?cmd=search&db=gene&term=Kitl) | kit ligand | 0.0001164 | 74.16 | 111.24 | 0.67 |
| 2 |  | [Erythrocyte Differentiation Pathway](http://cgap.nci.nih.gov/Pathways/BioCarta/m_erythPathway) | [Tgfb3](http://www.ncbi.nlm.nih.gov/entrez/query.fcgi?cmd=search&db=gene&term=Tgfb3) | transforming growth factor, beta 3 | 0.0032917 | 47.06 | 35.31 | 1.33 |
| 3 |  | [Erythrocyte Differentiation Pathway](http://cgap.nci.nih.gov/Pathways/BioCarta/m_erythPathway) | [Tgfb2](http://www.ncbi.nlm.nih.gov/entrez/query.fcgi?cmd=search&db=gene&term=Tgfb2) | transforming growth factor, beta 2 | 0.0042145 | 10.99 | 8.4 | 1.31 |
| 4 |  | [Erythrocyte Differentiation Pathway](http://cgap.nci.nih.gov/Pathways/BioCarta/m_erythPathway) | [Il1a](http://www.ncbi.nlm.nih.gov/entrez/query.fcgi?cmd=search&db=gene&term=Il1a) | interleukin 1 alpha | 0.0081701 | 66.39 | 101.68 | 0.65 |
| 5 |  | [Erythrocyte Differentiation Pathway](http://cgap.nci.nih.gov/Pathways/BioCarta/m_erythPathway) | [Csf2](http://www.ncbi.nlm.nih.gov/entrez/query.fcgi?cmd=search&db=gene&term=Csf2) | colony stimulating factor 2 (granulocyte-macrophage) | 0.0460919 | 17.74 | 22.15 | 0.8 |
| 1 | m_EfpPathway | [Estrogen-responsive protein Efp controls cell cycle and breast tumors growth](http://cgap.nci.nih.gov/Pathways/BioCarta/m_EfpPathway) | [Ccnb1](http://www.ncbi.nlm.nih.gov/entrez/query.fcgi?cmd=search&db=gene&term=Ccnb1) | cyclin B1 | 0.0003148 | 155.23 | 194.83 | 0.8 |
| 2 |  | [Estrogen-responsive protein Efp controls cell cycle and breast tumors growth](http://cgap.nci.nih.gov/Pathways/BioCarta/m_EfpPathway) | [Ccnb2](http://www.ncbi.nlm.nih.gov/entrez/query.fcgi?cmd=search&db=gene&term=Ccnb2) | cyclin B2 | 0.0016228 | 276.5 | 337.32 | 0.82 |
| 3 |  | [Estrogen-responsive protein Efp controls cell cycle and breast tumors growth](http://cgap.nci.nih.gov/Pathways/BioCarta/m_EfpPathway) | [Cdk1](http://www.ncbi.nlm.nih.gov/entrez/query.fcgi?cmd=search&db=gene&term=Cdk1) | cyclin-dependent kinase 1 | 0.0033808 | 246.97 | 282.88 | 0.87 |
| 4 |  | [Estrogen-responsive protein Efp controls cell cycle and breast tumors growth](http://cgap.nci.nih.gov/Pathways/BioCarta/m_EfpPathway) | [Trp53](http://www.ncbi.nlm.nih.gov/entrez/query.fcgi?cmd=search&db=gene&term=Trp53) | transformation related protein 53 | 0.0115487 | 12.39 | 14.37 | 0.86 |
| 5 |  | [Estrogen-responsive protein Efp controls cell cycle and breast tumors growth](http://cgap.nci.nih.gov/Pathways/BioCarta/m_EfpPathway) | [Cdk6](http://www.ncbi.nlm.nih.gov/entrez/query.fcgi?cmd=search&db=gene&term=Cdk6) | cyclin-dependent kinase 6 | 0.0652736 | 19.26 | 21.96 | 0.88 |
| 1 | m_g1Pathway | [Cell Cycle: G1/S Check Point](http://cgap.nci.nih.gov/Pathways/BioCarta/m_g1Pathway) | [Cdkn2b](http://www.ncbi.nlm.nih.gov/entrez/query.fcgi?cmd=search&db=gene&term=Cdkn2b) | cyclin-dependent kinase inhibitor 2B (p15, inhibits CDK4) | 4e-07 | 521.99 | 288.1 | 1.81 |
| 2 |  | [Cell Cycle: G1/S Check Point](http://cgap.nci.nih.gov/Pathways/BioCarta/m_g1Pathway) | [Smad4](http://www.ncbi.nlm.nih.gov/entrez/query.fcgi?cmd=search&db=gene&term=Smad4) | MAD homolog 4 (Drosophila) | 0.0001341 | 463.34 | 636.93 | 0.73 |
| 3 |  | [Cell Cycle: G1/S Check Point](http://cgap.nci.nih.gov/Pathways/BioCarta/m_g1Pathway) | [Tgfb3](http://www.ncbi.nlm.nih.gov/entrez/query.fcgi?cmd=search&db=gene&term=Tgfb3) | transforming growth factor, beta 3 | 0.0032917 | 47.06 | 35.31 | 1.33 |
| 4 |  | [Cell Cycle: G1/S Check Point](http://cgap.nci.nih.gov/Pathways/BioCarta/m_g1Pathway) | [Cdk1](http://www.ncbi.nlm.nih.gov/entrez/query.fcgi?cmd=search&db=gene&term=Cdk1) | cyclin-dependent kinase 1 | 0.0033808 | 246.97 | 282.88 | 0.87 |
| 5 |  | [Cell Cycle: G1/S Check Point](http://cgap.nci.nih.gov/Pathways/BioCarta/m_g1Pathway) | [Tgfb2](http://www.ncbi.nlm.nih.gov/entrez/query.fcgi?cmd=search&db=gene&term=Tgfb2) | transforming growth factor, beta 2 | 0.0042145 | 10.99 | 8.4 | 1.31 |
| 6 |  | [Cell Cycle: G1/S Check Point](http://cgap.nci.nih.gov/Pathways/BioCarta/m_g1Pathway) | [Trp53](http://www.ncbi.nlm.nih.gov/entrez/query.fcgi?cmd=search&db=gene&term=Trp53) | transformation related protein 53 | 0.0115487 | 12.39 | 14.37 | 0.86 |
| 7 |  | [Cell Cycle: G1/S Check Point](http://cgap.nci.nih.gov/Pathways/BioCarta/m_g1Pathway) | [Dhfr](http://www.ncbi.nlm.nih.gov/entrez/query.fcgi?cmd=search&db=gene&term=Dhfr) | dihydrofolate reductase | 0.0124596 | 115.43 | 129.8 | 0.89 |
| 8 |  | [Cell Cycle: G1/S Check Point](http://cgap.nci.nih.gov/Pathways/BioCarta/m_g1Pathway) | [Ccnd1](http://www.ncbi.nlm.nih.gov/entrez/query.fcgi?cmd=search&db=gene&term=Ccnd1) | cyclin D1 | 0.047587 | 420.26 | 465.33 | 0.9 |
| 9 |  | [Cell Cycle: G1/S Check Point](http://cgap.nci.nih.gov/Pathways/BioCarta/m_g1Pathway) | [Cdkn2a](http://www.ncbi.nlm.nih.gov/entrez/query.fcgi?cmd=search&db=gene&term=Cdkn2a) | cyclin-dependent kinase inhibitor 2A | 0.0590612 | 14.02 | 12.23 | 1.15 |
| 10 |  | [Cell Cycle: G1/S Check Point](http://cgap.nci.nih.gov/Pathways/BioCarta/m_g1Pathway) | [Cdk6](http://www.ncbi.nlm.nih.gov/entrez/query.fcgi?cmd=search&db=gene&term=Cdk6) | cyclin-dependent kinase 6 | 0.0652736 | 19.26 | 21.96 | 0.88 |
| 11 |  | [Cell Cycle: G1/S Check Point](http://cgap.nci.nih.gov/Pathways/BioCarta/m_g1Pathway) | [Cdkn1b](http://www.ncbi.nlm.nih.gov/entrez/query.fcgi?cmd=search&db=gene&term=Cdkn1b) | cyclin-dependent kinase inhibitor 1B | 0.0729406 | 41.45 | 55.78 | 0.74 |
| 12 |  | [Cell Cycle: G1/S Check Point](http://cgap.nci.nih.gov/Pathways/BioCarta/m_g1Pathway) | [Skp2](http://www.ncbi.nlm.nih.gov/entrez/query.fcgi?cmd=search&db=gene&term=Skp2) | S-phase kinase-associated protein 2 (p45) | 0.0745415 | 100.18 | 112.57 | 0.89 |
| 13 |  | [Cell Cycle: G1/S Check Point](http://cgap.nci.nih.gov/Pathways/BioCarta/m_g1Pathway) | [Smad3](http://www.ncbi.nlm.nih.gov/entrez/query.fcgi?cmd=search&db=gene&term=Smad3) | MAD homolog 3 (Drosophila) | 0.1793711 | 36.92 | 43.06 | 0.86 |
| 1 | m_il3Pathway | [IL 3 signaling pathway](http://cgap.nci.nih.gov/Pathways/BioCarta/m_il3Pathway) | [Fos](http://www.ncbi.nlm.nih.gov/entrez/query.fcgi?cmd=search&db=gene&term=Fos) | FBJ osteosarcoma oncogene | < 1e-07 | 2482.37 | 294.09 | 8.44 |
| 2 |  | [IL 3 signaling pathway](http://cgap.nci.nih.gov/Pathways/BioCarta/m_il3Pathway) | [Sos1](http://www.ncbi.nlm.nih.gov/entrez/query.fcgi?cmd=search&db=gene&term=Sos1) | son of sevenless homolog 1 (Drosophila) | 0.0657446 | 82.16 | 76.11 | 1.08 |
| 3 |  | [IL 3 signaling pathway](http://cgap.nci.nih.gov/Pathways/BioCarta/m_il3Pathway) | [Il3ra](http://www.ncbi.nlm.nih.gov/entrez/query.fcgi?cmd=search&db=gene&term=Il3ra) | interleukin 3 receptor, alpha chain | 0.0808439 | 38.53 | 34.37 | 1.12 |
| 4 |  | [IL 3 signaling pathway](http://cgap.nci.nih.gov/Pathways/BioCarta/m_il3Pathway) | [Csf2rb](http://www.ncbi.nlm.nih.gov/entrez/query.fcgi?cmd=search&db=gene&term=Csf2rb) | colony stimulating factor 2 receptor, beta, low-affinity (granulocyte-macrophage) | 0.2115411 | 39.02 | 42.88 | 0.91 |
| 5 |  | [IL 3 signaling pathway](http://cgap.nci.nih.gov/Pathways/BioCarta/m_il3Pathway) | [Stat5a](http://www.ncbi.nlm.nih.gov/entrez/query.fcgi?cmd=search&db=gene&term=Stat5a) | signal transducer and activator of transcription 5A | 0.4703697 | 76.75 | 73.88 | 1.04 |
| 1 | m_RacCycDPathway | [Influence of Ras and Rho proteins on G1 to S Transition](http://cgap.nci.nih.gov/Pathways/BioCarta/m_RacCycDPathway) | [Nfkbia](http://www.ncbi.nlm.nih.gov/entrez/query.fcgi?cmd=search&db=gene&term=Nfkbia) | nuclear factor of kappa light polypeptide gene enhancer in B-cells inhibitor, alpha | 4.2e-06 | 1283.3 | 852.99 | 1.5 |
| 2 |  | [Influence of Ras and Rho proteins on G1 to S Transition](http://cgap.nci.nih.gov/Pathways/BioCarta/m_RacCycDPathway) | [Pik3r1](http://www.ncbi.nlm.nih.gov/entrez/query.fcgi?cmd=search&db=gene&term=Pik3r1) | phosphatidylinositol 3-kinase, regulatory subunit, polypeptide 1 (p85 alpha) | 0.0011172 | 134.94 | 110.05 | 1.23 |
| 3 |  | [Influence of Ras and Rho proteins on G1 to S Transition](http://cgap.nci.nih.gov/Pathways/BioCarta/m_RacCycDPathway) | [Nfkb1](http://www.ncbi.nlm.nih.gov/entrez/query.fcgi?cmd=search&db=gene&term=Nfkb1) | nuclear factor of kappa light polypeptide gene enhancer in B-cells 1, p105 | 0.0055039 | 708.56 | 804.29 | 0.88 |
| 4 |  | [Influence of Ras and Rho proteins on G1 to S Transition](http://cgap.nci.nih.gov/Pathways/BioCarta/m_RacCycDPathway) | [Ccnd1](http://www.ncbi.nlm.nih.gov/entrez/query.fcgi?cmd=search&db=gene&term=Ccnd1) | cyclin D1 | 0.047587 | 420.26 | 465.33 | 0.9 |
| 5 |  | [Influence of Ras and Rho proteins on G1 to S Transition](http://cgap.nci.nih.gov/Pathways/BioCarta/m_RacCycDPathway) | [Cdk6](http://www.ncbi.nlm.nih.gov/entrez/query.fcgi?cmd=search&db=gene&term=Cdk6) | cyclin-dependent kinase 6 | 0.0652736 | 19.26 | 21.96 | 0.88 |
| 6 |  | [Influence of Ras and Rho proteins on G1 to S Transition](http://cgap.nci.nih.gov/Pathways/BioCarta/m_RacCycDPathway) | [Cdkn1b](http://www.ncbi.nlm.nih.gov/entrez/query.fcgi?cmd=search&db=gene&term=Cdkn1b) | cyclin-dependent kinase inhibitor 1B | 0.0729406 | 41.45 | 55.78 | 0.74 |
| 7 |  | [Influence of Ras and Rho proteins on G1 to S Transition](http://cgap.nci.nih.gov/Pathways/BioCarta/m_RacCycDPathway) | [Mapk1](http://www.ncbi.nlm.nih.gov/entrez/query.fcgi?cmd=search&db=gene&term=Mapk1) | mitogen-activated protein kinase 1 | 0.0854614 | 196.98 | 247.01 | 0.8 |
| 1 | m_th1th2Pathway | [Th1/Th2 Differentiation](http://cgap.nci.nih.gov/Pathways/BioCarta/m_th1th2Pathway) | [Il12rb2](http://www.ncbi.nlm.nih.gov/entrez/query.fcgi?cmd=search&db=gene&term=Il12rb2) | interleukin 12 receptor, beta 2 | 4.43e-05 | 69.29 | 94.02 | 0.74 |
| 2 |  | [Th1/Th2 Differentiation](http://cgap.nci.nih.gov/Pathways/BioCarta/m_th1th2Pathway) | [Il12b](http://www.ncbi.nlm.nih.gov/entrez/query.fcgi?cmd=search&db=gene&term=Il12b) | interleukin 12b | 0.0020464 | 10.47 | 12.36 | 0.85 |
| 3 |  | [Th1/Th2 Differentiation](http://cgap.nci.nih.gov/Pathways/BioCarta/m_th1th2Pathway) | [Il12rb1](http://www.ncbi.nlm.nih.gov/entrez/query.fcgi?cmd=search&db=gene&term=Il12rb1) | interleukin 12 receptor, beta 1 | 0.0036856 | 24.28 | 21.15 | 1.15 |
| 4 |  | [Th1/Th2 Differentiation](http://cgap.nci.nih.gov/Pathways/BioCarta/m_th1th2Pathway) | [Pvrl1](http://www.ncbi.nlm.nih.gov/entrez/query.fcgi?cmd=search&db=gene&term=Pvrl1) | poliovirus receptor-related 1 | 0.004869 | 47.63 | 58.7 | 0.81 |
| 5 |  | [Th1/Th2 Differentiation](http://cgap.nci.nih.gov/Pathways/BioCarta/m_th1th2Pathway) | [Ifngr2](http://www.ncbi.nlm.nih.gov/entrez/query.fcgi?cmd=search&db=gene&term=Ifngr2) | interferon gamma receptor 2 | 0.0203541 | 160.62 | 144.05 | 1.12 |
| 6 |  | [Th1/Th2 Differentiation](http://cgap.nci.nih.gov/Pathways/BioCarta/m_th1th2Pathway) | [Il18r1](http://www.ncbi.nlm.nih.gov/entrez/query.fcgi?cmd=search&db=gene&term=Il18r1) | interleukin 18 receptor 1 | 0.0728961 | 36.12 | 33.43 | 1.08 |
| 7 |  | [Th1/Th2 Differentiation](http://cgap.nci.nih.gov/Pathways/BioCarta/m_th1th2Pathway) | [Cd40lg](http://www.ncbi.nlm.nih.gov/entrez/query.fcgi?cmd=search&db=gene&term=Cd40lg) | CD40 ligand | 0.1265964 | 34.93 | 31.48 | 1.11 |
| 8 |  | [Th1/Th2 Differentiation](http://cgap.nci.nih.gov/Pathways/BioCarta/m_th1th2Pathway) | [Il4](http://www.ncbi.nlm.nih.gov/entrez/query.fcgi?cmd=search&db=gene&term=Il4) | interleukin 4 | 0.1464543 | 12.73 | 11.13 | 1.14 |
| 1 | m_GATA3pathway | [GATA3 participate in activating the Th2 cytokine genes expression](http://cgap.nci.nih.gov/Pathways/BioCarta/m_GATA3pathway) | [Prkacb](http://www.ncbi.nlm.nih.gov/entrez/query.fcgi?cmd=search&db=gene&term=Prkacb) | protein kinase, cAMP dependent, catalytic, beta | 0.073778 | 210.45 | 190.67 | 1.1 |
| 2 |  | [GATA3 participate in activating the Th2 cytokine genes expression](http://cgap.nci.nih.gov/Pathways/BioCarta/m_GATA3pathway) | [Il4](http://www.ncbi.nlm.nih.gov/entrez/query.fcgi?cmd=search&db=gene&term=Il4) | interleukin 4 | 0.1464543 | 12.73 | 11.13 | 1.14 |
| 3 |  | [GATA3 participate in activating the Th2 cytokine genes expression](http://cgap.nci.nih.gov/Pathways/BioCarta/m_GATA3pathway) | [Mapk14](http://www.ncbi.nlm.nih.gov/entrez/query.fcgi?cmd=search&db=gene&term=Mapk14) | mitogen-activated protein kinase 14 | 0.2051723 | 79.08 | 71.64 | 1.1 |
| 4 |  | [GATA3 participate in activating the Th2 cytokine genes expression](http://cgap.nci.nih.gov/Pathways/BioCarta/m_GATA3pathway) | [Gata3](http://www.ncbi.nlm.nih.gov/entrez/query.fcgi?cmd=search&db=gene&term=Gata3) | GATA binding protein 3 | 0.4859883 | 1814.84 | 1862.92 | 0.97 |
| 5 |  | [GATA3 participate in activating the Th2 cytokine genes expression](http://cgap.nci.nih.gov/Pathways/BioCarta/m_GATA3pathway) | [Nfatc2](http://www.ncbi.nlm.nih.gov/entrez/query.fcgi?cmd=search&db=gene&term=Nfatc2) | nuclear factor of activated T-cells, cytoplasmic, calcineurin-dependent 2 | 0.520464 | 17.55 | 16.65 | 1.05 |

Class 1: *Tcl1-/-*; Class 2: WT.
