# Supplementary material for: T Cell Leukemia/Lymphoma 1A is essential for mouse epidermal keratinocytes proliferation promoted by insulin-like growth factor 1
Source: PLoS One. 2018 Oct 4;13(10):e0204775. doi: 10.1371/journal.pone.0204775 (PMC6171881; doi:10.1371/journal.pone.0204775)
Supplement: S11 Table — 44 out of 151 investigated gene sets passed the 0.05 significance threshold. LS/KS permutation test found 34 significant gene sets. Efron-Tibshirani's maxmean test found 22 significant gene sets (under 200 permutations). Class 1: K14-TCL1; Class 2: WT. (DOCX) [file pone.0204775.s011.docx]

|  | **Biocarta Pathway** | **Pathway description** | **Gene symbol** | **Description** | **Parametric p-value** | **Geom mean of intensities in class 1** | **Geom mean of intensities in class 2** | **Fold-change** |
| --- | --- | --- | --- | --- | --- | --- | --- | --- |
| 1 | m_tcrPathway | [T Cell Receptor Signaling Pathway](http://cgap.nci.nih.gov/Pathways/BioCarta/m_tcrPathway) | [Fos](http://www.ncbi.nlm.nih.gov/entrez/query.fcgi?cmd=search&db=gene&term=Fos) | FBJ osteosarcoma oncogene | < 1e-07 | 1804.66 | 294.09 | 6.14 |
| 2 |  | [T Cell Receptor Signaling Pathway](http://cgap.nci.nih.gov/Pathways/BioCarta/m_tcrPathway) | [Cd3d](http://www.ncbi.nlm.nih.gov/entrez/query.fcgi?cmd=search&db=gene&term=Cd3d) | CD3 antigen, delta polypeptide | 1.3e-05 | 154.41 | 106.54 | 1.45 |
| 3 |  | [T Cell Receptor Signaling Pathway](http://cgap.nci.nih.gov/Pathways/BioCarta/m_tcrPathway) | [Cd3g](http://www.ncbi.nlm.nih.gov/entrez/query.fcgi?cmd=search&db=gene&term=Cd3g) | CD3 antigen, gamma polypeptide | 5.02e-05 | 250.04 | 181.14 | 1.38 |
| 4 |  | [T Cell Receptor Signaling Pathway](http://cgap.nci.nih.gov/Pathways/BioCarta/m_tcrPathway) | [Map2k4](http://www.ncbi.nlm.nih.gov/entrez/query.fcgi?cmd=search&db=gene&term=Map2k4) | mitogen-activated protein kinase kinase 4 | 9.41e-05 | 341.42 | 495.55 | 0.69 |
| 5 |  | [T Cell Receptor Signaling Pathway](http://cgap.nci.nih.gov/Pathways/BioCarta/m_tcrPathway) | [Prkcb](http://www.ncbi.nlm.nih.gov/entrez/query.fcgi?cmd=search&db=gene&term=Prkcb) | protein kinase C, beta | 0.000107 | 173.52 | 135.08 | 1.28 |
| 6 |  | [T Cell Receptor Signaling Pathway](http://cgap.nci.nih.gov/Pathways/BioCarta/m_tcrPathway) | [Nfkbia](http://www.ncbi.nlm.nih.gov/entrez/query.fcgi?cmd=search&db=gene&term=Nfkbia) | nuclear factor of kappa light polypeptide gene enhancer in B-cells inhibitor, alpha | 0.0002631 | 1140.45 | 852.99 | 1.34 |
| 7 |  | [T Cell Receptor Signaling Pathway](http://cgap.nci.nih.gov/Pathways/BioCarta/m_tcrPathway) | [Nfkb1](http://www.ncbi.nlm.nih.gov/entrez/query.fcgi?cmd=search&db=gene&term=Nfkb1) | nuclear factor of kappa light polypeptide gene enhancer in B-cells 1, p105 | 0.0002841 | 636.36 | 804.29 | 0.79 |
| 8 |  | [T Cell Receptor Signaling Pathway](http://cgap.nci.nih.gov/Pathways/BioCarta/m_tcrPathway) | [Jun](http://www.ncbi.nlm.nih.gov/entrez/query.fcgi?cmd=search&db=gene&term=Jun) | Jun oncogene | 0.0005914 | 270.82 | 205.78 | 1.32 |
| 9 |  | [T Cell Receptor Signaling Pathway](http://cgap.nci.nih.gov/Pathways/BioCarta/m_tcrPathway) | [Vav1](http://www.ncbi.nlm.nih.gov/entrez/query.fcgi?cmd=search&db=gene&term=Vav1) | vav 1 oncogene | 0.0006129 | 23.74 | 19.16 | 1.24 |
| 10 |  | [T Cell Receptor Signaling Pathway](http://cgap.nci.nih.gov/Pathways/BioCarta/m_tcrPathway) | [Tcra](http://www.ncbi.nlm.nih.gov/entrez/query.fcgi?cmd=search&db=gene&term=Tcra) | T-cell receptor alpha chain | 0.0007768 | 29.96 | 24.36 | 1.23 |
| 11 |  | [T Cell Receptor Signaling Pathway](http://cgap.nci.nih.gov/Pathways/BioCarta/m_tcrPathway) | [Zap70](http://www.ncbi.nlm.nih.gov/entrez/query.fcgi?cmd=search&db=gene&term=Zap70) | zeta-chain (TCR) associated protein kinase | 0.001192 | 540.43 | 424.72 | 1.27 |
| 12 |  | [T Cell Receptor Signaling Pathway](http://cgap.nci.nih.gov/Pathways/BioCarta/m_tcrPathway) | [Lck](http://www.ncbi.nlm.nih.gov/entrez/query.fcgi?cmd=search&db=gene&term=Lck) | lymphocyte protein tyrosine kinase | 0.0016033 | 90.08 | 69.9 | 1.29 |
| 13 |  | [T Cell Receptor Signaling Pathway](http://cgap.nci.nih.gov/Pathways/BioCarta/m_tcrPathway) | [Sos1](http://www.ncbi.nlm.nih.gov/entrez/query.fcgi?cmd=search&db=gene&term=Sos1) | son of sevenless homolog 1 (Drosophila) | 0.0052437 | 62.98 | 76.11 | 0.83 |
| 14 |  | [T Cell Receptor Signaling Pathway](http://cgap.nci.nih.gov/Pathways/BioCarta/m_tcrPathway) | [Fyn](http://www.ncbi.nlm.nih.gov/entrez/query.fcgi?cmd=search&db=gene&term=Fyn) | Fyn proto-oncogene | 0.0296932 | 108.73 | 88.59 | 1.23 |
| 15 |  | [T Cell Receptor Signaling Pathway](http://cgap.nci.nih.gov/Pathways/BioCarta/m_tcrPathway) | [Mapk8](http://www.ncbi.nlm.nih.gov/entrez/query.fcgi?cmd=search&db=gene&term=Mapk8) | mitogen-activated protein kinase 8 | 0.0431864 | 31.06 | 25.43 | 1.22 |
| 16 |  | [T Cell Receptor Signaling Pathway](http://cgap.nci.nih.gov/Pathways/BioCarta/m_tcrPathway) | [Nfatc2](http://www.ncbi.nlm.nih.gov/entrez/query.fcgi?cmd=search&db=gene&term=Nfatc2) | nuclear factor of activated T-cells, cytoplasmic, calcineurin-dependent 2 | 0.0683684 | 14.9 | 16.65 | 0.9 |
| 17 |  | [T Cell Receptor Signaling Pathway](http://cgap.nci.nih.gov/Pathways/BioCarta/m_tcrPathway) | [Nfatc3](http://www.ncbi.nlm.nih.gov/entrez/query.fcgi?cmd=search&db=gene&term=Nfatc3) | nuclear factor of activated T-cells, cytoplasmic, calcineurin-dependent 3 | 0.1778903 | 211.8 | 241.23 | 0.88 |
| 18 |  | [T Cell Receptor Signaling Pathway](http://cgap.nci.nih.gov/Pathways/BioCarta/m_tcrPathway) | [Pik3cg](http://www.ncbi.nlm.nih.gov/entrez/query.fcgi?cmd=search&db=gene&term=Pik3cg) | phosphoinositide-3-kinase, catalytic, gamma polypeptide | 0.3272953 | 16.25 | 14.76 | 1.1 |
| 19 |  | [T Cell Receptor Signaling Pathway](http://cgap.nci.nih.gov/Pathways/BioCarta/m_tcrPathway) | [Cd247](http://www.ncbi.nlm.nih.gov/entrez/query.fcgi?cmd=search&db=gene&term=Cd247) | CD247 antigen | 0.50389 | 12.94 | 13.92 | 0.93 |
| 20 |  | [T Cell Receptor Signaling Pathway](http://cgap.nci.nih.gov/Pathways/BioCarta/m_tcrPathway) | [Pik3r1](http://www.ncbi.nlm.nih.gov/entrez/query.fcgi?cmd=search&db=gene&term=Pik3r1) | phosphatidylinositol 3-kinase, regulatory subunit, polypeptide 1 (p85 alpha) | 0.6503694 | 113.16 | 110.05 | 1.03 |
| 1 | m_mPRPathway | [How Progesterone Initiates the Oocyte Maturation](http://cgap.nci.nih.gov/Pathways/BioCarta/m_mPRPathway) | [Cap1](http://www.ncbi.nlm.nih.gov/entrez/query.fcgi?cmd=search&db=gene&term=Cap1) | CAP, adenylate cyclase-associated protein 1 (yeast) | < 1e-07 | 334.44 | 61.37 | 5.45 |
| 2 |  | [How Progesterone Initiates the Oocyte Maturation](http://cgap.nci.nih.gov/Pathways/BioCarta/m_mPRPathway) | [Ccnb1](http://www.ncbi.nlm.nih.gov/entrez/query.fcgi?cmd=search&db=gene&term=Ccnb1) | cyclin B1 | 2.2e-06 | 132.48 | 194.83 | 0.68 |
| 3 |  | [How Progesterone Initiates the Oocyte Maturation](http://cgap.nci.nih.gov/Pathways/BioCarta/m_mPRPathway) | [Cdk1](http://www.ncbi.nlm.nih.gov/entrez/query.fcgi?cmd=search&db=gene&term=Cdk1) | cyclin-dependent kinase 1 | 4.79e-05 | 218.93 | 282.88 | 0.77 |
| 4 |  | [How Progesterone Initiates the Oocyte Maturation](http://cgap.nci.nih.gov/Pathways/BioCarta/m_mPRPathway) | [Prkacb](http://www.ncbi.nlm.nih.gov/entrez/query.fcgi?cmd=search&db=gene&term=Prkacb) | protein kinase, cAMP dependent, catalytic, beta | 0.0006388 | 238.56 | 190.67 | 1.25 |
| 5 |  | [How Progesterone Initiates the Oocyte Maturation](http://cgap.nci.nih.gov/Pathways/BioCarta/m_mPRPathway) | [Cdc25c](http://www.ncbi.nlm.nih.gov/entrez/query.fcgi?cmd=search&db=gene&term=Cdc25c) | cell division cycle 25 homolog C (S. pombe) | 0.022479 | 49.55 | 58.92 | 0.84 |
| 6 |  | [How Progesterone Initiates the Oocyte Maturation](http://cgap.nci.nih.gov/Pathways/BioCarta/m_mPRPathway) | [Mapk1](http://www.ncbi.nlm.nih.gov/entrez/query.fcgi?cmd=search&db=gene&term=Mapk1) | mitogen-activated protein kinase 1 | 0.9818276 | 246.1 | 247.01 | 1 |
| 1 | m_akap95Pathway | [AKAP95 role in mitosis and chromosome dynamics](http://cgap.nci.nih.gov/Pathways/BioCarta/m_akap95Pathway) | [Ccnb1](http://www.ncbi.nlm.nih.gov/entrez/query.fcgi?cmd=search&db=gene&term=Ccnb1) | cyclin B1 | 2.2e-06 | 132.48 | 194.83 | 0.68 |
| 2 |  | [AKAP95 role in mitosis and chromosome dynamics](http://cgap.nci.nih.gov/Pathways/BioCarta/m_akap95Pathway) | [Ncapd2](http://www.ncbi.nlm.nih.gov/entrez/query.fcgi?cmd=search&db=gene&term=Ncapd2) | non-SMC condensin I complex, subunit D2 | 1.04e-05 | 223.32 | 330.57 | 0.68 |
| 3 |  | [AKAP95 role in mitosis and chromosome dynamics](http://cgap.nci.nih.gov/Pathways/BioCarta/m_akap95Pathway) | [Cdk1](http://www.ncbi.nlm.nih.gov/entrez/query.fcgi?cmd=search&db=gene&term=Cdk1) | cyclin-dependent kinase 1 | 4.79e-05 | 218.93 | 282.88 | 0.77 |
| 4 |  | [AKAP95 role in mitosis and chromosome dynamics](http://cgap.nci.nih.gov/Pathways/BioCarta/m_akap95Pathway) | [Prkacb](http://www.ncbi.nlm.nih.gov/entrez/query.fcgi?cmd=search&db=gene&term=Prkacb) | protein kinase, cAMP dependent, catalytic, beta | 0.0006388 | 238.56 | 190.67 | 1.25 |
| 5 |  | [AKAP95 role in mitosis and chromosome dynamics](http://cgap.nci.nih.gov/Pathways/BioCarta/m_akap95Pathway) | [Ddx5](http://www.ncbi.nlm.nih.gov/entrez/query.fcgi?cmd=search&db=gene&term=Ddx5) | DEAD (Asp-Glu-Ala-Asp) box polypeptide 5 | 0.0555016 | 2922.46 | 3186.66 | 0.92 |
| 1 | m_EfpPathway | [Estrogen-responsive protein Efp controls cell cycle and breast tumors growth](http://cgap.nci.nih.gov/Pathways/BioCarta/m_EfpPathway) | [Ccnb2](http://www.ncbi.nlm.nih.gov/entrez/query.fcgi?cmd=search&db=gene&term=Ccnb2) | cyclin B2 | 9e-07 | 218.57 | 337.32 | 0.65 |
| 2 |  | [Estrogen-responsive protein Efp controls cell cycle and breast tumors growth](http://cgap.nci.nih.gov/Pathways/BioCarta/m_EfpPathway) | [Ccnb1](http://www.ncbi.nlm.nih.gov/entrez/query.fcgi?cmd=search&db=gene&term=Ccnb1) | cyclin B1 | 2.2e-06 | 132.48 | 194.83 | 0.68 |
| 3 |  | [Estrogen-responsive protein Efp controls cell cycle and breast tumors growth](http://cgap.nci.nih.gov/Pathways/BioCarta/m_EfpPathway) | [Cdk1](http://www.ncbi.nlm.nih.gov/entrez/query.fcgi?cmd=search&db=gene&term=Cdk1) | cyclin-dependent kinase 1 | 4.79e-05 | 218.93 | 282.88 | 0.77 |
| 4 |  | [Estrogen-responsive protein Efp controls cell cycle and breast tumors growth](http://cgap.nci.nih.gov/Pathways/BioCarta/m_EfpPathway) | [Trp53](http://www.ncbi.nlm.nih.gov/entrez/query.fcgi?cmd=search&db=gene&term=Trp53) | transformation related protein 53 | 0.0027856 | 11.53 | 14.37 | 0.8 |
| 5 |  | [Estrogen-responsive protein Efp controls cell cycle and breast tumors growth](http://cgap.nci.nih.gov/Pathways/BioCarta/m_EfpPathway) | [Cdk6](http://www.ncbi.nlm.nih.gov/entrez/query.fcgi?cmd=search&db=gene&term=Cdk6) | cyclin-dependent kinase 6 | 0.2236676 | 23.39 | 21.96 | 1.06 |
| 1 | m_cardiacegfPathway | [Role of EGF Receptor Transactivation by GPCRs in Cardiac Hypertrophy](http://cgap.nci.nih.gov/Pathways/BioCarta/m_cardiacegfPathway) | [Fos](http://www.ncbi.nlm.nih.gov/entrez/query.fcgi?cmd=search&db=gene&term=Fos) | FBJ osteosarcoma oncogene | < 1e-07 | 1804.66 | 294.09 | 6.14 |
| 2 |  | [Role of EGF Receptor Transactivation by GPCRs in Cardiac Hypertrophy](http://cgap.nci.nih.gov/Pathways/BioCarta/m_cardiacegfPathway) | [Prkcb](http://www.ncbi.nlm.nih.gov/entrez/query.fcgi?cmd=search&db=gene&term=Prkcb) | protein kinase C, beta | 0.000107 | 173.52 | 135.08 | 1.28 |
| 3 |  | [Role of EGF Receptor Transactivation by GPCRs in Cardiac Hypertrophy](http://cgap.nci.nih.gov/Pathways/BioCarta/m_cardiacegfPathway) | [Nfkb1](http://www.ncbi.nlm.nih.gov/entrez/query.fcgi?cmd=search&db=gene&term=Nfkb1) | nuclear factor of kappa light polypeptide gene enhancer in B-cells 1, p105 | 0.0002841 | 636.36 | 804.29 | 0.79 |
| 4 |  | [Role of EGF Receptor Transactivation by GPCRs in Cardiac Hypertrophy](http://cgap.nci.nih.gov/Pathways/BioCarta/m_cardiacegfPathway) | [Jun](http://www.ncbi.nlm.nih.gov/entrez/query.fcgi?cmd=search&db=gene&term=Jun) | Jun oncogene | 0.0005914 | 270.82 | 205.78 | 1.32 |
| 5 |  | [Role of EGF Receptor Transactivation by GPCRs in Cardiac Hypertrophy](http://cgap.nci.nih.gov/Pathways/BioCarta/m_cardiacegfPathway) | [Egf](http://www.ncbi.nlm.nih.gov/entrez/query.fcgi?cmd=search&db=gene&term=Egf) | epidermal growth factor | 0.0062555 | 12.25 | 11.05 | 1.11 |
| 6 |  | [Role of EGF Receptor Transactivation by GPCRs in Cardiac Hypertrophy](http://cgap.nci.nih.gov/Pathways/BioCarta/m_cardiacegfPathway) | [Ednra](http://www.ncbi.nlm.nih.gov/entrez/query.fcgi?cmd=search&db=gene&term=Ednra) | endothelin receptor type A | 0.0212656 | 10.58 | 8.97 | 1.18 |
| 7 |  | [Role of EGF Receptor Transactivation by GPCRs in Cardiac Hypertrophy](http://cgap.nci.nih.gov/Pathways/BioCarta/m_cardiacegfPathway) | [Edn1](http://www.ncbi.nlm.nih.gov/entrez/query.fcgi?cmd=search&db=gene&term=Edn1) | endothelin 1 | 0.0215425 | 70.81 | 56.14 | 1.26 |
| 1 | m_tollpathway | [Toll-Like Receptor Pathway](http://cgap.nci.nih.gov/Pathways/BioCarta/m_tollpathway) | [Fos](http://www.ncbi.nlm.nih.gov/entrez/query.fcgi?cmd=search&db=gene&term=Fos) | FBJ osteosarcoma oncogene | < 1e-07 | 1804.66 | 294.09 | 6.14 |
| 2 |  | [Toll-Like Receptor Pathway](http://cgap.nci.nih.gov/Pathways/BioCarta/m_tollpathway) | [Ppara](http://www.ncbi.nlm.nih.gov/entrez/query.fcgi?cmd=search&db=gene&term=Ppara) | peroxisome proliferator activated receptor alpha | 1.96e-05 | 282.28 | 188.35 | 1.5 |
| 3 |  | [Toll-Like Receptor Pathway](http://cgap.nci.nih.gov/Pathways/BioCarta/m_tollpathway) | [Map2k4](http://www.ncbi.nlm.nih.gov/entrez/query.fcgi?cmd=search&db=gene&term=Map2k4) | mitogen-activated protein kinase kinase 4 | 9.41e-05 | 341.42 | 495.55 | 0.69 |
| 4 |  | [Toll-Like Receptor Pathway](http://cgap.nci.nih.gov/Pathways/BioCarta/m_tollpathway) | [Nfkbia](http://www.ncbi.nlm.nih.gov/entrez/query.fcgi?cmd=search&db=gene&term=Nfkbia) | nuclear factor of kappa light polypeptide gene enhancer in B-cells inhibitor, alpha | 0.0002631 | 1140.45 | 852.99 | 1.34 |
| 5 |  | [Toll-Like Receptor Pathway](http://cgap.nci.nih.gov/Pathways/BioCarta/m_tollpathway) | [Nfkb1](http://www.ncbi.nlm.nih.gov/entrez/query.fcgi?cmd=search&db=gene&term=Nfkb1) | nuclear factor of kappa light polypeptide gene enhancer in B-cells 1, p105 | 0.0002841 | 636.36 | 804.29 | 0.79 |
| 6 |  | [Toll-Like Receptor Pathway](http://cgap.nci.nih.gov/Pathways/BioCarta/m_tollpathway) | [Jun](http://www.ncbi.nlm.nih.gov/entrez/query.fcgi?cmd=search&db=gene&term=Jun) | Jun oncogene | 0.0005914 | 270.82 | 205.78 | 1.32 |
| 7 |  | [Toll-Like Receptor Pathway](http://cgap.nci.nih.gov/Pathways/BioCarta/m_tollpathway) | [Mapk14](http://www.ncbi.nlm.nih.gov/entrez/query.fcgi?cmd=search&db=gene&term=Mapk14) | mitogen-activated protein kinase 14 | 0.0123923 | 55 | 71.64 | 0.77 |
| 8 |  | [Toll-Like Receptor Pathway](http://cgap.nci.nih.gov/Pathways/BioCarta/m_tollpathway) | [Mapk8](http://www.ncbi.nlm.nih.gov/entrez/query.fcgi?cmd=search&db=gene&term=Mapk8) | mitogen-activated protein kinase 8 | 0.0431864 | 31.06 | 25.43 | 1.22 |
| 9 |  | [Toll-Like Receptor Pathway](http://cgap.nci.nih.gov/Pathways/BioCarta/m_tollpathway) | [Map2k6](http://www.ncbi.nlm.nih.gov/entrez/query.fcgi?cmd=search&db=gene&term=Map2k6) | mitogen-activated protein kinase kinase 6 | 0.3995002 | 119.46 | 113.45 | 1.05 |
| 10 |  | [Toll-Like Receptor Pathway](http://cgap.nci.nih.gov/Pathways/BioCarta/m_tollpathway) | [Pglyrp1](http://www.ncbi.nlm.nih.gov/entrez/query.fcgi?cmd=search&db=gene&term=Pglyrp1) | peptidoglycan recognition protein 1 | 0.447871 | 13.43 | 13.77 | 0.98 |
| 1 | m_stathminPathway | [Stathmin and breast cancer resistance to antimicrotubule agents](http://cgap.nci.nih.gov/Pathways/BioCarta/m_stathminPathway) | [Ccnb1](http://www.ncbi.nlm.nih.gov/entrez/query.fcgi?cmd=search&db=gene&term=Ccnb1) | cyclin B1 | 2.2e-06 | 132.48 | 194.83 | 0.68 |
| 2 |  | [Stathmin and breast cancer resistance to antimicrotubule agents](http://cgap.nci.nih.gov/Pathways/BioCarta/m_stathminPathway) | [Cd3d](http://www.ncbi.nlm.nih.gov/entrez/query.fcgi?cmd=search&db=gene&term=Cd3d) | CD3 antigen, delta polypeptide | 1.3e-05 | 154.41 | 106.54 | 1.45 |
| 3 |  | [Stathmin and breast cancer resistance to antimicrotubule agents](http://cgap.nci.nih.gov/Pathways/BioCarta/m_stathminPathway) | [Cdk1](http://www.ncbi.nlm.nih.gov/entrez/query.fcgi?cmd=search&db=gene&term=Cdk1) | cyclin-dependent kinase 1 | 4.79e-05 | 218.93 | 282.88 | 0.77 |
| 4 |  | [Stathmin and breast cancer resistance to antimicrotubule agents](http://cgap.nci.nih.gov/Pathways/BioCarta/m_stathminPathway) | [Cd3g](http://www.ncbi.nlm.nih.gov/entrez/query.fcgi?cmd=search&db=gene&term=Cd3g) | CD3 antigen, gamma polypeptide | 5.02e-05 | 250.04 | 181.14 | 1.38 |
| 5 |  | [Stathmin and breast cancer resistance to antimicrotubule agents](http://cgap.nci.nih.gov/Pathways/BioCarta/m_stathminPathway) | [Prkacb](http://www.ncbi.nlm.nih.gov/entrez/query.fcgi?cmd=search&db=gene&term=Prkacb) | protein kinase, cAMP dependent, catalytic, beta | 0.0006388 | 238.56 | 190.67 | 1.25 |
| 6 |  | [Stathmin and breast cancer resistance to antimicrotubule agents](http://cgap.nci.nih.gov/Pathways/BioCarta/m_stathminPathway) | [Rb1cc1](http://www.ncbi.nlm.nih.gov/entrez/query.fcgi?cmd=search&db=gene&term=Rb1cc1) | RB1-inducible coiled-coil 1 | 0.0741236 | 437.25 | 515.22 | 0.85 |
| 7 |  | [Stathmin and breast cancer resistance to antimicrotubule agents](http://cgap.nci.nih.gov/Pathways/BioCarta/m_stathminPathway) | [Cd247](http://www.ncbi.nlm.nih.gov/entrez/query.fcgi?cmd=search&db=gene&term=Cd247) | CD247 antigen | 0.50389 | 12.94 | 13.92 | 0.93 |
| 1 | m_chemicalPathway | [Apoptotic Signaling in Response to DNA Damage](http://cgap.nci.nih.gov/Pathways/BioCarta/m_chemicalPathway) | [Stat1](http://www.ncbi.nlm.nih.gov/entrez/query.fcgi?cmd=search&db=gene&term=Stat1) | signal transducer and activator of transcription 1 | < 1e-07 | 804.27 | 394.42 | 2.04 |
| 2 |  | [Apoptotic Signaling in Response to DNA Damage](http://cgap.nci.nih.gov/Pathways/BioCarta/m_chemicalPathway) | [Prkcb](http://www.ncbi.nlm.nih.gov/entrez/query.fcgi?cmd=search&db=gene&term=Prkcb) | protein kinase C, beta | 0.000107 | 173.52 | 135.08 | 1.28 |
| 3 |  | [Apoptotic Signaling in Response to DNA Damage](http://cgap.nci.nih.gov/Pathways/BioCarta/m_chemicalPathway) | [Bid](http://www.ncbi.nlm.nih.gov/entrez/query.fcgi?cmd=search&db=gene&term=Bid) | BH3 interacting domain death agonist | 0.0001907 | 76.31 | 56.96 | 1.34 |
| 4 |  | [Apoptotic Signaling in Response to DNA Damage](http://cgap.nci.nih.gov/Pathways/BioCarta/m_chemicalPathway) | [Trp53](http://www.ncbi.nlm.nih.gov/entrez/query.fcgi?cmd=search&db=gene&term=Trp53) | transformation related protein 53 | 0.0027856 | 11.53 | 14.37 | 0.8 |
| 5 |  | [Apoptotic Signaling in Response to DNA Damage](http://cgap.nci.nih.gov/Pathways/BioCarta/m_chemicalPathway) | [Eif2s1](http://www.ncbi.nlm.nih.gov/entrez/query.fcgi?cmd=search&db=gene&term=Eif2s1) | eukaryotic translation initiation factor 2, subunit 1 alpha | 0.0653904 | 20.59 | 17.93 | 1.15 |
| 1 | m_egfPathway | [EGF Signaling Pathway](http://cgap.nci.nih.gov/Pathways/BioCarta/m_egfPathway) | [Fos](http://www.ncbi.nlm.nih.gov/entrez/query.fcgi?cmd=search&db=gene&term=Fos) | FBJ osteosarcoma oncogene | < 1e-07 | 1804.66 | 294.09 | 6.14 |
| 2 |  | [EGF Signaling Pathway](http://cgap.nci.nih.gov/Pathways/BioCarta/m_egfPathway) | [Stat1](http://www.ncbi.nlm.nih.gov/entrez/query.fcgi?cmd=search&db=gene&term=Stat1) | signal transducer and activator of transcription 1 | < 1e-07 | 804.27 | 394.42 | 2.04 |
| 3 |  | [EGF Signaling Pathway](http://cgap.nci.nih.gov/Pathways/BioCarta/m_egfPathway) | [Map2k4](http://www.ncbi.nlm.nih.gov/entrez/query.fcgi?cmd=search&db=gene&term=Map2k4) | mitogen-activated protein kinase kinase 4 | 9.41e-05 | 341.42 | 495.55 | 0.69 |
| 4 |  | [EGF Signaling Pathway](http://cgap.nci.nih.gov/Pathways/BioCarta/m_egfPathway) | [Prkcb](http://www.ncbi.nlm.nih.gov/entrez/query.fcgi?cmd=search&db=gene&term=Prkcb) | protein kinase C, beta | 0.000107 | 173.52 | 135.08 | 1.28 |
| 5 |  | [EGF Signaling Pathway](http://cgap.nci.nih.gov/Pathways/BioCarta/m_egfPathway) | [Jun](http://www.ncbi.nlm.nih.gov/entrez/query.fcgi?cmd=search&db=gene&term=Jun) | Jun oncogene | 0.0005914 | 270.82 | 205.78 | 1.32 |
| 6 |  | [EGF Signaling Pathway](http://cgap.nci.nih.gov/Pathways/BioCarta/m_egfPathway) | [Sos1](http://www.ncbi.nlm.nih.gov/entrez/query.fcgi?cmd=search&db=gene&term=Sos1) | son of sevenless homolog 1 (Drosophila) | 0.0052437 | 62.98 | 76.11 | 0.83 |
| 7 |  | [EGF Signaling Pathway](http://cgap.nci.nih.gov/Pathways/BioCarta/m_egfPathway) | [Egf](http://www.ncbi.nlm.nih.gov/entrez/query.fcgi?cmd=search&db=gene&term=Egf) | epidermal growth factor | 0.0062555 | 12.25 | 11.05 | 1.11 |
| 8 |  | [EGF Signaling Pathway](http://cgap.nci.nih.gov/Pathways/BioCarta/m_egfPathway) | [Csnk2a2](http://www.ncbi.nlm.nih.gov/entrez/query.fcgi?cmd=search&db=gene&term=Csnk2a2) | casein kinase 2, alpha prime polypeptide | 0.0102407 | 1559.16 | 1719.9 | 0.91 |
| 9 |  | [EGF Signaling Pathway](http://cgap.nci.nih.gov/Pathways/BioCarta/m_egfPathway) | [Stat5a](http://www.ncbi.nlm.nih.gov/entrez/query.fcgi?cmd=search&db=gene&term=Stat5a) | signal transducer and activator of transcription 5A | 0.0353627 | 63.97 | 73.88 | 0.87 |
| 10 |  | [EGF Signaling Pathway](http://cgap.nci.nih.gov/Pathways/BioCarta/m_egfPathway) | [Mapk8](http://www.ncbi.nlm.nih.gov/entrez/query.fcgi?cmd=search&db=gene&term=Mapk8) | mitogen-activated protein kinase 8 | 0.0431864 | 31.06 | 25.43 | 1.22 |
| 11 |  | [EGF Signaling Pathway](http://cgap.nci.nih.gov/Pathways/BioCarta/m_egfPathway) | [Jak1](http://www.ncbi.nlm.nih.gov/entrez/query.fcgi?cmd=search&db=gene&term=Jak1) | Janus kinase 1 | 0.1036342 | 128.89 | 156.56 | 0.82 |
| 12 |  | [EGF Signaling Pathway](http://cgap.nci.nih.gov/Pathways/BioCarta/m_egfPathway) | [Csnk2a1](http://www.ncbi.nlm.nih.gov/entrez/query.fcgi?cmd=search&db=gene&term=Csnk2a1) | casein kinase 2, alpha 1 polypeptide | 0.5759663 | 255.89 | 272.9 | 0.94 |
| 13 |  | [EGF Signaling Pathway](http://cgap.nci.nih.gov/Pathways/BioCarta/m_egfPathway) | [Pik3r1](http://www.ncbi.nlm.nih.gov/entrez/query.fcgi?cmd=search&db=gene&term=Pik3r1) | phosphatidylinositol 3-kinase, regulatory subunit, polypeptide 1 (p85 alpha) | 0.6503694 | 113.16 | 110.05 | 1.03 |
| 1 | m_Ccr5Pathway | [Pertussis toxin-insensitive CCR5 Signaling in Macrophage](http://cgap.nci.nih.gov/Pathways/BioCarta/m_Ccr5Pathway) | [Fos](http://www.ncbi.nlm.nih.gov/entrez/query.fcgi?cmd=search&db=gene&term=Fos) | FBJ osteosarcoma oncogene | < 1e-07 | 1804.66 | 294.09 | 6.14 |
| 2 |  | [Pertussis toxin-insensitive CCR5 Signaling in Macrophage](http://cgap.nci.nih.gov/Pathways/BioCarta/m_Ccr5Pathway) | [Prkcb](http://www.ncbi.nlm.nih.gov/entrez/query.fcgi?cmd=search&db=gene&term=Prkcb) | protein kinase C, beta | 0.000107 | 173.52 | 135.08 | 1.28 |
| 3 |  | [Pertussis toxin-insensitive CCR5 Signaling in Macrophage](http://cgap.nci.nih.gov/Pathways/BioCarta/m_Ccr5Pathway) | [Cxcl12](http://www.ncbi.nlm.nih.gov/entrez/query.fcgi?cmd=search&db=gene&term=Cxcl12) | chemokine (C-X-C motif) ligand 12 | 0.0001346 | 70.7 | 43.22 | 1.64 |
| 4 |  | [Pertussis toxin-insensitive CCR5 Signaling in Macrophage](http://cgap.nci.nih.gov/Pathways/BioCarta/m_Ccr5Pathway) | [Jun](http://www.ncbi.nlm.nih.gov/entrez/query.fcgi?cmd=search&db=gene&term=Jun) | Jun oncogene | 0.0005914 | 270.82 | 205.78 | 1.32 |
| 5 |  | [Pertussis toxin-insensitive CCR5 Signaling in Macrophage](http://cgap.nci.nih.gov/Pathways/BioCarta/m_Ccr5Pathway) | [Ccl4](http://www.ncbi.nlm.nih.gov/entrez/query.fcgi?cmd=search&db=gene&term=Ccl4) | chemokine (C-C motif) ligand 4 | 0.0061042 | 76.55 | 64.65 | 1.18 |
| 6 |  | [Pertussis toxin-insensitive CCR5 Signaling in Macrophage](http://cgap.nci.nih.gov/Pathways/BioCarta/m_Ccr5Pathway) | [Mapk14](http://www.ncbi.nlm.nih.gov/entrez/query.fcgi?cmd=search&db=gene&term=Mapk14) | mitogen-activated protein kinase 14 | 0.0123923 | 55 | 71.64 | 0.77 |
| 7 |  | [Pertussis toxin-insensitive CCR5 Signaling in Macrophage](http://cgap.nci.nih.gov/Pathways/BioCarta/m_Ccr5Pathway) | [Mapk8](http://www.ncbi.nlm.nih.gov/entrez/query.fcgi?cmd=search&db=gene&term=Mapk8) | mitogen-activated protein kinase 8 | 0.0431864 | 31.06 | 25.43 | 1.22 |
| 8 |  | [Pertussis toxin-insensitive CCR5 Signaling in Macrophage](http://cgap.nci.nih.gov/Pathways/BioCarta/m_Ccr5Pathway) | [Cxcr4](http://www.ncbi.nlm.nih.gov/entrez/query.fcgi?cmd=search&db=gene&term=Cxcr4) | chemokine (C-X-C motif) receptor 4 | 0.1674703 | 10.02 | 9.27 | 1.08 |
| 1 | m_cdMacPathway | [Cadmium induces DNA synthesis and proliferation in macrophages](http://cgap.nci.nih.gov/Pathways/BioCarta/m_cdMacPathway) | [Fos](http://www.ncbi.nlm.nih.gov/entrez/query.fcgi?cmd=search&db=gene&term=Fos) | FBJ osteosarcoma oncogene | < 1e-07 | 1804.66 | 294.09 | 6.14 |
| 2 |  | [Cadmium induces DNA synthesis and proliferation in macrophages](http://cgap.nci.nih.gov/Pathways/BioCarta/m_cdMacPathway) | [Prkcb](http://www.ncbi.nlm.nih.gov/entrez/query.fcgi?cmd=search&db=gene&term=Prkcb) | protein kinase C, beta | 0.000107 | 173.52 | 135.08 | 1.28 |
| 3 |  | [Cadmium induces DNA synthesis and proliferation in macrophages](http://cgap.nci.nih.gov/Pathways/BioCarta/m_cdMacPathway) | [Nfkbia](http://www.ncbi.nlm.nih.gov/entrez/query.fcgi?cmd=search&db=gene&term=Nfkbia) | nuclear factor of kappa light polypeptide gene enhancer in B-cells inhibitor, alpha | 0.0002631 | 1140.45 | 852.99 | 1.34 |
| 4 |  | [Cadmium induces DNA synthesis and proliferation in macrophages](http://cgap.nci.nih.gov/Pathways/BioCarta/m_cdMacPathway) | [Nfkb1](http://www.ncbi.nlm.nih.gov/entrez/query.fcgi?cmd=search&db=gene&term=Nfkb1) | nuclear factor of kappa light polypeptide gene enhancer in B-cells 1, p105 | 0.0002841 | 636.36 | 804.29 | 0.79 |
| 5 |  | [Cadmium induces DNA synthesis and proliferation in macrophages](http://cgap.nci.nih.gov/Pathways/BioCarta/m_cdMacPathway) | [Jun](http://www.ncbi.nlm.nih.gov/entrez/query.fcgi?cmd=search&db=gene&term=Jun) | Jun oncogene | 0.0005914 | 270.82 | 205.78 | 1.32 |
| 6 |  | [Cadmium induces DNA synthesis and proliferation in macrophages](http://cgap.nci.nih.gov/Pathways/BioCarta/m_cdMacPathway) | [Tnf](http://www.ncbi.nlm.nih.gov/entrez/query.fcgi?cmd=search&db=gene&term=Tnf) | tumor necrosis factor | 0.4350528 | 59.58 | 63.31 | 0.94 |
| 7 |  | [Cadmium induces DNA synthesis and proliferation in macrophages](http://cgap.nci.nih.gov/Pathways/BioCarta/m_cdMacPathway) | [Mapk1](http://www.ncbi.nlm.nih.gov/entrez/query.fcgi?cmd=search&db=gene&term=Mapk1) | mitogen-activated protein kinase 1 | 0.9818276 | 246.1 | 247.01 | 1 |
| 1 | m_tsp1Pathway | [TSP-1 Induced Apoptosis in Microvascular Endothelial Cell](http://cgap.nci.nih.gov/Pathways/BioCarta/m_tsp1Pathway) | [Fos](http://www.ncbi.nlm.nih.gov/entrez/query.fcgi?cmd=search&db=gene&term=Fos) | FBJ osteosarcoma oncogene | < 1e-07 | 1804.66 | 294.09 | 6.14 |
| 2 |  | [TSP-1 Induced Apoptosis in Microvascular Endothelial Cell](http://cgap.nci.nih.gov/Pathways/BioCarta/m_tsp1Pathway) | [Cd36](http://www.ncbi.nlm.nih.gov/entrez/query.fcgi?cmd=search&db=gene&term=Cd36) | CD36 antigen | 0.0002126 | 95.81 | 76.85 | 1.25 |
| 3 |  | [TSP-1 Induced Apoptosis in Microvascular Endothelial Cell](http://cgap.nci.nih.gov/Pathways/BioCarta/m_tsp1Pathway) | [Jun](http://www.ncbi.nlm.nih.gov/entrez/query.fcgi?cmd=search&db=gene&term=Jun) | Jun oncogene | 0.0005914 | 270.82 | 205.78 | 1.32 |
| 4 |  | [TSP-1 Induced Apoptosis in Microvascular Endothelial Cell](http://cgap.nci.nih.gov/Pathways/BioCarta/m_tsp1Pathway) | [Thbs1](http://www.ncbi.nlm.nih.gov/entrez/query.fcgi?cmd=search&db=gene&term=Thbs1) | thrombospondin 1 | 0.0120744 | 168.73 | 196.89 | 0.86 |
| 5 |  | [TSP-1 Induced Apoptosis in Microvascular Endothelial Cell](http://cgap.nci.nih.gov/Pathways/BioCarta/m_tsp1Pathway) | [Mapk14](http://www.ncbi.nlm.nih.gov/entrez/query.fcgi?cmd=search&db=gene&term=Mapk14) | mitogen-activated protein kinase 14 | 0.0123923 | 55 | 71.64 | 0.77 |
| 6 |  | [TSP-1 Induced Apoptosis in Microvascular Endothelial Cell](http://cgap.nci.nih.gov/Pathways/BioCarta/m_tsp1Pathway) | [Fyn](http://www.ncbi.nlm.nih.gov/entrez/query.fcgi?cmd=search&db=gene&term=Fyn) | Fyn proto-oncogene | 0.0296932 | 108.73 | 88.59 | 1.23 |
| 1 | m_cd40Pathway | [CD40L Signaling Pathway](http://cgap.nci.nih.gov/Pathways/BioCarta/m_cd40Pathway) | [Dusp1](http://www.ncbi.nlm.nih.gov/entrez/query.fcgi?cmd=search&db=gene&term=Dusp1) | dual specificity phosphatase 1 | < 1e-07 | 1513.19 | 673.69 | 2.25 |
| 2 |  | [CD40L Signaling Pathway](http://cgap.nci.nih.gov/Pathways/BioCarta/m_cd40Pathway) | [Nfkbia](http://www.ncbi.nlm.nih.gov/entrez/query.fcgi?cmd=search&db=gene&term=Nfkbia) | nuclear factor of kappa light polypeptide gene enhancer in B-cells inhibitor, alpha | 0.0002631 | 1140.45 | 852.99 | 1.34 |
| 3 |  | [CD40L Signaling Pathway](http://cgap.nci.nih.gov/Pathways/BioCarta/m_cd40Pathway) | [Nfkb1](http://www.ncbi.nlm.nih.gov/entrez/query.fcgi?cmd=search&db=gene&term=Nfkb1) | nuclear factor of kappa light polypeptide gene enhancer in B-cells 1, p105 | 0.0002841 | 636.36 | 804.29 | 0.79 |
| 4 |  | [CD40L Signaling Pathway](http://cgap.nci.nih.gov/Pathways/BioCarta/m_cd40Pathway) | [Tnfaip3](http://www.ncbi.nlm.nih.gov/entrez/query.fcgi?cmd=search&db=gene&term=Tnfaip3) | tumor necrosis factor, alpha-induced protein 3 | 0.0079502 | 70.87 | 86.44 | 0.82 |
| 5 |  | [CD40L Signaling Pathway](http://cgap.nci.nih.gov/Pathways/BioCarta/m_cd40Pathway) | [Cd40lg](http://www.ncbi.nlm.nih.gov/entrez/query.fcgi?cmd=search&db=gene&term=Cd40lg) | CD40 ligand | 0.4379373 | 30.36 | 31.48 | 0.96 |
| 1 | m_tnfr2Pathway | [TNFR2 Signaling Pathway](http://cgap.nci.nih.gov/Pathways/BioCarta/m_tnfr2Pathway) | [Dusp1](http://www.ncbi.nlm.nih.gov/entrez/query.fcgi?cmd=search&db=gene&term=Dusp1) | dual specificity phosphatase 1 | < 1e-07 | 1513.19 | 673.69 | 2.25 |
| 2 |  | [TNFR2 Signaling Pathway](http://cgap.nci.nih.gov/Pathways/BioCarta/m_tnfr2Pathway) | [Nfkbia](http://www.ncbi.nlm.nih.gov/entrez/query.fcgi?cmd=search&db=gene&term=Nfkbia) | nuclear factor of kappa light polypeptide gene enhancer in B-cells inhibitor, alpha | 0.0002631 | 1140.45 | 852.99 | 1.34 |
| 3 |  | [TNFR2 Signaling Pathway](http://cgap.nci.nih.gov/Pathways/BioCarta/m_tnfr2Pathway) | [Nfkb1](http://www.ncbi.nlm.nih.gov/entrez/query.fcgi?cmd=search&db=gene&term=Nfkb1) | nuclear factor of kappa light polypeptide gene enhancer in B-cells 1, p105 | 0.0002841 | 636.36 | 804.29 | 0.79 |
| 4 |  | [TNFR2 Signaling Pathway](http://cgap.nci.nih.gov/Pathways/BioCarta/m_tnfr2Pathway) | [Tnfaip3](http://www.ncbi.nlm.nih.gov/entrez/query.fcgi?cmd=search&db=gene&term=Tnfaip3) | tumor necrosis factor, alpha-induced protein 3 | 0.0079502 | 70.87 | 86.44 | 0.82 |
| 5 |  | [TNFR2 Signaling Pathway](http://cgap.nci.nih.gov/Pathways/BioCarta/m_tnfr2Pathway) | [Ikbkap](http://www.ncbi.nlm.nih.gov/entrez/query.fcgi?cmd=search&db=gene&term=Ikbkap) | inhibitor of kappa light polypeptide enhancer in B-cells, kinase complex-associated protein | 0.5405129 | 76.65 | 83.19 | 0.92 |
| 1 | m_tcytotoxicPathway | [T Cytotoxic Cell Surface Molecules](http://cgap.nci.nih.gov/Pathways/BioCarta/m_tcytotoxicPathway) | [Cd3d](http://www.ncbi.nlm.nih.gov/entrez/query.fcgi?cmd=search&db=gene&term=Cd3d) | CD3 antigen, delta polypeptide | 1.3e-05 | 154.41 | 106.54 | 1.45 |
| 2 |  | [T Cytotoxic Cell Surface Molecules](http://cgap.nci.nih.gov/Pathways/BioCarta/m_tcytotoxicPathway) | [Cd3g](http://www.ncbi.nlm.nih.gov/entrez/query.fcgi?cmd=search&db=gene&term=Cd3g) | CD3 antigen, gamma polypeptide | 5.02e-05 | 250.04 | 181.14 | 1.38 |
| 3 |  | [T Cytotoxic Cell Surface Molecules](http://cgap.nci.nih.gov/Pathways/BioCarta/m_tcytotoxicPathway) | [Ptprc](http://www.ncbi.nlm.nih.gov/entrez/query.fcgi?cmd=search&db=gene&term=Ptprc) | protein tyrosine phosphatase, receptor type, C | 0.0001698 | 219.5 | 166.65 | 1.32 |
| 4 |  | [T Cytotoxic Cell Surface Molecules](http://cgap.nci.nih.gov/Pathways/BioCarta/m_tcytotoxicPathway) | [Thy1](http://www.ncbi.nlm.nih.gov/entrez/query.fcgi?cmd=search&db=gene&term=Thy1) | thymus cell antigen 1, theta | 0.0002318 | 1032.93 | 1238.08 | 0.83 |
| 5 |  | [T Cytotoxic Cell Surface Molecules](http://cgap.nci.nih.gov/Pathways/BioCarta/m_tcytotoxicPathway) | [Icam1](http://www.ncbi.nlm.nih.gov/entrez/query.fcgi?cmd=search&db=gene&term=Icam1) | intercellular adhesion molecule 1 | 0.000258 | 161.75 | 131.2 | 1.23 |
| 6 |  | [T Cytotoxic Cell Surface Molecules](http://cgap.nci.nih.gov/Pathways/BioCarta/m_tcytotoxicPathway) | [Tcra](http://www.ncbi.nlm.nih.gov/entrez/query.fcgi?cmd=search&db=gene&term=Tcra) | T-cell receptor alpha chain | 0.0007768 | 29.96 | 24.36 | 1.23 |
| 7 |  | [T Cytotoxic Cell Surface Molecules](http://cgap.nci.nih.gov/Pathways/BioCarta/m_tcytotoxicPathway) | [Itgb2](http://www.ncbi.nlm.nih.gov/entrez/query.fcgi?cmd=search&db=gene&term=Itgb2) | integrin beta 2 | 0.2003852 | 63.72 | 59.67 | 1.07 |
| 8 |  | [T Cytotoxic Cell Surface Molecules](http://cgap.nci.nih.gov/Pathways/BioCarta/m_tcytotoxicPathway) | [Cd247](http://www.ncbi.nlm.nih.gov/entrez/query.fcgi?cmd=search&db=gene&term=Cd247) | CD247 antigen | 0.50389 | 12.94 | 13.92 | 0.93 |
| 1 | m_pdgfPathway | [PDGF Signaling Pathway](http://cgap.nci.nih.gov/Pathways/BioCarta/m_pdgfPathway) | [Fos](http://www.ncbi.nlm.nih.gov/entrez/query.fcgi?cmd=search&db=gene&term=Fos) | FBJ osteosarcoma oncogene | < 1e-07 | 1804.66 | 294.09 | 6.14 |
| 2 |  | [PDGF Signaling Pathway](http://cgap.nci.nih.gov/Pathways/BioCarta/m_pdgfPathway) | [Stat1](http://www.ncbi.nlm.nih.gov/entrez/query.fcgi?cmd=search&db=gene&term=Stat1) | signal transducer and activator of transcription 1 | < 1e-07 | 804.27 | 394.42 | 2.04 |
| 3 |  | [PDGF Signaling Pathway](http://cgap.nci.nih.gov/Pathways/BioCarta/m_pdgfPathway) | [Map2k4](http://www.ncbi.nlm.nih.gov/entrez/query.fcgi?cmd=search&db=gene&term=Map2k4) | mitogen-activated protein kinase kinase 4 | 9.41e-05 | 341.42 | 495.55 | 0.69 |
| 4 |  | [PDGF Signaling Pathway](http://cgap.nci.nih.gov/Pathways/BioCarta/m_pdgfPathway) | [Prkcb](http://www.ncbi.nlm.nih.gov/entrez/query.fcgi?cmd=search&db=gene&term=Prkcb) | protein kinase C, beta | 0.000107 | 173.52 | 135.08 | 1.28 |
| 5 |  | [PDGF Signaling Pathway](http://cgap.nci.nih.gov/Pathways/BioCarta/m_pdgfPathway) | [Jun](http://www.ncbi.nlm.nih.gov/entrez/query.fcgi?cmd=search&db=gene&term=Jun) | Jun oncogene | 0.0005914 | 270.82 | 205.78 | 1.32 |
| 6 |  | [PDGF Signaling Pathway](http://cgap.nci.nih.gov/Pathways/BioCarta/m_pdgfPathway) | [Sos1](http://www.ncbi.nlm.nih.gov/entrez/query.fcgi?cmd=search&db=gene&term=Sos1) | son of sevenless homolog 1 (Drosophila) | 0.0052437 | 62.98 | 76.11 | 0.83 |
| 7 |  | [PDGF Signaling Pathway](http://cgap.nci.nih.gov/Pathways/BioCarta/m_pdgfPathway) | [Csnk2a2](http://www.ncbi.nlm.nih.gov/entrez/query.fcgi?cmd=search&db=gene&term=Csnk2a2) | casein kinase 2, alpha prime polypeptide | 0.0102407 | 1559.16 | 1719.9 | 0.91 |
| 8 |  | [PDGF Signaling Pathway](http://cgap.nci.nih.gov/Pathways/BioCarta/m_pdgfPathway) | [Stat5a](http://www.ncbi.nlm.nih.gov/entrez/query.fcgi?cmd=search&db=gene&term=Stat5a) | signal transducer and activator of transcription 5A | 0.0353627 | 63.97 | 73.88 | 0.87 |
| 9 |  | [PDGF Signaling Pathway](http://cgap.nci.nih.gov/Pathways/BioCarta/m_pdgfPathway) | [Mapk8](http://www.ncbi.nlm.nih.gov/entrez/query.fcgi?cmd=search&db=gene&term=Mapk8) | mitogen-activated protein kinase 8 | 0.0431864 | 31.06 | 25.43 | 1.22 |
| 10 |  | [PDGF Signaling Pathway](http://cgap.nci.nih.gov/Pathways/BioCarta/m_pdgfPathway) | [Jak1](http://www.ncbi.nlm.nih.gov/entrez/query.fcgi?cmd=search&db=gene&term=Jak1) | Janus kinase 1 | 0.1036342 | 128.89 | 156.56 | 0.82 |
| 11 |  | [PDGF Signaling Pathway](http://cgap.nci.nih.gov/Pathways/BioCarta/m_pdgfPathway) | [Pik3cg](http://www.ncbi.nlm.nih.gov/entrez/query.fcgi?cmd=search&db=gene&term=Pik3cg) | phosphoinositide-3-kinase, catalytic, gamma polypeptide | 0.3272953 | 16.25 | 14.76 | 1.1 |
| 12 |  | [PDGF Signaling Pathway](http://cgap.nci.nih.gov/Pathways/BioCarta/m_pdgfPathway) | [Csnk2a1](http://www.ncbi.nlm.nih.gov/entrez/query.fcgi?cmd=search&db=gene&term=Csnk2a1) | casein kinase 2, alpha 1 polypeptide | 0.5759663 | 255.89 | 272.9 | 0.94 |
| 13 |  | [PDGF Signaling Pathway](http://cgap.nci.nih.gov/Pathways/BioCarta/m_pdgfPathway) | [Pik3r1](http://www.ncbi.nlm.nih.gov/entrez/query.fcgi?cmd=search&db=gene&term=Pik3r1) | phosphatidylinositol 3-kinase, regulatory subunit, polypeptide 1 (p85 alpha) | 0.6503694 | 113.16 | 110.05 | 1.03 |
| 1 | m_dspPathway | [Regulation of MAP Kinase Pathways Through Dual Specificity Phosphatases](http://cgap.nci.nih.gov/Pathways/BioCarta/m_dspPathway) | [Dusp1](http://www.ncbi.nlm.nih.gov/entrez/query.fcgi?cmd=search&db=gene&term=Dusp1) | dual specificity phosphatase 1 | < 1e-07 | 1513.19 | 673.69 | 2.25 |
| 2 |  | [Regulation of MAP Kinase Pathways Through Dual Specificity Phosphatases](http://cgap.nci.nih.gov/Pathways/BioCarta/m_dspPathway) | [Dusp2](http://www.ncbi.nlm.nih.gov/entrez/query.fcgi?cmd=search&db=gene&term=Dusp2) | dual specificity phosphatase 2 | 0.0001431 | 42.1 | 33.25 | 1.27 |
| 3 |  | [Regulation of MAP Kinase Pathways Through Dual Specificity Phosphatases](http://cgap.nci.nih.gov/Pathways/BioCarta/m_dspPathway) | [Dusp6](http://www.ncbi.nlm.nih.gov/entrez/query.fcgi?cmd=search&db=gene&term=Dusp6) | dual specificity phosphatase 6 | 0.0001618 | 219.29 | 266.43 | 0.82 |
| 4 |  | [Regulation of MAP Kinase Pathways Through Dual Specificity Phosphatases](http://cgap.nci.nih.gov/Pathways/BioCarta/m_dspPathway) | [Mapk14](http://www.ncbi.nlm.nih.gov/entrez/query.fcgi?cmd=search&db=gene&term=Mapk14) | mitogen-activated protein kinase 14 | 0.0123923 | 55 | 71.64 | 0.77 |
| 5 |  | [Regulation of MAP Kinase Pathways Through Dual Specificity Phosphatases](http://cgap.nci.nih.gov/Pathways/BioCarta/m_dspPathway) | [Mapk8](http://www.ncbi.nlm.nih.gov/entrez/query.fcgi?cmd=search&db=gene&term=Mapk8) | mitogen-activated protein kinase 8 | 0.0431864 | 31.06 | 25.43 | 1.22 |
| 6 |  | [Regulation of MAP Kinase Pathways Through Dual Specificity Phosphatases](http://cgap.nci.nih.gov/Pathways/BioCarta/m_dspPathway) | [Dusp9](http://www.ncbi.nlm.nih.gov/entrez/query.fcgi?cmd=search&db=gene&term=Dusp9) | dual specificity phosphatase 9 | 0.2460248 | 9.25 | 10.13 | 0.91 |
| 1 | m_il3Pathway | [IL 3 signaling pathway](http://cgap.nci.nih.gov/Pathways/BioCarta/m_il3Pathway) | [Fos](http://www.ncbi.nlm.nih.gov/entrez/query.fcgi?cmd=search&db=gene&term=Fos) | FBJ osteosarcoma oncogene | < 1e-07 | 1804.66 | 294.09 | 6.14 |
| 2 |  | [IL 3 signaling pathway](http://cgap.nci.nih.gov/Pathways/BioCarta/m_il3Pathway) | [Il3ra](http://www.ncbi.nlm.nih.gov/entrez/query.fcgi?cmd=search&db=gene&term=Il3ra) | interleukin 3 receptor, alpha chain | 4.36e-05 | 45.2 | 34.37 | 1.32 |
| 3 |  | [IL 3 signaling pathway](http://cgap.nci.nih.gov/Pathways/BioCarta/m_il3Pathway) | [Sos1](http://www.ncbi.nlm.nih.gov/entrez/query.fcgi?cmd=search&db=gene&term=Sos1) | son of sevenless homolog 1 (Drosophila) | 0.0052437 | 62.98 | 76.11 | 0.83 |
| 4 |  | [IL 3 signaling pathway](http://cgap.nci.nih.gov/Pathways/BioCarta/m_il3Pathway) | [Stat5a](http://www.ncbi.nlm.nih.gov/entrez/query.fcgi?cmd=search&db=gene&term=Stat5a) | signal transducer and activator of transcription 5A | 0.0353627 | 63.97 | 73.88 | 0.87 |
| 5 |  | [IL 3 signaling pathway](http://cgap.nci.nih.gov/Pathways/BioCarta/m_il3Pathway) | [Csf2rb](http://www.ncbi.nlm.nih.gov/entrez/query.fcgi?cmd=search&db=gene&term=Csf2rb) | colony stimulating factor 2 receptor, beta, low-affinity (granulocyte-macrophage) | 0.2247125 | 37.7 | 42.88 | 0.88 |
| 1 | m_gleevecpathway | [Inhibition of Cellular Proliferation by Gleevec](http://cgap.nci.nih.gov/Pathways/BioCarta/m_gleevecpathway) | [Fos](http://www.ncbi.nlm.nih.gov/entrez/query.fcgi?cmd=search&db=gene&term=Fos) | FBJ osteosarcoma oncogene | < 1e-07 | 1804.66 | 294.09 | 6.14 |
| 2 |  | [Inhibition of Cellular Proliferation by Gleevec](http://cgap.nci.nih.gov/Pathways/BioCarta/m_gleevecpathway) | [Stat1](http://www.ncbi.nlm.nih.gov/entrez/query.fcgi?cmd=search&db=gene&term=Stat1) | signal transducer and activator of transcription 1 | < 1e-07 | 804.27 | 394.42 | 2.04 |
| 3 |  | [Inhibition of Cellular Proliferation by Gleevec](http://cgap.nci.nih.gov/Pathways/BioCarta/m_gleevecpathway) | [Map2k4](http://www.ncbi.nlm.nih.gov/entrez/query.fcgi?cmd=search&db=gene&term=Map2k4) | mitogen-activated protein kinase kinase 4 | 9.41e-05 | 341.42 | 495.55 | 0.69 |
| 4 |  | [Inhibition of Cellular Proliferation by Gleevec](http://cgap.nci.nih.gov/Pathways/BioCarta/m_gleevecpathway) | [Jun](http://www.ncbi.nlm.nih.gov/entrez/query.fcgi?cmd=search&db=gene&term=Jun) | Jun oncogene | 0.0005914 | 270.82 | 205.78 | 1.32 |
| 5 |  | [Inhibition of Cellular Proliferation by Gleevec](http://cgap.nci.nih.gov/Pathways/BioCarta/m_gleevecpathway) | [Sos1](http://www.ncbi.nlm.nih.gov/entrez/query.fcgi?cmd=search&db=gene&term=Sos1) | son of sevenless homolog 1 (Drosophila) | 0.0052437 | 62.98 | 76.11 | 0.83 |
| 6 |  | [Inhibition of Cellular Proliferation by Gleevec](http://cgap.nci.nih.gov/Pathways/BioCarta/m_gleevecpathway) | [Bcr](http://www.ncbi.nlm.nih.gov/entrez/query.fcgi?cmd=search&db=gene&term=Bcr) | breakpoint cluster region | 0.0090594 | 189.32 | 223.1 | 0.85 |
| 7 |  | [Inhibition of Cellular Proliferation by Gleevec](http://cgap.nci.nih.gov/Pathways/BioCarta/m_gleevecpathway) | [Stat5a](http://www.ncbi.nlm.nih.gov/entrez/query.fcgi?cmd=search&db=gene&term=Stat5a) | signal transducer and activator of transcription 5A | 0.0353627 | 63.97 | 73.88 | 0.87 |
| 8 |  | [Inhibition of Cellular Proliferation by Gleevec](http://cgap.nci.nih.gov/Pathways/BioCarta/m_gleevecpathway) | [Mapk8](http://www.ncbi.nlm.nih.gov/entrez/query.fcgi?cmd=search&db=gene&term=Mapk8) | mitogen-activated protein kinase 8 | 0.0431864 | 31.06 | 25.43 | 1.22 |
| 9 |  | [Inhibition of Cellular Proliferation by Gleevec](http://cgap.nci.nih.gov/Pathways/BioCarta/m_gleevecpathway) | [Pik3cg](http://www.ncbi.nlm.nih.gov/entrez/query.fcgi?cmd=search&db=gene&term=Pik3cg) | phosphoinositide-3-kinase, catalytic, gamma polypeptide | 0.3272953 | 16.25 | 14.76 | 1.1 |
| 10 |  | [Inhibition of Cellular Proliferation by Gleevec](http://cgap.nci.nih.gov/Pathways/BioCarta/m_gleevecpathway) | [Pik3r1](http://www.ncbi.nlm.nih.gov/entrez/query.fcgi?cmd=search&db=gene&term=Pik3r1) | phosphatidylinositol 3-kinase, regulatory subunit, polypeptide 1 (p85 alpha) | 0.6503694 | 113.16 | 110.05 | 1.03 |
| 11 |  | [Inhibition of Cellular Proliferation by Gleevec](http://cgap.nci.nih.gov/Pathways/BioCarta/m_gleevecpathway) | [Crkl](http://www.ncbi.nlm.nih.gov/entrez/query.fcgi?cmd=search&db=gene&term=Crkl) | v-crk sarcoma virus CT10 oncogene homolog (avian)-like | 0.9375339 | 19.66 | 19.51 | 1.01 |
| 1 | m_TPOPathway | [TPO Signaling Pathway](http://cgap.nci.nih.gov/Pathways/BioCarta/m_TPOPathway) | [Fos](http://www.ncbi.nlm.nih.gov/entrez/query.fcgi?cmd=search&db=gene&term=Fos) | FBJ osteosarcoma oncogene | < 1e-07 | 1804.66 | 294.09 | 6.14 |
| 2 |  | [TPO Signaling Pathway](http://cgap.nci.nih.gov/Pathways/BioCarta/m_TPOPathway) | [Stat1](http://www.ncbi.nlm.nih.gov/entrez/query.fcgi?cmd=search&db=gene&term=Stat1) | signal transducer and activator of transcription 1 | < 1e-07 | 804.27 | 394.42 | 2.04 |
| 3 |  | [TPO Signaling Pathway](http://cgap.nci.nih.gov/Pathways/BioCarta/m_TPOPathway) | [Prkcb](http://www.ncbi.nlm.nih.gov/entrez/query.fcgi?cmd=search&db=gene&term=Prkcb) | protein kinase C, beta | 0.000107 | 173.52 | 135.08 | 1.28 |
| 4 |  | [TPO Signaling Pathway](http://cgap.nci.nih.gov/Pathways/BioCarta/m_TPOPathway) | [Jun](http://www.ncbi.nlm.nih.gov/entrez/query.fcgi?cmd=search&db=gene&term=Jun) | Jun oncogene | 0.0005914 | 270.82 | 205.78 | 1.32 |
| 5 |  | [TPO Signaling Pathway](http://cgap.nci.nih.gov/Pathways/BioCarta/m_TPOPathway) | [Sos1](http://www.ncbi.nlm.nih.gov/entrez/query.fcgi?cmd=search&db=gene&term=Sos1) | son of sevenless homolog 1 (Drosophila) | 0.0052437 | 62.98 | 76.11 | 0.83 |
| 6 |  | [TPO Signaling Pathway](http://cgap.nci.nih.gov/Pathways/BioCarta/m_TPOPathway) | [Csnk2a2](http://www.ncbi.nlm.nih.gov/entrez/query.fcgi?cmd=search&db=gene&term=Csnk2a2) | casein kinase 2, alpha prime polypeptide | 0.0102407 | 1559.16 | 1719.9 | 0.91 |
| 7 |  | [TPO Signaling Pathway](http://cgap.nci.nih.gov/Pathways/BioCarta/m_TPOPathway) | [Stat5a](http://www.ncbi.nlm.nih.gov/entrez/query.fcgi?cmd=search&db=gene&term=Stat5a) | signal transducer and activator of transcription 5A | 0.0353627 | 63.97 | 73.88 | 0.87 |
| 8 |  | [TPO Signaling Pathway](http://cgap.nci.nih.gov/Pathways/BioCarta/m_TPOPathway) | [Mpl](http://www.ncbi.nlm.nih.gov/entrez/query.fcgi?cmd=search&db=gene&term=Mpl) | myeloproliferative leukemia virus oncogene | 0.2130524 | 22.39 | 20.1 | 1.11 |
| 9 |  | [TPO Signaling Pathway](http://cgap.nci.nih.gov/Pathways/BioCarta/m_TPOPathway) | [Pik3cg](http://www.ncbi.nlm.nih.gov/entrez/query.fcgi?cmd=search&db=gene&term=Pik3cg) | phosphoinositide-3-kinase, catalytic, gamma polypeptide | 0.3272953 | 16.25 | 14.76 | 1.1 |
| 10 |  | [TPO Signaling Pathway](http://cgap.nci.nih.gov/Pathways/BioCarta/m_TPOPathway) | [Csnk2a1](http://www.ncbi.nlm.nih.gov/entrez/query.fcgi?cmd=search&db=gene&term=Csnk2a1) | casein kinase 2, alpha 1 polypeptide | 0.5759663 | 255.89 | 272.9 | 0.94 |
| 11 |  | [TPO Signaling Pathway](http://cgap.nci.nih.gov/Pathways/BioCarta/m_TPOPathway) | [Pik3r1](http://www.ncbi.nlm.nih.gov/entrez/query.fcgi?cmd=search&db=gene&term=Pik3r1) | phosphatidylinositol 3-kinase, regulatory subunit, polypeptide 1 (p85 alpha) | 0.6503694 | 113.16 | 110.05 | 1.03 |
| 1 | m_CSKPathway | [Activation of Csk by cAMP-dependent Protein Kinase Inhibits Signaling through the T Cell Receptor](http://cgap.nci.nih.gov/Pathways/BioCarta/m_CSKPathway) | [Cd3d](http://www.ncbi.nlm.nih.gov/entrez/query.fcgi?cmd=search&db=gene&term=Cd3d) | CD3 antigen, delta polypeptide | 1.3e-05 | 154.41 | 106.54 | 1.45 |
| 2 |  | [Activation of Csk by cAMP-dependent Protein Kinase Inhibits Signaling through the T Cell Receptor](http://cgap.nci.nih.gov/Pathways/BioCarta/m_CSKPathway) | [Cd3g](http://www.ncbi.nlm.nih.gov/entrez/query.fcgi?cmd=search&db=gene&term=Cd3g) | CD3 antigen, gamma polypeptide | 5.02e-05 | 250.04 | 181.14 | 1.38 |
| 3 |  | [Activation of Csk by cAMP-dependent Protein Kinase Inhibits Signaling through the T Cell Receptor](http://cgap.nci.nih.gov/Pathways/BioCarta/m_CSKPathway) | [Ptprc](http://www.ncbi.nlm.nih.gov/entrez/query.fcgi?cmd=search&db=gene&term=Ptprc) | protein tyrosine phosphatase, receptor type, C | 0.0001698 | 219.5 | 166.65 | 1.32 |
| 4 |  | [Activation of Csk by cAMP-dependent Protein Kinase Inhibits Signaling through the T Cell Receptor](http://cgap.nci.nih.gov/Pathways/BioCarta/m_CSKPathway) | [Prkacb](http://www.ncbi.nlm.nih.gov/entrez/query.fcgi?cmd=search&db=gene&term=Prkacb) | protein kinase, cAMP dependent, catalytic, beta | 0.0006388 | 238.56 | 190.67 | 1.25 |
| 5 |  | [Activation of Csk by cAMP-dependent Protein Kinase Inhibits Signaling through the T Cell Receptor](http://cgap.nci.nih.gov/Pathways/BioCarta/m_CSKPathway) | [Tcra](http://www.ncbi.nlm.nih.gov/entrez/query.fcgi?cmd=search&db=gene&term=Tcra) | T-cell receptor alpha chain | 0.0007768 | 29.96 | 24.36 | 1.23 |
| 6 |  | [Activation of Csk by cAMP-dependent Protein Kinase Inhibits Signaling through the T Cell Receptor](http://cgap.nci.nih.gov/Pathways/BioCarta/m_CSKPathway) | [Zap70](http://www.ncbi.nlm.nih.gov/entrez/query.fcgi?cmd=search&db=gene&term=Zap70) | zeta-chain (TCR) associated protein kinase | 0.001192 | 540.43 | 424.72 | 1.27 |
| 7 |  | [Activation of Csk by cAMP-dependent Protein Kinase Inhibits Signaling through the T Cell Receptor](http://cgap.nci.nih.gov/Pathways/BioCarta/m_CSKPathway) | [Lck](http://www.ncbi.nlm.nih.gov/entrez/query.fcgi?cmd=search&db=gene&term=Lck) | lymphocyte protein tyrosine kinase | 0.0016033 | 90.08 | 69.9 | 1.29 |
| 8 |  | [Activation of Csk by cAMP-dependent Protein Kinase Inhibits Signaling through the T Cell Receptor](http://cgap.nci.nih.gov/Pathways/BioCarta/m_CSKPathway) | [Cd247](http://www.ncbi.nlm.nih.gov/entrez/query.fcgi?cmd=search&db=gene&term=Cd247) | CD247 antigen | 0.50389 | 12.94 | 13.92 | 0.93 |
| 9 |  | [Activation of Csk by cAMP-dependent Protein Kinase Inhibits Signaling through the T Cell Receptor](http://cgap.nci.nih.gov/Pathways/BioCarta/m_CSKPathway) | [Cd4](http://www.ncbi.nlm.nih.gov/entrez/query.fcgi?cmd=search&db=gene&term=Cd4) | CD4 antigen | 0.9710901 | 18.59 | 18.53 | 1 |
| 1 | m_arenrf2Pathway | [Oxidative Stress Induced Gene Expression Via Nrf2](http://cgap.nci.nih.gov/Pathways/BioCarta/m_arenrf2Pathway) | [Fos](http://www.ncbi.nlm.nih.gov/entrez/query.fcgi?cmd=search&db=gene&term=Fos) | FBJ osteosarcoma oncogene | < 1e-07 | 1804.66 | 294.09 | 6.14 |
| 2 |  | [Oxidative Stress Induced Gene Expression Via Nrf2](http://cgap.nci.nih.gov/Pathways/BioCarta/m_arenrf2Pathway) | [Gsta2](http://www.ncbi.nlm.nih.gov/entrez/query.fcgi?cmd=search&db=gene&term=Gsta2) | glutathione S-transferase, alpha 2 (Yc2) | 6.69e-05 | 549.61 | 402.79 | 1.36 |
| 3 |  | [Oxidative Stress Induced Gene Expression Via Nrf2](http://cgap.nci.nih.gov/Pathways/BioCarta/m_arenrf2Pathway) | [Prkcb](http://www.ncbi.nlm.nih.gov/entrez/query.fcgi?cmd=search&db=gene&term=Prkcb) | protein kinase C, beta | 0.000107 | 173.52 | 135.08 | 1.28 |
| 4 |  | [Oxidative Stress Induced Gene Expression Via Nrf2](http://cgap.nci.nih.gov/Pathways/BioCarta/m_arenrf2Pathway) | [Jun](http://www.ncbi.nlm.nih.gov/entrez/query.fcgi?cmd=search&db=gene&term=Jun) | Jun oncogene | 0.0005914 | 270.82 | 205.78 | 1.32 |
| 5 |  | [Oxidative Stress Induced Gene Expression Via Nrf2](http://cgap.nci.nih.gov/Pathways/BioCarta/m_arenrf2Pathway) | [Mapk14](http://www.ncbi.nlm.nih.gov/entrez/query.fcgi?cmd=search&db=gene&term=Mapk14) | mitogen-activated protein kinase 14 | 0.0123923 | 55 | 71.64 | 0.77 |
| 6 |  | [Oxidative Stress Induced Gene Expression Via Nrf2](http://cgap.nci.nih.gov/Pathways/BioCarta/m_arenrf2Pathway) | [Mapk8](http://www.ncbi.nlm.nih.gov/entrez/query.fcgi?cmd=search&db=gene&term=Mapk8) | mitogen-activated protein kinase 8 | 0.0431864 | 31.06 | 25.43 | 1.22 |
| 7 |  | [Oxidative Stress Induced Gene Expression Via Nrf2](http://cgap.nci.nih.gov/Pathways/BioCarta/m_arenrf2Pathway) | [Creb1](http://www.ncbi.nlm.nih.gov/entrez/query.fcgi?cmd=search&db=gene&term=Creb1) | cAMP responsive element binding protein 1 | 0.7553857 | 48.38 | 50.82 | 0.95 |
| 8 |  | [Oxidative Stress Induced Gene Expression Via Nrf2](http://cgap.nci.nih.gov/Pathways/BioCarta/m_arenrf2Pathway) | [Mapk1](http://www.ncbi.nlm.nih.gov/entrez/query.fcgi?cmd=search&db=gene&term=Mapk1) | mitogen-activated protein kinase 1 | 0.9818276 | 246.1 | 247.01 | 1 |
| 1 | m_cdk5Pathway | [Phosphorylation of MEK1 by cdk5/p35 down regulates the MAP kinase pathway](http://cgap.nci.nih.gov/Pathways/BioCarta/m_cdk5Pathway) | [Egr1](http://www.ncbi.nlm.nih.gov/entrez/query.fcgi?cmd=search&db=gene&term=Egr1) | early growth response 1 | 2.7e-06 | 2008.88 | 1401.3 | 1.43 |
| 2 |  | [Phosphorylation of MEK1 by cdk5/p35 down regulates the MAP kinase pathway](http://cgap.nci.nih.gov/Pathways/BioCarta/m_cdk5Pathway) | [Ngfr](http://www.ncbi.nlm.nih.gov/entrez/query.fcgi?cmd=search&db=gene&term=Ngfr) | nerve growth factor receptor (TNFR superfamily, member 16) | 0.000162 | 230.03 | 189.19 | 1.22 |
| 3 |  | [Phosphorylation of MEK1 by cdk5/p35 down regulates the MAP kinase pathway](http://cgap.nci.nih.gov/Pathways/BioCarta/m_cdk5Pathway) | [Ngf](http://www.ncbi.nlm.nih.gov/entrez/query.fcgi?cmd=search&db=gene&term=Ngf) | nerve growth factor | 0.0001997 | 56.89 | 72.78 | 0.78 |
| 4 |  | [Phosphorylation of MEK1 by cdk5/p35 down regulates the MAP kinase pathway](http://cgap.nci.nih.gov/Pathways/BioCarta/m_cdk5Pathway) | [Cdk5r1](http://www.ncbi.nlm.nih.gov/entrez/query.fcgi?cmd=search&db=gene&term=Cdk5r1) | cyclin-dependent kinase 5, regulatory subunit 1 (p35) | 0.0013398 | 50.41 | 40.71 | 1.24 |
| 5 |  | [Phosphorylation of MEK1 by cdk5/p35 down regulates the MAP kinase pathway](http://cgap.nci.nih.gov/Pathways/BioCarta/m_cdk5Pathway) | [Mapk1](http://www.ncbi.nlm.nih.gov/entrez/query.fcgi?cmd=search&db=gene&term=Mapk1) | mitogen-activated protein kinase 1 | 0.9818276 | 246.1 | 247.01 | 1 |
| 1 | m_ifnaPathway | [IFN alpha signaling pathway](http://cgap.nci.nih.gov/Pathways/BioCarta/m_ifnaPathway) | [Stat1](http://www.ncbi.nlm.nih.gov/entrez/query.fcgi?cmd=search&db=gene&term=Stat1) | signal transducer and activator of transcription 1 | < 1e-07 | 804.27 | 394.42 | 2.04 |
| 2 |  | [IFN alpha signaling pathway](http://cgap.nci.nih.gov/Pathways/BioCarta/m_ifnaPathway) | [Irf9](http://www.ncbi.nlm.nih.gov/entrez/query.fcgi?cmd=search&db=gene&term=Irf9) | interferon regulatory factor 9 | 8.72e-05 | 469.83 | 332.72 | 1.41 |
| 3 |  | [IFN alpha signaling pathway](http://cgap.nci.nih.gov/Pathways/BioCarta/m_ifnaPathway) | [Stat2](http://www.ncbi.nlm.nih.gov/entrez/query.fcgi?cmd=search&db=gene&term=Stat2) | signal transducer and activator of transcription 2 | 0.0005157 | 95.25 | 66.23 | 1.44 |
| 4 |  | [IFN alpha signaling pathway](http://cgap.nci.nih.gov/Pathways/BioCarta/m_ifnaPathway) | [Jak1](http://www.ncbi.nlm.nih.gov/entrez/query.fcgi?cmd=search&db=gene&term=Jak1) | Janus kinase 1 | 0.1036342 | 128.89 | 156.56 | 0.82 |
| 5 |  | [IFN alpha signaling pathway](http://cgap.nci.nih.gov/Pathways/BioCarta/m_ifnaPathway) | [Ifnb1](http://www.ncbi.nlm.nih.gov/entrez/query.fcgi?cmd=search&db=gene&term=Ifnb1) | interferon beta 1, fibroblast | 0.5018009 | 22.87 | 21.44 | 1.07 |
| 1 | m_gpcrPathway | [Signaling Pathway from G-Protein Families](http://cgap.nci.nih.gov/Pathways/BioCarta/m_gpcrPathway) | [Fos](http://www.ncbi.nlm.nih.gov/entrez/query.fcgi?cmd=search&db=gene&term=Fos) | FBJ osteosarcoma oncogene | < 1e-07 | 1804.66 | 294.09 | 6.14 |
| 2 |  | [Signaling Pathway from G-Protein Families](http://cgap.nci.nih.gov/Pathways/BioCarta/m_gpcrPathway) | [Prkcb](http://www.ncbi.nlm.nih.gov/entrez/query.fcgi?cmd=search&db=gene&term=Prkcb) | protein kinase C, beta | 0.000107 | 173.52 | 135.08 | 1.28 |
| 3 |  | [Signaling Pathway from G-Protein Families](http://cgap.nci.nih.gov/Pathways/BioCarta/m_gpcrPathway) | [Jun](http://www.ncbi.nlm.nih.gov/entrez/query.fcgi?cmd=search&db=gene&term=Jun) | Jun oncogene | 0.0005914 | 270.82 | 205.78 | 1.32 |
| 4 |  | [Signaling Pathway from G-Protein Families](http://cgap.nci.nih.gov/Pathways/BioCarta/m_gpcrPathway) | [Prkacb](http://www.ncbi.nlm.nih.gov/entrez/query.fcgi?cmd=search&db=gene&term=Prkacb) | protein kinase, cAMP dependent, catalytic, beta | 0.0006388 | 238.56 | 190.67 | 1.25 |
| 5 |  | [Signaling Pathway from G-Protein Families](http://cgap.nci.nih.gov/Pathways/BioCarta/m_gpcrPathway) | [Rps6ka3](http://www.ncbi.nlm.nih.gov/entrez/query.fcgi?cmd=search&db=gene&term=Rps6ka3) | ribosomal protein S6 kinase polypeptide 3 | 0.0125409 | 26.26 | 31.84 | 0.82 |
| 6 |  | [Signaling Pathway from G-Protein Families](http://cgap.nci.nih.gov/Pathways/BioCarta/m_gpcrPathway) | [Nfatc2](http://www.ncbi.nlm.nih.gov/entrez/query.fcgi?cmd=search&db=gene&term=Nfatc2) | nuclear factor of activated T-cells, cytoplasmic, calcineurin-dependent 2 | 0.0683684 | 14.9 | 16.65 | 0.9 |
| 7 |  | [Signaling Pathway from G-Protein Families](http://cgap.nci.nih.gov/Pathways/BioCarta/m_gpcrPathway) | [Nfatc3](http://www.ncbi.nlm.nih.gov/entrez/query.fcgi?cmd=search&db=gene&term=Nfatc3) | nuclear factor of activated T-cells, cytoplasmic, calcineurin-dependent 3 | 0.1778903 | 211.8 | 241.23 | 0.88 |
| 8 |  | [Signaling Pathway from G-Protein Families](http://cgap.nci.nih.gov/Pathways/BioCarta/m_gpcrPathway) | [Creb1](http://www.ncbi.nlm.nih.gov/entrez/query.fcgi?cmd=search&db=gene&term=Creb1) | cAMP responsive element binding protein 1 | 0.7553857 | 48.38 | 50.82 | 0.95 |
| 1 | m_thelperPathway | [T Helper Cell Surface Molecules](http://cgap.nci.nih.gov/Pathways/BioCarta/m_thelperPathway) | [Cd3d](http://www.ncbi.nlm.nih.gov/entrez/query.fcgi?cmd=search&db=gene&term=Cd3d) | CD3 antigen, delta polypeptide | 1.3e-05 | 154.41 | 106.54 | 1.45 |
| 2 |  | [T Helper Cell Surface Molecules](http://cgap.nci.nih.gov/Pathways/BioCarta/m_thelperPathway) | [Cd3g](http://www.ncbi.nlm.nih.gov/entrez/query.fcgi?cmd=search&db=gene&term=Cd3g) | CD3 antigen, gamma polypeptide | 5.02e-05 | 250.04 | 181.14 | 1.38 |
| 3 |  | [T Helper Cell Surface Molecules](http://cgap.nci.nih.gov/Pathways/BioCarta/m_thelperPathway) | [Ptprc](http://www.ncbi.nlm.nih.gov/entrez/query.fcgi?cmd=search&db=gene&term=Ptprc) | protein tyrosine phosphatase, receptor type, C | 0.0001698 | 219.5 | 166.65 | 1.32 |
| 4 |  | [T Helper Cell Surface Molecules](http://cgap.nci.nih.gov/Pathways/BioCarta/m_thelperPathway) | [Thy1](http://www.ncbi.nlm.nih.gov/entrez/query.fcgi?cmd=search&db=gene&term=Thy1) | thymus cell antigen 1, theta | 0.0002318 | 1032.93 | 1238.08 | 0.83 |
| 5 |  | [T Helper Cell Surface Molecules](http://cgap.nci.nih.gov/Pathways/BioCarta/m_thelperPathway) | [Icam1](http://www.ncbi.nlm.nih.gov/entrez/query.fcgi?cmd=search&db=gene&term=Icam1) | intercellular adhesion molecule 1 | 0.000258 | 161.75 | 131.2 | 1.23 |
| 6 |  | [T Helper Cell Surface Molecules](http://cgap.nci.nih.gov/Pathways/BioCarta/m_thelperPathway) | [Tcra](http://www.ncbi.nlm.nih.gov/entrez/query.fcgi?cmd=search&db=gene&term=Tcra) | T-cell receptor alpha chain | 0.0007768 | 29.96 | 24.36 | 1.23 |
| 7 |  | [T Helper Cell Surface Molecules](http://cgap.nci.nih.gov/Pathways/BioCarta/m_thelperPathway) | [Itgb2](http://www.ncbi.nlm.nih.gov/entrez/query.fcgi?cmd=search&db=gene&term=Itgb2) | integrin beta 2 | 0.2003852 | 63.72 | 59.67 | 1.07 |
| 8 |  | [T Helper Cell Surface Molecules](http://cgap.nci.nih.gov/Pathways/BioCarta/m_thelperPathway) | [Cd247](http://www.ncbi.nlm.nih.gov/entrez/query.fcgi?cmd=search&db=gene&term=Cd247) | CD247 antigen | 0.50389 | 12.94 | 13.92 | 0.93 |
| 9 |  | [T Helper Cell Surface Molecules](http://cgap.nci.nih.gov/Pathways/BioCarta/m_thelperPathway) | [Cd4](http://www.ncbi.nlm.nih.gov/entrez/query.fcgi?cmd=search&db=gene&term=Cd4) | CD4 antigen | 0.9710901 | 18.59 | 18.53 | 1 |
| 1 | m_g2Pathway | [Cell Cycle: G2/M Checkpoint](http://cgap.nci.nih.gov/Pathways/BioCarta/m_g2Pathway) | [Ccnb1](http://www.ncbi.nlm.nih.gov/entrez/query.fcgi?cmd=search&db=gene&term=Ccnb1) | cyclin B1 | 2.2e-06 | 132.48 | 194.83 | 0.68 |
| 2 |  | [Cell Cycle: G2/M Checkpoint](http://cgap.nci.nih.gov/Pathways/BioCarta/m_g2Pathway) | [Cdk1](http://www.ncbi.nlm.nih.gov/entrez/query.fcgi?cmd=search&db=gene&term=Cdk1) | cyclin-dependent kinase 1 | 4.79e-05 | 218.93 | 282.88 | 0.77 |
| 3 |  | [Cell Cycle: G2/M Checkpoint](http://cgap.nci.nih.gov/Pathways/BioCarta/m_g2Pathway) | [Plk1](http://www.ncbi.nlm.nih.gov/entrez/query.fcgi?cmd=search&db=gene&term=Plk1) | polo-like kinase 1 (Drosophila) | 0.0002232 | 169.96 | 212.11 | 0.8 |
| 4 |  | [Cell Cycle: G2/M Checkpoint](http://cgap.nci.nih.gov/Pathways/BioCarta/m_g2Pathway) | [Gadd45a](http://www.ncbi.nlm.nih.gov/entrez/query.fcgi?cmd=search&db=gene&term=Gadd45a) | growth arrest and DNA-damage-inducible 45 alpha | 0.001651 | 66.3 | 85.78 | 0.77 |
| 5 |  | [Cell Cycle: G2/M Checkpoint](http://cgap.nci.nih.gov/Pathways/BioCarta/m_g2Pathway) | [Trp53](http://www.ncbi.nlm.nih.gov/entrez/query.fcgi?cmd=search&db=gene&term=Trp53) | transformation related protein 53 | 0.0027856 | 11.53 | 14.37 | 0.8 |
| 6 |  | [Cell Cycle: G2/M Checkpoint](http://cgap.nci.nih.gov/Pathways/BioCarta/m_g2Pathway) | [Wee1](http://www.ncbi.nlm.nih.gov/entrez/query.fcgi?cmd=search&db=gene&term=Wee1) | WEE 1 homolog 1 (S. pombe) | 0.0029317 | 357.86 | 313.74 | 1.14 |
| 7 |  | [Cell Cycle: G2/M Checkpoint](http://cgap.nci.nih.gov/Pathways/BioCarta/m_g2Pathway) | [Cdc25c](http://www.ncbi.nlm.nih.gov/entrez/query.fcgi?cmd=search&db=gene&term=Cdc25c) | cell division cycle 25 homolog C (S. pombe) | 0.022479 | 49.55 | 58.92 | 0.84 |
| 8 |  | [Cell Cycle: G2/M Checkpoint](http://cgap.nci.nih.gov/Pathways/BioCarta/m_g2Pathway) | [Brca1](http://www.ncbi.nlm.nih.gov/entrez/query.fcgi?cmd=search&db=gene&term=Brca1) | breast cancer 1 | 0.0787369 | 12.37 | 14.04 | 0.88 |
| 9 |  | [Cell Cycle: G2/M Checkpoint](http://cgap.nci.nih.gov/Pathways/BioCarta/m_g2Pathway) | [Chek1](http://www.ncbi.nlm.nih.gov/entrez/query.fcgi?cmd=search&db=gene&term=Chek1) | checkpoint kinase 1 homolog (S. pombe) | 0.9139504 | 60.12 | 59.74 | 1.01 |
| 1 | m_dreampathway | [Repression of Pain Sensation by the Transcriptional Regulator DREAM](http://cgap.nci.nih.gov/Pathways/BioCarta/m_dreampathway) | [Fos](http://www.ncbi.nlm.nih.gov/entrez/query.fcgi?cmd=search&db=gene&term=Fos) | FBJ osteosarcoma oncogene | < 1e-07 | 1804.66 | 294.09 | 6.14 |
| 2 |  | [Repression of Pain Sensation by the Transcriptional Regulator DREAM](http://cgap.nci.nih.gov/Pathways/BioCarta/m_dreampathway) | [Jun](http://www.ncbi.nlm.nih.gov/entrez/query.fcgi?cmd=search&db=gene&term=Jun) | Jun oncogene | 0.0005914 | 270.82 | 205.78 | 1.32 |
| 3 |  | [Repression of Pain Sensation by the Transcriptional Regulator DREAM](http://cgap.nci.nih.gov/Pathways/BioCarta/m_dreampathway) | [Prkacb](http://www.ncbi.nlm.nih.gov/entrez/query.fcgi?cmd=search&db=gene&term=Prkacb) | protein kinase, cAMP dependent, catalytic, beta | 0.0006388 | 238.56 | 190.67 | 1.25 |
| 4 |  | [Repression of Pain Sensation by the Transcriptional Regulator DREAM](http://cgap.nci.nih.gov/Pathways/BioCarta/m_dreampathway) | [Crem](http://www.ncbi.nlm.nih.gov/entrez/query.fcgi?cmd=search&db=gene&term=Crem) | cAMP responsive element modulator | 0.3144703 | 38.14 | 35.59 | 1.07 |
| 5 |  | [Repression of Pain Sensation by the Transcriptional Regulator DREAM](http://cgap.nci.nih.gov/Pathways/BioCarta/m_dreampathway) | [Creb1](http://www.ncbi.nlm.nih.gov/entrez/query.fcgi?cmd=search&db=gene&term=Creb1) | cAMP responsive element binding protein 1 | 0.7553857 | 48.38 | 50.82 | 0.95 |
| 1 | m_bcrPathway | [BCR Signaling Pathway](http://cgap.nci.nih.gov/Pathways/BioCarta/m_bcrPathway) | [Fos](http://www.ncbi.nlm.nih.gov/entrez/query.fcgi?cmd=search&db=gene&term=Fos) | FBJ osteosarcoma oncogene | < 1e-07 | 1804.66 | 294.09 | 6.14 |
| 2 |  | [BCR Signaling Pathway](http://cgap.nci.nih.gov/Pathways/BioCarta/m_bcrPathway) | [Prkcb](http://www.ncbi.nlm.nih.gov/entrez/query.fcgi?cmd=search&db=gene&term=Prkcb) | protein kinase C, beta | 0.000107 | 173.52 | 135.08 | 1.28 |
| 3 |  | [BCR Signaling Pathway](http://cgap.nci.nih.gov/Pathways/BioCarta/m_bcrPathway) | [Jun](http://www.ncbi.nlm.nih.gov/entrez/query.fcgi?cmd=search&db=gene&term=Jun) | Jun oncogene | 0.0005914 | 270.82 | 205.78 | 1.32 |
| 4 |  | [BCR Signaling Pathway](http://cgap.nci.nih.gov/Pathways/BioCarta/m_bcrPathway) | [Vav1](http://www.ncbi.nlm.nih.gov/entrez/query.fcgi?cmd=search&db=gene&term=Vav1) | vav 1 oncogene | 0.0006129 | 23.74 | 19.16 | 1.24 |
| 5 |  | [BCR Signaling Pathway](http://cgap.nci.nih.gov/Pathways/BioCarta/m_bcrPathway) | [Sos1](http://www.ncbi.nlm.nih.gov/entrez/query.fcgi?cmd=search&db=gene&term=Sos1) | son of sevenless homolog 1 (Drosophila) | 0.0052437 | 62.98 | 76.11 | 0.83 |
| 6 |  | [BCR Signaling Pathway](http://cgap.nci.nih.gov/Pathways/BioCarta/m_bcrPathway) | [Mapk14](http://www.ncbi.nlm.nih.gov/entrez/query.fcgi?cmd=search&db=gene&term=Mapk14) | mitogen-activated protein kinase 14 | 0.0123923 | 55 | 71.64 | 0.77 |
| 7 |  | [BCR Signaling Pathway](http://cgap.nci.nih.gov/Pathways/BioCarta/m_bcrPathway) | [Mapk8](http://www.ncbi.nlm.nih.gov/entrez/query.fcgi?cmd=search&db=gene&term=Mapk8) | mitogen-activated protein kinase 8 | 0.0431864 | 31.06 | 25.43 | 1.22 |
| 8 |  | [BCR Signaling Pathway](http://cgap.nci.nih.gov/Pathways/BioCarta/m_bcrPathway) | [Nfatc2](http://www.ncbi.nlm.nih.gov/entrez/query.fcgi?cmd=search&db=gene&term=Nfatc2) | nuclear factor of activated T-cells, cytoplasmic, calcineurin-dependent 2 | 0.0683684 | 14.9 | 16.65 | 0.9 |
| 9 |  | [BCR Signaling Pathway](http://cgap.nci.nih.gov/Pathways/BioCarta/m_bcrPathway) | [Nfatc3](http://www.ncbi.nlm.nih.gov/entrez/query.fcgi?cmd=search&db=gene&term=Nfatc3) | nuclear factor of activated T-cells, cytoplasmic, calcineurin-dependent 3 | 0.1778903 | 211.8 | 241.23 | 0.88 |
| 10 |  | [BCR Signaling Pathway](http://cgap.nci.nih.gov/Pathways/BioCarta/m_bcrPathway) | [Btk](http://www.ncbi.nlm.nih.gov/entrez/query.fcgi?cmd=search&db=gene&term=Btk) | Bruton agammaglobulinemia tyrosine kinase | 0.8353834 | 57.93 | 57.15 | 1.01 |
| 1 | m_tcraPathway | [Lck and Fyn tyrosine kinases in initiation of TCR Activation](http://cgap.nci.nih.gov/Pathways/BioCarta/m_tcraPathway) | [Cd3d](http://www.ncbi.nlm.nih.gov/entrez/query.fcgi?cmd=search&db=gene&term=Cd3d) | CD3 antigen, delta polypeptide | 1.3e-05 | 154.41 | 106.54 | 1.45 |
| 2 |  | [Lck and Fyn tyrosine kinases in initiation of TCR Activation](http://cgap.nci.nih.gov/Pathways/BioCarta/m_tcraPathway) | [Cd3g](http://www.ncbi.nlm.nih.gov/entrez/query.fcgi?cmd=search&db=gene&term=Cd3g) | CD3 antigen, gamma polypeptide | 5.02e-05 | 250.04 | 181.14 | 1.38 |
| 3 |  | [Lck and Fyn tyrosine kinases in initiation of TCR Activation](http://cgap.nci.nih.gov/Pathways/BioCarta/m_tcraPathway) | [Ptprc](http://www.ncbi.nlm.nih.gov/entrez/query.fcgi?cmd=search&db=gene&term=Ptprc) | protein tyrosine phosphatase, receptor type, C | 0.0001698 | 219.5 | 166.65 | 1.32 |
| 4 |  | [Lck and Fyn tyrosine kinases in initiation of TCR Activation](http://cgap.nci.nih.gov/Pathways/BioCarta/m_tcraPathway) | [Tcra](http://www.ncbi.nlm.nih.gov/entrez/query.fcgi?cmd=search&db=gene&term=Tcra) | T-cell receptor alpha chain | 0.0007768 | 29.96 | 24.36 | 1.23 |
| 5 |  | [Lck and Fyn tyrosine kinases in initiation of TCR Activation](http://cgap.nci.nih.gov/Pathways/BioCarta/m_tcraPathway) | [Zap70](http://www.ncbi.nlm.nih.gov/entrez/query.fcgi?cmd=search&db=gene&term=Zap70) | zeta-chain (TCR) associated protein kinase | 0.001192 | 540.43 | 424.72 | 1.27 |
| 6 |  | [Lck and Fyn tyrosine kinases in initiation of TCR Activation](http://cgap.nci.nih.gov/Pathways/BioCarta/m_tcraPathway) | [Lck](http://www.ncbi.nlm.nih.gov/entrez/query.fcgi?cmd=search&db=gene&term=Lck) | lymphocyte protein tyrosine kinase | 0.0016033 | 90.08 | 69.9 | 1.29 |
| 7 |  | [Lck and Fyn tyrosine kinases in initiation of TCR Activation](http://cgap.nci.nih.gov/Pathways/BioCarta/m_tcraPathway) | [Fyn](http://www.ncbi.nlm.nih.gov/entrez/query.fcgi?cmd=search&db=gene&term=Fyn) | Fyn proto-oncogene | 0.0296932 | 108.73 | 88.59 | 1.23 |
| 8 |  | [Lck and Fyn tyrosine kinases in initiation of TCR Activation](http://cgap.nci.nih.gov/Pathways/BioCarta/m_tcraPathway) | [Cd247](http://www.ncbi.nlm.nih.gov/entrez/query.fcgi?cmd=search&db=gene&term=Cd247) | CD247 antigen | 0.50389 | 12.94 | 13.92 | 0.93 |
| 9 |  | [Lck and Fyn tyrosine kinases in initiation of TCR Activation](http://cgap.nci.nih.gov/Pathways/BioCarta/m_tcraPathway) | [Cd4](http://www.ncbi.nlm.nih.gov/entrez/query.fcgi?cmd=search&db=gene&term=Cd4) | CD4 antigen | 0.9710901 | 18.59 | 18.53 | 1 |
| 1 | m_ngfPathway | [Nerve growth factor pathway (NGF)](http://cgap.nci.nih.gov/Pathways/BioCarta/m_ngfPathway) | [Fos](http://www.ncbi.nlm.nih.gov/entrez/query.fcgi?cmd=search&db=gene&term=Fos) | FBJ osteosarcoma oncogene | < 1e-07 | 1804.66 | 294.09 | 6.14 |
| 2 |  | [Nerve growth factor pathway (NGF)](http://cgap.nci.nih.gov/Pathways/BioCarta/m_ngfPathway) | [Ngfr](http://www.ncbi.nlm.nih.gov/entrez/query.fcgi?cmd=search&db=gene&term=Ngfr) | nerve growth factor receptor (TNFR superfamily, member 16) | 0.000162 | 230.03 | 189.19 | 1.22 |
| 3 |  | [Nerve growth factor pathway (NGF)](http://cgap.nci.nih.gov/Pathways/BioCarta/m_ngfPathway) | [Ngf](http://www.ncbi.nlm.nih.gov/entrez/query.fcgi?cmd=search&db=gene&term=Ngf) | nerve growth factor | 0.0001997 | 56.89 | 72.78 | 0.78 |
| 4 |  | [Nerve growth factor pathway (NGF)](http://cgap.nci.nih.gov/Pathways/BioCarta/m_ngfPathway) | [Jun](http://www.ncbi.nlm.nih.gov/entrez/query.fcgi?cmd=search&db=gene&term=Jun) | Jun oncogene | 0.0005914 | 270.82 | 205.78 | 1.32 |
| 5 |  | [Nerve growth factor pathway (NGF)](http://cgap.nci.nih.gov/Pathways/BioCarta/m_ngfPathway) | [Sos1](http://www.ncbi.nlm.nih.gov/entrez/query.fcgi?cmd=search&db=gene&term=Sos1) | son of sevenless homolog 1 (Drosophila) | 0.0052437 | 62.98 | 76.11 | 0.83 |
| 6 |  | [Nerve growth factor pathway (NGF)](http://cgap.nci.nih.gov/Pathways/BioCarta/m_ngfPathway) | [Csnk2a2](http://www.ncbi.nlm.nih.gov/entrez/query.fcgi?cmd=search&db=gene&term=Csnk2a2) | casein kinase 2, alpha prime polypeptide | 0.0102407 | 1559.16 | 1719.9 | 0.91 |
| 7 |  | [Nerve growth factor pathway (NGF)](http://cgap.nci.nih.gov/Pathways/BioCarta/m_ngfPathway) | [Mapk8](http://www.ncbi.nlm.nih.gov/entrez/query.fcgi?cmd=search&db=gene&term=Mapk8) | mitogen-activated protein kinase 8 | 0.0431864 | 31.06 | 25.43 | 1.22 |
| 8 |  | [Nerve growth factor pathway (NGF)](http://cgap.nci.nih.gov/Pathways/BioCarta/m_ngfPathway) | [Pik3cg](http://www.ncbi.nlm.nih.gov/entrez/query.fcgi?cmd=search&db=gene&term=Pik3cg) | phosphoinositide-3-kinase, catalytic, gamma polypeptide | 0.3272953 | 16.25 | 14.76 | 1.1 |
| 9 |  | [Nerve growth factor pathway (NGF)](http://cgap.nci.nih.gov/Pathways/BioCarta/m_ngfPathway) | [Csnk2a1](http://www.ncbi.nlm.nih.gov/entrez/query.fcgi?cmd=search&db=gene&term=Csnk2a1) | casein kinase 2, alpha 1 polypeptide | 0.5759663 | 255.89 | 272.9 | 0.94 |
| 10 |  | [Nerve growth factor pathway (NGF)](http://cgap.nci.nih.gov/Pathways/BioCarta/m_ngfPathway) | [Pik3r1](http://www.ncbi.nlm.nih.gov/entrez/query.fcgi?cmd=search&db=gene&term=Pik3r1) | phosphatidylinositol 3-kinase, regulatory subunit, polypeptide 1 (p85 alpha) | 0.6503694 | 113.16 | 110.05 | 1.03 |
| 1 | m_epoPathway | [EPO Signaling Pathway](http://cgap.nci.nih.gov/Pathways/BioCarta/m_epoPathway) | [Fos](http://www.ncbi.nlm.nih.gov/entrez/query.fcgi?cmd=search&db=gene&term=Fos) | FBJ osteosarcoma oncogene | < 1e-07 | 1804.66 | 294.09 | 6.14 |
| 2 |  | [EPO Signaling Pathway](http://cgap.nci.nih.gov/Pathways/BioCarta/m_epoPathway) | [Jun](http://www.ncbi.nlm.nih.gov/entrez/query.fcgi?cmd=search&db=gene&term=Jun) | Jun oncogene | 0.0005914 | 270.82 | 205.78 | 1.32 |
| 3 |  | [EPO Signaling Pathway](http://cgap.nci.nih.gov/Pathways/BioCarta/m_epoPathway) | [Sos1](http://www.ncbi.nlm.nih.gov/entrez/query.fcgi?cmd=search&db=gene&term=Sos1) | son of sevenless homolog 1 (Drosophila) | 0.0052437 | 62.98 | 76.11 | 0.83 |
| 4 |  | [EPO Signaling Pathway](http://cgap.nci.nih.gov/Pathways/BioCarta/m_epoPathway) | [Csnk2a2](http://www.ncbi.nlm.nih.gov/entrez/query.fcgi?cmd=search&db=gene&term=Csnk2a2) | casein kinase 2, alpha prime polypeptide | 0.0102407 | 1559.16 | 1719.9 | 0.91 |
| 5 |  | [EPO Signaling Pathway](http://cgap.nci.nih.gov/Pathways/BioCarta/m_epoPathway) | [Stat5a](http://www.ncbi.nlm.nih.gov/entrez/query.fcgi?cmd=search&db=gene&term=Stat5a) | signal transducer and activator of transcription 5A | 0.0353627 | 63.97 | 73.88 | 0.87 |
| 6 |  | [EPO Signaling Pathway](http://cgap.nci.nih.gov/Pathways/BioCarta/m_epoPathway) | [Mapk8](http://www.ncbi.nlm.nih.gov/entrez/query.fcgi?cmd=search&db=gene&term=Mapk8) | mitogen-activated protein kinase 8 | 0.0431864 | 31.06 | 25.43 | 1.22 |
| 7 |  | [EPO Signaling Pathway](http://cgap.nci.nih.gov/Pathways/BioCarta/m_epoPathway) | [Csnk2a1](http://www.ncbi.nlm.nih.gov/entrez/query.fcgi?cmd=search&db=gene&term=Csnk2a1) | casein kinase 2, alpha 1 polypeptide | 0.5759663 | 255.89 | 272.9 | 0.94 |
| 1 | m_pcafpathway | [The information-processing pathway at the IFN-beta enhancer](http://cgap.nci.nih.gov/Pathways/BioCarta/m_pcafpathway) | [Irf1](http://www.ncbi.nlm.nih.gov/entrez/query.fcgi?cmd=search&db=gene&term=Irf1) | interferon regulatory factor 1 | 3.64e-05 | 302.47 | 221.93 | 1.36 |
| 2 |  | [The information-processing pathway at the IFN-beta enhancer](http://cgap.nci.nih.gov/Pathways/BioCarta/m_pcafpathway) | [Nfkb1](http://www.ncbi.nlm.nih.gov/entrez/query.fcgi?cmd=search&db=gene&term=Nfkb1) | nuclear factor of kappa light polypeptide gene enhancer in B-cells 1, p105 | 0.0002841 | 636.36 | 804.29 | 0.79 |
| 3 |  | [The information-processing pathway at the IFN-beta enhancer](http://cgap.nci.nih.gov/Pathways/BioCarta/m_pcafpathway) | [Jun](http://www.ncbi.nlm.nih.gov/entrez/query.fcgi?cmd=search&db=gene&term=Jun) | Jun oncogene | 0.0005914 | 270.82 | 205.78 | 1.32 |
| 4 |  | [The information-processing pathway at the IFN-beta enhancer](http://cgap.nci.nih.gov/Pathways/BioCarta/m_pcafpathway) | [Irf3](http://www.ncbi.nlm.nih.gov/entrez/query.fcgi?cmd=search&db=gene&term=Irf3) | interferon regulatory factor 3 | 0.0042774 | 64.27 | 80.65 | 0.8 |
| 5 |  | [The information-processing pathway at the IFN-beta enhancer](http://cgap.nci.nih.gov/Pathways/BioCarta/m_pcafpathway) | [Kat2b](http://www.ncbi.nlm.nih.gov/entrez/query.fcgi?cmd=search&db=gene&term=Kat2b) | K(lysine) acetyltransferase 2B | 0.4449167 | 32.44 | 34.15 | 0.95 |
| 1 | m_vipPathway | [Neuropeptides VIP and PACAP inhibit the apoptosis of activated T cells](http://cgap.nci.nih.gov/Pathways/BioCarta/m_vipPathway) | [Nfkbia](http://www.ncbi.nlm.nih.gov/entrez/query.fcgi?cmd=search&db=gene&term=Nfkbia) | nuclear factor of kappa light polypeptide gene enhancer in B-cells inhibitor, alpha | 0.0002631 | 1140.45 | 852.99 | 1.34 |
| 2 |  | [Neuropeptides VIP and PACAP inhibit the apoptosis of activated T cells](http://cgap.nci.nih.gov/Pathways/BioCarta/m_vipPathway) | [Nfkb1](http://www.ncbi.nlm.nih.gov/entrez/query.fcgi?cmd=search&db=gene&term=Nfkb1) | nuclear factor of kappa light polypeptide gene enhancer in B-cells 1, p105 | 0.0002841 | 636.36 | 804.29 | 0.79 |
| 3 |  | [Neuropeptides VIP and PACAP inhibit the apoptosis of activated T cells](http://cgap.nci.nih.gov/Pathways/BioCarta/m_vipPathway) | [Prkacb](http://www.ncbi.nlm.nih.gov/entrez/query.fcgi?cmd=search&db=gene&term=Prkacb) | protein kinase, cAMP dependent, catalytic, beta | 0.0006388 | 238.56 | 190.67 | 1.25 |
| 4 |  | [Neuropeptides VIP and PACAP inhibit the apoptosis of activated T cells](http://cgap.nci.nih.gov/Pathways/BioCarta/m_vipPathway) | [Egr2](http://www.ncbi.nlm.nih.gov/entrez/query.fcgi?cmd=search&db=gene&term=Egr2) | early growth response 2 | 0.0185323 | 620.73 | 683.03 | 0.91 |
| 5 |  | [Neuropeptides VIP and PACAP inhibit the apoptosis of activated T cells](http://cgap.nci.nih.gov/Pathways/BioCarta/m_vipPathway) | [Nfatc2](http://www.ncbi.nlm.nih.gov/entrez/query.fcgi?cmd=search&db=gene&term=Nfatc2) | nuclear factor of activated T-cells, cytoplasmic, calcineurin-dependent 2 | 0.0683684 | 14.9 | 16.65 | 0.9 |
| 1 | m_biopeptidesPathway | [Bioactive Peptide Induced Signaling Pathway](http://cgap.nci.nih.gov/Pathways/BioCarta/m_biopeptidesPathway) | [Stat1](http://www.ncbi.nlm.nih.gov/entrez/query.fcgi?cmd=search&db=gene&term=Stat1) | signal transducer and activator of transcription 1 | < 1e-07 | 804.27 | 394.42 | 2.04 |
| 2 |  | [Bioactive Peptide Induced Signaling Pathway](http://cgap.nci.nih.gov/Pathways/BioCarta/m_biopeptidesPathway) | [Prkcb](http://www.ncbi.nlm.nih.gov/entrez/query.fcgi?cmd=search&db=gene&term=Prkcb) | protein kinase C, beta | 0.000107 | 173.52 | 135.08 | 1.28 |
| 3 |  | [Bioactive Peptide Induced Signaling Pathway](http://cgap.nci.nih.gov/Pathways/BioCarta/m_biopeptidesPathway) | [Sos1](http://www.ncbi.nlm.nih.gov/entrez/query.fcgi?cmd=search&db=gene&term=Sos1) | son of sevenless homolog 1 (Drosophila) | 0.0052437 | 62.98 | 76.11 | 0.83 |
| 4 |  | [Bioactive Peptide Induced Signaling Pathway](http://cgap.nci.nih.gov/Pathways/BioCarta/m_biopeptidesPathway) | [Mapk14](http://www.ncbi.nlm.nih.gov/entrez/query.fcgi?cmd=search&db=gene&term=Mapk14) | mitogen-activated protein kinase 14 | 0.0123923 | 55 | 71.64 | 0.77 |
| 5 |  | [Bioactive Peptide Induced Signaling Pathway](http://cgap.nci.nih.gov/Pathways/BioCarta/m_biopeptidesPathway) | [Fyn](http://www.ncbi.nlm.nih.gov/entrez/query.fcgi?cmd=search&db=gene&term=Fyn) | Fyn proto-oncogene | 0.0296932 | 108.73 | 88.59 | 1.23 |
| 6 |  | [Bioactive Peptide Induced Signaling Pathway](http://cgap.nci.nih.gov/Pathways/BioCarta/m_biopeptidesPathway) | [Stat5a](http://www.ncbi.nlm.nih.gov/entrez/query.fcgi?cmd=search&db=gene&term=Stat5a) | signal transducer and activator of transcription 5A | 0.0353627 | 63.97 | 73.88 | 0.87 |
| 7 |  | [Bioactive Peptide Induced Signaling Pathway](http://cgap.nci.nih.gov/Pathways/BioCarta/m_biopeptidesPathway) | [Mapk8](http://www.ncbi.nlm.nih.gov/entrez/query.fcgi?cmd=search&db=gene&term=Mapk8) | mitogen-activated protein kinase 8 | 0.0431864 | 31.06 | 25.43 | 1.22 |
| 8 |  | [Bioactive Peptide Induced Signaling Pathway](http://cgap.nci.nih.gov/Pathways/BioCarta/m_biopeptidesPathway) | [Mapk1](http://www.ncbi.nlm.nih.gov/entrez/query.fcgi?cmd=search&db=gene&term=Mapk1) | mitogen-activated protein kinase 1 | 0.9818276 | 246.1 | 247.01 | 1 |
| 1 | m_il2Pathway | [IL 2 signaling pathway](http://cgap.nci.nih.gov/Pathways/BioCarta/m_il2Pathway) | [Fos](http://www.ncbi.nlm.nih.gov/entrez/query.fcgi?cmd=search&db=gene&term=Fos) | FBJ osteosarcoma oncogene | < 1e-07 | 1804.66 | 294.09 | 6.14 |
| 2 |  | [IL 2 signaling pathway](http://cgap.nci.nih.gov/Pathways/BioCarta/m_il2Pathway) | [Jun](http://www.ncbi.nlm.nih.gov/entrez/query.fcgi?cmd=search&db=gene&term=Jun) | Jun oncogene | 0.0005914 | 270.82 | 205.78 | 1.32 |
| 3 |  | [IL 2 signaling pathway](http://cgap.nci.nih.gov/Pathways/BioCarta/m_il2Pathway) | [Lck](http://www.ncbi.nlm.nih.gov/entrez/query.fcgi?cmd=search&db=gene&term=Lck) | lymphocyte protein tyrosine kinase | 0.0016033 | 90.08 | 69.9 | 1.29 |
| 4 |  | [IL 2 signaling pathway](http://cgap.nci.nih.gov/Pathways/BioCarta/m_il2Pathway) | [Il2rg](http://www.ncbi.nlm.nih.gov/entrez/query.fcgi?cmd=search&db=gene&term=Il2rg) | interleukin 2 receptor, gamma chain | 0.0034212 | 219.49 | 271.54 | 0.81 |
| 5 |  | [IL 2 signaling pathway](http://cgap.nci.nih.gov/Pathways/BioCarta/m_il2Pathway) | [Il2rb](http://www.ncbi.nlm.nih.gov/entrez/query.fcgi?cmd=search&db=gene&term=Il2rb) | interleukin 2 receptor, beta chain | 0.0038096 | 78.49 | 63.56 | 1.23 |
| 6 |  | [IL 2 signaling pathway](http://cgap.nci.nih.gov/Pathways/BioCarta/m_il2Pathway) | [Sos1](http://www.ncbi.nlm.nih.gov/entrez/query.fcgi?cmd=search&db=gene&term=Sos1) | son of sevenless homolog 1 (Drosophila) | 0.0052437 | 62.98 | 76.11 | 0.83 |
| 7 |  | [IL 2 signaling pathway](http://cgap.nci.nih.gov/Pathways/BioCarta/m_il2Pathway) | [Csnk2a2](http://www.ncbi.nlm.nih.gov/entrez/query.fcgi?cmd=search&db=gene&term=Csnk2a2) | casein kinase 2, alpha prime polypeptide | 0.0102407 | 1559.16 | 1719.9 | 0.91 |
| 8 |  | [IL 2 signaling pathway](http://cgap.nci.nih.gov/Pathways/BioCarta/m_il2Pathway) | [Stat5a](http://www.ncbi.nlm.nih.gov/entrez/query.fcgi?cmd=search&db=gene&term=Stat5a) | signal transducer and activator of transcription 5A | 0.0353627 | 63.97 | 73.88 | 0.87 |
| 9 |  | [IL 2 signaling pathway](http://cgap.nci.nih.gov/Pathways/BioCarta/m_il2Pathway) | [Mapk8](http://www.ncbi.nlm.nih.gov/entrez/query.fcgi?cmd=search&db=gene&term=Mapk8) | mitogen-activated protein kinase 8 | 0.0431864 | 31.06 | 25.43 | 1.22 |
| 10 |  | [IL 2 signaling pathway](http://cgap.nci.nih.gov/Pathways/BioCarta/m_il2Pathway) | [Jak1](http://www.ncbi.nlm.nih.gov/entrez/query.fcgi?cmd=search&db=gene&term=Jak1) | Janus kinase 1 | 0.1036342 | 128.89 | 156.56 | 0.82 |
| 11 |  | [IL 2 signaling pathway](http://cgap.nci.nih.gov/Pathways/BioCarta/m_il2Pathway) | [Csnk2a1](http://www.ncbi.nlm.nih.gov/entrez/query.fcgi?cmd=search&db=gene&term=Csnk2a1) | casein kinase 2, alpha 1 polypeptide | 0.5759663 | 255.89 | 272.9 | 0.94 |
| 12 |  | [IL 2 signaling pathway](http://cgap.nci.nih.gov/Pathways/BioCarta/m_il2Pathway) | [Jak3](http://www.ncbi.nlm.nih.gov/entrez/query.fcgi?cmd=search&db=gene&term=Jak3) | Janus kinase 3 | 0.5989007 | 10.8 | 10.31 | 1.05 |
| 1 | m_rnaPathway | [Double Stranded RNA Induced Gene Expression](http://cgap.nci.nih.gov/Pathways/BioCarta/m_rnaPathway) | [Nfkbia](http://www.ncbi.nlm.nih.gov/entrez/query.fcgi?cmd=search&db=gene&term=Nfkbia) | nuclear factor of kappa light polypeptide gene enhancer in B-cells inhibitor, alpha | 0.0002631 | 1140.45 | 852.99 | 1.34 |
| 2 |  | [Double Stranded RNA Induced Gene Expression](http://cgap.nci.nih.gov/Pathways/BioCarta/m_rnaPathway) | [Nfkb1](http://www.ncbi.nlm.nih.gov/entrez/query.fcgi?cmd=search&db=gene&term=Nfkb1) | nuclear factor of kappa light polypeptide gene enhancer in B-cells 1, p105 | 0.0002841 | 636.36 | 804.29 | 0.79 |
| 3 |  | [Double Stranded RNA Induced Gene Expression](http://cgap.nci.nih.gov/Pathways/BioCarta/m_rnaPathway) | [Trp53](http://www.ncbi.nlm.nih.gov/entrez/query.fcgi?cmd=search&db=gene&term=Trp53) | transformation related protein 53 | 0.0027856 | 11.53 | 14.37 | 0.8 |
| 4 |  | [Double Stranded RNA Induced Gene Expression](http://cgap.nci.nih.gov/Pathways/BioCarta/m_rnaPathway) | [Eif2s2](http://www.ncbi.nlm.nih.gov/entrez/query.fcgi?cmd=search&db=gene&term=Eif2s2) | eukaryotic translation initiation factor 2, subunit 2 (beta) | 0.0535128 | 65.33 | 75.67 | 0.86 |
| 5 |  | [Double Stranded RNA Induced Gene Expression](http://cgap.nci.nih.gov/Pathways/BioCarta/m_rnaPathway) | [Eif2s1](http://www.ncbi.nlm.nih.gov/entrez/query.fcgi?cmd=search&db=gene&term=Eif2s1) | eukaryotic translation initiation factor 2, subunit 1 alpha | 0.0653904 | 20.59 | 17.93 | 1.15 |
| 1 | m_atmPathway | [ATM Signaling Pathway](http://cgap.nci.nih.gov/Pathways/BioCarta/m_atmPathway) | [Nfkbia](http://www.ncbi.nlm.nih.gov/entrez/query.fcgi?cmd=search&db=gene&term=Nfkbia) | nuclear factor of kappa light polypeptide gene enhancer in B-cells inhibitor, alpha | 0.0002631 | 1140.45 | 852.99 | 1.34 |
| 2 |  | [ATM Signaling Pathway](http://cgap.nci.nih.gov/Pathways/BioCarta/m_atmPathway) | [Nfkb1](http://www.ncbi.nlm.nih.gov/entrez/query.fcgi?cmd=search&db=gene&term=Nfkb1) | nuclear factor of kappa light polypeptide gene enhancer in B-cells 1, p105 | 0.0002841 | 636.36 | 804.29 | 0.79 |
| 3 |  | [ATM Signaling Pathway](http://cgap.nci.nih.gov/Pathways/BioCarta/m_atmPathway) | [Jun](http://www.ncbi.nlm.nih.gov/entrez/query.fcgi?cmd=search&db=gene&term=Jun) | Jun oncogene | 0.0005914 | 270.82 | 205.78 | 1.32 |
| 4 |  | [ATM Signaling Pathway](http://cgap.nci.nih.gov/Pathways/BioCarta/m_atmPathway) | [Gadd45a](http://www.ncbi.nlm.nih.gov/entrez/query.fcgi?cmd=search&db=gene&term=Gadd45a) | growth arrest and DNA-damage-inducible 45 alpha | 0.001651 | 66.3 | 85.78 | 0.77 |
| 5 |  | [ATM Signaling Pathway](http://cgap.nci.nih.gov/Pathways/BioCarta/m_atmPathway) | [Trp53](http://www.ncbi.nlm.nih.gov/entrez/query.fcgi?cmd=search&db=gene&term=Trp53) | transformation related protein 53 | 0.0027856 | 11.53 | 14.37 | 0.8 |
| 6 |  | [ATM Signaling Pathway](http://cgap.nci.nih.gov/Pathways/BioCarta/m_atmPathway) | [Rad51](http://www.ncbi.nlm.nih.gov/entrez/query.fcgi?cmd=search&db=gene&term=Rad51) | RAD51 homolog (S. cerevisiae) | 0.0029127 | 101.8 | 128.53 | 0.79 |
| 7 |  | [ATM Signaling Pathway](http://cgap.nci.nih.gov/Pathways/BioCarta/m_atmPathway) | [Mapk8](http://www.ncbi.nlm.nih.gov/entrez/query.fcgi?cmd=search&db=gene&term=Mapk8) | mitogen-activated protein kinase 8 | 0.0431864 | 31.06 | 25.43 | 1.22 |
| 8 |  | [ATM Signaling Pathway](http://cgap.nci.nih.gov/Pathways/BioCarta/m_atmPathway) | [Mre11a](http://www.ncbi.nlm.nih.gov/entrez/query.fcgi?cmd=search&db=gene&term=Mre11a) | meiotic recombination 11 homolog A (S. cerevisiae) | 0.0523881 | 132.58 | 118.41 | 1.12 |
| 9 |  | [ATM Signaling Pathway](http://cgap.nci.nih.gov/Pathways/BioCarta/m_atmPathway) | [Brca1](http://www.ncbi.nlm.nih.gov/entrez/query.fcgi?cmd=search&db=gene&term=Brca1) | breast cancer 1 | 0.0787369 | 12.37 | 14.04 | 0.88 |
| 10 |  | [ATM Signaling Pathway](http://cgap.nci.nih.gov/Pathways/BioCarta/m_atmPathway) | [Chek1](http://www.ncbi.nlm.nih.gov/entrez/query.fcgi?cmd=search&db=gene&term=Chek1) | checkpoint kinase 1 homolog (S. pombe) | 0.9139504 | 60.12 | 59.74 | 1.01 |
| 1 | m_DNAfragmentPathway | [Apoptotic DNA fragmentation and tissue homeostasis](http://cgap.nci.nih.gov/Pathways/BioCarta/m_DNAfragmentPathway) | [Top2a](http://www.ncbi.nlm.nih.gov/entrez/query.fcgi?cmd=search&db=gene&term=Top2a) | topoisomerase (DNA) II alpha | 2.22e-05 | 399.02 | 549.67 | 0.73 |
| 2 |  | [Apoptotic DNA fragmentation and tissue homeostasis](http://cgap.nci.nih.gov/Pathways/BioCarta/m_DNAfragmentPathway) | [Hmgb2](http://www.ncbi.nlm.nih.gov/entrez/query.fcgi?cmd=search&db=gene&term=Hmgb2) | high mobility group box 2 | 0.0003332 | 511.67 | 624.45 | 0.82 |
| 3 |  | [Apoptotic DNA fragmentation and tissue homeostasis](http://cgap.nci.nih.gov/Pathways/BioCarta/m_DNAfragmentPathway) | [Gzmb](http://www.ncbi.nlm.nih.gov/entrez/query.fcgi?cmd=search&db=gene&term=Gzmb) | granzyme B | 0.0063458 | 29.91 | 22.72 | 1.32 |
| 4 |  | [Apoptotic DNA fragmentation and tissue homeostasis](http://cgap.nci.nih.gov/Pathways/BioCarta/m_DNAfragmentPathway) | [Top2b](http://www.ncbi.nlm.nih.gov/entrez/query.fcgi?cmd=search&db=gene&term=Top2b) | topoisomerase (DNA) II beta | 0.0350419 | 199.33 | 224.8 | 0.89 |
| 5 |  | [Apoptotic DNA fragmentation and tissue homeostasis](http://cgap.nci.nih.gov/Pathways/BioCarta/m_DNAfragmentPathway) | [Dffa](http://www.ncbi.nlm.nih.gov/entrez/query.fcgi?cmd=search&db=gene&term=Dffa) | DNA fragmentation factor, alpha subunit | 0.9246302 | 901.2 | 904.58 | 1 |
| 1 | m_etsPathway | [METS affect on Macrophage Differentiation](http://cgap.nci.nih.gov/Pathways/BioCarta/m_etsPathway) | [Fos](http://www.ncbi.nlm.nih.gov/entrez/query.fcgi?cmd=search&db=gene&term=Fos) | FBJ osteosarcoma oncogene | < 1e-07 | 1804.66 | 294.09 | 6.14 |
| 2 |  | [METS affect on Macrophage Differentiation](http://cgap.nci.nih.gov/Pathways/BioCarta/m_etsPathway) | [Jun](http://www.ncbi.nlm.nih.gov/entrez/query.fcgi?cmd=search&db=gene&term=Jun) | Jun oncogene | 0.0005914 | 270.82 | 205.78 | 1.32 |
| 3 |  | [METS affect on Macrophage Differentiation](http://cgap.nci.nih.gov/Pathways/BioCarta/m_etsPathway) | [Csf1r](http://www.ncbi.nlm.nih.gov/entrez/query.fcgi?cmd=search&db=gene&term=Csf1r) | colony stimulating factor 1 receptor | 0.0006572 | 988.19 | 829.17 | 1.19 |
| 4 |  | [METS affect on Macrophage Differentiation](http://cgap.nci.nih.gov/Pathways/BioCarta/m_etsPathway) | [Sin3b](http://www.ncbi.nlm.nih.gov/entrez/query.fcgi?cmd=search&db=gene&term=Sin3b) | transcriptional regulator, SIN3B (yeast) | 0.01136 | 36.89 | 32.02 | 1.15 |
| 5 |  | [METS affect on Macrophage Differentiation](http://cgap.nci.nih.gov/Pathways/BioCarta/m_etsPathway) | [Ets1](http://www.ncbi.nlm.nih.gov/entrez/query.fcgi?cmd=search&db=gene&term=Ets1) | E26 avian leukemia oncogene 1, 5' domain | 0.0272048 | 115.24 | 130.49 | 0.88 |
| 6 |  | [METS affect on Macrophage Differentiation](http://cgap.nci.nih.gov/Pathways/BioCarta/m_etsPathway) | [Etv3](http://www.ncbi.nlm.nih.gov/entrez/query.fcgi?cmd=search&db=gene&term=Etv3) | ets variant gene 3 | 0.0386373 | 521.32 | 581.33 | 0.9 |
| 7 |  | [METS affect on Macrophage Differentiation](http://cgap.nci.nih.gov/Pathways/BioCarta/m_etsPathway) | [Hdac5](http://www.ncbi.nlm.nih.gov/entrez/query.fcgi?cmd=search&db=gene&term=Hdac5) | histone deacetylase 5 | 0.1124833 | 576.79 | 547.32 | 1.05 |
| 8 |  | [METS affect on Macrophage Differentiation](http://cgap.nci.nih.gov/Pathways/BioCarta/m_etsPathway) | [Csf1](http://www.ncbi.nlm.nih.gov/entrez/query.fcgi?cmd=search&db=gene&term=Csf1) | colony stimulating factor 1 (macrophage) | 0.2870445 | 27.07 | 29.17 | 0.93 |
| 9 |  | [METS affect on Macrophage Differentiation](http://cgap.nci.nih.gov/Pathways/BioCarta/m_etsPathway) | [Rbl1](http://www.ncbi.nlm.nih.gov/entrez/query.fcgi?cmd=search&db=gene&term=Rbl1) | retinoblastoma-like 1 (p107) | 0.416018 | 20.93 | 22.45 | 0.93 |
| 10 |  | [METS affect on Macrophage Differentiation](http://cgap.nci.nih.gov/Pathways/BioCarta/m_etsPathway) | [Sin3a](http://www.ncbi.nlm.nih.gov/entrez/query.fcgi?cmd=search&db=gene&term=Sin3a) | transcriptional regulator, SIN3A (yeast) | 0.8778968 | 147.16 | 148.64 | 0.99 |
| 1 | m_igf1Pathway | [IGF-1 Signaling Pathway](http://cgap.nci.nih.gov/Pathways/BioCarta/m_igf1Pathway) | [Fos](http://www.ncbi.nlm.nih.gov/entrez/query.fcgi?cmd=search&db=gene&term=Fos) | FBJ osteosarcoma oncogene | < 1e-07 | 1804.66 | 294.09 | 6.14 |
| 2 |  | [IGF-1 Signaling Pathway](http://cgap.nci.nih.gov/Pathways/BioCarta/m_igf1Pathway) | [Jun](http://www.ncbi.nlm.nih.gov/entrez/query.fcgi?cmd=search&db=gene&term=Jun) | Jun oncogene | 0.0005914 | 270.82 | 205.78 | 1.32 |
| 3 |  | [IGF-1 Signaling Pathway](http://cgap.nci.nih.gov/Pathways/BioCarta/m_igf1Pathway) | [Irs1](http://www.ncbi.nlm.nih.gov/entrez/query.fcgi?cmd=search&db=gene&term=Irs1) | insulin receptor substrate 1 | 0.0009505 | 495.34 | 397.14 | 1.25 |
| 4 |  | [IGF-1 Signaling Pathway](http://cgap.nci.nih.gov/Pathways/BioCarta/m_igf1Pathway) | [Sos1](http://www.ncbi.nlm.nih.gov/entrez/query.fcgi?cmd=search&db=gene&term=Sos1) | son of sevenless homolog 1 (Drosophila) | 0.0052437 | 62.98 | 76.11 | 0.83 |
| 5 |  | [IGF-1 Signaling Pathway](http://cgap.nci.nih.gov/Pathways/BioCarta/m_igf1Pathway) | [Csnk2a2](http://www.ncbi.nlm.nih.gov/entrez/query.fcgi?cmd=search&db=gene&term=Csnk2a2) | casein kinase 2, alpha prime polypeptide | 0.0102407 | 1559.16 | 1719.9 | 0.91 |
| 6 |  | [IGF-1 Signaling Pathway](http://cgap.nci.nih.gov/Pathways/BioCarta/m_igf1Pathway) | [Mapk8](http://www.ncbi.nlm.nih.gov/entrez/query.fcgi?cmd=search&db=gene&term=Mapk8) | mitogen-activated protein kinase 8 | 0.0431864 | 31.06 | 25.43 | 1.22 |
| 7 |  | [IGF-1 Signaling Pathway](http://cgap.nci.nih.gov/Pathways/BioCarta/m_igf1Pathway) | [Igf1r](http://www.ncbi.nlm.nih.gov/entrez/query.fcgi?cmd=search&db=gene&term=Igf1r) | insulin-like growth factor I receptor | 0.0742816 | 28.83 | 31.96 | 0.9 |
| 8 |  | [IGF-1 Signaling Pathway](http://cgap.nci.nih.gov/Pathways/BioCarta/m_igf1Pathway) | [Pik3cg](http://www.ncbi.nlm.nih.gov/entrez/query.fcgi?cmd=search&db=gene&term=Pik3cg) | phosphoinositide-3-kinase, catalytic, gamma polypeptide | 0.3272953 | 16.25 | 14.76 | 1.1 |
| 9 |  | [IGF-1 Signaling Pathway](http://cgap.nci.nih.gov/Pathways/BioCarta/m_igf1Pathway) | [Csnk2a1](http://www.ncbi.nlm.nih.gov/entrez/query.fcgi?cmd=search&db=gene&term=Csnk2a1) | casein kinase 2, alpha 1 polypeptide | 0.5759663 | 255.89 | 272.9 | 0.94 |
| 10 |  | [IGF-1 Signaling Pathway](http://cgap.nci.nih.gov/Pathways/BioCarta/m_igf1Pathway) | [Pik3r1](http://www.ncbi.nlm.nih.gov/entrez/query.fcgi?cmd=search&db=gene&term=Pik3r1) | phosphatidylinositol 3-kinase, regulatory subunit, polypeptide 1 (p85 alpha) | 0.6503694 | 113.16 | 110.05 | 1.03 |
| 11 |  | [IGF-1 Signaling Pathway](http://cgap.nci.nih.gov/Pathways/BioCarta/m_igf1Pathway) | [Ptpn11](http://www.ncbi.nlm.nih.gov/entrez/query.fcgi?cmd=search&db=gene&term=Ptpn11) | protein tyrosine phosphatase, non-receptor type 11 | 0.6617678 | 64.25 | 67.58 | 0.95 |
| 1 | m_fasPathway | [FAS signaling pathway ( CD95 )](http://cgap.nci.nih.gov/Pathways/BioCarta/m_fasPathway) | [Lmna](http://www.ncbi.nlm.nih.gov/entrez/query.fcgi?cmd=search&db=gene&term=Lmna) | lamin A | 3.51e-05 | 3448.81 | 4356.2 | 0.79 |
| 2 |  | [FAS signaling pathway ( CD95 )](http://cgap.nci.nih.gov/Pathways/BioCarta/m_fasPathway) | [Map2k4](http://www.ncbi.nlm.nih.gov/entrez/query.fcgi?cmd=search&db=gene&term=Map2k4) | mitogen-activated protein kinase kinase 4 | 9.41e-05 | 341.42 | 495.55 | 0.69 |
| 3 |  | [FAS signaling pathway ( CD95 )](http://cgap.nci.nih.gov/Pathways/BioCarta/m_fasPathway) | [Jun](http://www.ncbi.nlm.nih.gov/entrez/query.fcgi?cmd=search&db=gene&term=Jun) | Jun oncogene | 0.0005914 | 270.82 | 205.78 | 1.32 |
| 4 |  | [FAS signaling pathway ( CD95 )](http://cgap.nci.nih.gov/Pathways/BioCarta/m_fasPathway) | [Lmnb1](http://www.ncbi.nlm.nih.gov/entrez/query.fcgi?cmd=search&db=gene&term=Lmnb1) | lamin B1 | 0.0347832 | 36.29 | 42.79 | 0.85 |
| 5 |  | [FAS signaling pathway ( CD95 )](http://cgap.nci.nih.gov/Pathways/BioCarta/m_fasPathway) | [Mapk8](http://www.ncbi.nlm.nih.gov/entrez/query.fcgi?cmd=search&db=gene&term=Mapk8) | mitogen-activated protein kinase 8 | 0.0431864 | 31.06 | 25.43 | 1.22 |
| 6 |  | [FAS signaling pathway ( CD95 )](http://cgap.nci.nih.gov/Pathways/BioCarta/m_fasPathway) | [Ptpn13](http://www.ncbi.nlm.nih.gov/entrez/query.fcgi?cmd=search&db=gene&term=Ptpn13) | protein tyrosine phosphatase, non-receptor type 13 | 0.0827796 | 74.72 | 87.51 | 0.85 |
| 7 |  | [FAS signaling pathway ( CD95 )](http://cgap.nci.nih.gov/Pathways/BioCarta/m_fasPathway) | [Pak2](http://www.ncbi.nlm.nih.gov/entrez/query.fcgi?cmd=search&db=gene&term=Pak2) | p21 protein (Cdc42/Rac)-activated kinase 2 | 0.2543885 | 52.58 | 58.87 | 0.89 |
| 8 |  | [FAS signaling pathway ( CD95 )](http://cgap.nci.nih.gov/Pathways/BioCarta/m_fasPathway) | [Dffa](http://www.ncbi.nlm.nih.gov/entrez/query.fcgi?cmd=search&db=gene&term=Dffa) | DNA fragmentation factor, alpha subunit | 0.9246302 | 901.2 | 904.58 | 1 |
| 1 | m_tgfbPathway | [TGF beta signaling pathway](http://cgap.nci.nih.gov/Pathways/BioCarta/m_tgfbPathway) | [Tgfb3](http://www.ncbi.nlm.nih.gov/entrez/query.fcgi?cmd=search&db=gene&term=Tgfb3) | transforming growth factor, beta 3 | 0.0015677 | 43.25 | 35.31 | 1.22 |
| 2 |  | [TGF beta signaling pathway](http://cgap.nci.nih.gov/Pathways/BioCarta/m_tgfbPathway) | [Tgfb2](http://www.ncbi.nlm.nih.gov/entrez/query.fcgi?cmd=search&db=gene&term=Tgfb2) | transforming growth factor, beta 2 | 0.0075951 | 10.4 | 8.4 | 1.24 |
| 3 |  | [TGF beta signaling pathway](http://cgap.nci.nih.gov/Pathways/BioCarta/m_tgfbPathway) | [Tgfbr2](http://www.ncbi.nlm.nih.gov/entrez/query.fcgi?cmd=search&db=gene&term=Tgfbr2) | transforming growth factor, beta receptor II | 0.0078495 | 632.88 | 761.54 | 0.83 |
| 4 |  | [TGF beta signaling pathway](http://cgap.nci.nih.gov/Pathways/BioCarta/m_tgfbPathway) | [Smad4](http://www.ncbi.nlm.nih.gov/entrez/query.fcgi?cmd=search&db=gene&term=Smad4) | MAD homolog 4 (Drosophila) | 0.0079572 | 561.82 | 636.93 | 0.88 |
| 5 |  | [TGF beta signaling pathway](http://cgap.nci.nih.gov/Pathways/BioCarta/m_tgfbPathway) | [Apc](http://www.ncbi.nlm.nih.gov/entrez/query.fcgi?cmd=search&db=gene&term=Apc) | adenomatosis polyposis coli | 0.0135079 | 10.53 | 13.4 | 0.79 |
| 6 |  | [TGF beta signaling pathway](http://cgap.nci.nih.gov/Pathways/BioCarta/m_tgfbPathway) | [Smad3](http://www.ncbi.nlm.nih.gov/entrez/query.fcgi?cmd=search&db=gene&term=Smad3) | MAD homolog 3 (Drosophila) | 0.0693902 | 36.62 | 43.06 | 0.85 |
| 7 |  | [TGF beta signaling pathway](http://cgap.nci.nih.gov/Pathways/BioCarta/m_tgfbPathway) | [Tgfbr1](http://www.ncbi.nlm.nih.gov/entrez/query.fcgi?cmd=search&db=gene&term=Tgfbr1) | transforming growth factor, beta receptor I | 0.9560264 | 9.78 | 9.73 | 1.01 |
| 1 | m_eradPathway | [ER¿associated degradation (ERAD) Pathway](http://cgap.nci.nih.gov/Pathways/BioCarta/m_eradPathway) | [Ganab](http://www.ncbi.nlm.nih.gov/entrez/query.fcgi?cmd=search&db=gene&term=Ganab) | alpha glucosidase 2 alpha neutral subunit | 0.0019434 | 8.29 | 10.46 | 0.79 |
| 2 |  | [ER¿associated degradation (ERAD) Pathway](http://cgap.nci.nih.gov/Pathways/BioCarta/m_eradPathway) | [Skp1a](http://www.ncbi.nlm.nih.gov/entrez/query.fcgi?cmd=search&db=gene&term=Skp1a) | S-phase kinase-associated protein 1A | 0.0052038 | 780.22 | 899.69 | 0.87 |
| 3 |  | [ER¿associated degradation (ERAD) Pathway](http://cgap.nci.nih.gov/Pathways/BioCarta/m_eradPathway) | [Man1a](http://www.ncbi.nlm.nih.gov/entrez/query.fcgi?cmd=search&db=gene&term=Man1a) | mannosidase 1, alpha | 0.0299614 | 63.48 | 74.9 | 0.85 |
| 4 |  | [ER¿associated degradation (ERAD) Pathway](http://cgap.nci.nih.gov/Pathways/BioCarta/m_eradPathway) | [Uba2](http://www.ncbi.nlm.nih.gov/entrez/query.fcgi?cmd=search&db=gene&term=Uba2) | ubiquitin-like modifier activating enzyme 2 | 0.0847592 | 1209.73 | 1058.33 | 1.14 |
| 5 |  | [ER¿associated degradation (ERAD) Pathway](http://cgap.nci.nih.gov/Pathways/BioCarta/m_eradPathway) | [Edem1](http://www.ncbi.nlm.nih.gov/entrez/query.fcgi?cmd=search&db=gene&term=Edem1) | ER degradation enhancer, mannosidase alpha-like 1 | 0.5775872 | 452.69 | 463.23 | 0.98 |

Class 1: *K14-TCL1*; Class 2: WT.
