# Supplementary material for: T Cell Leukemia/Lymphoma 1A is essential for mouse epidermal keratinocytes proliferation promoted by insulin-like growth factor 1
Source: PLoS One. 2018 Oct 4;13(10):e0204775. doi: 10.1371/journal.pone.0204775 (PMC6171881; doi:10.1371/journal.pone.0204775)
Supplement: S12 Table — 23 out of 151 investigated gene sets passed the 0.05 significance threshold. LS/KS permutation test found 20 significant gene sets. Efron-Tibshirani's maxmean test found 7 significant gene sets (under 200 permutations). Class 1: Tcl1-/-; Class 2: K14-TCL1. (DOCX) [file pone.0204775.s012.docx]

|  | **Biocarta Pathway** | **Pathway description** | **Gene symbol** | **Description** | **Parametric p-value** | **Geom mean of intensities in class 1** | **Geom mean of intensities in class 2** | **Fold-change** |
| --- | --- | --- | --- | --- | --- | --- | --- | --- |
| 1 | m_nkcellsPathway | [Ras-Independent pathway in NK cell-mediated cytotoxicity](http://cgap.nci.nih.gov/Pathways/BioCarta/m_nkcellsPathway) | [H2-D1](http://www.ncbi.nlm.nih.gov/entrez/query.fcgi?cmd=search&db=gene&term=H2-D1) | histocompatibility 2, D region locus 1 | < 1e-07 | 2751.11 | 66.62 | 41.29 |
| 2 |  | [Ras-Independent pathway in NK cell-mediated cytotoxicity](http://cgap.nci.nih.gov/Pathways/BioCarta/m_nkcellsPathway) | [H2-K1](http://www.ncbi.nlm.nih.gov/entrez/query.fcgi?cmd=search&db=gene&term=H2-K1) | histocompatibility 2, K1, K region | < 1e-07 | 496.79 | 83.71 | 5.93 |
| 3 |  | [Ras-Independent pathway in NK cell-mediated cytotoxicity](http://cgap.nci.nih.gov/Pathways/BioCarta/m_nkcellsPathway) | [Klrc1](http://www.ncbi.nlm.nih.gov/entrez/query.fcgi?cmd=search&db=gene&term=Klrc1) | killer cell lectin-like receptor subfamily C, member 1 | 0.0011221 | 9.86 | 14.14 | 0.7 |
| 4 |  | [Ras-Independent pathway in NK cell-mediated cytotoxicity](http://cgap.nci.nih.gov/Pathways/BioCarta/m_nkcellsPathway) | [Klrd1](http://www.ncbi.nlm.nih.gov/entrez/query.fcgi?cmd=search&db=gene&term=Klrd1) | killer cell lectin-like receptor, subfamily D, member 1 | 0.0012128 | 86.87 | 105.95 | 0.82 |
| 5 |  | [Ras-Independent pathway in NK cell-mediated cytotoxicity](http://cgap.nci.nih.gov/Pathways/BioCarta/m_nkcellsPathway) | [Pik3r1](http://www.ncbi.nlm.nih.gov/entrez/query.fcgi?cmd=search&db=gene&term=Pik3r1) | phosphatidylinositol 3-kinase, regulatory subunit, polypeptide 1 (p85 alpha) | 0.0048997 | 134.94 | 113.16 | 1.19 |
| 6 |  | [Ras-Independent pathway in NK cell-mediated cytotoxicity](http://cgap.nci.nih.gov/Pathways/BioCarta/m_nkcellsPathway) | [Vav1](http://www.ncbi.nlm.nih.gov/entrez/query.fcgi?cmd=search&db=gene&term=Vav1) | vav 1 oncogene | 0.9632314 | 23.69 | 23.74 | 1 |
| 1 | m_TPOPathway | [TPO Signaling Pathway](http://cgap.nci.nih.gov/Pathways/BioCarta/m_TPOPathway) | [Fos](http://www.ncbi.nlm.nih.gov/entrez/query.fcgi?cmd=search&db=gene&term=Fos) | FBJ osteosarcoma oncogene | 1.7e-06 | 2482.37 | 1804.66 | 1.38 |
| 2 |  | [TPO Signaling Pathway](http://cgap.nci.nih.gov/Pathways/BioCarta/m_TPOPathway) | [Stat1](http://www.ncbi.nlm.nih.gov/entrez/query.fcgi?cmd=search&db=gene&term=Stat1) | signal transducer and activator of transcription 1 | 3.4e-06 | 589.81 | 804.27 | 0.73 |
| 3 |  | [TPO Signaling Pathway](http://cgap.nci.nih.gov/Pathways/BioCarta/m_TPOPathway) | [Csnk2a2](http://www.ncbi.nlm.nih.gov/entrez/query.fcgi?cmd=search&db=gene&term=Csnk2a2) | casein kinase 2, alpha prime polypeptide | 5.3e-06 | 2035.41 | 1559.16 | 1.31 |
| 4 |  | [TPO Signaling Pathway](http://cgap.nci.nih.gov/Pathways/BioCarta/m_TPOPathway) | [Prkcb](http://www.ncbi.nlm.nih.gov/entrez/query.fcgi?cmd=search&db=gene&term=Prkcb) | protein kinase C, beta | 7e-06 | 231.8 | 173.52 | 1.34 |
| 5 |  | [TPO Signaling Pathway](http://cgap.nci.nih.gov/Pathways/BioCarta/m_TPOPathway) | [Sos1](http://www.ncbi.nlm.nih.gov/entrez/query.fcgi?cmd=search&db=gene&term=Sos1) | son of sevenless homolog 1 (Drosophila) | 0.0001904 | 82.16 | 62.98 | 1.3 |
| 6 |  | [TPO Signaling Pathway](http://cgap.nci.nih.gov/Pathways/BioCarta/m_TPOPathway) | [Jun](http://www.ncbi.nlm.nih.gov/entrez/query.fcgi?cmd=search&db=gene&term=Jun) | Jun oncogene | 0.0013368 | 325.38 | 270.82 | 1.2 |
| 7 |  | [TPO Signaling Pathway](http://cgap.nci.nih.gov/Pathways/BioCarta/m_TPOPathway) | [Stat5a](http://www.ncbi.nlm.nih.gov/entrez/query.fcgi?cmd=search&db=gene&term=Stat5a) | signal transducer and activator of transcription 5A | 0.0038094 | 76.75 | 63.97 | 1.2 |
| 8 |  | [TPO Signaling Pathway](http://cgap.nci.nih.gov/Pathways/BioCarta/m_TPOPathway) | [Pik3r1](http://www.ncbi.nlm.nih.gov/entrez/query.fcgi?cmd=search&db=gene&term=Pik3r1) | phosphatidylinositol 3-kinase, regulatory subunit, polypeptide 1 (p85 alpha) | 0.0048997 | 134.94 | 113.16 | 1.19 |
| 9 |  | [TPO Signaling Pathway](http://cgap.nci.nih.gov/Pathways/BioCarta/m_TPOPathway) | [Csnk2a1](http://www.ncbi.nlm.nih.gov/entrez/query.fcgi?cmd=search&db=gene&term=Csnk2a1) | casein kinase 2, alpha 1 polypeptide | 0.0158401 | 200.24 | 255.89 | 0.78 |
| 10 |  | [TPO Signaling Pathway](http://cgap.nci.nih.gov/Pathways/BioCarta/m_TPOPathway) | [Pik3cg](http://www.ncbi.nlm.nih.gov/entrez/query.fcgi?cmd=search&db=gene&term=Pik3cg) | phosphoinositide-3-kinase, catalytic, gamma polypeptide | 0.2441421 | 18.24 | 16.25 | 1.12 |
| 11 |  | [TPO Signaling Pathway](http://cgap.nci.nih.gov/Pathways/BioCarta/m_TPOPathway) | [Mpl](http://www.ncbi.nlm.nih.gov/entrez/query.fcgi?cmd=search&db=gene&term=Mpl) | myeloproliferative leukemia virus oncogene | 0.919742 | 22.22 | 22.39 | 0.99 |
| 1 | m_pdgfPathway | [PDGF Signaling Pathway](http://cgap.nci.nih.gov/Pathways/BioCarta/m_pdgfPathway) | [Fos](http://www.ncbi.nlm.nih.gov/entrez/query.fcgi?cmd=search&db=gene&term=Fos) | FBJ osteosarcoma oncogene | 1.7e-06 | 2482.37 | 1804.66 | 1.38 |
| 2 |  | [PDGF Signaling Pathway](http://cgap.nci.nih.gov/Pathways/BioCarta/m_pdgfPathway) | [Stat1](http://www.ncbi.nlm.nih.gov/entrez/query.fcgi?cmd=search&db=gene&term=Stat1) | signal transducer and activator of transcription 1 | 3.4e-06 | 589.81 | 804.27 | 0.73 |
| 3 |  | [PDGF Signaling Pathway](http://cgap.nci.nih.gov/Pathways/BioCarta/m_pdgfPathway) | [Csnk2a2](http://www.ncbi.nlm.nih.gov/entrez/query.fcgi?cmd=search&db=gene&term=Csnk2a2) | casein kinase 2, alpha prime polypeptide | 5.3e-06 | 2035.41 | 1559.16 | 1.31 |
| 4 |  | [PDGF Signaling Pathway](http://cgap.nci.nih.gov/Pathways/BioCarta/m_pdgfPathway) | [Prkcb](http://www.ncbi.nlm.nih.gov/entrez/query.fcgi?cmd=search&db=gene&term=Prkcb) | protein kinase C, beta | 7e-06 | 231.8 | 173.52 | 1.34 |
| 5 |  | [PDGF Signaling Pathway](http://cgap.nci.nih.gov/Pathways/BioCarta/m_pdgfPathway) | [Sos1](http://www.ncbi.nlm.nih.gov/entrez/query.fcgi?cmd=search&db=gene&term=Sos1) | son of sevenless homolog 1 (Drosophila) | 0.0001904 | 82.16 | 62.98 | 1.3 |
| 6 |  | [PDGF Signaling Pathway](http://cgap.nci.nih.gov/Pathways/BioCarta/m_pdgfPathway) | [Jun](http://www.ncbi.nlm.nih.gov/entrez/query.fcgi?cmd=search&db=gene&term=Jun) | Jun oncogene | 0.0013368 | 325.38 | 270.82 | 1.2 |
| 7 |  | [PDGF Signaling Pathway](http://cgap.nci.nih.gov/Pathways/BioCarta/m_pdgfPathway) | [Stat5a](http://www.ncbi.nlm.nih.gov/entrez/query.fcgi?cmd=search&db=gene&term=Stat5a) | signal transducer and activator of transcription 5A | 0.0038094 | 76.75 | 63.97 | 1.2 |
| 8 |  | [PDGF Signaling Pathway](http://cgap.nci.nih.gov/Pathways/BioCarta/m_pdgfPathway) | [Map2k4](http://www.ncbi.nlm.nih.gov/entrez/query.fcgi?cmd=search&db=gene&term=Map2k4) | mitogen-activated protein kinase kinase 4 | 0.0044999 | 400.03 | 341.42 | 1.17 |
| 9 |  | [PDGF Signaling Pathway](http://cgap.nci.nih.gov/Pathways/BioCarta/m_pdgfPathway) | [Pik3r1](http://www.ncbi.nlm.nih.gov/entrez/query.fcgi?cmd=search&db=gene&term=Pik3r1) | phosphatidylinositol 3-kinase, regulatory subunit, polypeptide 1 (p85 alpha) | 0.0048997 | 134.94 | 113.16 | 1.19 |
| 10 |  | [PDGF Signaling Pathway](http://cgap.nci.nih.gov/Pathways/BioCarta/m_pdgfPathway) | [Csnk2a1](http://www.ncbi.nlm.nih.gov/entrez/query.fcgi?cmd=search&db=gene&term=Csnk2a1) | casein kinase 2, alpha 1 polypeptide | 0.0158401 | 200.24 | 255.89 | 0.78 |
| 11 |  | [PDGF Signaling Pathway](http://cgap.nci.nih.gov/Pathways/BioCarta/m_pdgfPathway) | [Jak1](http://www.ncbi.nlm.nih.gov/entrez/query.fcgi?cmd=search&db=gene&term=Jak1) | Janus kinase 1 | 0.1004723 | 151.14 | 128.89 | 1.17 |
| 12 |  | [PDGF Signaling Pathway](http://cgap.nci.nih.gov/Pathways/BioCarta/m_pdgfPathway) | [Pik3cg](http://www.ncbi.nlm.nih.gov/entrez/query.fcgi?cmd=search&db=gene&term=Pik3cg) | phosphoinositide-3-kinase, catalytic, gamma polypeptide | 0.2441421 | 18.24 | 16.25 | 1.12 |
| 13 |  | [PDGF Signaling Pathway](http://cgap.nci.nih.gov/Pathways/BioCarta/m_pdgfPathway) | [Mapk8](http://www.ncbi.nlm.nih.gov/entrez/query.fcgi?cmd=search&db=gene&term=Mapk8) | mitogen-activated protein kinase 8 | 0.4053373 | 29.15 | 31.06 | 0.94 |
| 1 | m_egfPathway | [EGF Signaling Pathway](http://cgap.nci.nih.gov/Pathways/BioCarta/m_egfPathway) | [Fos](http://www.ncbi.nlm.nih.gov/entrez/query.fcgi?cmd=search&db=gene&term=Fos) | FBJ osteosarcoma oncogene | 1.7e-06 | 2482.37 | 1804.66 | 1.38 |
| 2 |  | [EGF Signaling Pathway](http://cgap.nci.nih.gov/Pathways/BioCarta/m_egfPathway) | [Stat1](http://www.ncbi.nlm.nih.gov/entrez/query.fcgi?cmd=search&db=gene&term=Stat1) | signal transducer and activator of transcription 1 | 3.4e-06 | 589.81 | 804.27 | 0.73 |
| 3 |  | [EGF Signaling Pathway](http://cgap.nci.nih.gov/Pathways/BioCarta/m_egfPathway) | [Csnk2a2](http://www.ncbi.nlm.nih.gov/entrez/query.fcgi?cmd=search&db=gene&term=Csnk2a2) | casein kinase 2, alpha prime polypeptide | 5.3e-06 | 2035.41 | 1559.16 | 1.31 |
| 4 |  | [EGF Signaling Pathway](http://cgap.nci.nih.gov/Pathways/BioCarta/m_egfPathway) | [Prkcb](http://www.ncbi.nlm.nih.gov/entrez/query.fcgi?cmd=search&db=gene&term=Prkcb) | protein kinase C, beta | 7e-06 | 231.8 | 173.52 | 1.34 |
| 5 |  | [EGF Signaling Pathway](http://cgap.nci.nih.gov/Pathways/BioCarta/m_egfPathway) | [Sos1](http://www.ncbi.nlm.nih.gov/entrez/query.fcgi?cmd=search&db=gene&term=Sos1) | son of sevenless homolog 1 (Drosophila) | 0.0001904 | 82.16 | 62.98 | 1.3 |
| 6 |  | [EGF Signaling Pathway](http://cgap.nci.nih.gov/Pathways/BioCarta/m_egfPathway) | [Jun](http://www.ncbi.nlm.nih.gov/entrez/query.fcgi?cmd=search&db=gene&term=Jun) | Jun oncogene | 0.0013368 | 325.38 | 270.82 | 1.2 |
| 7 |  | [EGF Signaling Pathway](http://cgap.nci.nih.gov/Pathways/BioCarta/m_egfPathway) | [Stat5a](http://www.ncbi.nlm.nih.gov/entrez/query.fcgi?cmd=search&db=gene&term=Stat5a) | signal transducer and activator of transcription 5A | 0.0038094 | 76.75 | 63.97 | 1.2 |
| 8 |  | [EGF Signaling Pathway](http://cgap.nci.nih.gov/Pathways/BioCarta/m_egfPathway) | [Map2k4](http://www.ncbi.nlm.nih.gov/entrez/query.fcgi?cmd=search&db=gene&term=Map2k4) | mitogen-activated protein kinase kinase 4 | 0.0044999 | 400.03 | 341.42 | 1.17 |
| 9 |  | [EGF Signaling Pathway](http://cgap.nci.nih.gov/Pathways/BioCarta/m_egfPathway) | [Pik3r1](http://www.ncbi.nlm.nih.gov/entrez/query.fcgi?cmd=search&db=gene&term=Pik3r1) | phosphatidylinositol 3-kinase, regulatory subunit, polypeptide 1 (p85 alpha) | 0.0048997 | 134.94 | 113.16 | 1.19 |
| 10 |  | [EGF Signaling Pathway](http://cgap.nci.nih.gov/Pathways/BioCarta/m_egfPathway) | [Csnk2a1](http://www.ncbi.nlm.nih.gov/entrez/query.fcgi?cmd=search&db=gene&term=Csnk2a1) | casein kinase 2, alpha 1 polypeptide | 0.0158401 | 200.24 | 255.89 | 0.78 |
| 11 |  | [EGF Signaling Pathway](http://cgap.nci.nih.gov/Pathways/BioCarta/m_egfPathway) | [Jak1](http://www.ncbi.nlm.nih.gov/entrez/query.fcgi?cmd=search&db=gene&term=Jak1) | Janus kinase 1 | 0.1004723 | 151.14 | 128.89 | 1.17 |
| 12 |  | [EGF Signaling Pathway](http://cgap.nci.nih.gov/Pathways/BioCarta/m_egfPathway) | [Mapk8](http://www.ncbi.nlm.nih.gov/entrez/query.fcgi?cmd=search&db=gene&term=Mapk8) | mitogen-activated protein kinase 8 | 0.4053373 | 29.15 | 31.06 | 0.94 |
| 13 |  | [EGF Signaling Pathway](http://cgap.nci.nih.gov/Pathways/BioCarta/m_egfPathway) | [Egf](http://www.ncbi.nlm.nih.gov/entrez/query.fcgi?cmd=search&db=gene&term=Egf) | epidermal growth factor | 0.4812978 | 12.86 | 12.25 | 1.05 |
| 1 | m_ctlPathway | [CTL mediated immune response against target cells](http://cgap.nci.nih.gov/Pathways/BioCarta/m_ctlPathway) | [H2-D1](http://www.ncbi.nlm.nih.gov/entrez/query.fcgi?cmd=search&db=gene&term=H2-D1) | histocompatibility 2, D region locus 1 | < 1e-07 | 2751.11 | 66.62 | 41.29 |
| 2 |  | [CTL mediated immune response against target cells](http://cgap.nci.nih.gov/Pathways/BioCarta/m_ctlPathway) | [H2-K1](http://www.ncbi.nlm.nih.gov/entrez/query.fcgi?cmd=search&db=gene&term=H2-K1) | histocompatibility 2, K1, K region | < 1e-07 | 496.79 | 83.71 | 5.93 |
| 3 |  | [CTL mediated immune response against target cells](http://cgap.nci.nih.gov/Pathways/BioCarta/m_ctlPathway) | [Icam1](http://www.ncbi.nlm.nih.gov/entrez/query.fcgi?cmd=search&db=gene&term=Icam1) | intercellular adhesion molecule 1 | 3.7e-05 | 128.64 | 161.75 | 0.8 |
| 4 |  | [CTL mediated immune response against target cells](http://cgap.nci.nih.gov/Pathways/BioCarta/m_ctlPathway) | [Cd3d](http://www.ncbi.nlm.nih.gov/entrez/query.fcgi?cmd=search&db=gene&term=Cd3d) | CD3 antigen, delta polypeptide | 4.52e-05 | 116.66 | 154.41 | 0.76 |
| 5 |  | [CTL mediated immune response against target cells](http://cgap.nci.nih.gov/Pathways/BioCarta/m_ctlPathway) | [Itgb2](http://www.ncbi.nlm.nih.gov/entrez/query.fcgi?cmd=search&db=gene&term=Itgb2) | integrin beta 2 | 0.0103169 | 75.8 | 63.72 | 1.19 |
| 6 |  | [CTL mediated immune response against target cells](http://cgap.nci.nih.gov/Pathways/BioCarta/m_ctlPathway) | [Cd3g](http://www.ncbi.nlm.nih.gov/entrez/query.fcgi?cmd=search&db=gene&term=Cd3g) | CD3 antigen, gamma polypeptide | 0.0257662 | 228.71 | 250.04 | 0.91 |
| 7 |  | [CTL mediated immune response against target cells](http://cgap.nci.nih.gov/Pathways/BioCarta/m_ctlPathway) | [Prf1](http://www.ncbi.nlm.nih.gov/entrez/query.fcgi?cmd=search&db=gene&term=Prf1) | perforin 1 (pore forming protein) | 0.071834 | 72.94 | 79.96 | 0.91 |
| 8 |  | [CTL mediated immune response against target cells](http://cgap.nci.nih.gov/Pathways/BioCarta/m_ctlPathway) | [Gzmb](http://www.ncbi.nlm.nih.gov/entrez/query.fcgi?cmd=search&db=gene&term=Gzmb) | granzyme B | 0.1678509 | 27.4 | 29.91 | 0.92 |
| 9 |  | [CTL mediated immune response against target cells](http://cgap.nci.nih.gov/Pathways/BioCarta/m_ctlPathway) | [Tcra](http://www.ncbi.nlm.nih.gov/entrez/query.fcgi?cmd=search&db=gene&term=Tcra) | T-cell receptor alpha chain | 0.4497809 | 30.87 | 29.96 | 1.03 |
| 10 |  | [CTL mediated immune response against target cells](http://cgap.nci.nih.gov/Pathways/BioCarta/m_ctlPathway) | [Cd247](http://www.ncbi.nlm.nih.gov/entrez/query.fcgi?cmd=search&db=gene&term=Cd247) | CD247 antigen | 0.9910032 | 12.93 | 12.94 | 1 |
| 1 | m_mef2dPathway | [Role of MEF2D in T-cell Apoptosis](http://cgap.nci.nih.gov/Pathways/BioCarta/m_mef2dPathway) | [Capn2](http://www.ncbi.nlm.nih.gov/entrez/query.fcgi?cmd=search&db=gene&term=Capn2) | calpain 2 | 2.1e-06 | 266.92 | 380.59 | 0.7 |
| 2 |  | [Role of MEF2D in T-cell Apoptosis](http://cgap.nci.nih.gov/Pathways/BioCarta/m_mef2dPathway) | [Prkcb](http://www.ncbi.nlm.nih.gov/entrez/query.fcgi?cmd=search&db=gene&term=Prkcb) | protein kinase C, beta | 7e-06 | 231.8 | 173.52 | 1.34 |
| 3 |  | [Role of MEF2D in T-cell Apoptosis](http://cgap.nci.nih.gov/Pathways/BioCarta/m_mef2dPathway) | [Capns1](http://www.ncbi.nlm.nih.gov/entrez/query.fcgi?cmd=search&db=gene&term=Capns1) | calpain, small subunit 1 | 1.19e-05 | 728.59 | 919.26 | 0.79 |
| 4 |  | [Role of MEF2D in T-cell Apoptosis](http://cgap.nci.nih.gov/Pathways/BioCarta/m_mef2dPathway) | [Nfatc2](http://www.ncbi.nlm.nih.gov/entrez/query.fcgi?cmd=search&db=gene&term=Nfatc2) | nuclear factor of activated T-cells, cytoplasmic, calcineurin-dependent 2 | 0.0344999 | 17.55 | 14.9 | 1.18 |
| 5 |  | [Role of MEF2D in T-cell Apoptosis](http://cgap.nci.nih.gov/Pathways/BioCarta/m_mef2dPathway) | [Tcra](http://www.ncbi.nlm.nih.gov/entrez/query.fcgi?cmd=search&db=gene&term=Tcra) | T-cell receptor alpha chain | 0.4497809 | 30.87 | 29.96 | 1.03 |
| 1 | m_epoPathway | [EPO Signaling Pathway](http://cgap.nci.nih.gov/Pathways/BioCarta/m_epoPathway) | [Fos](http://www.ncbi.nlm.nih.gov/entrez/query.fcgi?cmd=search&db=gene&term=Fos) | FBJ osteosarcoma oncogene | 1.7e-06 | 2482.37 | 1804.66 | 1.38 |
| 2 |  | [EPO Signaling Pathway](http://cgap.nci.nih.gov/Pathways/BioCarta/m_epoPathway) | [Csnk2a2](http://www.ncbi.nlm.nih.gov/entrez/query.fcgi?cmd=search&db=gene&term=Csnk2a2) | casein kinase 2, alpha prime polypeptide | 5.3e-06 | 2035.41 | 1559.16 | 1.31 |
| 3 |  | [EPO Signaling Pathway](http://cgap.nci.nih.gov/Pathways/BioCarta/m_epoPathway) | [Sos1](http://www.ncbi.nlm.nih.gov/entrez/query.fcgi?cmd=search&db=gene&term=Sos1) | son of sevenless homolog 1 (Drosophila) | 0.0001904 | 82.16 | 62.98 | 1.3 |
| 4 |  | [EPO Signaling Pathway](http://cgap.nci.nih.gov/Pathways/BioCarta/m_epoPathway) | [Jun](http://www.ncbi.nlm.nih.gov/entrez/query.fcgi?cmd=search&db=gene&term=Jun) | Jun oncogene | 0.0013368 | 325.38 | 270.82 | 1.2 |
| 5 |  | [EPO Signaling Pathway](http://cgap.nci.nih.gov/Pathways/BioCarta/m_epoPathway) | [Stat5a](http://www.ncbi.nlm.nih.gov/entrez/query.fcgi?cmd=search&db=gene&term=Stat5a) | signal transducer and activator of transcription 5A | 0.0038094 | 76.75 | 63.97 | 1.2 |
| 6 |  | [EPO Signaling Pathway](http://cgap.nci.nih.gov/Pathways/BioCarta/m_epoPathway) | [Csnk2a1](http://www.ncbi.nlm.nih.gov/entrez/query.fcgi?cmd=search&db=gene&term=Csnk2a1) | casein kinase 2, alpha 1 polypeptide | 0.0158401 | 200.24 | 255.89 | 0.78 |
| 7 |  | [EPO Signaling Pathway](http://cgap.nci.nih.gov/Pathways/BioCarta/m_epoPathway) | [Mapk8](http://www.ncbi.nlm.nih.gov/entrez/query.fcgi?cmd=search&db=gene&term=Mapk8) | mitogen-activated protein kinase 8 | 0.4053373 | 29.15 | 31.06 | 0.94 |
| 1 | m_insulinPathway | [Insulin Signaling Pathway](http://cgap.nci.nih.gov/Pathways/BioCarta/m_insulinPathway) | [Fos](http://www.ncbi.nlm.nih.gov/entrez/query.fcgi?cmd=search&db=gene&term=Fos) | FBJ osteosarcoma oncogene | 1.7e-06 | 2482.37 | 1804.66 | 1.38 |
| 2 |  | [Insulin Signaling Pathway](http://cgap.nci.nih.gov/Pathways/BioCarta/m_insulinPathway) | [Csnk2a2](http://www.ncbi.nlm.nih.gov/entrez/query.fcgi?cmd=search&db=gene&term=Csnk2a2) | casein kinase 2, alpha prime polypeptide | 5.3e-06 | 2035.41 | 1559.16 | 1.31 |
| 3 |  | [Insulin Signaling Pathway](http://cgap.nci.nih.gov/Pathways/BioCarta/m_insulinPathway) | [Irs1](http://www.ncbi.nlm.nih.gov/entrez/query.fcgi?cmd=search&db=gene&term=Irs1) | insulin receptor substrate 1 | 3.3e-05 | 652.96 | 495.34 | 1.32 |
| 4 |  | [Insulin Signaling Pathway](http://cgap.nci.nih.gov/Pathways/BioCarta/m_insulinPathway) | [Sos1](http://www.ncbi.nlm.nih.gov/entrez/query.fcgi?cmd=search&db=gene&term=Sos1) | son of sevenless homolog 1 (Drosophila) | 0.0001904 | 82.16 | 62.98 | 1.3 |
| 5 |  | [Insulin Signaling Pathway](http://cgap.nci.nih.gov/Pathways/BioCarta/m_insulinPathway) | [Jun](http://www.ncbi.nlm.nih.gov/entrez/query.fcgi?cmd=search&db=gene&term=Jun) | Jun oncogene | 0.0013368 | 325.38 | 270.82 | 1.2 |
| 6 |  | [Insulin Signaling Pathway](http://cgap.nci.nih.gov/Pathways/BioCarta/m_insulinPathway) | [Pik3r1](http://www.ncbi.nlm.nih.gov/entrez/query.fcgi?cmd=search&db=gene&term=Pik3r1) | phosphatidylinositol 3-kinase, regulatory subunit, polypeptide 1 (p85 alpha) | 0.0048997 | 134.94 | 113.16 | 1.19 |
| 7 |  | [Insulin Signaling Pathway](http://cgap.nci.nih.gov/Pathways/BioCarta/m_insulinPathway) | [Csnk2a1](http://www.ncbi.nlm.nih.gov/entrez/query.fcgi?cmd=search&db=gene&term=Csnk2a1) | casein kinase 2, alpha 1 polypeptide | 0.0158401 | 200.24 | 255.89 | 0.78 |
| 8 |  | [Insulin Signaling Pathway](http://cgap.nci.nih.gov/Pathways/BioCarta/m_insulinPathway) | [Ptpn11](http://www.ncbi.nlm.nih.gov/entrez/query.fcgi?cmd=search&db=gene&term=Ptpn11) | protein tyrosine phosphatase, non-receptor type 11 | 0.0251854 | 49.82 | 64.25 | 0.78 |
| 9 |  | [Insulin Signaling Pathway](http://cgap.nci.nih.gov/Pathways/BioCarta/m_insulinPathway) | [Pik3cg](http://www.ncbi.nlm.nih.gov/entrez/query.fcgi?cmd=search&db=gene&term=Pik3cg) | phosphoinositide-3-kinase, catalytic, gamma polypeptide | 0.2441421 | 18.24 | 16.25 | 1.12 |
| 10 |  | [Insulin Signaling Pathway](http://cgap.nci.nih.gov/Pathways/BioCarta/m_insulinPathway) | [Mapk8](http://www.ncbi.nlm.nih.gov/entrez/query.fcgi?cmd=search&db=gene&term=Mapk8) | mitogen-activated protein kinase 8 | 0.4053373 | 29.15 | 31.06 | 0.94 |
| 1 | m_igf1Pathway | [IGF-1 Signaling Pathway](http://cgap.nci.nih.gov/Pathways/BioCarta/m_igf1Pathway) | [Fos](http://www.ncbi.nlm.nih.gov/entrez/query.fcgi?cmd=search&db=gene&term=Fos) | FBJ osteosarcoma oncogene | 1.7e-06 | 2482.37 | 1804.66 | 1.38 |
| 2 |  | [IGF-1 Signaling Pathway](http://cgap.nci.nih.gov/Pathways/BioCarta/m_igf1Pathway) | [Csnk2a2](http://www.ncbi.nlm.nih.gov/entrez/query.fcgi?cmd=search&db=gene&term=Csnk2a2) | casein kinase 2, alpha prime polypeptide | 5.3e-06 | 2035.41 | 1559.16 | 1.31 |
| 3 |  | [IGF-1 Signaling Pathway](http://cgap.nci.nih.gov/Pathways/BioCarta/m_igf1Pathway) | [Irs1](http://www.ncbi.nlm.nih.gov/entrez/query.fcgi?cmd=search&db=gene&term=Irs1) | insulin receptor substrate 1 | 3.3e-05 | 652.96 | 495.34 | 1.32 |
| 4 |  | [IGF-1 Signaling Pathway](http://cgap.nci.nih.gov/Pathways/BioCarta/m_igf1Pathway) | [Sos1](http://www.ncbi.nlm.nih.gov/entrez/query.fcgi?cmd=search&db=gene&term=Sos1) | son of sevenless homolog 1 (Drosophila) | 0.0001904 | 82.16 | 62.98 | 1.3 |
| 5 |  | [IGF-1 Signaling Pathway](http://cgap.nci.nih.gov/Pathways/BioCarta/m_igf1Pathway) | [Jun](http://www.ncbi.nlm.nih.gov/entrez/query.fcgi?cmd=search&db=gene&term=Jun) | Jun oncogene | 0.0013368 | 325.38 | 270.82 | 1.2 |
| 6 |  | [IGF-1 Signaling Pathway](http://cgap.nci.nih.gov/Pathways/BioCarta/m_igf1Pathway) | [Pik3r1](http://www.ncbi.nlm.nih.gov/entrez/query.fcgi?cmd=search&db=gene&term=Pik3r1) | phosphatidylinositol 3-kinase, regulatory subunit, polypeptide 1 (p85 alpha) | 0.0048997 | 134.94 | 113.16 | 1.19 |
| 7 |  | [IGF-1 Signaling Pathway](http://cgap.nci.nih.gov/Pathways/BioCarta/m_igf1Pathway) | [Igf1r](http://www.ncbi.nlm.nih.gov/entrez/query.fcgi?cmd=search&db=gene&term=Igf1r) | insulin-like growth factor I receptor | 0.0098994 | 33.81 | 28.83 | 1.17 |
| 8 |  | [IGF-1 Signaling Pathway](http://cgap.nci.nih.gov/Pathways/BioCarta/m_igf1Pathway) | [Csnk2a1](http://www.ncbi.nlm.nih.gov/entrez/query.fcgi?cmd=search&db=gene&term=Csnk2a1) | casein kinase 2, alpha 1 polypeptide | 0.0158401 | 200.24 | 255.89 | 0.78 |
| 9 |  | [IGF-1 Signaling Pathway](http://cgap.nci.nih.gov/Pathways/BioCarta/m_igf1Pathway) | [Ptpn11](http://www.ncbi.nlm.nih.gov/entrez/query.fcgi?cmd=search&db=gene&term=Ptpn11) | protein tyrosine phosphatase, non-receptor type 11 | 0.0251854 | 49.82 | 64.25 | 0.78 |
| 10 |  | [IGF-1 Signaling Pathway](http://cgap.nci.nih.gov/Pathways/BioCarta/m_igf1Pathway) | [Pik3cg](http://www.ncbi.nlm.nih.gov/entrez/query.fcgi?cmd=search&db=gene&term=Pik3cg) | phosphoinositide-3-kinase, catalytic, gamma polypeptide | 0.2441421 | 18.24 | 16.25 | 1.12 |
| 11 |  | [IGF-1 Signaling Pathway](http://cgap.nci.nih.gov/Pathways/BioCarta/m_igf1Pathway) | [Mapk8](http://www.ncbi.nlm.nih.gov/entrez/query.fcgi?cmd=search&db=gene&term=Mapk8) | mitogen-activated protein kinase 8 | 0.4053373 | 29.15 | 31.06 | 0.94 |
| 1 | m_wntPathway | [WNT Signaling Pathway](http://cgap.nci.nih.gov/Pathways/BioCarta/m_wntPathway) | [Csnk2a2](http://www.ncbi.nlm.nih.gov/entrez/query.fcgi?cmd=search&db=gene&term=Csnk2a2) | casein kinase 2, alpha prime polypeptide | 5.3e-06 | 2035.41 | 1559.16 | 1.31 |
| 2 |  | [WNT Signaling Pathway](http://cgap.nci.nih.gov/Pathways/BioCarta/m_wntPathway) | [Ctnnb1](http://www.ncbi.nlm.nih.gov/entrez/query.fcgi?cmd=search&db=gene&term=Ctnnb1) | catenin (cadherin associated protein), beta 1 | 0.0001397 | 1041.24 | 853.48 | 1.22 |
| 3 |  | [WNT Signaling Pathway](http://cgap.nci.nih.gov/Pathways/BioCarta/m_wntPathway) | [Smad4](http://www.ncbi.nlm.nih.gov/entrez/query.fcgi?cmd=search&db=gene&term=Smad4) | MAD homolog 4 (Drosophila) | 0.0002321 | 463.34 | 561.82 | 0.82 |
| 4 |  | [WNT Signaling Pathway](http://cgap.nci.nih.gov/Pathways/BioCarta/m_wntPathway) | [Ccnd1](http://www.ncbi.nlm.nih.gov/entrez/query.fcgi?cmd=search&db=gene&term=Ccnd1) | cyclin D1 | 0.0003833 | 420.26 | 519.9 | 0.81 |
| 5 |  | [WNT Signaling Pathway](http://cgap.nci.nih.gov/Pathways/BioCarta/m_wntPathway) | [Tle1](http://www.ncbi.nlm.nih.gov/entrez/query.fcgi?cmd=search&db=gene&term=Tle1) | transducin-like enhancer of split 1, homolog of Drosophila E(spl) | 0.0018859 | 83.77 | 104.76 | 0.8 |
| 6 |  | [WNT Signaling Pathway](http://cgap.nci.nih.gov/Pathways/BioCarta/m_wntPathway) | [Apc](http://www.ncbi.nlm.nih.gov/entrez/query.fcgi?cmd=search&db=gene&term=Apc) | adenomatosis polyposis coli | 0.0100428 | 12.59 | 10.53 | 1.2 |
| 7 |  | [WNT Signaling Pathway](http://cgap.nci.nih.gov/Pathways/BioCarta/m_wntPathway) | [Csnk2a1](http://www.ncbi.nlm.nih.gov/entrez/query.fcgi?cmd=search&db=gene&term=Csnk2a1) | casein kinase 2, alpha 1 polypeptide | 0.0158401 | 200.24 | 255.89 | 0.78 |
| 8 |  | [WNT Signaling Pathway](http://cgap.nci.nih.gov/Pathways/BioCarta/m_wntPathway) | [Btrc](http://www.ncbi.nlm.nih.gov/entrez/query.fcgi?cmd=search&db=gene&term=Btrc) | beta-transducin repeat containing protein | 0.3851419 | 14.47 | 15.45 | 0.94 |
| 1 | m_g2Pathway | [Cell Cycle: G2/M Checkpoint](http://cgap.nci.nih.gov/Pathways/BioCarta/m_g2Pathway) | [Wee1](http://www.ncbi.nlm.nih.gov/entrez/query.fcgi?cmd=search&db=gene&term=Wee1) | WEE 1 homolog 1 (S. pombe) | 6.9e-06 | 274.9 | 357.86 | 0.77 |
| 2 |  | [Cell Cycle: G2/M Checkpoint](http://cgap.nci.nih.gov/Pathways/BioCarta/m_g2Pathway) | [Ccnb1](http://www.ncbi.nlm.nih.gov/entrez/query.fcgi?cmd=search&db=gene&term=Ccnb1) | cyclin B1 | 0.0002607 | 155.23 | 132.48 | 1.17 |
| 3 |  | [Cell Cycle: G2/M Checkpoint](http://cgap.nci.nih.gov/Pathways/BioCarta/m_g2Pathway) | [Brca1](http://www.ncbi.nlm.nih.gov/entrez/query.fcgi?cmd=search&db=gene&term=Brca1) | breast cancer 1 | 0.0010201 | 15.04 | 12.37 | 1.22 |
| 4 |  | [Cell Cycle: G2/M Checkpoint](http://cgap.nci.nih.gov/Pathways/BioCarta/m_g2Pathway) | [Chek1](http://www.ncbi.nlm.nih.gov/entrez/query.fcgi?cmd=search&db=gene&term=Chek1) | checkpoint kinase 1 homolog (S. pombe) | 0.0012346 | 50.49 | 60.12 | 0.84 |
| 5 |  | [Cell Cycle: G2/M Checkpoint](http://cgap.nci.nih.gov/Pathways/BioCarta/m_g2Pathway) | [Cdk1](http://www.ncbi.nlm.nih.gov/entrez/query.fcgi?cmd=search&db=gene&term=Cdk1) | cyclin-dependent kinase 1 | 0.0013225 | 246.97 | 218.93 | 1.13 |
| 6 |  | [Cell Cycle: G2/M Checkpoint](http://cgap.nci.nih.gov/Pathways/BioCarta/m_g2Pathway) | [Plk1](http://www.ncbi.nlm.nih.gov/entrez/query.fcgi?cmd=search&db=gene&term=Plk1) | polo-like kinase 1 (Drosophila) | 0.0024306 | 190.3 | 169.96 | 1.12 |
| 7 |  | [Cell Cycle: G2/M Checkpoint](http://cgap.nci.nih.gov/Pathways/BioCarta/m_g2Pathway) | [Cdc25c](http://www.ncbi.nlm.nih.gov/entrez/query.fcgi?cmd=search&db=gene&term=Cdc25c) | cell division cycle 25 homolog C (S. pombe) | 0.012068 | 55.1 | 49.55 | 1.11 |
| 8 |  | [Cell Cycle: G2/M Checkpoint](http://cgap.nci.nih.gov/Pathways/BioCarta/m_g2Pathway) | [Gadd45a](http://www.ncbi.nlm.nih.gov/entrez/query.fcgi?cmd=search&db=gene&term=Gadd45a) | growth arrest and DNA-damage-inducible 45 alpha | 0.018802 | 75.13 | 66.3 | 1.13 |
| 9 |  | [Cell Cycle: G2/M Checkpoint](http://cgap.nci.nih.gov/Pathways/BioCarta/m_g2Pathway) | [Trp53](http://www.ncbi.nlm.nih.gov/entrez/query.fcgi?cmd=search&db=gene&term=Trp53) | transformation related protein 53 | 0.1390267 | 12.39 | 11.53 | 1.07 |
| 1 | m_akap95Pathway | [AKAP95 role in mitosis and chromosome dynamics](http://cgap.nci.nih.gov/Pathways/BioCarta/m_akap95Pathway) | [Ccnb1](http://www.ncbi.nlm.nih.gov/entrez/query.fcgi?cmd=search&db=gene&term=Ccnb1) | cyclin B1 | 0.0002607 | 155.23 | 132.48 | 1.17 |
| 2 |  | [AKAP95 role in mitosis and chromosome dynamics](http://cgap.nci.nih.gov/Pathways/BioCarta/m_akap95Pathway) | [Ddx5](http://www.ncbi.nlm.nih.gov/entrez/query.fcgi?cmd=search&db=gene&term=Ddx5) | DEAD (Asp-Glu-Ala-Asp) box polypeptide 5 | 0.0010293 | 2543.94 | 2922.46 | 0.87 |
| 3 |  | [AKAP95 role in mitosis and chromosome dynamics](http://cgap.nci.nih.gov/Pathways/BioCarta/m_akap95Pathway) | [Ncapd2](http://www.ncbi.nlm.nih.gov/entrez/query.fcgi?cmd=search&db=gene&term=Ncapd2) | non-SMC condensin I complex, subunit D2 | 0.0013022 | 260.95 | 223.32 | 1.17 |
| 4 |  | [AKAP95 role in mitosis and chromosome dynamics](http://cgap.nci.nih.gov/Pathways/BioCarta/m_akap95Pathway) | [Cdk1](http://www.ncbi.nlm.nih.gov/entrez/query.fcgi?cmd=search&db=gene&term=Cdk1) | cyclin-dependent kinase 1 | 0.0013225 | 246.97 | 218.93 | 1.13 |
| 5 |  | [AKAP95 role in mitosis and chromosome dynamics](http://cgap.nci.nih.gov/Pathways/BioCarta/m_akap95Pathway) | [Prkacb](http://www.ncbi.nlm.nih.gov/entrez/query.fcgi?cmd=search&db=gene&term=Prkacb) | protein kinase, cAMP dependent, catalytic, beta | 0.0020132 | 210.45 | 238.56 | 0.88 |
| 1 | m_mPRPathway | [How Progesterone Initiates the Oocyte Maturation](http://cgap.nci.nih.gov/Pathways/BioCarta/m_mPRPathway) | [Cap1](http://www.ncbi.nlm.nih.gov/entrez/query.fcgi?cmd=search&db=gene&term=Cap1) | CAP, adenylate cyclase-associated protein 1 (yeast) | 4.3e-06 | 464.38 | 334.44 | 1.39 |
| 2 |  | [How Progesterone Initiates the Oocyte Maturation](http://cgap.nci.nih.gov/Pathways/BioCarta/m_mPRPathway) | [Ccnb1](http://www.ncbi.nlm.nih.gov/entrez/query.fcgi?cmd=search&db=gene&term=Ccnb1) | cyclin B1 | 0.0002607 | 155.23 | 132.48 | 1.17 |
| 3 |  | [How Progesterone Initiates the Oocyte Maturation](http://cgap.nci.nih.gov/Pathways/BioCarta/m_mPRPathway) | [Cdk1](http://www.ncbi.nlm.nih.gov/entrez/query.fcgi?cmd=search&db=gene&term=Cdk1) | cyclin-dependent kinase 1 | 0.0013225 | 246.97 | 218.93 | 1.13 |
| 4 |  | [How Progesterone Initiates the Oocyte Maturation](http://cgap.nci.nih.gov/Pathways/BioCarta/m_mPRPathway) | [Prkacb](http://www.ncbi.nlm.nih.gov/entrez/query.fcgi?cmd=search&db=gene&term=Prkacb) | protein kinase, cAMP dependent, catalytic, beta | 0.0020132 | 210.45 | 238.56 | 0.88 |
| 5 |  | [How Progesterone Initiates the Oocyte Maturation](http://cgap.nci.nih.gov/Pathways/BioCarta/m_mPRPathway) | [Cdc25c](http://www.ncbi.nlm.nih.gov/entrez/query.fcgi?cmd=search&db=gene&term=Cdc25c) | cell division cycle 25 homolog C (S. pombe) | 0.012068 | 55.1 | 49.55 | 1.11 |
| 6 |  | [How Progesterone Initiates the Oocyte Maturation](http://cgap.nci.nih.gov/Pathways/BioCarta/m_mPRPathway) | [Mapk1](http://www.ncbi.nlm.nih.gov/entrez/query.fcgi?cmd=search&db=gene&term=Mapk1) | mitogen-activated protein kinase 1 | 0.1003226 | 196.98 | 246.1 | 0.8 |
| 1 | m_fibrinolysisPathway | [Fibrinolysis Pathway](http://cgap.nci.nih.gov/Pathways/BioCarta/m_fibrinolysisPathway) | [Serpine1](http://www.ncbi.nlm.nih.gov/entrez/query.fcgi?cmd=search&db=gene&term=Serpine1) | serine (or cysteine) peptidase inhibitor, clade E, member 1 | < 1e-07 | 20.35 | 107.28 | 0.19 |
| 2 |  | [Fibrinolysis Pathway](http://cgap.nci.nih.gov/Pathways/BioCarta/m_fibrinolysisPathway) | [F2r](http://www.ncbi.nlm.nih.gov/entrez/query.fcgi?cmd=search&db=gene&term=F2r) | coagulation factor II (thrombin) receptor | 0.0003161 | 222.57 | 179.83 | 1.24 |
| 3 |  | [Fibrinolysis Pathway](http://cgap.nci.nih.gov/Pathways/BioCarta/m_fibrinolysisPathway) | [F13a1](http://www.ncbi.nlm.nih.gov/entrez/query.fcgi?cmd=search&db=gene&term=F13a1) | coagulation factor XIII, A1 subunit | 0.0085206 | 12.37 | 10.43 | 1.19 |
| 4 |  | [Fibrinolysis Pathway](http://cgap.nci.nih.gov/Pathways/BioCarta/m_fibrinolysisPathway) | [Serpinb2](http://www.ncbi.nlm.nih.gov/entrez/query.fcgi?cmd=search&db=gene&term=Serpinb2) | serine (or cysteine) peptidase inhibitor, clade B, member 2 | 0.0393281 | 1408.47 | 1553.16 | 0.91 |
| 5 |  | [Fibrinolysis Pathway](http://cgap.nci.nih.gov/Pathways/BioCarta/m_fibrinolysisPathway) | [Fgb](http://www.ncbi.nlm.nih.gov/entrez/query.fcgi?cmd=search&db=gene&term=Fgb) | fibrinogen beta chain | 0.8205121 | 9.77 | 9.95 | 0.98 |
| 1 | m_cd40Pathway | [CD40L Signaling Pathway](http://cgap.nci.nih.gov/Pathways/BioCarta/m_cd40Pathway) | [Dusp1](http://www.ncbi.nlm.nih.gov/entrez/query.fcgi?cmd=search&db=gene&term=Dusp1) | dual specificity phosphatase 1 | < 1e-07 | 2280.02 | 1513.19 | 1.51 |
| 2 |  | [CD40L Signaling Pathway](http://cgap.nci.nih.gov/Pathways/BioCarta/m_cd40Pathway) | [Nfkb1](http://www.ncbi.nlm.nih.gov/entrez/query.fcgi?cmd=search&db=gene&term=Nfkb1) | nuclear factor of kappa light polypeptide gene enhancer in B-cells 1, p105 | 0.0063419 | 708.56 | 636.36 | 1.11 |
| 3 |  | [CD40L Signaling Pathway](http://cgap.nci.nih.gov/Pathways/BioCarta/m_cd40Pathway) | [Nfkbia](http://www.ncbi.nlm.nih.gov/entrez/query.fcgi?cmd=search&db=gene&term=Nfkbia) | nuclear factor of kappa light polypeptide gene enhancer in B-cells inhibitor, alpha | 0.011393 | 1283.3 | 1140.45 | 1.13 |
| 4 |  | [CD40L Signaling Pathway](http://cgap.nci.nih.gov/Pathways/BioCarta/m_cd40Pathway) | [Cd40lg](http://www.ncbi.nlm.nih.gov/entrez/query.fcgi?cmd=search&db=gene&term=Cd40lg) | CD40 ligand | 0.0275698 | 34.93 | 30.36 | 1.15 |
| 5 |  | [CD40L Signaling Pathway](http://cgap.nci.nih.gov/Pathways/BioCarta/m_cd40Pathway) | [Tnfaip3](http://www.ncbi.nlm.nih.gov/entrez/query.fcgi?cmd=search&db=gene&term=Tnfaip3) | tumor necrosis factor, alpha-induced protein 3 | 0.0331905 | 80.55 | 70.87 | 1.14 |
| 1 | m_igf1rPathway | [Multiple antiapoptotic pathways from IGF-1R signaling lead to BAD phosphorylation](http://cgap.nci.nih.gov/Pathways/BioCarta/m_igf1rPathway) | [Irs1](http://www.ncbi.nlm.nih.gov/entrez/query.fcgi?cmd=search&db=gene&term=Irs1) | insulin receptor substrate 1 | 3.3e-05 | 652.96 | 495.34 | 1.32 |
| 2 |  | [Multiple antiapoptotic pathways from IGF-1R signaling lead to BAD phosphorylation](http://cgap.nci.nih.gov/Pathways/BioCarta/m_igf1rPathway) | [Sos1](http://www.ncbi.nlm.nih.gov/entrez/query.fcgi?cmd=search&db=gene&term=Sos1) | son of sevenless homolog 1 (Drosophila) | 0.0001904 | 82.16 | 62.98 | 1.3 |
| 3 |  | [Multiple antiapoptotic pathways from IGF-1R signaling lead to BAD phosphorylation](http://cgap.nci.nih.gov/Pathways/BioCarta/m_igf1rPathway) | [Prkacb](http://www.ncbi.nlm.nih.gov/entrez/query.fcgi?cmd=search&db=gene&term=Prkacb) | protein kinase, cAMP dependent, catalytic, beta | 0.0020132 | 210.45 | 238.56 | 0.88 |
| 4 |  | [Multiple antiapoptotic pathways from IGF-1R signaling lead to BAD phosphorylation](http://cgap.nci.nih.gov/Pathways/BioCarta/m_igf1rPathway) | [Pik3r1](http://www.ncbi.nlm.nih.gov/entrez/query.fcgi?cmd=search&db=gene&term=Pik3r1) | phosphatidylinositol 3-kinase, regulatory subunit, polypeptide 1 (p85 alpha) | 0.0048997 | 134.94 | 113.16 | 1.19 |
| 5 |  | [Multiple antiapoptotic pathways from IGF-1R signaling lead to BAD phosphorylation](http://cgap.nci.nih.gov/Pathways/BioCarta/m_igf1rPathway) | [Igf1r](http://www.ncbi.nlm.nih.gov/entrez/query.fcgi?cmd=search&db=gene&term=Igf1r) | insulin-like growth factor I receptor | 0.0098994 | 33.81 | 28.83 | 1.17 |
| 6 |  | [Multiple antiapoptotic pathways from IGF-1R signaling lead to BAD phosphorylation](http://cgap.nci.nih.gov/Pathways/BioCarta/m_igf1rPathway) | [Mapk1](http://www.ncbi.nlm.nih.gov/entrez/query.fcgi?cmd=search&db=gene&term=Mapk1) | mitogen-activated protein kinase 1 | 0.1003226 | 196.98 | 246.1 | 0.8 |
| 1 | m_vipPathway | [Neuropeptides VIP and PACAP inhibit the apoptosis of activated T cells](http://cgap.nci.nih.gov/Pathways/BioCarta/m_vipPathway) | [Egr2](http://www.ncbi.nlm.nih.gov/entrez/query.fcgi?cmd=search&db=gene&term=Egr2) | early growth response 2 | 1.06e-05 | 807.09 | 620.73 | 1.3 |
| 2 |  | [Neuropeptides VIP and PACAP inhibit the apoptosis of activated T cells](http://cgap.nci.nih.gov/Pathways/BioCarta/m_vipPathway) | [Prkacb](http://www.ncbi.nlm.nih.gov/entrez/query.fcgi?cmd=search&db=gene&term=Prkacb) | protein kinase, cAMP dependent, catalytic, beta | 0.0020132 | 210.45 | 238.56 | 0.88 |
| 3 |  | [Neuropeptides VIP and PACAP inhibit the apoptosis of activated T cells](http://cgap.nci.nih.gov/Pathways/BioCarta/m_vipPathway) | [Nfkb1](http://www.ncbi.nlm.nih.gov/entrez/query.fcgi?cmd=search&db=gene&term=Nfkb1) | nuclear factor of kappa light polypeptide gene enhancer in B-cells 1, p105 | 0.0063419 | 708.56 | 636.36 | 1.11 |
| 4 |  | [Neuropeptides VIP and PACAP inhibit the apoptosis of activated T cells](http://cgap.nci.nih.gov/Pathways/BioCarta/m_vipPathway) | [Nfkbia](http://www.ncbi.nlm.nih.gov/entrez/query.fcgi?cmd=search&db=gene&term=Nfkbia) | nuclear factor of kappa light polypeptide gene enhancer in B-cells inhibitor, alpha | 0.011393 | 1283.3 | 1140.45 | 1.13 |
| 5 |  | [Neuropeptides VIP and PACAP inhibit the apoptosis of activated T cells](http://cgap.nci.nih.gov/Pathways/BioCarta/m_vipPathway) | [Nfatc2](http://www.ncbi.nlm.nih.gov/entrez/query.fcgi?cmd=search&db=gene&term=Nfatc2) | nuclear factor of activated T-cells, cytoplasmic, calcineurin-dependent 2 | 0.0344999 | 17.55 | 14.9 | 1.18 |
| 1 | m_plateletAppPathway | [Platelet Amyloid Precursor Protein Pathway](http://cgap.nci.nih.gov/Pathways/BioCarta/m_plateletAppPathway) | [Serpine1](http://www.ncbi.nlm.nih.gov/entrez/query.fcgi?cmd=search&db=gene&term=Serpine1) | serine (or cysteine) peptidase inhibitor, clade E, member 1 | < 1e-07 | 20.35 | 107.28 | 0.19 |
| 2 |  | [Platelet Amyloid Precursor Protein Pathway](http://cgap.nci.nih.gov/Pathways/BioCarta/m_plateletAppPathway) | [Col4a6](http://www.ncbi.nlm.nih.gov/entrez/query.fcgi?cmd=search&db=gene&term=Col4a6) | collagen, type IV, alpha 6 | 0.000604 | 147.67 | 123.11 | 1.2 |
| 3 |  | [Platelet Amyloid Precursor Protein Pathway](http://cgap.nci.nih.gov/Pathways/BioCarta/m_plateletAppPathway) | [Col4a5](http://www.ncbi.nlm.nih.gov/entrez/query.fcgi?cmd=search&db=gene&term=Col4a5) | collagen, type IV, alpha 5 | 0.002788 | 103.88 | 87.8 | 1.18 |
| 4 |  | [Platelet Amyloid Precursor Protein Pathway](http://cgap.nci.nih.gov/Pathways/BioCarta/m_plateletAppPathway) | [Col4a1](http://www.ncbi.nlm.nih.gov/entrez/query.fcgi?cmd=search&db=gene&term=Col4a1) | collagen, type IV, alpha 1 | 0.0224983 | 96.53 | 111.84 | 0.86 |
| 5 |  | [Platelet Amyloid Precursor Protein Pathway](http://cgap.nci.nih.gov/Pathways/BioCarta/m_plateletAppPathway) | [Col4a2](http://www.ncbi.nlm.nih.gov/entrez/query.fcgi?cmd=search&db=gene&term=Col4a2) | collagen, type IV, alpha 2 | 0.4762027 | 73.17 | 76.39 | 0.96 |
| 6 |  | [Platelet Amyloid Precursor Protein Pathway](http://cgap.nci.nih.gov/Pathways/BioCarta/m_plateletAppPathway) | [Col4a3](http://www.ncbi.nlm.nih.gov/entrez/query.fcgi?cmd=search&db=gene&term=Col4a3) | collagen, type IV, alpha 3 | 0.874094 | 20.64 | 20.46 | 1.01 |
| 1 | m_aktPathway | [AKT Signaling Pathway](http://cgap.nci.nih.gov/Pathways/BioCarta/m_aktPathway) | [Pdpk1](http://www.ncbi.nlm.nih.gov/entrez/query.fcgi?cmd=search&db=gene&term=Pdpk1) | 3-phosphoinositide dependent protein kinase-1 | 0.0002312 | 327.81 | 398.1 | 0.82 |
| 2 |  | [AKT Signaling Pathway](http://cgap.nci.nih.gov/Pathways/BioCarta/m_aktPathway) | [Hsp90aa1](http://www.ncbi.nlm.nih.gov/entrez/query.fcgi?cmd=search&db=gene&term=Hsp90aa1) | heat shock protein 90, alpha (cytosolic), class A member 1 | 0.0015541 | 697.45 | 916.44 | 0.76 |
| 3 |  | [AKT Signaling Pathway](http://cgap.nci.nih.gov/Pathways/BioCarta/m_aktPathway) | [Pik3r1](http://www.ncbi.nlm.nih.gov/entrez/query.fcgi?cmd=search&db=gene&term=Pik3r1) | phosphatidylinositol 3-kinase, regulatory subunit, polypeptide 1 (p85 alpha) | 0.0048997 | 134.94 | 113.16 | 1.19 |
| 4 |  | [AKT Signaling Pathway](http://cgap.nci.nih.gov/Pathways/BioCarta/m_aktPathway) | [Nfkb1](http://www.ncbi.nlm.nih.gov/entrez/query.fcgi?cmd=search&db=gene&term=Nfkb1) | nuclear factor of kappa light polypeptide gene enhancer in B-cells 1, p105 | 0.0063419 | 708.56 | 636.36 | 1.11 |
| 5 |  | [AKT Signaling Pathway](http://cgap.nci.nih.gov/Pathways/BioCarta/m_aktPathway) | [Gh](http://www.ncbi.nlm.nih.gov/entrez/query.fcgi?cmd=search&db=gene&term=Gh) | growth hormone | 0.0082185 | 57.36 | 69.3 | 0.83 |
| 6 |  | [AKT Signaling Pathway](http://cgap.nci.nih.gov/Pathways/BioCarta/m_aktPathway) | [Nfkbia](http://www.ncbi.nlm.nih.gov/entrez/query.fcgi?cmd=search&db=gene&term=Nfkbia) | nuclear factor of kappa light polypeptide gene enhancer in B-cells inhibitor, alpha | 0.011393 | 1283.3 | 1140.45 | 1.13 |
| 1 | m_GATA3pathway | [GATA3 participate in activating the Th2 cytokine genes expression](http://cgap.nci.nih.gov/Pathways/BioCarta/m_GATA3pathway) | [Mapk14](http://www.ncbi.nlm.nih.gov/entrez/query.fcgi?cmd=search&db=gene&term=Mapk14) | mitogen-activated protein kinase 14 | 0.0009873 | 79.08 | 55 | 1.44 |
| 2 |  | [GATA3 participate in activating the Th2 cytokine genes expression](http://cgap.nci.nih.gov/Pathways/BioCarta/m_GATA3pathway) | [Prkacb](http://www.ncbi.nlm.nih.gov/entrez/query.fcgi?cmd=search&db=gene&term=Prkacb) | protein kinase, cAMP dependent, catalytic, beta | 0.0020132 | 210.45 | 238.56 | 0.88 |
| 3 |  | [GATA3 participate in activating the Th2 cytokine genes expression](http://cgap.nci.nih.gov/Pathways/BioCarta/m_GATA3pathway) | [Il4](http://www.ncbi.nlm.nih.gov/entrez/query.fcgi?cmd=search&db=gene&term=Il4) | interleukin 4 | 0.004304 | 12.73 | 9.62 | 1.32 |
| 4 |  | [GATA3 participate in activating the Th2 cytokine genes expression](http://cgap.nci.nih.gov/Pathways/BioCarta/m_GATA3pathway) | [Gata3](http://www.ncbi.nlm.nih.gov/entrez/query.fcgi?cmd=search&db=gene&term=Gata3) | GATA binding protein 3 | 0.0129284 | 1814.84 | 1542.03 | 1.18 |
| 5 |  | [GATA3 participate in activating the Th2 cytokine genes expression](http://cgap.nci.nih.gov/Pathways/BioCarta/m_GATA3pathway) | [Nfatc2](http://www.ncbi.nlm.nih.gov/entrez/query.fcgi?cmd=search&db=gene&term=Nfatc2) | nuclear factor of activated T-cells, cytoplasmic, calcineurin-dependent 2 | 0.0344999 | 17.55 | 14.9 | 1.18 |
| 1 | m_erkPathway | [Erk1/Erk2 Mapk Signaling pathway](http://cgap.nci.nih.gov/Pathways/BioCarta/m_erkPathway) | [Sos1](http://www.ncbi.nlm.nih.gov/entrez/query.fcgi?cmd=search&db=gene&term=Sos1) | son of sevenless homolog 1 (Drosophila) | 0.0001904 | 82.16 | 62.98 | 1.3 |
| 2 |  | [Erk1/Erk2 Mapk Signaling pathway](http://cgap.nci.nih.gov/Pathways/BioCarta/m_erkPathway) | [Mknk2](http://www.ncbi.nlm.nih.gov/entrez/query.fcgi?cmd=search&db=gene&term=Mknk2) | MAP kinase-interacting serine/threonine kinase 2 | 0.0003662 | 360.68 | 284.54 | 1.27 |
| 3 |  | [Erk1/Erk2 Mapk Signaling pathway](http://cgap.nci.nih.gov/Pathways/BioCarta/m_erkPathway) | [Igf1r](http://www.ncbi.nlm.nih.gov/entrez/query.fcgi?cmd=search&db=gene&term=Igf1r) | insulin-like growth factor I receptor | 0.0098994 | 33.81 | 28.83 | 1.17 |
| 4 |  | [Erk1/Erk2 Mapk Signaling pathway](http://cgap.nci.nih.gov/Pathways/BioCarta/m_erkPathway) | [Ngf](http://www.ncbi.nlm.nih.gov/entrez/query.fcgi?cmd=search&db=gene&term=Ngf) | nerve growth factor | 0.0104602 | 41.98 | 56.89 | 0.74 |
| 5 |  | [Erk1/Erk2 Mapk Signaling pathway](http://cgap.nci.nih.gov/Pathways/BioCarta/m_erkPathway) | [Ngfr](http://www.ncbi.nlm.nih.gov/entrez/query.fcgi?cmd=search&db=gene&term=Ngfr) | nerve growth factor receptor (TNFR superfamily, member 16) | 0.0489613 | 243.71 | 230.03 | 1.06 |
| 6 |  | [Erk1/Erk2 Mapk Signaling pathway](http://cgap.nci.nih.gov/Pathways/BioCarta/m_erkPathway) | [Mapk1](http://www.ncbi.nlm.nih.gov/entrez/query.fcgi?cmd=search&db=gene&term=Mapk1) | mitogen-activated protein kinase 1 | 0.1003226 | 196.98 | 246.1 | 0.8 |
| 1 | m_pcafpathway | [The information-processing pathway at the IFN-beta enhancer](http://cgap.nci.nih.gov/Pathways/BioCarta/m_pcafpathway) | [Jun](http://www.ncbi.nlm.nih.gov/entrez/query.fcgi?cmd=search&db=gene&term=Jun) | Jun oncogene | 0.0013368 | 325.38 | 270.82 | 1.2 |
| 2 |  | [The information-processing pathway at the IFN-beta enhancer](http://cgap.nci.nih.gov/Pathways/BioCarta/m_pcafpathway) | [Irf3](http://www.ncbi.nlm.nih.gov/entrez/query.fcgi?cmd=search&db=gene&term=Irf3) | interferon regulatory factor 3 | 0.0035626 | 79.05 | 64.27 | 1.23 |
| 3 |  | [The information-processing pathway at the IFN-beta enhancer](http://cgap.nci.nih.gov/Pathways/BioCarta/m_pcafpathway) | [Nfkb1](http://www.ncbi.nlm.nih.gov/entrez/query.fcgi?cmd=search&db=gene&term=Nfkb1) | nuclear factor of kappa light polypeptide gene enhancer in B-cells 1, p105 | 0.0063419 | 708.56 | 636.36 | 1.11 |
| 4 |  | [The information-processing pathway at the IFN-beta enhancer](http://cgap.nci.nih.gov/Pathways/BioCarta/m_pcafpathway) | [Kat2b](http://www.ncbi.nlm.nih.gov/entrez/query.fcgi?cmd=search&db=gene&term=Kat2b) | K(lysine) acetyltransferase 2B | 0.011407 | 38.06 | 32.44 | 1.17 |
| 5 |  | [The information-processing pathway at the IFN-beta enhancer](http://cgap.nci.nih.gov/Pathways/BioCarta/m_pcafpathway) | [Irf1](http://www.ncbi.nlm.nih.gov/entrez/query.fcgi?cmd=search&db=gene&term=Irf1) | interferon regulatory factor 1 | 0.0456713 | 284.61 | 302.47 | 0.94 |
| 1 | m_HivnefPathway | [HIV-I Nef: negative effector of Fas and TNF](http://cgap.nci.nih.gov/Pathways/BioCarta/m_HivnefPathway) | [Dffa](http://www.ncbi.nlm.nih.gov/entrez/query.fcgi?cmd=search&db=gene&term=Dffa) | DNA fragmentation factor, alpha subunit | 7.09e-05 | 1144.1 | 901.2 | 1.27 |
| 2 |  | [HIV-I Nef: negative effector of Fas and TNF](http://cgap.nci.nih.gov/Pathways/BioCarta/m_HivnefPathway) | [Prkcd](http://www.ncbi.nlm.nih.gov/entrez/query.fcgi?cmd=search&db=gene&term=Prkcd) | protein kinase C, delta | 0.0001569 | 920.89 | 754.59 | 1.22 |
| 3 |  | [HIV-I Nef: negative effector of Fas and TNF](http://cgap.nci.nih.gov/Pathways/BioCarta/m_HivnefPathway) | [Lmna](http://www.ncbi.nlm.nih.gov/entrez/query.fcgi?cmd=search&db=gene&term=Lmna) | lamin A | 0.0035417 | 3780.19 | 3448.81 | 1.1 |
| 4 |  | [HIV-I Nef: negative effector of Fas and TNF](http://cgap.nci.nih.gov/Pathways/BioCarta/m_HivnefPathway) | [Nfkb1](http://www.ncbi.nlm.nih.gov/entrez/query.fcgi?cmd=search&db=gene&term=Nfkb1) | nuclear factor of kappa light polypeptide gene enhancer in B-cells 1, p105 | 0.0063419 | 708.56 | 636.36 | 1.11 |
| 5 |  | [HIV-I Nef: negative effector of Fas and TNF](http://cgap.nci.nih.gov/Pathways/BioCarta/m_HivnefPathway) | [Nfkbia](http://www.ncbi.nlm.nih.gov/entrez/query.fcgi?cmd=search&db=gene&term=Nfkbia) | nuclear factor of kappa light polypeptide gene enhancer in B-cells inhibitor, alpha | 0.011393 | 1283.3 | 1140.45 | 1.13 |
| 6 |  | [HIV-I Nef: negative effector of Fas and TNF](http://cgap.nci.nih.gov/Pathways/BioCarta/m_HivnefPathway) | [Bag4](http://www.ncbi.nlm.nih.gov/entrez/query.fcgi?cmd=search&db=gene&term=Bag4) | BCL2-associated athanogene 4 | 0.0230985 | 80.53 | 90.63 | 0.89 |
| 7 |  | [HIV-I Nef: negative effector of Fas and TNF](http://cgap.nci.nih.gov/Pathways/BioCarta/m_HivnefPathway) | [Lmnb1](http://www.ncbi.nlm.nih.gov/entrez/query.fcgi?cmd=search&db=gene&term=Lmnb1) | lamin B1 | 0.1817273 | 40.19 | 36.29 | 1.11 |
| 8 |  | [HIV-I Nef: negative effector of Fas and TNF](http://cgap.nci.nih.gov/Pathways/BioCarta/m_HivnefPathway) | [Xiap](http://www.ncbi.nlm.nih.gov/entrez/query.fcgi?cmd=search&db=gene&term=Xiap) | X-linked inhibitor of apoptosis | 0.3312718 | 13.2 | 14.25 | 0.93 |
| 9 |  | [HIV-I Nef: negative effector of Fas and TNF](http://cgap.nci.nih.gov/Pathways/BioCarta/m_HivnefPathway) | [Bid](http://www.ncbi.nlm.nih.gov/entrez/query.fcgi?cmd=search&db=gene&term=Bid) | BH3 interacting domain death agonist | 0.3374486 | 79.7 | 76.31 | 1.04 |
| 10 |  | [HIV-I Nef: negative effector of Fas and TNF](http://cgap.nci.nih.gov/Pathways/BioCarta/m_HivnefPathway) | [Gsn](http://www.ncbi.nlm.nih.gov/entrez/query.fcgi?cmd=search&db=gene&term=Gsn) | gelsolin | 0.4007605 | 8.59 | 8.18 | 1.05 |
| 11 |  | [HIV-I Nef: negative effector of Fas and TNF](http://cgap.nci.nih.gov/Pathways/BioCarta/m_HivnefPathway) | [Mapk8](http://www.ncbi.nlm.nih.gov/entrez/query.fcgi?cmd=search&db=gene&term=Mapk8) | mitogen-activated protein kinase 8 | 0.4053373 | 29.15 | 31.06 | 0.94 |
| 12 |  | [HIV-I Nef: negative effector of Fas and TNF](http://cgap.nci.nih.gov/Pathways/BioCarta/m_HivnefPathway) | [Psen1](http://www.ncbi.nlm.nih.gov/entrez/query.fcgi?cmd=search&db=gene&term=Psen1) | presenilin 1 | 0.511973 | 94.7 | 99.58 | 0.95 |
| 13 |  | [HIV-I Nef: negative effector of Fas and TNF](http://cgap.nci.nih.gov/Pathways/BioCarta/m_HivnefPathway) | [Map3k5](http://www.ncbi.nlm.nih.gov/entrez/query.fcgi?cmd=search&db=gene&term=Map3k5) | mitogen-activated protein kinase kinase kinase 5 | 0.6487235 | 54.99 | 56.65 | 0.97 |
| 14 |  | [HIV-I Nef: negative effector of Fas and TNF](http://cgap.nci.nih.gov/Pathways/BioCarta/m_HivnefPathway) | [Tnf](http://www.ncbi.nlm.nih.gov/entrez/query.fcgi?cmd=search&db=gene&term=Tnf) | tumor necrosis factor | 0.8447753 | 58.77 | 59.58 | 0.99 |
| 15 |  | [HIV-I Nef: negative effector of Fas and TNF](http://cgap.nci.nih.gov/Pathways/BioCarta/m_HivnefPathway) | [Pak2](http://www.ncbi.nlm.nih.gov/entrez/query.fcgi?cmd=search&db=gene&term=Pak2) | p21 protein (Cdc42/Rac)-activated kinase 2 | 0.9876799 | 52.52 | 52.58 | 1 |

Class 1: *ko*; Class 2: *tg*.
