# Supplementary figures and images for: T Cell Leukemia/Lymphoma 1A is essential for mouse epidermal keratinocytes proliferation promoted by insulin-like growth factor 1
Source: PLoS One. 2018 Oct 4;13(10):e0204775. doi: 10.1371/journal.pone.0204775 (PMC6171881; doi:10.1371/journal.pone.0204775)

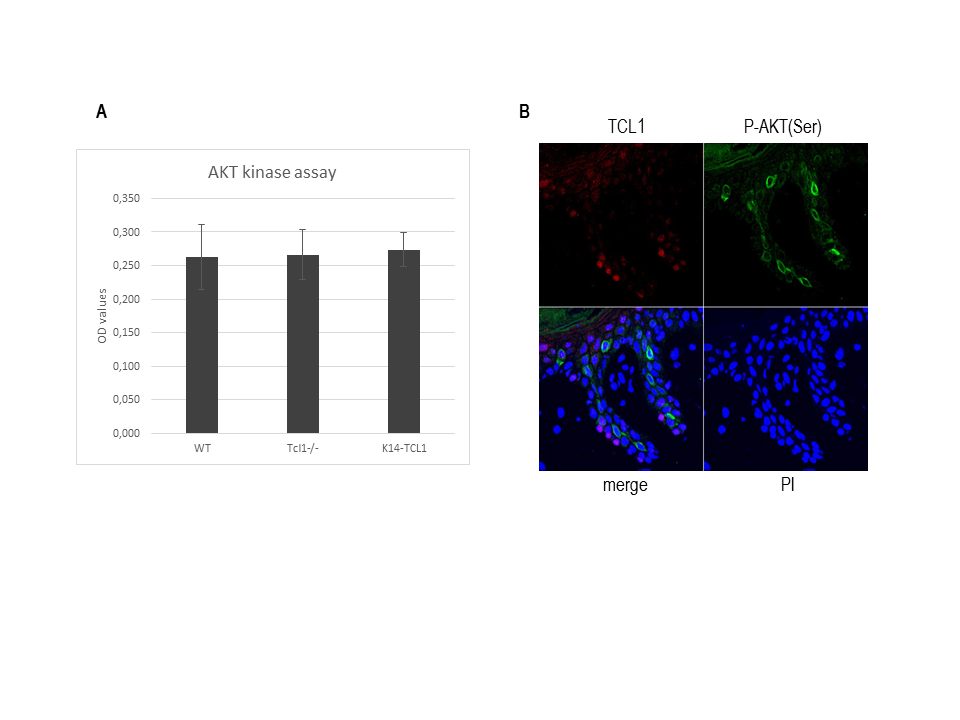

Supplement: S1 Fig — (A) Kinase assay was performed on keratinocyte lysates, according to manufacturer’s protocol. Graph represents mean OD values and standard error bars of three independent experiments. No differences were observed between the three genotypes. (B) Immunofluorescence for mouse TCL1 protein (red) and phosphorylated AKT(Ser473) (green). Nuclear counterstain is propidium iodide (PI, blue). Merging of red and blue fluorescence represents TCL1 nuclear localization (pink). Magnification: 40X. Images were acquired by confocal laser scanning. (TIF) [file pone.0204775.s013.tif]

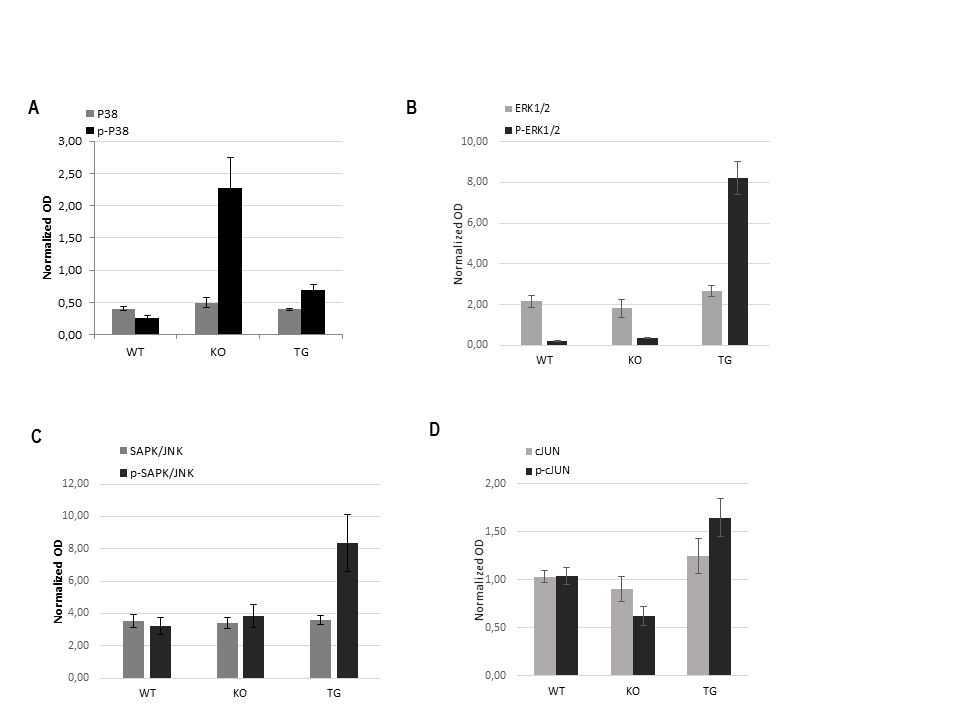

Supplement: S2 Fig — WB analysis of phosphorylated (dark gray) and entire (light gray) forms of P38 (A), ERK1/2 (B) SAPK/JNK MAPKs (C) and cJUN (D) on protein extracts from keratinocytes of WT, Tcl1-/- and K14-TCL1 mice. Tubulin alpha (αTUB) was used to normalize protein load. Normalized OD values are graphed as the mean and standard deviation of three independent experiments. (TIF) [file pone.0204775.s014.tif]
